# A non-tree-based comprehensive study of metazoan Hox and ParaHox genes prompts new insights into their origin and evolution

**DOI:** 10.1186/1471-2148-10-73

**Published:** 2010-03-11

**Authors:** Morgane Thomas-Chollier, Valérie Ledent, Luc Leyns, Michel Vervoort

**Affiliations:** 1 Laboratoire de Bioinformatique des Génomes et des Réseaux (BiGRe), Université Libre de Bruxelles, Campus Plaine, CP 263, Boulevard du Triomphe, B-1050 Bruxelles, Belgium; 2 Laboratory for Cell Genetics, Vrije Universiteit Brussel, Pleinlaan 2, B-1050 Brussels, Belgium; 3 Belgian EMBnet Node, Université Libre de Bruxelles, CP 257, Bd du Triomphe, B-1050 Brussels, Belgium; 4 Development and Neurobiology Program, Institut Jacques Monod, UMR 7592 CNRS/Université Paris Diderot - Paris 7, 15 rue Hélène Brion, 75205 Paris Cedex 13, France; 5 UFR des Sciences du Vivant, Université Paris Diderot - Paris 7, 5, rue Marie-Andrée Lagroua Weill-Hallé, 75205 Paris Cedex 13, France; 6 Current address: Department of Computational Molecular Biology, Max Planck Institute for Molecular Genetics, Ihnestrasse 73, 14195 Berlin, Germany

## Abstract

**Background:**

Hox and the closely-related ParaHox genes, which emerged prior to the divergence between cnidarians and bilaterians, are the most well-known members of the ancient genetic toolkit that controls embryonic development across all metazoans. Fundamental questions relative to their origin and evolutionary relationships remain however unresolved. We investigate here the evolution of metazoan Hox and ParaHox genes using the HoxPred program that allows the identification of Hox genes without the need of phylogenetic tree reconstructions.

**Results:**

We show that HoxPred provides an efficient and accurate classification of Hox and ParaHox genes in their respective homology groups, including Hox paralogous groups (PGs). We analyzed more than 10,000 sequences from 310 metazoan species, from 6 genome projects and the complete UniProtKB database. The HoxPred program and all results arranged in the Datab'Hox database are freely available at http://cege.vub.ac.be/hoxpred/. Results for the genome-scale studies are coherent with previous studies, and also brings knowledge on the Hox repertoire and clusters for newly-sequenced species. The unprecedented scale of this study and the use of a non-tree-based approach allows unresolved key questions about Hox and ParaHox genes evolution to be addressed.

**Conclusions:**

Our analysis suggests that the presence of a single type of Posterior Hox genes (PG9-like) is ancestral to bilaterians, and that new Posterior PGs would have arisen in deuterostomes through independent gene duplications. Four types of Central genes would also be ancestral to bilaterians, with two of them, PG6- and PG7-like that gave rise, in protostomes, to the UbdA- and ftz/Antp/Lox5-type genes, respectively. A fifth type of Central genes (PG8) would have emerged in the vertebrate lineage. Our results also suggest the presence of Anterior (PG1 and PG3), Central and Posterior Hox genes in the cnidarians, supporting an ancestral four-gene Hox cluster. In addition, our data support the relationship of the bilaterian ParaHox genes *Gsx *and *Xlox *with PG3, and *Cdx *with the Central genes. Our study therefore indicates three possible models for the origin of Hox and ParaHox in early metazoans, a two-gene (Anterior/PG3 - Central/Posterior), a three-gene (Anterior/PG1, Anterior/PG3 and Central/Posterior), or a four-gene (Anterior/PG1 - Anterior/PG3 - Central - Posterior) ProtoHox cluster.

## Background

Hox genes encode a large family of closely-related transcription factors from the homeobox class that is characterised by a 60 amino acids region called the homeodomain [1,2]. These genes play crucial roles in the development of metazoans, principally by controlling the patterning along the anteroposterior axis in a wide variety of animals (e.g. [3,4]). Their role in the tetrapod limb differentiation is also well-known (reviewed in [5]). Hox genes are usually organized in clusters whose genomic organization reflects domains of expression along the anteroposterior axis (spatial colinearity) [6], as well as, in some species, timing of expression during development (temporal colinearity) [7,8]. Members of this gene family have been reported in both bilaterians (animals presenting a bilateral symmetry) and cnidarians (group including sea anemones, corals, jellyfish), which suggests that Hox genes emerged prior to the divergence between bilaterians and cnidarians [9-12].

The ParaHox genes, *Gsx *(*genomic screened homeobox*), *Xlox *(*Xenopus laevis homeobox 8*) and *Cdx *(*Caudal-type homeobox*), are closely related to the Hox genes, and are also involved in developmental processes. Like the Hox genes, they encompass a homeodomain region and form a cluster, at least in chordates (the individual genes are present in non chordate species but are usually scattered in the genome) [13-16]. It is widely believed that the presence of a cluster of three ParaHox genes, although observed so far only in chordates, is ancestral to bilaterians [11,13-15,17-19].

Hox and ParaHox genes have been classified in homology groups, which serve as basis to study their evolutionary relationships [14,20,21] and infer their origin in early metazoans [9-12]. In vertebrates, Hox genes are classified in 14 Paralogous Groups (PGs) [22] that can themselves be grouped in broader classes, known as Anterior (PG1-3), Central (PG4-8) and Posterior (PG9-14) (e.g. [23]). In some studies, the PG3 proteins have been proposed to form a 4th independent class (e.g. [14]), although their homeodomain shows a high similarity with that of PG2 proteins [18,24] (Additional file 1, Figure S1). Hox genes from non-vertebrate bilaterian species have been assigned to the aforementioned classes, suggesting that these classes represent ancient types of Hox genes. The ParaHox genes form three groups, named Gsx, Xlox and Cdx [13], yet, they are more closely related to some Hox PGs than to each other. *Gsx *genes have been reported to be closer to Hox Anterior group genes, *Xlox *to PG3 genes, and *Cdx *to Hox Posterior genes [13,25-27]. This has lead to the model that the Hox and ParaHox clusters arose through the duplication of a hypothetical ProtoHox cluster (reviewed in [18]).

Most studies aiming at understanding Hox and ParaHox gene evolution used phylogenetic tree reconstruction based on multiple alignments of their homeodomain. Such trees often lack resolution [28], thereby preventing a clear assignment of sequences in homology groups (e.g. [12,29]). Nodes of the trees frequently have low statistical support values that can be explained by the short length of the homeodomain (60 amino acids) and its strong conservation [23,30]. Different tree reconstruction methods may furthermore produce conflicting results, giving rise to controversial conclusions [12,27,31,32]. Complementary methods, such as sequence similarity, position of the genes in the cluster and Hox/ParaHox signatures [33,34], thus may provide crucial information about the evolution of these genes.

**Figure 1:**
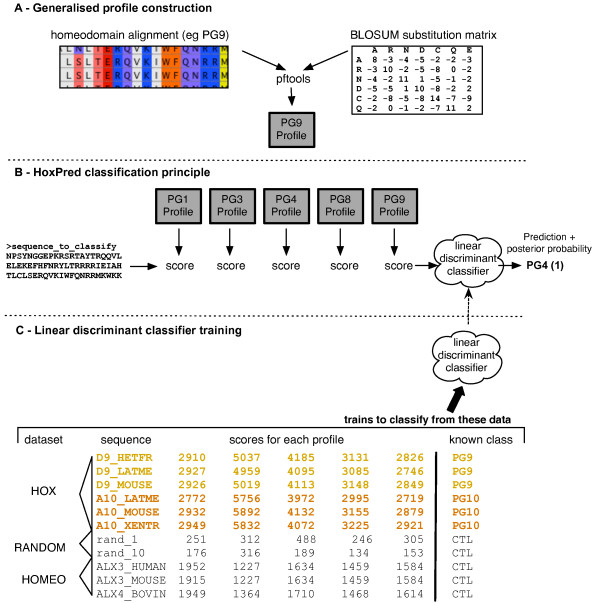
**HoxPred classification approach**. **A**. Generalised profile construction. A multiple alignment is built from a set of non-redundant homeodomain sequences that belong to a given homology group (PG9 for this illustration). This alignment then serves as input to a program from the pftools suite [62], which generates the corresponding generalised profile. This profile is a scoring matrix that allows to assign a score to a sequence, based on its similarity with the profile. Contrary to more simple pattern search technique, a profile can provide scores for residues that were not originally found at a given position of the motif. These scores are residue-specific, and extrapolated by using a substitution matrix when building the profile. **B**. HoxPred classification principle. The sequence to classify is scored by an optimal combination of profiles. The resulting vector of scores then serves as input to a discriminant function that has been previously trained to classify such a vector of scores into a specific class (eg PG4). **C**. Linear discriminant classifier training. The training phase aims at generating the discriminant function. The training dataset comprise sequences for which the class is known. They can be HOX, RANDOM or HOMEO sequences (see Materials and methods). All sequences are scored by the profiles, so that each sequence is represented by a vector of scores. The classifier is then trained to classify such vector of scores into their associated class (specified on the right). CTL is the control class (see Materials and methods).

HoxPred [35,36] is a Hox-dedicated program designed to classify Hox protein sequences, without phylogenetic reconstructions. Figure 1 illustrates the general approach; see [35] for a full description of the method. The underlying principle is an extension of the Hox signatures. However, instead of attempting to explicitly discover the few key positions that would define a given homology group, the homeodomain is considered in its entirety as a motif, and described as a generalised profile (Figure 1A). Optimal combinations of such profiles allow the classification of sequences, through a supervised classification approach (Figure 1B) in which discriminant functions are trained to assign sequences to predefined homology groups (Figure 1C). This technique thus differs from pattern search techniques (as used in [37] for homeobox sequences) where a sequence either matches or not a given pattern that describes qualitatively a motif. The profiles used here are quantitative motif descriptors that are more flexible [38], and that take into account amino acid substitutability for all positions of the motif in a residue-specific way i.e. substitution G to A is less penalized than G to W. The discriminant functions of HoxPred moreover allows to use the information of multiple profiles, which increases the accuracy of the predictions [35]. These discriminant functions finally return posterior probabilities for all possible homology groups, thereby providing confidence estimates for the predictions [35] (Figure 1B). HoxPred was originally designed for vertebrate sequences classification, and has already proven successful in clarifying the evolutionary history of the HoxC1a genes in teleost fish [39].

Although the origin and evolution of the Hox and ParaHox genes have been addressed by a huge number of studies over this last decade, several fundamental and unsolved questions remain [10,14,21,29,40,41]. How did this family emerge in early metazoans? What are the evolutionary relationships between Hox and ParaHox genes? How do cnidarian genes relate to the different classes of bilaterian Hox and ParaHox genes? How did Central and Posterior genes evolve among bilaterians?

We wondered whether a non-tree-based approach could shed light on these crucial questions. In this study, we re-analyzed all published sequence datasets and several newly-sequenced genomes using new versions of the HoxPred program. The extensive evaluation of HoxPred predictions confirmed its accuracy on bilaterian sequences. Its computational efficiency allowed us to simultaneously investigate 310 metazoan species accounting for more than 10,000 homeodomain genes, and infer evolutionary scenarios for the emergence of homologous groups. We found that among the Central Hox genes, PG4, PG5, PG6 and PG7 were likely present in the last common ancestor of bilaterians (Urbilateria). PG8 emerged in vertebrates. For the Posterior Hox genes, PG9 would have been present in Urbilateria; new paralogous groups then emerged in deuterostomes (group including echinoderms and chordates). PG14 appears in vertebrates, suggesting that the amphioxus *Hox14 *gene is not related to this PG14. Altogether, our results favor the independent duplication model over the 'Deuterostome Posterior Flexibility' model alone, for the evolution of the Posterior Hox genes. Regarding cnidarians, our results clearly indicate the presence of Central Hox genes, an observation which contradicts commonly-accepted views [12,27]. Our analysis of ParaHox genes indicates that *Gsx *is related to PG3 rather than to PG1, across all metazoans. The ParaHox *Cdx *gene would be closer to Central than to Posterior genes. The *Xlox *genes from bilaterians appear mostly related to PG3, while the few known cnidarian *Xlox *are closer to the Central group. The evolutionary scenario of *Xlox *thus remains unclear. Nevertheless, these results, coherent with our Hox analysis, suggest three possible models for the early evolution of Hox and ParaHox genes from an ancestral ProtoHox cluster.

## Results and Discussion

### HoxPred, an accurate tool to classify bilaterian Hox and ParaHox genes

We developed three new versions of HoxPred [36] to study the Hox and ParaHox genes at the scale of the metazoans (Table 1; see Materials and Methods for a description of these three versions). The "Bilateria" version aims at classifying Hox and ParaHox genes. The "Bilateria_relaxed" and "Vertebrate_relaxed" versions have been designed to study the evolutionary relationships between Hox and ParaHox genes. These versions were constructed and evaluated as in [35]. The prior probability for the control (CTL) group is very high in order to avoid misclassifications. HoxPred consequently shows tendency to classify divergent sequences into the CTL group, rather than any other group [35]. To assess the quality of the predictions, we applied the three new versions of HoxPred on a large set of 800 homeodomain sequences from 9 non-vertebrate species spanning various bilaterian phylogenetic groups, and which include well-characterized Hox and ParaHox genes (Additional file 2, Tables S1, S2, S3). Overall, the accuracy of all versions is high (Table 1). As expected, the "Bilateria" version provides an efficient and very stringent classification of Hox and ParaHox sequences (accuracy of 0.97): most Hox and ParaHox genes are correctly assigned to their class or group while non-Hox sequences are consistently classified in the control group. The two "relaxed" versions also provide correct predictions for Hox genes, with accuracy values higher than 0.90.

**Figure 2:**
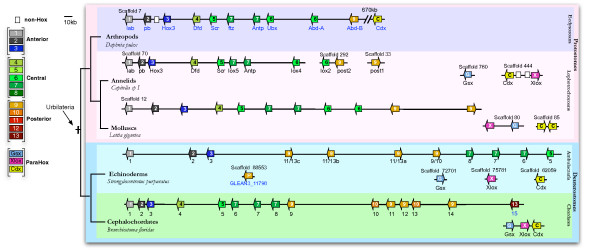
**Genomic organization of the Hox genes identified with HoxPred in the genome-scale analyses**. Hox and ParaHox genes are depicted with arrows indicating transcription orientation, over black lines representing the scaffolds. This representation takes into account the relative distance between the genes. The transcription orientation is the same as provided by the JGI genome browser. The color of the arrows relates to HoxPred classification (see color code on the left); white squares are non-Hox genes. The Hox cluster of *Strongylocentrotus *is from [46] and the ParaHox genes are from SpBase [63]. The Hox cluster of *Branchiostoma *is from [43], with the additional *Branchiostoma Hox15 *gene found in the genome assembly. *Hellobdella *genes are not indicated as they span many scaffolds, probably due to poor genome assembly. When available, gene names are specified: in black (from published studies [16,42,43,46]) or in blue (from the JGI genome browser or SpBase). An additional putative Hox gene, showing sequence similarities with *Sp-Hox11/13c*, lies outside the Hox cluster in *Strongylocentrotus*. See Additional file 2, Table S4 for the genomic coordinates.

To further investigate the evolution of Hox genes and the usefulness of HoxPred for its study, we applied the three new versions of HoxPred on a comprehensive dataset of 10,538 homeodomain sequences, from the UniprotKB database and 6 completely sequenced bilaterian genomes. To our knowledge, this is the first study on Hox sequences conducted on such a large scale. All results are freely available from the Datab'Hox database [36], through a friendly Web interface enabling complex queries and providing links to external databases. The HoxPred program is accessible on the same website [36]. Multiple alignments of Hox and ParaHox sequences ordered by PGs, are also available from this website and in the Additional file 3. Figure 2 and Additional file 2, Table S4 summarize the results obtained for the 6 species whose genome is completely sequenced: 3 lophotrochozoans (group including annelid worms and mollusks) (*Capitella sp. I, Helobdella robusta *and *Lottia gigantea*), 1 ecdysozoan (*Daphnia pulex*), and 2 deuterostomes (*Strongylocentrotus purpuratus *and *Branchiostoma floridae*). Results for *Capitella sp. I *and *Branchiostoma floridae *are coherent with published studies [42,43]. Of particular interest, we identified 11 Hox genes in the *Lottia *genome and found that these genes are clustered on a single scaffold and display the same orientation except for one of them, the last Posterior gene similar to *Capitella Post1*. Similarly, the 10 *Daphnia *genes are found on a single scaffold. A complex situation is found in *Helobdella *where 19 Hox genes are found on several different scaffolds. We also identified ParaHox genes (Figure 2 and Additional file 2, Table S4). While a single ParaHox gene (*Cdx*) is found in *Daphnia*, the 3 types of ParaHox genes are found in lophotrochozoans and deuterostomes (in agreement with previous studies [16,19,42]) and they are mostly organised into two- or three-genes clusters.

HoxPred therefore appears to be a suitable tool to identify Hox and ParaHox genes and we therefore used these identifications to address unsolved questions about the evolution of these genes.

**Table 1:** Evaluation of predictions for the four versions of HoxPred.

Reference	Version	Training Sequences	CTL group sequences	Homology groups	Accuracy
[35]	Vertebrate	Vertebrate	RANDOM + HOMEO	PG1-14 + orthologous groups	0.97

new	Bilateria	Bilateria	RANDOM + HOMEO	ANT/CENT/POST GSX/XLOX/CDX	0.97

new	Vertebrate_relaxed	Vertebrate	RANDOM	PG1-14	0.92

new	Bilateria_relaxed	Bilateria	RANDOM	ANT/CENT/POST	0.98

The Vertebrate version was not used in this study.

### A global model for the evolution of Posterior Hox genes

**Figure 3:**
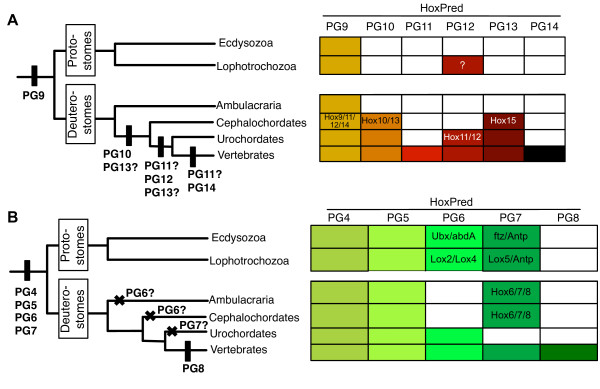
**Models for the evolution of Posterior and Central Hox genes in bilaterians**. **A**. Posterior Hox genes. The predicted PGs for each phylogenetic group are indicated with colors in the tables. Inside these table, the names of the genes are indicated when HoxPred predictions differ from their current annotation. The possible emergence of individual PGs are indicated on the schematic tree with vertical bars (only the PG content is considered, not the actual number of genes belonging to each PG, i.e. lineage-specific duplication and losses of individual genes are not indicated). Given that both protostomes and deuterostomes have PG9 predictions, it seems that a *Hox9 *gene was already present in Urbilateria. PG10 would have emerged in deuterostomes, in the lineage leading to chordates. After the divergence of cephalochordates, the lineage leading to urochordates and vertebrates would have acquired PG12. PG14 appeared in vertebrates. With respect to PG11, this group could have emerged either before or after the split between urochordates and vertebrates. Considering that both *Ciona intestinalis *and *Oikopleura dioica *have disintegrated clusters and likely miss PGs, we cannot exclude a possible loss of PG11 in urochordates. The emergence of PG13 is uncertain due to the prediction of the amphioxus *Hox15 *gene as PG13. It could either be early in the chordate lineage, or in the last common ancestor of urochordates and vertebrates. **B**. Central Hox genes. The possible emergence and loss of individual PGs are indicated on the schematic tree with vertical bars and crosses, respectively. Four Central PGs were present in Urbilateria (PG4, PG5, PG6 and PG7). PG6 and PG7 would have been independently lost within deuterostomes. PG8 emerged in vertebrates.

Posterior Hox genes of non-vertebrate bilaterians, including deuterostomes such as cephalochordates, urochordates, and ambulacraria (echinoderms and hemichordates), can not be confidently related to specific vertebrate PG using phylogenetic analyses [21,29,43,44] (Additional file 1, Figure S2). It has been therefore proposed that the blurred relationships between Hox Posterior genes would be explained by an accelerated evolution rate of these genes, a process called 'Deuterostome Posterior Flexibility' [29]. An alternative hypothesis suggests multiple independent duplications to shape the posterior portion of the Hox clusters [29,45,46]. We used HoxPred to analyse the bilaterian Posterior Hox genes (Figures 2, 3A and Additional file 2, Table S4).

HoxPred assigned all amphioxus Posterior genes to PG9 and PG10, to the exception of *Hox15*, predicted as PG13 (Figure 3A). The latter has not been classified into any particular homology group with phylogenetic approaches, but it was clustered with PG13 with low statistical values [47]. Our own phylogenetic analyses show that the amphioxus *Hox15 *clustered with PG13 sequences, with a high posterior probability of 0.97 for the Bayesian tree (Additional file 1, Figure S2). Our data therefore suggest that the amphioxus *Hox11-14 *genes would have arisen from duplications of *Hox9- *and *Hox10-like *genes, independent to those which produced the vertebrate PG11 to PG14 Posterior Hox genes. HoxPred prediction for the *Hox14 *gene thus brings additional support for an independent origin of the amphioxus and vertebrate Hox14 genes [47-50]. The assignment of *Hox15 *to PG13 suggests that a PG13 gene was present in the chordate ancestor. The intriguing interspersed order of the PG9 and PG10 genes (Figure 2) might be compatible with duplication of a *Hox9*-*Hox10 *genes segment. Comparison of surrounding non-coding sequence may give further insights into the duplication events.

In the case of the urochordate *Oikopleura dioica, Hox9 *and *Hox10 *were classified into the PG9 and PG10, respectively, while both *Hox11 *and *Hox12 *were assigned to PG12 (Figure 3A; in agreement with the published phylogenetic tree [27]). These genes should be respectively renamed, e.g. *Hox12a *and *Hox12b*. The ambulacraria posterior genes (9/10 and 11/13 groups) were all assigned to PG9. HoxPred also systematically classified ecdyosozoan Posterior Hox sequences in PG9. Finally, in lophotrochozans, the predictions were ambiguous: *Post1 *and *Post2 *both alternated between PG9, PG12 and CTL, likely because these sequences are quite divergent [51]. An alternative hypothesis is that this uncertainty reflects an affinity for both PG9 and PG12, already present in an ancestral bilaterian *PG9/PG12-like *posterior gene and retained in lophotrochozoans.

HoxPred predictions of posterior sequences have strong statistical support, with posterior probabilities similarly high as Anterior or Central sequences (Additional file 2, Tables S1, S4). However, as many of the predictions fall in the PG9 and PG10 groups, we conducted additional statistical tests (Additional file 1, Figure S3) to provide evidences that neither the PG9 nor PG10 acts as a general 'Hox class' that would attract Hox sequences irrespective of their real identity.

Our analysis of HoxPred assignments favors the hypothesis of multiple independent duplications over the 'Deuterostome Posterior Flexibility' hypothesis alone, and allows to propose a global model for Posterior genes evolution in bilaterians (Figure 3A). In this model, we have only considered the PG9 predictions for protostomes, since the PG12 predictions in lophotrochozoans do not seem consistent. These predictions could be artifactual and might not indicate the presence of a PG12 gene in the protostome ancestor. Although poorly parsimonious in terms of duplication events, our model is supported by (i) the fact that urochordates, rather than cephalochordates, would be closer to vertebrates [52], thereby challenging the view that the amphioxus Hox cluster is the archetypal cluster from which aroused the vertebrate clusters - interestingly, there are more HoxPred predictions in common between vertebrates and urochordates than with cephalochordates; (ii) Many gene families, such as the bHLH family of transcription factors, have undergone multiple duplications specifically in the amphioxus [47,53]; (iii) Deuterostome Posterior Flexibity has been questioned in ambulacraria [44,45]; (iv) The amphioxus and vertebrate *Hox14 *genes do not group together in phylogenetic trees [48].

### The bilaterian Central genes enigma

While evolutionary relationships between Central genes from PG4 and PG5 across bilaterians were quite well resolved, phylogenetic approaches failed to decipher the relationships between the other three Central genes, eventually classified in a single broad PG6-8 group [21,44,46]. HoxPred predictions for PG4 and PG5 were consistent with tree-based annotation (Figure 3B and Additional file 2, Table S4). For PG6-8, we found that ecdysozoan and lophotrochozoan genes are predicted into PG6 and PG7, with a strong tendency towards *Ubx, abd-A, Lox2 *and *Lox4 *predicted as PG6, and *ftz, Antp *and *Lox5 *predicted as PG7 (Figure 3B and Additional file 2, Table S4). Ambulacraria and cephalochordates predictions are restricted to PG7, whereas urochordates only have PG6 predictions. Vertebrates is the only phylogenetic group having PG8 predictions, in addition to PG6 and PG7. These data suggest that PG4, PG5, PG6 and PG7 would have been present in the last common ancestor of all bilaterians (*Urbilateria*), and PG8 would have emerged in Vertebrates. The deuterostome predictions call for caution, as they would imply a loss of PG6 along with an expansion of PG7 genes in both Ambulacraria and cephalochordates, and a loss of PG7 in urochordates, a scenario which seems poorly parsimonious. However, urochordates have clearly lost members of many gene families [53,54] and similar poorly parsimonious scenarios have been proposed for other genes families, for example the *iroquois/Irx *genes [55].

### Identification of Hox genes in Cnidaria

**Figure 4:**
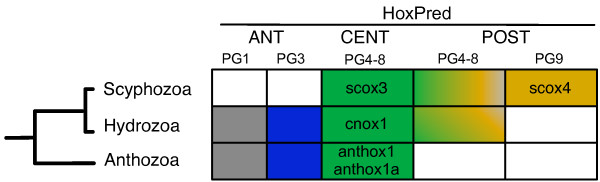
**Summary of HoxPred predictions in cnidarians**. The predicted homology group are indicated with colors in the table. ANT, CENT and POST predictions were obtained with the "Bilateria_relaxed" version, while the PG predictions were obtained with the "Vertebrate_relaxed" version.

Reconstructing the Hox repertoire of the Cnidaria/Bilateria ancestor is a notorious challenge, as cnidarian 'Hox-like' genes are difficult to relate to the bilaterian homologous groups with traditionnal sequence similarity-based or phylogenetic analyses. The various phylogenetic studies published so far yielded a somewhat confuse picture [12,27,31,32,56]. We analysed the HoxPred assignments for all the homeobox genes from the fully sequenced *Nematostella vectensis *genome, as well as for 37 additional homeodomain sequences, from 11 other cnidarian species (Figure 4 and Additional file 2, Table S5). Using the "Bilateria" version of HoxPred, we found that most cnidarian Hox and ParaHox genes were classified as CTL, at the exception of a few Anterior and Gsx genes. This is not surprising given the divergent nature of the cnidarian Hox and ParaHox genes with respect to those from bilaterians. The "relaxed" versions of HoxPred, however, allow to classify cnidarian genes. The cnidarian Hox genes fall in Anterior, Central and Posterior groups predictions which strongly contradict the commonly accepted idea of a lack of Central Hox genes in Cnidaria [12,27], but see alternative hypotheses [10,56]. Genes predicted as Central include *anthox1 *and *anthox1a *from the anthozoan *Nematostella*, which have been difficult to relate to a bilaterian group of homology, considered as either Central/Posterior [56], Posterior [12], cnidarian-specific [27] or even non-Hox [31]. Still, they are usually considered as non-anterior Hox genes [12,27,31,56]; their classification by HoxPred in the Central group is therefore in agreement with this view. Predictions as Central genes also encompass genes from hydrozoans (e.g. *cnox1 *from *Eleutheria dichotoma*) and a scyphozoan (*scox3 *from *Cassiopea xamachana*). Within the Anterior group, we found predictions for PG1 and PG3 that corroborate phylogenetic analyses [18,27], but not for PG2 in contradiction to what has been reported in [12,32]. Predictions as Posterior genes, only found in hydrozoans and scyphozoans, are compatible with previous assignments [18,32]. However, most of the genes predicted as Posterior by the "Bilateria_relaxed" version of HoxPred are classified as Central genes with the "Vertebrate_relaxed" version, apart from *scox4 *that is predicted as Posterior by the two "relaxed" versions. This uncertainty may reflect that these genes may have arisen from an ancestral Central/Posterior gene.

**Figure 5:**
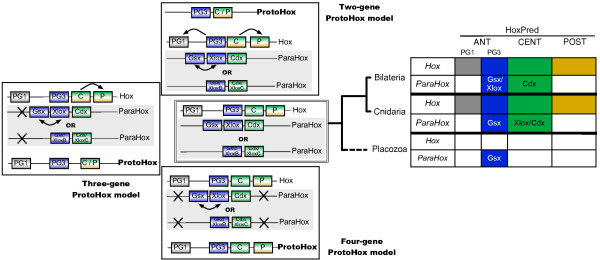
**Models for the early evolution of Hox and ParaHox genes**. The predicted homology groups for each phylogenetic group are indicated with colors in the table. The uncertainty of the phylogenetic position of placozoans is indicated by a dashed line [64,65]. The cnidarian/bilaterian ancestor inferred Hox-ParaHox repertoire is depicted in a double box. Posterior genes are depicted with two colors to reflect the uncertainty of predictions into Central and Posterior groups. This repertoire would result from three equally parsimonious scenarios: a two-gene ProtoHox cluster composed of ancestral Anterior/PG3 and Central (or Central/Posterior) genes, undergoing two or three duplications; a three-gene ProtoHox cluster composed of ancestral Anterior/PG1, Anterior/PG3 and Central (or Central/Posterior) genes undergoing one gene loss, and one or two duplication; or a four-gene ProtoHox cluster composed of ancestral Anterior/PG1, Anterior/PG3, Central and Posterior genes, undergoing two gene losses and a possible duplication.

In summary, our analysis indicates that cnidarians would possess three to four types of Hox genes, namely Anterior PG1, Anterior PG3, Central, and Posterior (or Central/Posterior) and therefore suggest that these three to four categories of Hox genes were already present in the last common ancestor of cnidarians and bilaterians (Figure 5).

### ParaHox predictions: implications for the ProtoHox models

The ParaHox cluster of genes has long been supposed to be the sister cluster of the Hox cluster, with the *Gsx, Xlox *and *Cdx *genes corresponding to the Anterior, PG3 and Posterior groups, respectively [13]. This view has been recently challenged by analyses of cnidaria data, by questioning both the cluster duplication model [12,32] and the grouping of ParaHox genes with the Hox homology groups [18]. Assuming that Hox and ParaHox are nevertheless sister clusters which derived from a single ancestral ProtoHox cluster (reviewed in [18]), we tried to determine how the ParaHox genes can be related to the Hox genes. To do this we adopted a "Hox-centric" view, i.e. we used the "relaxed" versions of HoxPred to assign ParaHox genes in Hox PGs (Figure 5 and Additional file 2, Table S5, S6). Our attempt to perform the reverse analysis (i.e. to define in which ParaHox groups individual Hox genes would be assigned) remained inconclusive due to the very small size of the training dataset.

We found that *Gsx *and *Xlox *genes were consistently predicted as Anterior/PG3 across bilaterians. *Gsx *is similarly predicted as Anterior/PG3 in cnidarians and placozoans. The few *Xlox *genes from cnidarians predicted as Central could indicate that cnidarians *Xlox *would have emerged from a Central-like gene and are therefore distinct from the bilaterian PG3-derived *Xlox *genes. Alternatively, cnidarians *Xlox *might be related to bilaterian *Xlox*, but because of derived sequences as compared to their bilaterian counterpart, they may have been misclassified by HoxPred. Our results do not support the traditional grouping of Gsx with PG1, but are consistent with a recent phylogenetic analysis that regroups *Gsx *and *Xlox *into a PG2/PG3 group [18]. *Cdx *genes are consistently predicted in the Central group, rather than in the Posterior group.

Taken together with our data on cnidarian Hox genes, the cnidarian/bilaterian ancestor would have had a minimal Hox repertoire of four genes, composed of Anterior/PG1, Anterior/PG3, Central and Posterior, and a minimal ParaHox repertoire of two genes (*Gsx *and *Cdx*). Our results are coherent with three main ProtoHox models that seem equally parsimonious (Figure 5): (1) a four-gene ProtoHox cluster (Anterior/PG1, Anterior/PG3, Central and Posterior) [13] - the four genes would have been conserved in the Hox cluster and two genes (Anterior/PG1 and Posterior) would have been lost in the ParaHox cluster; (2) a three-gene ProtoHox cluster (Anterior/PG1, Anterior/PG3 and Central/Posterior) - previously proposed [10] but where the Central genes would have emerged specifically in bilaterians and not in cnidarians - one duplication would have produced the Posterior and Central Hox genes and one loss would explain the absence of Anterior/PG1 ParaHox genes; (3) a two-gene ProtoHox cluster (Anterior/PG3 and Central/Posterior) - this is somewhat different to a previously suggested two-gene ProtoHox model (Anterior/PG1 and Posterior) [14] - in our model, the ancestral Anterior/PG3 and Central/Posterior genes would have respectively given rise to the Hox genes of the PG3, PG1 and of the Central, Posterior groups, by two duplication events. In the three proposed models, *Gsx *and *Cdx *derive from ancestral Anterior/PG3-like and Central-like genes, respectively; *Xlox *might have evolved from a PG3-like gene by duplication prior to the cnidarians/bilaterians split, or independently in bilaterians (from PG3-like) and cnidarians (from Central-like).

## Conclusions

The extensions of HoxPred presented here fulfill the needs for automatic Hox classification across all bilaterians. This method is well-suited for the ever-growing amount of sequences to analyse, by combining predictive accuracy and time efficiency (3000 sequences screened per hour on a standard laptop). HoxPred is easily accessible to the community through a user-friendly web page [36]. The HoxPred automatic classification for thousands of homeodomain sequences are provided in the freely available Datab'Hox database [36]. This new resource thereby offers supplementary information to the existing database HomeoDB [57], a manually curated database of homeobox genes with annotations based on published articles - when possible, links to HomeoDB are provided from Datab'Hox.

Our analyses illustrate the capacity of HoxPred to provide valuable predictions in ongoing genome projects. It is particularly appropriate for dispersed Hox clusters, as it directly pinpoints the potential Hox sequences. Beyond its classification purposes, we showed that HoxPred can also serve to study the evolution of Hox genes in metazoans. In this respect, we propose here evolutionary scenarios for several crucial questions. Bilaterian posterior Hox genes would have arisen from an ancestral PG9, with new homology groups arising in chordates, and in ambulacraria (group 11/13). Our model favours independent duplications, or a mixture of the two processes as suggested in [44,47], over the "Deuterostome Posterior Flexibility" hypothesis alone. It would be beneficial for the community to revise the nomenclature of the posterior Hox genes in non-vertebrate deuterostomes, so that the number of a gene with respect to its position within the cluster would not be confused with its homology group. Our evolutionary scenario for bilaterian Central genes suggests that *Urbilateria *would have possessed Central genes from PG4, PG5, PG6 and PG7; PG8 appearing later in vertebrates. Besides, our results bring additional support to the grouping of the Central protostome genes into Ubd-A-type and ftz/Antp/Lox5. We also provide further insights into the notoriously controversial relationships between cnidaria and bilateria Hox genes. Our analysis suggests that the cnidarian/bilaterian ancestor would have had a minimal Hox repertoire of four genes, from the Anterior/PG1, Anterior/PG3, Central and Posterior groups. HoxPred thus yields stimulating results in the context of the current views, by indicating the presence of cnidarian Central gene. Regarding the ParaHox genes, we suggest that *Gsx *derived from an ancestral PG3-like gene, while *Cdx *would be closer to the Central (Central/Posterior) genes, which is coherent with our data on cnidarian Hox genes. *Xlox *would have independently emerged from a PG3-like gene in bilaterians and from a Central-like gene in cnidarians, or alternatively emerged earlier, from a PG3-like gene. Taken together, our results are consistent with three possible models for the early evolution of Hox and ParaHox genes: a two-gene (Anterior/PG3, Central/Posterior), a three-gene (Anterior/PG1, Anterior/PG3 and Central/Posterior), or a four-gene (Anterior/PG1, Anterior/PG3, Central, Posterior) ProtoHox model.

## Methods

### Sequence Datasets

Sequences were mostly retrieved from UniprotKB [58] release 14.5. *Strongylocentrotus purpuratus *sequences were deduced from the annotation of the Hox cluster sequence (Genbank accession AC165428) and also retrieved from the sea urchin genome project http://www.hgsc.bcm.tmc.edu/projects/seaurchin/[59]. The draft assemblies of the *Capitella sp. I, Helobdella robusta, Lottia gigantea, Daphnia pulex *and *Branchiostoma floridae *genomes are accessible at the DOE Joint Genome Institute http://www.jgi.doe.gov/. To analyse these genomes with HoxPred, the complete sets of homeodomain proteins were first filtered as in [35]. Here, only proteins matching the InterPro IPR001356 or IPR001827 homeobox domain were retrieved and further analysed.

### The four versions of HoxPred

The initial HoxPred program [35] was constructed from vertebrate sequences only, and constitutes the "Vertebrate" version (not used in this study). The same procedure as in [35] was followed to produce the three new versions (including leave-one-out cross-validation, permutation tests and a variable selection step). The prior probabilities values were similar. For the "Bilateria" and "Bilateria_relaxed" versions, 6 new profiles corresponding to the Anterior, Central, Posterior, Gsx, Xlox and Cdx homology groups were developed. These profiles were constructed from alignments of 440 Hox and 37 ParaHox non-redundant homeodomain sequences (Additional file 4). These sequences were collected from publicly available databases and then manually curated. Sequences that were well-annotated and unambiguously classified into the Anterior, Central, Posterior, Gsx, Xlox and Cdx homology groups in a phylogenetic tree (not shown) were included in this training dataset. Contrary to the Vertebrate version, these sequences were extracted from various bilaterian phyla (including vertebrates). The "Vertebrate"/"Vertebrate_relaxed" and "Bilateria"/"Bilateria_relaxed" versions were respectively built upon the same collections of profiles. The main difference is related to the datasets used for the training of the discriminant analysis. In the "relaxed" versions, the control (CTL) group contains randomly-generated (RANDOM) sequences only. In the "non-relaxed" versions, the CTL group consists of both RANDOM and non-Hox homeodomain sequences (HOMEO) (1074 bilaterian sequences for the "Bilateria" version). The "non-relaxed" versions thus return all non-Hox sequences in the CTL group, while the "relaxed" versions can classify such sequences into Hox PG, thereby allowing the study of ParaHox sequences. All versions of HoxPred return classifications with posterior probabilities; the group with the highest posterior probability is considered as the prediction.

### Evaluation of HoxPred predictions

The statistical evaluation of HoxPred predictions was performed on 800 public sequences (89 Hox and 711 non-Hox), with the programs compare-classes (option matrix file) and contigency-stats from the Regulatory Sequence Analysis Tools (RSAT) [60]/Network Analysis Tools (NeAT) [61], available at http://rsat.ulb.ac.be/rsat/. The statistic used to evaluate the performance of HoxPred (Table 1), is the geometric accuracy, as previously described in [35]. For "relaxed" versions that are not intended for direct classification purposes, the accuracy is calculated for Hox genes only (excluding predictions of non-Hox homeobox genes), which under-estimates the global accuracy.

## Authors' contributions

MT-C extended HoxPred, performed the sequence analyses and designed the Datab'Hox database. MT-C and MV constructed the evolutionary scenarios, and MV performed the phylogenetic analyses. MV, VL and LL participated in the design and coordination of the study. MT-C drafted the manuscript and all the authors participated in the editing of the manuscript. All the authors read and approved the final manuscript.

## Supplementary Material

Additional file 1 **Supplementary figures**. This file contains the supplementary figures S1, S2 and S3. Click here for file

Additional file 2 **Supplementary tables**. Tables with accession numbers, protein names and HoxPred predictions. Click here for file

Additional file 3 **Multiple alignments of Hox and ParaHox homeodomains**. Alignments of homedomain sequences of Hox and ParaHox sequences from the Datab'Hox database, ordered by homology groups. Click here for file

Additional file 4 **Accession numbers of training sequences**. List of accession number and name of sequences used in the training dataset. Corresponding multiple alignments are available upon request. Click here for file
