# Supplementary material for: A non-tree-based comprehensive study of metazoan Hox and ParaHox genes prompts new insights into their origin and evolution
Source: BMC Evol Biol. 2010 Mar 11;10:73. doi: 10.1186/1471-2148-10-73 (PMC2842273; doi:10.1186/1471-2148-10-73)
Supplement: Additional file 1 — Supplementary figures. This file contains the supplementary figures S1, S2 and S3. [file 1471-2148-10-73-S1.PDF]

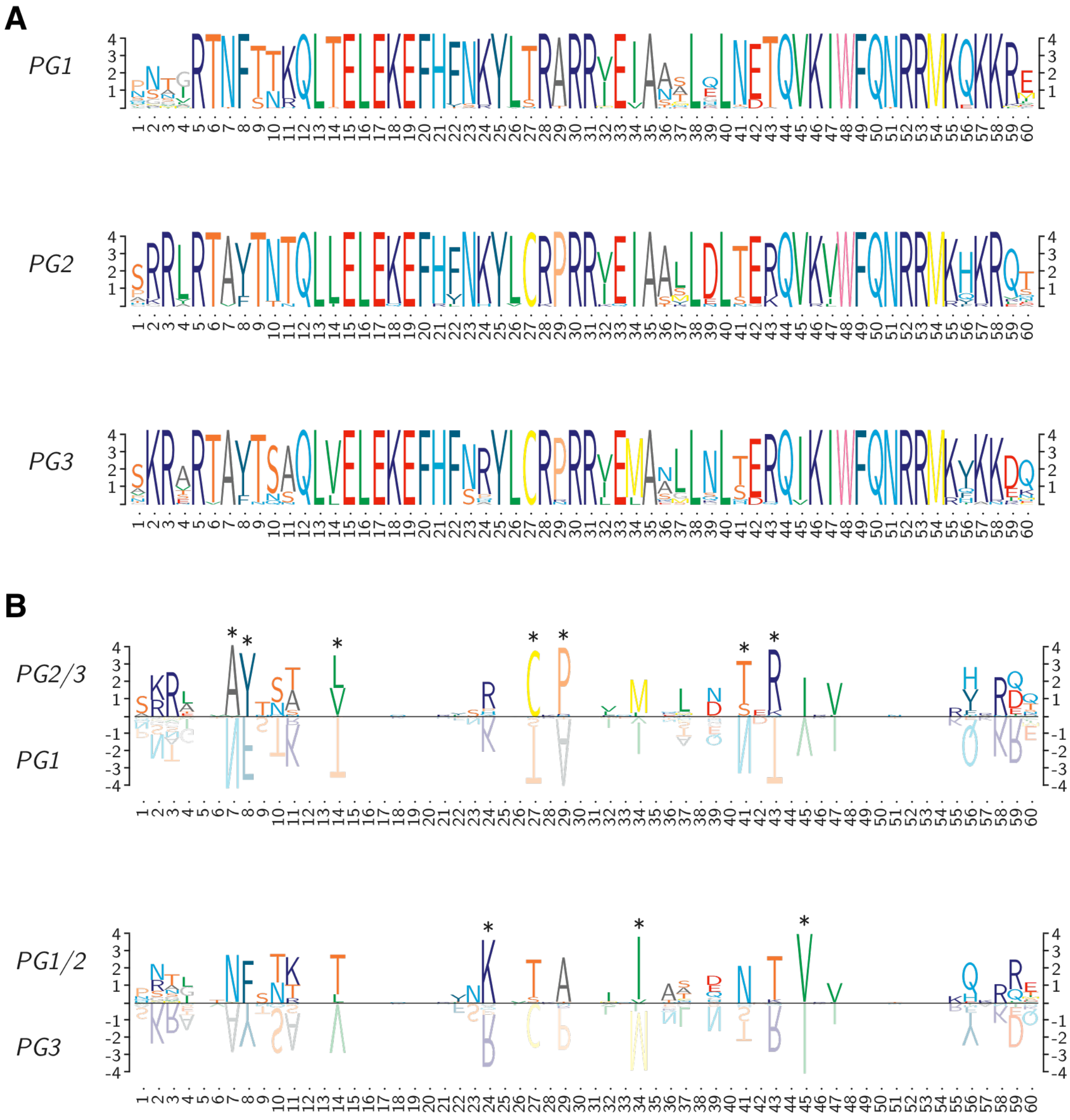

Figure S1. Residue conservation of bilaterian homeodomain sequences from the Anterior homology group. A. The homeodomain motif is represented as sequence Logos. They were obtained with TeXshade [1], from the alignments of unique bilaterian sequences of various phyla, belonging to PG1 (56 sequences), PG2 (24 sequences) and PG3 (27 sequences). **B.** Differential logos showing sequence deviation between the PG1,PG2 and PG3 groups, obtained with Subfamily [2]. The upper part of the logos shows residues that are characteristic for the indicated subfamily of sequences, compared to the remaining sequences whose typical residues are indicated in the lower part, in the form of a shaded and reversed logo. Residues conserved in all sequences are not shown. The stars indicate positions that have been identified by Subfamily as significantly deviating from the other subfamily. The groups PG2/3 share 7 characteristic residues when compared to PG1 (top logo), whereas PG1/2 only have 3 residues in common when compared to PG3 (bottom logo). The positions of the 7 characteristic residues between PG2 and PG3 are 7-8 (PG1 = NF, PG2/3: AY), 14 (PG1=T, PG2/3= L or V), positions 27-29 (PG1 = TRA, PG2/3 = CRP), 41 (PG1=N, PG2/3= T or S) and 43 (PG1= T, PG2/3=R or K).

[1] Beitz. TEXshade: shading and labeling of multiple sequence alignments using LATEX2 epsilon. Bioinformatics (2000) vol. 16 (2) pp. 135-9

[2] Beitz. Subfamily logos: visualization of sequence deviations at alignment positions with high information content. BMC Bioinformatics (2006) vol. 7 pp. 313

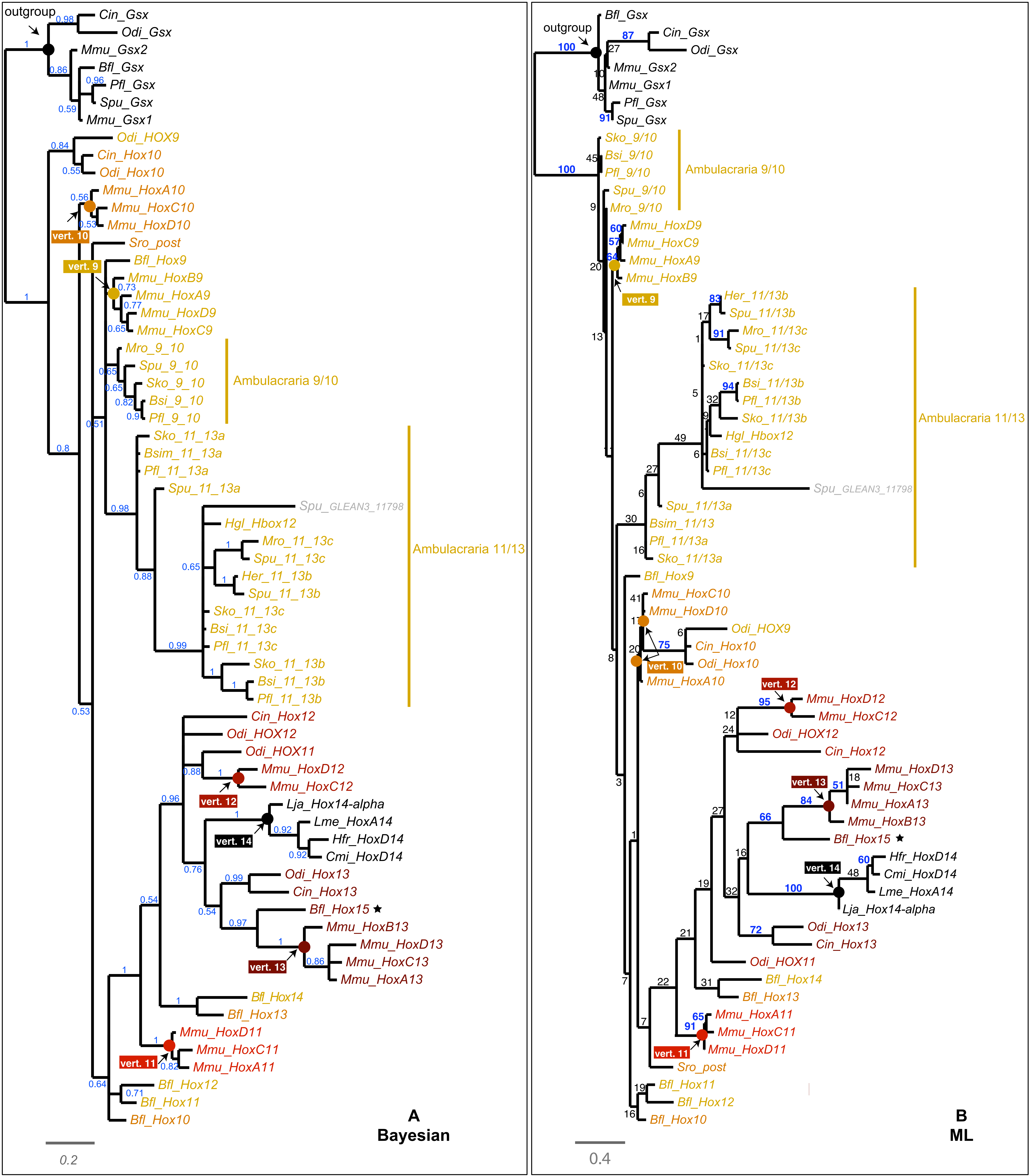

outgroup
Cin_Gsx
Odi_Gsx
Mmu_Gsx2
Bfl_Gsx
Pfl_Gsx
Spu_Gsx
Mmu_Gsx1
Odi_HOX9
Cin_Hox10
Odi_Hox10
Mmu_HoxA10
Mmu_HoxC10
Mmu_HoxD10
vert. 10
Sro_post
vert. 9
Bfl_Hox9
Mmu_HoxB9
Mmu_HoxA9
Mmu_HoxD9
Mmu_HoxC9
Mro_9_10
Spu_9_10
Sko_9_10
Bsi_9_10
Pfl_9_10
Ambulacraria 9/10
Sko_11_13a
Bsim_11_13a
Pfl_11_13a
Spu_11_13a
Spu_GLEAN3_11798
Hgl_Hbox12
Mro_11_13c
Spu_11_13c
Her_11_13b
Spu_11_13b
Sko_11_13c
Bsi_11_13c
Pfl_11_13c
Sko_11_13b
Bsi_11_13b
Pfl_11_13b
Ambulacraria 11/13
Cin_Hox12
Odi_HOX12
Odi_HOX11
Mmu_HoxD12
Mmu_HoxC12
vert. 12
Lja_Hox14-alpha
Lme_HoxA14
Hfr_HoxD14
Cmi_HoxD14
vert. 14
Odi_Hox13
Cin_Hox13
Bfl_Hox15 ★
Mmu_HoxB13
Mmu_HoxD13
Mmu_HoxC13
Mmu_HoxA13
vert. 13
Bfl_Hox14
Bfl_Hox13
Mmu_HoxD11
Mmu_HoxC11
Mmu_HoxA11
vert. 11
Bfl_Hox12
Bfl_Hox11
Bfl_Hox10
0.2
A
Bayesian
Bfl_Gsx
outgroup
Cin_Gsx
Odi_Gsx
Mmu_Gsx2
Mmu_Gsx1
Pfl_Gsx
Spu_Gsx
Sko_9/10
Bsi_9/10
Pfl_9/10
Spu_9/10
Mro_9/10
Ambulacraria 9/10
Mmu_HoxD9
Mmu_HoxC9
Mmu_HoxA9
Mmu_HoxB9
vert. 9
Her_11/13b
Spu_11/13b
Mro_11/13c
Spu_11/13c
Sko_11/13c
Bsi_11/13b
Pfl_11/13b
Sko_11/13b
Hgl_Hbox12
Bsi_11/13c
Pfl_11/13c
Spu_GLEAN3_11798
Spu_11/13a
Bsim_11/13
Pfl_11/13a
Sko_11/13a
Ambulacraria 11/13
Bfl_Hox9
Mmu_HoxC10
Mmu_HoxD10
Odi_HOX9
Cin_Hox10
Odi_Hox10
vert. 10
Mmu_HoxA10
vert. 12
Mmu_HoxD12
Mmu_HoxC12
Odi_HOX12
Cin_Hox12
Mmu_HoxD13
vert. 13
Mmu_HoxC13
Mmu_HoxA13
Mmu_HoxB13
Bfl_Hox15 ★
Hfr_HoxD14
vert. 14
Cmi_HoxD14
Lme_HoxA14
Lja_Hox14-alpha
Odi_Hox13
Cin_Hox13
Odi_HOX11
Bfl_Hox14
Bfl_Hox13
Mmu_HoxA11
Mmu_HoxC11
Mmu_HoxD11
vert. 11
Sro_post
Bfl_Hox11
Bfl_Hox12
Bfl_Hox10
0.4
B
ML

Figure S2. Phylogenetic analyses of the deuterostome posterior Hox genes. Both trees were constructed from a multiple alignment of homeodomain sequences (60 residues). The sequence names are colored according to the classification by HoxPred. The vertebrate PGs clusters and the ambulacraria groups 9/10 and 11/13 are indicated. The amphioxus Hox15 sequence is indicated by a star. A. The tree is a Bayesian inference tree, which has been rooted using the Gsx gene of various deuterostomes as an outgroup. The statistical support values are posterior probabilities, indicated in blue. B. The tree is a maximum-likelihood tree, which has been rooted using the Gsx gene of various deuterostomes as an outgroup. The statistical support values are bootstrap support (100 bootstrap replicates). Support values >50 are indicated in blue.

Methods:

Maximum likelihood (ML) analyses were performed with PHYML [1]. PHYML analyses were performed using the WAG amino-acid substitution model [2], the frequencies of amino acids being estimated from the data set, and rate heterogeneity across sites being modelled by two rate categories (one constant and eight γ-rates). Statistical support for the different internal branches was assessed by bootstrap resampling (150 bootstrap replicates), as implemented in PHYML [1]. Bayesian inference was performed using the Markov chain Monte Carlo method as implemented in the MRBAYES (version 3) package [3,4]. We used the WAG substitution frequency matrix [2] with among-sites rate variation modelled by means of a discrete distribution with four equally probable categories. Two independent Markov chains were run, each containing 3,000,000 Monte Carlo steps. One out of every 200 trees was saved. The trees obtained in the two runs were meshed and the first 25% of the trees were discarded as 'burnin'. Posterior probabilities at each internal branch were taken as a measure of statistical support.

References:

1. Guindon S, Gascuel O: **A simple, fast, and accurate algorithm to estimate large phylogenies by maximum likelihood**. *Systematic Biology* 2003, **52**(5):696-704.

2. Whelan S, Goldman N: **A general empirical model of protein evolution derived from multiple protein families using a maximum-likelihood approach**. *Molecular Biology and Evolution* 2001, **18**(5):691-699.

3. Ronquist F, Huelsenbeck JP: **MrBayes 3: Bayesian phylogenetic inference under mixed models**. *Bioinformatics* 2003, **19**(12):1572-1574.

4. Huelsenbeck JP, Ronquist F: **MRBAYES: Bayesian inference of phylogenetic trees**. *Bioinformatics* 2001, **17**(8):754-755.

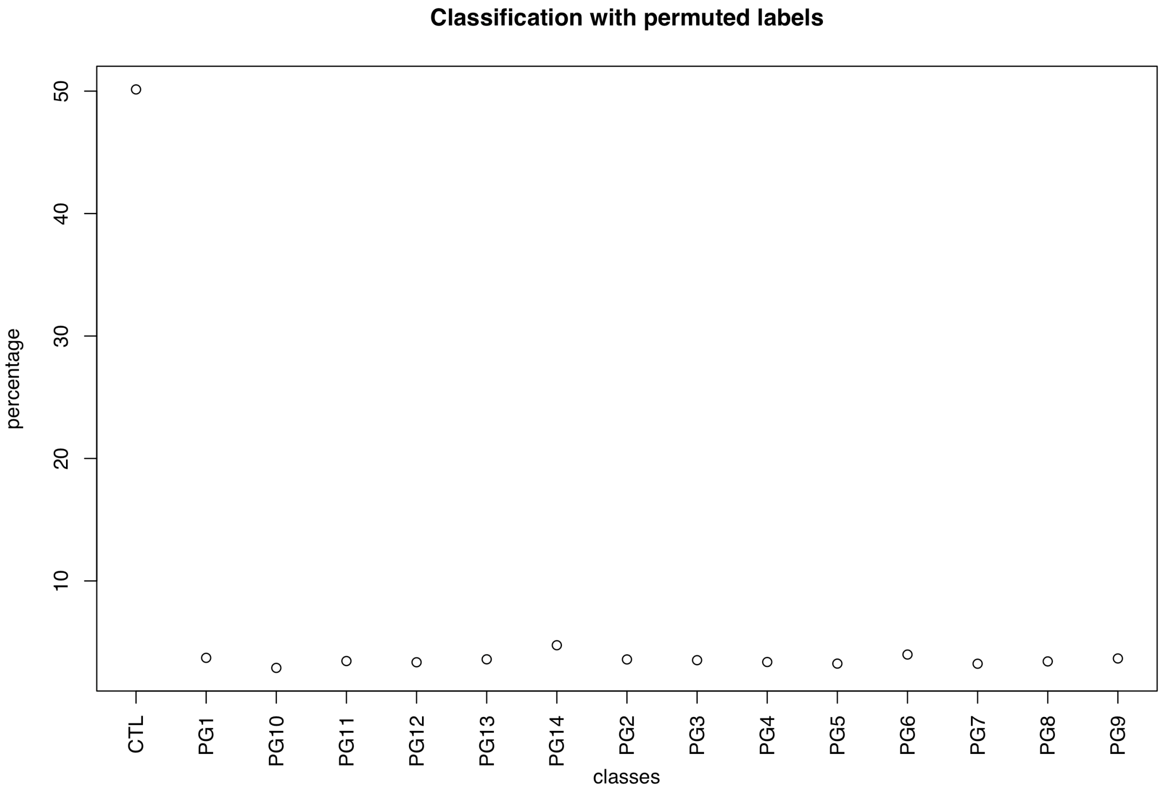

Figure S3. Permutation tests for Hox classes. The labels of the training dataset were permuted to evaluate whether specific classes are favored for the predictions. As permutation tests always return 100% classification into the CTL group (due to its high prior probability), only the Hox class labels were permutated here (100 independent permutation tests performed). Half of the training set are CTL sequences, here correctly classified into CTL class. The Hox sequences are classified in all PGs with a similar percentage, showing a random classification rather than a bias towards one particular class.
