# Supplementary material for: A non-tree-based comprehensive study of metazoan Hox and ParaHox genes prompts new insights into their origin and evolution
Source: BMC Evol Biol. 2010 Mar 11;10:73. doi: 10.1186/1471-2148-10-73 (PMC2842273; doi:10.1186/1471-2148-10-73)
Supplement: Additional file 2 — Supplementary tables. Tables with accession numbers, protein names and HoxPred predictions. [file 1471-2148-10-73-S2.PDF]

Species	Accession number	Protein name	Reference assignation		HoxPred assignation	
			vert. PG	broad groups	Vert_relaxed	Bilateria/ Bilateria_relaxed
**Cephalochordates**						
	Q9UAL6_BRAFL	AmphiHox-1	PG1	Anterior	PG1 (1)	Anterior (1)
	Q9UAL7_BRAFL	AmphiHox-2	PG2	Anterior	PG2 (1)	Anterior (1)
	HOX3_BRAFL	AmphiHox-3	PG3	Anterior	PG3 (1)	Anterior (1)
	Q9UAL8_BRAFL	AmphiHox-4	PG4	Central	PG4 (1)	Central (1)
	Q17140_BRAFL	AmphiHox-5	PG5	Central	PG5 (1)	Central (1)
	Q17141_BRAFL	AmphiHox-6	**PG6**	Central	**PG7** (1)	Central (1)
Branchiostoma floridae	Q17142_BRAFL	AmphiHox-7	PG7	Central	PG7 (1)	Central (1)
	Q17143_BRAFL	AmphiHox-8	**PG8**	Central	**PG7** (0.890)	Central (1)
	Q17144_BRAFL	AmphiHox-9*	PG9	Posterior	PG9 (1)	Posterior (1)
	Q17136_BRAFL	AmphiHox-10*	PG10	Posterior	PG10 (1)	Posterior (1)
	Q9NAZ2_BRAFL	AmphiHox-11*	**PG11**	Posterior	**PG9** (1)	Posterior (1)
	Q9NAZ1_BRAFL	AmphiHox-12*	**PG12**	Posterior	**PG9** (0.998)	Posterior (1)
	Q9NAZ0_BRAFL	AmphiHox-13*	**PG13**	Posterior	**PG10 (1)**	Posterior (1)
	Q9N2K2_BRAFL	AmphiHox-14*	**PG14**	Posterior	**PG9** (0.999)	Posterior (1)
**Urochordates**						
	Q4H3D2_CIOIN	CiHox1	PG1	Anterior	PG1 (1)	Anterior (1)
	Q8I7D1_CIOIN	CiHox2	PG2	Anterior	PG2 (1)	Anterior (1)
	Q4H3C7_CIOIN	CiHox3	PG3	Anterior	PG3 (1)	Anterior (1)
	Q4H3C6_CIOIN	CiHox4	PG4	Central	PG4 (0.819)	Central (1)
Ciona Intestinalis	O18313_CIOIN	CiHox5	PG5	Central	PG5 (1)	Central (1)
	Q8I7C9_CIOIN	CiHox6/7	PG6-8	Central	PG6 (0.938)	Central (1)
	Q4H3D1_CIOIN	CiHox10	PG10	Posterior	PG10 (1)	Posterior (1)
	Q4H3D0_CIOIN	CiHox12	PG12	Posterior	PG12 (1)	Posterior (1)
	Q4H3C9_CIOIN	CiHox13	PG13	Posterior	PG13 (1)	Posterior (1)
	Q675P0_OIKDI	Hox1	PG1	Anterior	PG1 (1)	Anterior (1)
	Q4VWU1_OIKDI	Hox2	PG2	Anterior	PG2 (1)	Anterior (1)
	Q4VWU0_OIKDI	Hox4	PG4	Central	PG4 (0.999)	Central (1)
	Q66S86_OIKDI	Hox4.1*	**PG4**	Central	**PG5** (0.907)	Central (1)
Oikopleura dioica	Q675T8_OIKDI	Hox9*	PG9	Posterior	PG9 (1)	Posterior (0.999)
	Q66S38_OIKDI	Hox10	PG10	Posterior	PG10 (1)	Posterior (1)
	Q675V3_OIKDI	Hox11	**PG11**	Posterior	**PG12** (1)	Posterior (1)
	Q675Y2_OIKDI	Hox12	PG12	Posterior	PG12 (1)	Posterior (1)
	Q4VWT5_OIKDI	Hox13	PG13	Posterior	PG13 (1)	Posterior (1)
**Echinoderms**						
	AC165428	Hox1	PG1	Anterior	PG1 (1)	Anterior (1)
	"	Hox2	PG2	Anterior	PG2 (1)	Anterior (1)
	"	Hox3	PG3	Anterior	PG3 (1)	Anterior (0.999)
	"	Hox5	PG5	Central	PG5 (1)	Central (1)
	"	Hox6	PG6-8	Central	PG7 (0.999)	Central (1)
Strongylocentrotus purpuratus	"	Hox7	PG6-8	Central	PG7 (0.998)	Central (1)
	"	Hox8	PG6-8	Central	PG7 (1)	Central (1)
	"	Hox9/10	PG9-10	Posterior	**PG9** (1)	Posterior (1)
	"	Hox11/13a	PG9-13	Posterior	**PG9** (1)	Posterior (1)
	"	Hox11/13b	PG9-13	Posterior	**PG9** (1)	Posterior (1)
	GLEAN3_00388	Hox11/13c	PG9-13	Posterior	**PG9** (1)	Posterior (1)
**Hemichordates**						
	Q7YTB8_SACKO	Hox1	PG1	Anterior	PG1 (1)	Anterior (1)
	A0FDP3_SACKO	Hox2	PG2	Anterior	PG2 (1)	Anterior (1)
	Q7YTC7_SACKO	Hox3	PG3	Anterior	PG3 (1)	**CTL (0.999)/Anterior (1)**
	Q7YTB7_SACKO	Hox4	**PG4**	Central	**PG5** (0.999)	Central (1)
	A0FDP4_SACKO	Hox5	PG5	Central	PG5 (1)	Central (1)
Saccoglossus kowalevskii	A0FDP5_SACKO	Hox6	PG6-8	Central	PG7 (0.999)	Central (1)
	Q7YTC6_SACKO	Hox7	PG6-8	Central	PG7 (0.626)	Central (1)
	A0FDP6_SACKO	Hox9/10	PG9-10	Posterior	**PG9** (1)	Posterior (1)
	A0FDP7_SACKO	Hox11/13a	PG9-13	Posterior	**PG9** (1)	Posterior (1)
	A0FDP8_SACKO	Hox11/13b	PG9-13	Posterior	**PG9** (0.999)	Posterior(1)
	Q7YTC5_SACKO	Hox11/13c	PG9-13	Posterior	**PG9** (1)	Posterior (1)

Table S1: Classification of non-vertebrate deuterostome Hox proteins with HoxPred. The posterior probability associated with each HoxPred prediction is shown in parentheses. Differences between the reference annotations and the predictions are highlighted in bold. *Branchiostoma floridae*: The reference annotation follows the Deuterostome Posterior Flexibility hypothesis. *Oikopleura dioica*: Hox4.1 was not taken into account in the original analysis (Seo et al, Nature,2004). Hox9 could either be Hox9a or Hox9b, as only one sequence is available in public databases. Reference assignations are according to Ferrier et al, Evol Dev, 2000 ; Ikuta et al, PNAS, 2004 ; Seo et al, Nature, 2004 ; Cameron et al, J Exp Zool B Mol Dev Evol, 2006 and Aronowicz et al, Integrative and Comparative Biology, 2006.

Species	Accession number	Protein name	Reference assignation		HoxPred assignation	
			vert. PG	broad groups	Vert_relaxed	Bilateria/ Bilateria_relaxed
**Arthropods**						
	LAB_DROME	labial (CG1264)	PG1	Anterior	PG1 (1)	Anterior (1)
	HMPB_DROME	proboscipedia (CG31481)	PG2	Anterior	PG2 (1)	Anterior (1)
	ZEN1_DROME	*Zerknullt 1(CG1046)*	*PG3*	*Anterior*	*PG3 (0.999)*	*CTL (1)/Anterior (1)*
	ZEN2_DROME	*Zerknullt 2 (CG1048)*	*PG3*	*Anterior*	*PG3 (0.999)*	*CTL (1)/Anterior (1)*
	BCD_DROME	*Bicoid (CG1034)*	*PG3*	*PG3*	*CTL (1)*	*CTL (1)/Anterior (1)*
Drosophila melanogaster	DFD_DROME	Deformed (CG2189)	PG4	Central	PG4 (1)	Central (1)
	SCR_DROME	Sex combs reduced (CG1030)	PG5	Central	PG5 (1)	Central (1)
	FTZ_DROME	*fushi tarazu (CG2047)*	*PG6-8*	*Central*	*PG7 0.999*	*Central (1)*
	ANTP_DROME	Antennapedia (CG1028)	PG6-8	Central	PG7 (1)	Central (1)
	UBX_DROME	Ultrabithorax (CG10388)	PG6-8	Central	PG6 (0.984)	Central (1)
	ABDA_DROME	abdominal-A (CG10325)	PG6-8	Central	PG6 (0.999)	Central (1)
	ABDB_DROME	Abdominal-B (CG10291)	PG9-13	Posterior	PG9 (1)	Posterior (1)
**Nematodes**						
	HM13_CAEEL	ceh-13	PG1	Anterior	PG1 (1)	Anterior (1)
	LIN39_CAEEL	lin-39	PG4	Central	PG4 (0.999)	Central (0.999)
Caenorhabditis elegans	MAB 5_CAEEL	mab-5	PG6-8	Central	PG6 (1)	Central (1)
	EGL5_CAEEL	egl-5	PG9-13	**Posterior**	**CTL (1)**	**Posterior(0.999)/Central(0.998)**
	Q9XW88_CAEEL	php-3	PG9-13	Posterior	PG9 (1)	Posterior (1)
	Q9XW76_CAEEL	nob-1	PG9-13	**Posterior**	**CTL (1)**	Posterior (1)
**Annelids**						
	Q9U9U2_NERVI	labial	PG1	Anterior	PG1 (1)	Anterior (1)
	Q9U9U1_NERVI	proboscipedia	PG2	Anterior	PG2 (1)	Anterior (1)
	Q9U9U0_NERVI	Hox3	PG3	Anterior	PG3 (1)	Anterior (1)
Nereis virens	Q9U9T9_NERVI	Deformed	**PG4**	Central	**PG5 (1)**	Central (1)
	Q9U9T8_NERVI	Sex combs reduced	PG5	Central	PG5 (1)	Central (1)
	Q9U9T7_NERVI	Lox2	PG6-8	Central	PG6 (1)	Central (1)
	Q9U9T4_NERVI	Lox5	PG6-8	Central	PG7 (1)	Central (1)
	Q0QII7_NERVI	Hox7	PG6-8	Central	PG7 (1)	Central (1)
	Q9U9T6_NERVI	Lox4	PG6-8	Central	PG7 (0.955)	Central (1)
	Q9U9T3_NERVI	Post1	PG9-13	Posterior	PG12 (1)	Posterior (1)
	Q9U9T2_NERVI	Post2	PG9-13	Posterior	PG12 (0.999)	Posterior (1)
**Nemertine**						
	O62548_LINSA	Hox1	PG1	Anterior	PG1 (1)	Anterior (1)
	O62549_LINSA	Hox3	PG3	Anterior	PG3 (1)	Anterior (1)
Lineus sanguineus	HXA4_LINSA 8	Hox4	PG4	Central	PG4 (1)	Central (1)
	O62550_LINSA	Hox6	PG6-8	Central	PG7 (1)	Central (1)
	O62551_LINSA	Hox7	PG6-8	Central	PG7 (1)	Central (1)
	O62552_LINSA	Hox9	PG9-13	Posterior	PG9 (1)	Posterior (1)

Table S2: Classification of protostome Hox proteins with HoxPred. The posterior probability associated with each HoxPred prediction is shown in parentheses. Differences between the reference annotations and the predictions are highlighted in bold. The *Drosophila melanogaster* proteins written in italics are Hox-derived. Reference assignations are according to Balavoine et al., Mol Phylogenet Evol, 2002. For the *C. elegans egl-5* gene, the Bilateria prediction as Posterior is more reliable, but the discordant Bilateria_relaxed prediction as Central is informative, and could be coherent with an evolution via fusion with a Central gene, as observed in the nematode *Brugia Malayi* (Aboobaker and Blaxter. Hox gene evolution in nematodes: novelty conserved. Curr Opin Genet Dev (2003) vol. 13 (6) pp. 593-8).

Species	Accession number	Protein name	HoxPred assignation		
			Vert_relaxed	Bilateria	Bilateria_relaxed
**DEUTEROSTOMES**					
Branchiostoma floridae	O61586_BRAFL	AmphiGsx	PG2 (1)	Gsx (1)	Anterior (1)
	Q9TZY9_BRAFL	AmphiXlox	PG3 (1)	Xlox (1)	Anterior (1)
	Q9TZY8_BRAFL	AmphiCdx	CTL (1)	Cdx (1)	Central (0.999)
	Q95W41_BRAFL	AmphiEvx	PG3 (0.997)	CTL (1)	Anterior (1)
	Q1WAB2_BRAFL	Gastrulation brain homeobox	PG4 (1)	CTL (1)	Anterior (1)
	Q9GSF1_BRAFL	Mnx	PG4 (1)	CTL (1)	Anterior (1)
	Q8T600_BRAFL	Amphimox	PG4 (1)	CTL (1)	Posterior (0.999)
Ciona intestinalis	Q9BMT5_CIOIN	Gsx	PG3 (1)	Gsx (1)	Anterior (1)
	Q9GP86_CIOIN	Ci-Xlox/IPF1	PG3 (1)	Xlox (1)	Anterior (0.984)
	Q4H3T1_CIOIN	Ci-Cdx	PG6 (1)	Cdx (1)	Central (1)
	Q4H364_CIOIN	Ci-Mnx	PG3 (0.999)	CTL (1)	Anterior (1)
	Q4H338_CIOIN	Ci-Nkx-C	PG4 (0.999)	CTL (1)	Anterior (0.999)
Oikopleura dioica	Q5EVF3_OIKDI	Gsx	PG4 (1)	Gsx (1)	Anterior (1)
	Q5EVF1_OIKDI	Cdx1	CTL (1)	Cdx (1)	Posterior (1)
	Q5EVF0_OIKDI	Cdx2	CTL (1)	Cdx (1)	Central (1)
	Q5EVE9_OIKDI	Cdx3	CTL (1)	Cdx (1)	Posterior (0.999)
	Q5EVM9_OIKDI	Mnx	PG4 (0.999)	CTL (1)	Anterior (1)
	Q5EVN1_OIKDI	CG13424-related protein 1	PG4 (1)	CTL (1)	Central (1)
Strongylocentrotus purpuratus	GLEAN3_13436	Sp-Gsx	PG3 (1)	Gsx (1)	Anterior (1)
	Q8IT48_STRPU	Splox	PG3 (1)	Xlox (1)	Anterior (1)
	GLEAN3_24715	Sp-Cdx	PG6 (0.999)	Cdx (1)	Central (1)
	GLEAN3_25486	Sp-Mox	PG4 (1)	CTL (1)	Posterior (1)
	GLEAN3_02816	Sp-Hb9	PG4 (1)	CTL (1)	Anterior (1)
	Q6JJ71_STRPU	Nk1	PG4 (0.977)	CTL (1)	Anterior (1)
Saccoglossus kowalevskii	B5B3S6_SACKO	Cdx	PG7 (0.999)	Cdx (1)	Central (1)
	Q1PHP7_SACKO	Mox	PG4 (1)	CTL (1)	Posterior (1)
	Q1PHQ5_SACKO	Motor neuron homeobox	PG4 (1)	CTL (1)	Anterior (1)
	Q7YTC8_SACKO	Gastrulation brain homeobox	PG4 (1)	CTL (1)	Anterior (1)
**PROTOSTOMES**					
Drosophila melanogaster	Q7KUL4_DROME	ind (CG11551) (=Dm-gsx)	PG3 (1)	Gsx (1)	Anterior (1)
	CAD_DROME	Homeotic protein caudal (cad)	CTL (1)	Cdx (1)	Central (0.999)
	EVE_DROME	even skipped (CG2328)	PG3 (0.999)	CTL (1)	Anterior (1)
	UNPG_DROME	unplugged (CG1650) (=Dm-gbx)	PG4 (1)	CTL (1)	Anterior (1)
	Q9VSC2_DROME	extra-extra (CG8254)(=Dm-HB9)	PG4 (1)	CTL (1)	Anterior (1)
	SLOU_DROME	slou/NK-1 (CG6534)	PG4 (0.954)	CTL (1)	Anterior (0.999)
Caenorhabditis elegans	Q65ZB9_CAEEL	Cad / Pal-1	CTL (1)	Cdx (1)	Central (0.999)
Nereis virens	Q0ZRS5_NERVI	Gsx	PG3 (1)	Gsx (1)	Anterior (1)
	Q0QIJ2_NERVI	Xlox	PG3 (1)	Xlox (0.999)	Anterior (1)
	Q7Z0F5_NERVI	Cdx	PG5 (0.907)	Cdx (1)	Central (1)
Lineus sanguineus	CDX_LINSA	Cdx	PG6 (0.999)	Cdx (1)	Central (1)

Table S3: Classification of non-Hox proteins with HoxPred in nine non-vertebrate species. The posterior probability associated with each HoxPred prediction is shown in parentheses. The complete dataset of non-Hox homeodomain sequences comprises 711 sequences. Sequences classied by all versions of HoxPred in the control (CTL) group or only classified with the Bilateria_relaxed version are not shown.

Species	Genomic localisation	JGI identifier	Protein name	HoxPred assignation	
				Vert_relaxed	Bilateria
**Arthropods**					
	(-) scaffold_7:211891-218605	YAS_Hox_Lab	Labial	PG1 (1)	Anterior (1)
	(-) scaffold_7:236655-244364	YAS_NCBI_GNO_0700045	Hox2	PG2 (1)	Anterior (1)
	(-) scaffold_7:262501-266259	YAS_fgenesh1_pg.C_scaffold_7000048	Hox3	PG3 (1)	Anterior (1)
	(-) scaffold_7:297160-298774	YAS_Hox_Dfd1	Dfd	PG4 (1)	Central (1)
	(-) scaffold_7:320058-326935	YAS_YAS_Hox_Scr	Scr	PG5 (1)	Central (1)
	(-) scaffold_7:337520-339647	YAS_Hox_Ftz	Ftz	PG7 (0.994)	Central (1)
Daphnia pulex	(-) scaffold_7:367247-370699	YAS_fgenesh1_pm.C_scaffold_7000012	Antp	PG7 (1)	Central (1)
	(-) scaffold_7:387197-402061	YAS_NCBI_GNO_0700054	Ubx	PG6 (0.983)	Central (1)
	(-) scaffold_7:468873-488617	YAS_Hox_Abd-A	Abd-A	PG6 (0.999)	Central (1)
	(-) scaffold_7:547973-550822	YAS_Hox_Abd-B	Abd-B	PG9 (1)	Posterior (1)
	(-) scaffold_7:1219725-1227076	YAS_pHox_Cad	Cdx	PG6 (0.999)	Cdx (1)
	(+) scaffold_25:781975-782169	gw1.25.116.1	Extra extra	PG4 (1)	CTL (1)
**Molluscs**					
	(-) sca_12:2450141-2450374	gw1.12.670.1	Labial	PG1 (1)	Anterior (1)
	(-) sca_12:2478487-2479080	e_gw1.12.69.1	Proboscipedia	PG2 (1)	Anterior (1)
	(-) sca_12:2515357-2523362	gw1.12.35.1	Hox3	PG3 (1)	Anterior (1)
	(-) sca_12:2563935-2570098	e_gw1.12.439.1	Hox4	PG4 (1)	Central (1)
	(-) sca_12:2595934-2607819	gw1.12.429.1	Hox5	PG5 (1)	Central (1)
	(-) sca_12:2641587-2651146	gw1.12.445.1	Antennapedia-like	PG7 (1)	Central (1)
	(-) sca_12:2675803-2676486	fgenesh2_pm.C_sca_12000048	Antennapedia-like	PG7 (1)	Central (1)
	(-) sca_12:2723164-2733374	estExt_Genewise1.C_sca_120441	Lox4	PG6 (0.999)	Central (1)
	(-) sca_12:2767594-2768703	fgenesh2_pg.C_sca_12000242	Lox2	PG6 (1)	Central (1)
	(-) sca_12:2815596-2817267	gw1.12.448.1	Post2	PG12 (1)	Posterior (1)
Lottia gigantea	(+) sca_12:2913905-2921811	gw1.12.658.1	Post1	CTL (0.942)	Posterior (1)
	(-) sca_80:336370-336576	gw1.80.268.1	Xlox	PG3 (1)	Xlox (1)
	(-) sca_80:362865-363038	gw1.80.263.1	Gsx	PG3 (1)	Gsx (1)
	(-) sca_30:1633167-1633517	e_gw1.30.54.1	Gbx	PG4 (1)	CTL (1)
	(-) sca_12:3355560-3360804	gw1.12.378.1	Mnr	PG4 (1)	CTL (1)
	(+) sca_12:3699383-3704915	gw1.12.28.1	Mox	PG4 (1)	CTL (1)
	(-) sca_12:3771326-3771821	gw1.12.57.1	Mox	PG4 (1)	CTL (1)
	(+) sca_85:64719-65385	gw1.85.148.1	Cdx	CTL (1)	Cdx (1)
	(-) sca_85:69967-76013	estExt_Genewise1.C_sca_850005	Cdx	PG6 (0.999)	Cdx (1)

Table S4: HoxPred predictions for genome-scale analyses of homeodomain sequences. The posterior probability associated with each HoxPred prediction is shown in parentheses. The protein names have been retrieved from the automatic annotation of the draft assemblies. The orientation of the gene is indicated: (-) reverse (+) forward strands.

Species	Genomic localisation	JGI identifier	Protein name	HoxPred assignation	
				Vert_relaxed	Bilateria
**Annelid**					
	(-) scaffold_70:155465-161596	estExt_fgenesh1_pg.C_700021	Hox1	PG1 (1)	Anterior (1)
	(-) scaffold_70:173028-173815	e_gw1.70.61.1	Hox2	PG2 (1)	Anterior (1)
	(-) scaffold_70:189986-191934	estExt_fgenesh1_pg.C_700024	Hox3	PG3 (1)	Anterior (1)
	(-) scaffold_70:250366-252257	estExt_Genewise1.C_700045	Hox4	PG4 (1)	Central (1)
	(-) scaffold_70:283623-289869	estExt_Genewise1Plus.C_700047	Hox5	PG5 (1)	Central (1)
	(-) scaffold_70:299228-303202	estExt_Genewise1Plus.C_700050	Hox6	PG7 (1)	Central (1)
	(-) scaffold_70:320257-326393	e_gw1.70.47.1	Hox7	PG7 (1)	Central (1)
	(-) scaffold_70:394020-397750	e_gw1.70.60.1	Hox8	PG6 (0.999)	Central (1)
Capitella sp. I	(-) scaffold_292:24288-28949	estExt_fgenesh1_pg.C_2920001	Lox2	PG6 (1)	Central (1)
	(-) scaffold_292:40858-41257	e_gw1.292.19.1	Post2	CTL (1)	Posterior (1)
	(+) scaffold_33:423772-423957	gw1.33.125.1	Post1	CTL (1)	Posterior (1)
	(-) scaffold_313:59222-67802	estExt_Genewise1Plus.C_3130021	Mox	PG4 (1)	CTL (1)
	(-) scaffold_36:673805-678104	estExt_Genewise1.C_360086	Gbx	PG4 (1)	CTL (1)
	(-) scaffold_760:63083-64717	estExt_Genewise1.C_7600007	Gsh	PG3 (1)	Gsx (1)
	(+) scaffold_444:80326-87289	fgenesh1_pg.C_scaffold_444000011	Xlox	PG3 (0.999)	Xlox (1)
	(-) scaffold_444:40713-47085	e_gw1.444.23.1	Cdx	CTL (1)	Cdx (1)
	(+) scaffold_18:1046599-1046820	e_gw1.18.96.1	Lox7 / Hox1	PG1 (1)	Anterior (1)
	(-) scaffold_18:989468-1000771	e_gw1.18.37.1	Lox18 / Hox4	PG5 (1)	Central (1)
	(+) scaffold_18:936073-943566	e_gw1.18.97.1	Lox2 / Abd-A	PG6 (0.999)	Central (1)
	(+) scaffold_18:832989-835094	estExt_Genewise1Plus.C_180102	Post2	PG12 (0.967)	Posterior (1)
	(-) scaffold_90:401000-403305	gw2.90.17.1	Lox7 / Hox1	PG1 (1)	Anterior (1)
	(-) scaffold_90:460560-462620	e_gw1.90.8.1	Lox20 / Hox4 / Hox5	PG5 (1)	Central (1)
	(-) scaffold_90:467415-470824	estExt_Genewise1Plus.C_900048	Lox5 / Antp	PG7 (1)	Central (1)
	(+) scaffold_63:328025-329287	e_gw1.63.157.1	Lox18 / Hox5	PG5 (1)	Central (1)
	(+) scaffold_63:303221-303791	e_gw1.63.77.1	Antp-like	PG6 (0.877)	Central (1)
	(+) scaffold_108:54453-60503	e_gw1.108.1.1	Lox6 / Hox4	PG5 (1)	Central (1)
	(-) scaffold_108:597-7266	fgenesh4_pg.C_scaffold_108000001	Lox2 / Abd-A	PG6 (0.999)	Central (1)
Helobdella robusta	(-) scaffold_22:1169127-1174343	gw2.22.102.1	Lox20 / Hox5	PG5 (1)	Central (1)
	(-) scaffold_22:1294561-1301592	e_gw1.22.27.1	Lox2 / Abd-A	PG6 (0.999)	Central (1)
	(-) scaffold_23:2743557-2745328	e_gw1.23.267.1	Hox3	PG3 (1)	Anterior (1)
	(-) scaffold_231:13890-14378	e_gw1.231.3.1	Lox20 / Hox5	PG5 (1)	Central (0.972)
	(-) scaffold_1448:591-3309	gw2.1448.2.1	Hox5	CTL (0.999)	Central (0.999)
	(-) scaffold_9:1760763-1761190	gw2.9.567.1	?	PG5 (0.999)	Central (0.963)
	(+) scaffold_13:3381895-3382598	gw2.13.147.1	Post2	CTL (1)	Posterior (1)
	(+) scaffold_103:255334-255540	e_gw1.103.6.1	Post2	CTL (1)	Posterior (1)
	(+) scaffold_40:1687050-1687244	gw2.40.136.1	Nk1	PG5 (0.967)	CTL (1)
	(+) scaffold_36:300155-301543	e_gw1.36.193.1	Mox	PG9 (0.999)	CTL (1)
	(+) scaffold_14:347936-348695	e_gw1.14.620.1	Gbh	PG4 (1)	CTL (1)
	(+) scaffold_30:192568-197426	gw2.30.421.1	extra-extra	PG4 (1)	CTL (1)
	(-) scaffold_30:2147548-2152023	estExt_Genewise1.C_300434	Xlox	PG3 (1)	Xlox (1)
	(-) scaffold_18:1483211-1485093	estExt_Genewise1.C_180241	Gsh	PG3 (0.984)	Gsx (1)
	(-) scaffold_53:985556-985913	gw2.53.143.1	Gsh	CTL (1)	Gsx (1)
	(-) scaffold_39:241791-242344	gw2.39.154.1	Cdx	CTL (1)	Cdx (1)

				HoxPred assignation	
**Species**	**Genomic localisation**	**JGI identifier**	**Protein name**	**Vert_relaxed**	**Bilateria**
**Cephalochordates**					
	(+) scaffold_402:248453-250377	estExt_fgenesh2_kg.C_4020003	Hox1	PG1 (1)	Anterior (1)
	(+) scaffold_402:241803-242105	e_gw.402.17.1	Hox2	PG2 (1)	Anterior (1)
	(+) scaffold_402:228121-236046	fgenesh2_pg.scaffold_402000006	Hox3	PG3 (1)	Anterior (1)
	(+) scaffold_402:187111-189331	estExt_fgenesh2_pg.C_4020005	Hox4	PG4 (1)	Central (1)
	(+) scaffold_402:110567-110812	e_gw.402.27.1	Hox7	PG7 (1)	Central (1)
	(+) scaffold_402:92321-92596	e_gw.402.19.1	Hox8	PG7 (0.878)	Central (1)
	(+) scaffold_402:78786-79022	e_gw.402.47.1	Hox9	PG9 (1)	Posterior (1)
	(+) scaffold_260:1100511-1102896	e_gw.260.11.1	Hox5	PG5 (1)	Central (1)
	(+) scaffold_260:1091790-1093056	fgenesh2_pg.scaffold_260000039	Hox6	PG7 (1)	Central (1)
	(+) scaffold_260:1022851-1024410	e_gw.260.32.1	Hox7	PG7 (1)	Central (1)
	(+) scaffold_260:1006152-1006364	e_gw.260.61.1	Hox8	PG6 (0.999) (PG7*)	Central (1)
Branchiostoma floridae	(+) scaffold_260:992285-992521	e_gw.260.52.1	Hox9	PG9 (1)	Posterior (1)
	(+) scaffold_260:907047-907322	e_gw.260.79.1	Hox10	PG10 (1)	Posterior (1)
	(+) scaffold_260:852581-854259	gw.260.58.1	Hox12	PG9 (0.998)	Posterior (1)
	(+) scaffold_260:844739-844975	e_gw.260.40.1	Hox13	PG10 (1)	Posterior (1)
	(+) scaffold_260:784555-789846	e_gw.260.37.1	Hox14	PG13 (0.999) (PG9*)	Posterior (1)
	(+) scaffold_260:670950-673972	e_gw.260.82.1	Hox11	PG9 (1)	Posterior (1)
	(+) scaffold_260:655347-657048	gw.260.71.1	Hox12	PG9 (0.998)	Posterior (1)
	(+) scaffold_260:646441-646677	e_gw.260.41.1	Hox13	PG10 (1)	Posterior (1)
	(+) scaffold_260:590496-593373	e_gw.260.36.1	Hox14	PG13 (0.999) (PG9*)	Posterior (1)
	(+) scaffold_260:506554-506763	gw.260.83.1	Hox15	PG13 (1)	Posterior (1)
	(+) scaffold_260:428076-428285	gw.260.84.1	Hox15	PG13 (1)	Posterior (1)
	(-) scaffold_544:299313-314313	fgenesh2_kg.scaffold_544000001	Mox	PG4 (1)	CTL (1)
	(+) scaffold_260:66882-81498	fgenesh2_kg.scaffold_260000001	Mox	PG4 (1)	CTL (1)
	(-) scaffold_34:3329708-3333499	estExt_fgenesh2_pg.C_340237	Gbx	PG4 (1)	CTL (1)
	(-) scaffold_407:344931-348126	estExt_gwp.C_4070151	Gbx	PG4 (1)	CTL (1)
	(-) scaffold_601:245826-246640	e_gw.601.16.1	Mnx	PG4 (1)	CTL (1)
	(-) scaffold_169:1130580-1139557	estExt_fgenesh2_pg.C_1690055	Mnx	PG4 (1)	CTL (1)
	(+) scaffold_24:481718-481921	e_gw.24.290.1	Cdx	CTL (1)	Cdx (1)
	(-) scaffold_24:523505-523711	e_gw.24.254.1	Gsh	PG2 (0.771)	Gsx (1)
	(+) scaffold_24:553762-553965	e_gw.24.291.1	Cdx	CTL (1)	Cdx (1)
	(-) scaffold_24:561478-561744	gw.24.415.1	Xlox	PG3 (1)	Xlox (1)
	(-) scaffold_24:601597-601803	gw.24.256.1	Gsh	PG2 (0.771)	Gsx (1)
	(-) scaffold_116:1759788-1763506	fgenesh2_pg.scaffold_116000088	Gsh	PG2 (0.999)	Gsx (1)

(*) Predicted proteins have an incomplete homeodomain. The HoxPred assignation in brackets is the assignation for the complete homeodomain, from sequences found in the Uniprot database.

Species	Genomic localisation	GLEAN identifier	Protein name	HoxPred assignation	
				Vert_relaxed	Bilateria
**Echinoderms**					
Strongylocentrotus purpuratus	(-) scaffold_v2_44497:185676-87427	GLEAN3_17352	Hox1	PG1 (1)	Anterior (1)
	(-) scaffold_v2_44497:271721-273542	GLEAN3_12252	Hox2	PG2 (1)	Anterior (0.999)
	(-) scaffold_v2_44497:291159-291887	GLEAN3_27568	Hox3	PG3 (1)	Anterior (1)
	(+) scaffold_v2_44497:273250-398694	GLEAN3_00388	Hox11/13c	PG9 (1)	Posterior (1)
	(-) scaffold_v2_44497:450814-468241	GLEAN3_02631	Hox11/13b	PG9 (1)	Posterior (1)
	(+) scaffold_v2_44497:525185-543760	GLEAN3_02632	Hox11/13a	PG9 (1)	Posterior (1)
	(-) scaffold_v2_44497:557042-596751	GLEAN3_02630	Hox8	PG7 (1)	Central (1)
	(+) scaffold_v2_44497:603830-604087	GLEAN3_02633	Hox9/10	PG9 (1)	Posterior (1)
	(+) scaffold_v2_44497:630995-644222	GLEAN3_21309	Hox8	PG7 (1)	Central (1)
	(-) scaffold_v2_33324:178158-184449	GLEAN3_05170	Hox7	PG7 (0.998)	Central (1)
	(-) scaffold_v2_33324:125248..144906	GLEAN3_05171	Hox6	PG7 (1)	Central (1)
	(+) scaffold_v2_69286:754181-767593	GLEAN3_05169	Hox5	PG5 (1)	Central (1)
	(-) scaffold_v2_88553:243840.-246578	GLEAN3_11798	?	CTL (1)	Posterior (0.599)
	(+) scaffold_v2_72701:253059-262686	GLEAN3_13436	Sp-Gsx	PG3 (1)	Gsx (1)
	(-) scaffold_v2_75781:156598-181301	GLEAN3_26099	Sp-lox	PG3 (1)	Xlox (1)
	(-) scaffold_v2_62059:140430-152201	GLEAN3_24715	Sp-Cdx	PG6 (0.999)	Cdx (1)
	(-) scaffold_v2_6414:198663-227633	GLEAN3_02816	Sp-Hb9	PG4 (1)	CTL (1)
	(-) scaffold_v2_67899:96754-113575	GLEAN3_12491	Sp-Nk1	PG4 (0.977)	CTL (1)
	(-) scaffold_v2_50970:31414-43231	GLEAN3_25486	Sp-Mox	PG4 (1)	CTL (1)

Species	Accession number	Protein name	Reference assignation	HoxPred assignation		
				Vert relaxed	Bilateria	Bilateria relaxed
**Cnidaria**						
Nematostella vectensis	Q9Y1T7_NEMVE	HoxA/anthox6	Anterior/Group 3	PG6 (0.999)	CTL (0.999)	Anterior (1)
	Q0N4N3_NEMVE	HoxB/anthox6a	Anterior/Group 3	PG3 (0.999)	Anterior (0.998)	Anterior (1)
	Q9GR14_NEMVE	HoxC/anthox7	Anterior/Group 3	PG3 (1)	Anterior (0.993)	Anterior (1)
	Q0ZRJ7_NEMVE	HoxDa/anthox8a	Anterior/Group 3	PG3 (1)	Anterior (0.953)	Anterior (1)
	Q0ZP93_NEMVE	HoxDbanthox8b	Anterior/Group 3	PG3 (1)	Anterior (0.953)	Anterior (1)
	Q0ZRK9_NEMVE	HoxF/anthox1	Central/Posterior/none	PG5 (1)	CTL (1)	Central (0.999)
	Q0ZRF9_NEMVE	HoxE/anthox1a	Central/Posterior/none	PG5 (1)	CTL (0.983)	Central (1)
	Q0ZRK6_NEMVE	Hox-like/NVHD117	Gsx-related/pseudogene	CTL (1)	CTL (1)	Anterior (0.997)
	Q0ZRK1_NEMVE	Gsx/Hox2	ParaHox (Gsx)	PG3 (1)	Gsx (1)	Anterior (1)
	Q0ZPQ5_NEMVE	Xlox-Cdx/NVHD065	ParaHox	PG4 (1)	CTL (1)	Central (0.995)
	Q0ZRI8_NEMVE	CG13424B-like/NVHD021	CG13424-like	PG1 (0.999)	CTL (1)	Anterior (1)
	Q0ZRN3_NEMVE	CG13424A-like/NVHD027	CG13424-like	PG4 (1)	CTL (1)	Anterior (1)
	Q0ZRF7_NEMVE	MOXA/MOXb-ANTP	Mox	PG4 (1)	CTL (1)	Posterior (1)
	Q0ZRF4_NEMVE	MOXB/MOXd-ANTP	Mox	CTL (1)	CTL (1)	Posterior (1)
	Q0ZRJ0_NEMVE	MOXC/MOXc-ANTP	Mox	CTL (1)	CTL (1)	Posterior (1)
	Q0ZRI3_NEMVE	MOXD/MOXa-ANTP	Mox	PG4 (1)	CTL (1)	Posterior (0.983)
	Q0ZRH2_NEMVE	Mnx/HLXB9-ANTP	Mnx	PG6 (0.996)	CTL (1)	Anterior (0.999)
	Q0ZRJ1_NEMVE	Gbx	Gbx	CTL (1)	CTL (1)	Anterior (0.999)
	Q0ZRK3_NEMVE	Rough	Rough	CTL (1)	CTL (1)	Anterior (1)
	Q0R511_NEMVE	Evx	Evx	CTL (1)	CTL (1)	Anterior (1)
Acropora formosa	Q7M3U4 _ACRFO	protein antpC	Anterior	PG1 (1)	Anterior (0.931)	Anterior (1)
Podocoryne carnea	Q25679 _PODCA	Hox1 protein	Anterior	PG1 (1)	CTL (0.962)	Anterior (1)
	Q9NLA0 _PODCA	Cnox2-Pc	Central ?	PG5 (0.993)	CTL (1)	Anterior (1)
	Q962C9 _PODCA	Cnox4-Pc	?	PG3 (1)	CTL (1)	Anterior (0.912)
	Q9GTK8 _PODCA	GSX	ParaHox	PG5 (0.999)	Gsx (0.999)	Anterior (1)
Hydractinia symbiolongicarpus	Q2PWL7 _HYDSY	Cnox-2	ParaHox (Gsx)	PG3 (0.999)	Gsx (0.999)	Anterior (1)
Hydra attenuata	Q25186 _HYDAT	Cnox-1/Cnox-3	?	PG1 (1)	CTL (1)	Anterior (1)
	Q9NFW4 _HYDAT	Cnox-3	?	PG6 (0.663)	CTL (0.999)	Posterior (0.999)
	Q25182 _HYDAT	Cnox-2	ParaHox (Gsx)	PG3 (0.999)	Gsx (0.999)	Anterior (1)
Hydra viridis	Q2UZ91 _CHLVR	Cnox-2	ParaHox (Gsx)	PG3 (0.999)	Gsx (0.999)	Anterior (1)
Eleutheria dichotoma	Q1HP68 _9CNID	Cnox-5	Anterior	PG1 (1)	Anterior (0.900)	Anterior (1)
	Q24781 _9CNID	Cnox-4	Posterior/Cdx?	CTL (1)	CTL (1)	Central (1)
	Q24780 _9CNID	Cnox-3	Posterior?	PG4 (0.794)	CTL (0.999)	Posterior (0.999)
	Q1HP71 _9CNID	Cnox-1	?	PG7 (0.763)	CTL (1)	Central (1)
Cassiopea xamachana	Q9XYT9 _CASXA	Scox-5	Central/Posterior ?	CTL* (1)	Posterior* (0.994)	Central* (0.999)
	Q9XYT7 _CASXA	Scox-3	Central/Posterior ?	PG4 (0.999)	CTL (0.999)	Central (1)
	Q9XYT5 _CASXA	Scox-1	Central/Posterior ?	PG6 (1)	CTL (0.763)	Posterior (0.774)
	Q9XYT8 _CASXA	Scox-4	Central/Posterior ?	PG9 (1)	CTL (1)	Posterior (0.998)
	Q9XYT6 _CASXA	Scox-2	ParaHox (Gsx)	PG3 (0.999)	Gsx (0.999)	Anterior (1)
Hydra magnipapillata	-*	HmHoxa/cnox4	Anterior	PG1 (1)	CTL (1)	Anterior (1)
	-*	HmHoxb	?	PG4 (1)	CTL (1)	Posterior (1)
	-*	HmHoxc1	?	PG6 (0.999)	CTL (0.999)	Posterior (0.999)
	-*	HmHoxc2	?	PG6 (0.996)	CTL (0.990)	Central (0.998)
	-*	HmHoxc3	?	PG7 (0.908)	CTL (0.999))	Central (1)
	-*	HmHoxd	?	CTL (1)	CTL (0.999)	Posterior (1)
	-*	HmGsx	ParaHox (Gsx)	PG3 (0.999)	Gsx (0.999)	Anterior (1)
	-*	HmMox/cnox5	Mox ?	PG4 (0.999)	CTL (0.999)	Posterior (1)
Clytia hemisphaerica	B9V2D2_9CNID	Hox1	Anterior	PG1 (1)	CTL (0.999)	Anterior (1)
	B9V2D3_9CNID	Hox9-14A	Posterior	PG5 (1)	CTL (0.999)	Posterior (0.993)
	B9V2D4_9CNID	Hox9-14B	Posterior	CTL (1)	CTL (0.999)	Posterior (1)
	B9V2D5_9CNID	Hox9-14C	Posterior	PG6 (0.932)	CTL (1)	Central (1)
	B9V2C9_9CNID	Gsx	ParaHox (Gsx)	PG3 (0.999)	Gsx (0.999)	Anterior (1)
	B6ZCD9_9CNID	Xlox	ParaHox (Xlox)	PG5 (1)	CTL (1)	Central (0.999)
	B9V2C5_9CNID	Cdx	ParaHox (Cdx)	CTL (1)	CTL (1)	Central (1)
Turritopsis dohrnii	B6ZCE4_9CNID	Xlox	ParaHox (Xlox)	PG5 (1)	CTL (1)	Central (1)
	B6ZCE3_9CNID	CnoxA	?	PG4 (0.999)	CTL (1)	Anterior (1)
Cladonema radiatum	B6ZCE2_9CNID	CnoxA	?	PG4 (0.999)	CTL (1)	Anterior (1)
**Placozoa**						
Trichoplax adhaerens	Q6VYH7_9METZ	Trox-2	ParaHox Gsx ?	PG3 (1)	Gsx (1)	Anterior (0.998)
	Q27W40_9METZ	Mnx	Mnx	PG4 (0.997)	CTL (1)	Central (1)

Table S5: Classification of Cnidaria and Placozoa Hox-related proteins by HoxPred. The posterior probability associated with each HoxPred prediction is shown in parentheses. *Hydra magnipapillatta* sequences from Chourrout et al, Nature, 2006 have not been deposited in public databases. The *Scox-5* homeodomain sequence of *Cassiopea xamachana* is 14 amino acids short ; its prediction is thus dubious.

ParaHox	Species	Accession number	Protein name	HoxPred assignation		
				Vert relaxed	Bilateria	Bilateria relaxed
Gsx	*Tribolium castaneum*	Q58Y77_TRICA	ind	PG3 (1)	Gsx (1)	Anterior (1)
	Apis mellifera	refseq:XP_001120954.1	ind	PG3 (1)	Gsx (1)	Anterior (1)
	Drosophila melanogaster	Q7KUL4_DROME	ind	PG3 (1)	Gsx (1)	Anterior (1)
	Euprymna scolopes	Q49QY0_9MOLL	Gsx	PG3 (1)	Gsx (1)	Anterio (1)
	Nereis virens	Q0ZRS5_NERVI	Gsx	PG3 (1)	Gsx (1)	Anterior (1)
	Capitella sp. I	Q2FA45_9ANNE	Gsx	PG3 (1)	Gsx (1)	Anterior (1)
	Ptychodera flava	Q6T4Q6_9BILA	Gsx	PG3 (1)	Gsx (1)	Anterior (1)
	Strongylocentrotus purp.	refseq:XP_001189039.1	Gsx	PG3 (1)	Gsx (1)	Anterior (1)
	Branchiostoma floridae	O61586 BRAFL	AmphiGsx	PG2 (1)	Gsx (1)	Anterior (1)
	Ciona intestinalis	Q9BMT5 CIOIN	Ci-Gsx	PG3 (1)	Gsx (1)	Anterior (1)
	Oikopleura dioica	Q5EVF3 OIKDI	Gsx	PG4 (1)	Gsx (1)	Anterior (0.724)
	Diplosoma listerianum	Q7Z1M6_9ASCI	ParaHox-GSX	PG5 (0.999)	Gsx (1)	Central (0.752)
	Mus musculus	GSX1_MOUSE	Gsh1	PG3 (1)	Gsx (1)	Anterior (1)
		GSX2_MOUSE	Gsh2	PG3 (1)	Gsx (1)	Anterior (1)
	Danio rerio	Q58ED8_DANRE	Gsh1	PG3 (1)	Gsx (1)	Anterior (1)
		Q5TZB7_DANRE	Gsx2	PG3 (1)	Gsx (1)	Anterior (1)
Xlox	*Capitella sp. I*	Q2FBI9_9ANNE	Xlox	PG3 (0.999)	Xlox (1)	Anterior (0.999)
	Nereis virens	Q0QIJ2_NERVI	Xlox	PG3 (0.999)	Xlox (1)	Anterior (1)
	Perionyx excavatus	Q533T4_9ANNE	Xlox	PG3 (1)	Xlox (1)	Anterior (1)
	Archaster typicus	Q86M80_9ECHI	Xlox	PG3 (1)	Xlox (1)	Anterior (1)
	Ptychodera flava	Q6T4Q5_9BILA	Lox1	PG3 (1)	Xlox (1)	Anterior (1)
		Q6T4Q4_9BILA	Lox2	PG3 (1)	Xlox (1)	Anterior (1)
	Strongylocentrotus purp.	Q8IT48_STRPU	Splox	PG3 (1)	Xlox (1)	Anterior (1)
	Branchiostoma floridae	Q9TZY9 BRAFL	AmphiXlox	PG3 (1)	Xlox (1)	Anterior (1)
	Ciona intestinalis	Q9GP86_CIOIN	Ci-Xlox	PG3 (1)	Xlox (1)	Anterior (0.984)
	Diplosoma listerianum	Q7Z1M8_9ASCI	ParaHox-XLOX	PG3 (1)	Xlox (1)	Anterior (1)
	Mus musculus	PDX1_MOUSE	Ipf1	PG3 (1)	Xlox (1)	Anterior (0.997)
	Brachydanio rerio	Q6DC85_DANRE	Ipf1	PG5 (0.999)	Xlox (1)	Central (0.999)
Cdx	*Bombyx mori*	Q17243_BOMMO	cad protein	PG6 (0.999)	Cdx (1)	Central (1)
	Tribolium castaneum	O96714_TRICA	caudal	CTL (1)	Cdx (1)	Central (1)
	Aedes aegypti	Q16G14_AEDAE	cdx	PG4 (1)	Cdx (1)	Central (1)
	Drosophila melanogaster	CAD_DROME	caudal CG1759	CTL (1)	Cdx (1)	Central (0.999)
	Gryllus bimaculatus	Q60FK2_GRYBI	caudal	PG6 (0.999)	Cdx (1)	Central (1)
	Artemia franciscana	Q70LF3_ARTSF	caudal	PG6 (0.999)	Cdx (1)	Central (1)
	Euscorpius flavicaudis	Q68LB1_9SCOR	caudal	PG6 (1)	Cdx (1)	Central (1)
	Achaearanea tepidariorum	Q869A1_ACHTE	caudal	PG6 (0.999)	Cdx (1)	Central (1)
	Strigamia maritima	Q698K3_9MYRI	caudal	PG6 (0.999)	Cdx (1)	Central (1)
	Patella vulgata	Q8I757_PATVU	caudal protein	PG5 (0.860)	Cdx (1)	Central (1)
	Nereis virens	Q7Z0F5_NERVI	caudal	PG5 (0.907)	Cdx (1)	Central (1)
	Platynereis dumerilii	Q3LRS0_PLADU	caudal	PG6 (0.973)	Cdx (1)	Central (1)
	Capitella sp. I	Q2FBJ0_9ANNE	caudal	CTL (1)	Cdx (1)	Posterior (1)
	Strongylocentrotus purp.	refseq:XP_789158.2	Cad protein	PG6 (0.999)	Cdx (1)	Central (1)
	Ptychodera flava	Q6T4Q3_9BILA	Cad	PG6 (0.964)	Cdx (1)	Central (1)
	Branchiostoma floridae	Q9TZY8_BRAFL	AmphiCdx	CTL (1)	Cdx (1)	Central (0.999)
	Diplosoma listerianum	Q7Z1M7_9ASCI	ParaHox-CDX	PG6 (1)	Cdx (1)	Central (1)
	Halocynthia roretzi	Q9U8Q3_HALRO	caudal	PG6 (0.999)	Cdx (1)	Central (1)
	Ciona intestinalis	Q4H3T1_CIOIN	Ci-Cdx	PG6 (1)	Cdx (1)	Central (1)
	Oikopleura dioica	Q5EVF1_OIKDI	Cdx-1	CTL (1)	Cdx (1)	Posterior (0.999)
		Q5EVF0_OIKDI	Cdx-2	CTL (1)	Cdx (1)	Central (1)
		Q5EVE9_OIKDI	Cdx-3	CTL (1)	Cdx (1)	Posterior (1)
	Herdmania curvata	Q9NGR3_9ASCI	Cdx	CTL (1)	Cdx (1)	Central (1)
	Mus musculus	CDX1_MOUSE	Cdx1	PG5 (0.646)	Cdx (1)	Central (1)
		CDX2_MOUSE	Cdx2	PG4 (0.842)	Cdx (1)	Central (1)
		CDX4_MOUSE	Cdx4	CTL (1)	Cdx (1)	Central (0.999)
	Danio rerio	Q90262_DANRE	cdx4	CTL (0.980)	Cdx (1)	Central (1)
		Q8AXR4_DANRE	cdx1a	PG6 (0.999)	Cdx (1)	Central (1)

Table S6: Classification of bilaterians ParaHox proteins with HoxPred. The posterior probability associated with each HoxPred prediction is shown in parentheses.
