# Supplementary material for: A non-tree-based comprehensive study of metazoan Hox and ParaHox genes prompts new insights into their origin and evolution
Source: BMC Evol Biol. 2010 Mar 11;10:73. doi: 10.1186/1471-2148-10-73 (PMC2842273; doi:10.1186/1471-2148-10-73)
Supplement: Additional file 3 — Multiple alignments of Hox and ParaHox homeodomains. Alignments of homedomain sequences of Hox and ParaHox sequences from the Datab'Hox database, ordered by homology groups. [file 1471-2148-10-73-S3.PDF]

Save alignment [fasta format] - Save complete sequences [fasta format]

PG1

ID	organism	10 20 30 40 50 60	description
DH_5977	Oikopleura dioica	NGSGRTTFSTRQLTELEKEFHYNKYLTRARRVEIASNLALNETQVKIWFQNRR-------	Hox1 (Fragment) (Hox1)
DH_7337	Oikopleura dioica	NGSGRTTFSTRQLTELEKEFHYNKYLTRARRVEIASNLALNETQVKIWFQNRRMKQKKRD	HOX1 (006-36)
DH_6678	Oikopleura dioica	NGSGRTTFSTRQLTELEKEFHYNKYLTRARRVEIASNLALNETQVKIWFQNRRMKQKKRD	Hox1 (Fragment) (Hox1)
DH_8920	Pachymerium ferrugineum	-------FSTKQLTELEKEFHYNKYLTRARRIEIASALQLNETQVKIW------------	Labial (Fragment) (lab)
DH_4591	Cupiennius salei	NGSGRTNFTTKQLTELEKEFHYNKYLTRARRIEIATALQLNETQVKIWFQNRRMKQKKRM	Homeobox protein (labial)
DH_2466	Nymphon gracile	NNTGRTAFNTKQLTELEKEXHYNKYLTRARRIEIASALSLNETQVKIWFQNRRMKQKKRM	Homeobox protein labial (Lab)
DH_5382	Endeis spinosa	NNTGRTAFNTKQLTELEKEFHFNKYLTRARXIEIASALSLNETQVKIWFQNRRMKQKKRM	Homeobox protein labial (Fragment) (Lab)
DH_235	Caenorhabditis elegans	NGTNRTNFTTHQLTELEKEFHTAKYVNRTRRTEIASNLKLQEAQVKIWFQNRRMKEKKRE	Homeobox protein ceh-13 (ceh-13)
DH_2345	Caenorhabditis briggsae	NGTNRTNFTTHQLTELEKEFHTAKYVNRTRRTEIASNLKLQEAQVKIWFQNRRMKEKKRE	CBR-CEH-13 protein (Cbr-ceh-13)
DH_607	Gallus gallus	AFSLRTSFSTRQLTELEKEFHFSRYLSRARRLEVARSLRLRDAQVKVWFQNRRMKQKKRE	Homeobox protein Hox-D1 (Fragment) (HOXD1)
DH_5396	Eleutheria dichotoma	ICKKRVCFTQKQIVELEKEFHYNRYLTRARRVEIAQLLKLTEAQIKIWFQNRRMKQKREQ	Cnox-5
DH_5566	Eleutheria dichotoma	ICKKRVCFTQKQIVELEKEFHYNRYLTRARRVEIAQLLKLTEAQIKIWFQNRRMKQKREQ	Cnox5 homeodomain protein
DH_7821	Acropora formosa	SNKKRFTFTQRQLVELEKEFHFSKYLTRTRRIEIASNLDLTETQIKIWFQNRRMKWKREL	Homeotic protein antpC
DH_8434	Metridium senile	---KRFTFTQLQLVELEKEFHFSKYLTRSRRIEIATSLKLTETQIKIWFQNRRM------	Homeobox protein anthox6 (Fragment) (anthox6)
DH_7688	Schistosoma mansoni	SLSGRTNFTTRQLTELEKEFHFNRYLSRARRIEIAADLNLTETQVKIWFQNRRMKQKKRI	Labial-like protein (Fragment)
DH_8004	Latimeria menadoensis	VNTARTNFTTKQLTELEKEFHFNKYLSRTRRIEIANALHLNETQVKIWFQNRRMKQKKRG	HoxD1 (Fragment)
DH_370	Rattus norvegicus	PNAVRTNFTTKQLTELEKEFHFNKYLTRAR-SEIAASLQLNETQVKIWFQNRRMKQKKRE	Homeobox protein Hox-A1 (Hoxa1)
DH_368	Homo sapiens	PNAVRTNFTTKQLTELEKEFHFNKYLTRARRVEIAASLQLNETQVKIWFQNRRMKQKKRE	Homeobox protein Hox-A1 (HOXA1)
DH_1689	Homo sapiens	PNAVRTNFTTKQLTELEKEFHFNKYLTRARRVEIAASLQLNETQVKIWFQNRRMKQKKRE	Homeo box A1 (Homeobox A1, isoform CRA_a) (HOXA1)
DH_2831	Homo sapiens	PNAVRTNFTTKQLTELEKEFHFNKYLTRARRVEIAASLQLNETQVKIWFQNRRMKQKKRE	cDNA, FLJ94072, highly similar to Homo sapiens homeo box A1 (HOXA1), transcript variant 1, mRNA
DH_2573	Callithrix jacchus	PNAVRTNFTTKQLTELEKEFHFNKYLTRARRVEIAASLQLNETQVKIWFQNRRMKQKKRE	Homeobox A1 isoform a (Predicted) (HOXA1)
DH_2443	Papio anubis	PNAVRTNFTTKQLTELEKEFHFNKYLTRARRVEIAASLQLNETQVKIWFQNRRMKQKKRE	Homeobox A1, isoform 1 (Predicted) (HOXA1)
DH_2743	Callicebus moloch	PNAVRTNFTTKQLTELEKEFHFNKYLTRARRVEIAASLQLNETQVKIWFQNRRMKQKKRE	Homeobox A1 isoform a (Predicted) (HOXA1)
DH_2807	Rhinolophus ferrumequinum	PNAVRTNFTTKQLTELEKEFHFNKYLTRARRVEIAASLQLNETQVKIWFQNRRMKQKKRE	Homeobox A1 isoform a (Predicted) (HOXA1)
DH_369	Mus musculus	PNAVRTNFTTKQLTELEKEFHFNKYLTRARRVEIAASLQLNETQVKIWFQNRRMKQKKRE	Homeobox protein Hox-A1 (Hoxa1)
DH_8136	Neochildia fusca	-----------------KEFHFNKYLTRARRVEIAASLQLNETQVKIWF-----------	Hox-type homeodomain-containing protein (Fragment)
DH_8306	Mus musculus	PNAVRTNFNTKQLTELEKEFHFNKYLTRARRVEIAASLQLNETQVKIWFQNRRMKQKKRE	Putative uncharacterized protein (Hoxa1)
DH_372	Danio rerio	PNTVRTNFSTKQLTELEKEFHFNKYLTRARRVEIAASLQLNETQVKIWFQNRRMKQKKRE	Homeobox protein Hox-A1a (hoxa1a)
DH_2911	Danio rerio	PNTVRTNFSTKQLTELEKEFHFNKYLTRARRVEIAASLQLNETQVKIWFQNRRMKQKKRE	Hoxa1a protein (hoxa1a)
DH_5436	Danio rerio	PNTVRTNFSTKQLTELEKEFHFNKYLTRARRVEIAASLQLNETQVKIWFQNRRMKQKKRE	Homeo box A1a (hoxa1a)
DH_8511	Petromyzon marinus	IATQRTNFSTKQLTELEKEFHFNKYLTRARRVEIAAALQLNETQVKIWFQNRRMKQKKRE	HoxQ8 (Fragment) (HoxQ8)
DH_1805	Lampetra japonica	IATQRTNFSTKQLTELEKEFHFNKYLTRARRVEIAAALQLNETQVKIWFQNRRMKQKKRE	LjHox1w homeobox (Fragment) (LjHox1w)
DH_8605	Petromyzon marinus	IATHRTNFSTKQLTELEKEFHFNKYLTRARRVEIAAALQLNETQVKIWFQNRRMKQKKRE	Hox1w (Fragment) (hox1w)
DH_9400	Oryzias latipes	--------------------HFNKYLTRARRVEIAAALQLNETQVKIWFQNRRMKQKKRE	HOXA1A (Fragment) (hoxa1a)
DH_8036	Polyodon spathula	-------------------FHFSNYLTRARRVEIAAALQLNETQVKIWFQNRRMKQKKRE	Transcription factor Hoxa1 (Fragment) (Hoxa1)
DH_11802	Gasterosteus aculeatus	ANTVRTNFTTKQLTELEKEFHFNKYLTRARRVEIAAALQLNETQVKIWFQNRRMKQKKRE	Homeobox protein Hox-A1 (Hox-1F) [Source:UniProtKB/Swiss-Prot;Acc:P49639]
DH_3291	Salmo salar	SNTVRTNFTTKQLTELEKEFHFNKYLTRARRVEIAAALQLNETQVKIWFQNRRMKQKKRE	Homeobox protein HoxA1aa (HoxA1aa)
DH_7313	Xenopus tropicalis	PNTARTNFTTKQLTELEKEFHFNKYLTRARRVEIAAALQLNETQVKIWFQNRRMKQKKRE	Homeobox A1 (hoxa1)
DH_7983	Xenopus laevis	PNTARTNFTTKQLTELEKEFHFNKYLTRARRVEIAAALQLNETQVKIWFQNRRMKQKKRE	Hoxa1-A protein (hoxa1)
DH_371	Xenopus laevis	PNTARTNFTTKQLTELEKEFHFNKYLTRARRVEIAAALQLNETQVKIWFQNRRMKQKKRE	Homeobox protein Hox-A1 (Fragment) (hoxa1)
DH_373	Fugu rubripes	PNTVRTNFTTKQLTELEKEFHFNKYLTRARRVEIAAALQLNETQVKIWFQNRRMKQKKRE	Homeobox protein Hox-A1a (hoxa1a)
DH_6539	Tetraodon nigroviridis	PNTVRTNFTTKQLTELEKEFHFNKYLTRARRVEIAAALQLNETQVKIWFQNRRMKQKKRE	Chromosome 21 SCAF14577, whole genome shotgun sequence (GSTENG00017483001)
DH_6190	Oryzias latipes	PNTVRTNFTTKQLTELEKEFHFNKYLTRARRVEIAAALQLNETQVKIWFQNRRMKQKKRE	HoxA1a (hoxA1a)
DH_2156	Haplochromis burtoni	PNTVRTNFTTKQLTELEKEFHFNKYLTRARRVEIAAALQLNETQVKIWFQNRRMKQKKRE	Hoxa1a (hoxa1a)
DH_1522	Megalobrama amblycephala	PNTVRTNFTTKQLTELEKEFHFNKYLTRARRVEIAAALQLNETQVKIWFQNRRMKQKKRE	Homeodomain protein (Fragment) (HoxA1a)
DH_8029	Latimeria menadoensis	PNTVRTNFTTKQLTELEKEFHFNKYLTRARRVEIAAALQLNETQVKIWFQNRRMKQKKRE	HoxA1 (Fragment)
DH_367	Heterodontus francisci	PNTVRTNFTTKQLTELEKEFHFNKYLTRARRVEIAAALQLNETQVKIWFQNRRMKQKKRE	Homeobox protein Hox-A1 (HOXA1)
DH_7658	Perionyx excavatus	-----TNFTNKQLTELEKEFHFSRYLTRSRRIEIAASLGLNETQIKIWFQNRRMKQKKR-	Homeobox protein lab01 (Fragment)
DH_7009	Urechis unicinctus	-----TNFTNKQLTELEKEFHFSRYLTRSRRIEIAASLGLNETQIKIWFQNRR-------	Homeobox protein lab01 (Fragment)
DH_7963	Symsagittifera roscoffensis	TRGGRTNFTNKQLTELEKEFHFNRYLTRARRIEIATSLTLNETQVKIWFQTRRMKQKKLL	Labial-like homeobox protein SrHox1 (Fragment) (Hox1)
DH_7657	Perionyx excavatus	-----TNFTNKQLTELEKEFHFNRYLTRARRIEIASSLGLNETQVKIWFQNRRMKQKKR-	Homeobox protein lab02 (Fragment)
DH_12067	Capitella sp. I Grassle and Grassle, 1976	--MGRTNFTNKQLTELEKEFHFNKYLTRARRIEIAASLGLNETQVKIWFQNRRMKQKKRL	estExt_fgenesh1_pg.C_700021|Capca1
DH_12223	Lottia gigantea	PNSGRTNFTNKQLTELEKEFHFNKYLTRARRIEIAASLGLNETQVKIWFQNRRMKQKKR-	gw1.12.670.1|Lotgi1
DH_7008	Urechis unicinctus	-----TNFTNKQLTELEKEFHFNKYLTRARRIEIAAALGLNETQIKIWFQNRR-------	Homeobox protein lab02 (Fragment)
DH_12403	Helobdella robusta	SNLGRTNFTNKQLTELEKEFHFNKYLTRARRIEIAAALGLNETQVKIWFQNRRMKQKKRV	gw2.90.17.1|Helro1
DH_8355	Platynereis dumerilii	PNMGRTNFTNKQLTELEKEFHFNKYLTRARRIEIAAALGLNETQVKIWFQNRRMKQKKRM	Homeobox protein (hox1)
DH_9486	Nereis virens	PNMGRTNFTNKQLTELEKEFHFNKYLTRARRIEIAAALGLNETQVKIWFQNRRMKQKKRM	Labial homeobox protein (Fragment) (lab)
DH_9465	Chaetopterus variopedatus	PNLGRTNFTNKQLTELEKEFHFNKYLTRARRIEIAAALGLNETQVKIWFQNRRMKQKKRM	Homeoprotein CH-Hox1 (Hox1)
DH_4547	Lineus sanguineus	PNTGRTNFTNKQLTELEKEFHFNKYLTRARRIEIAAALGLNETQVKIWFQNRRMKQKKRM	Homeodomain protein (Fragment) (Hox-1)
DH_9140	Discocelis tigrina	KGNGRTNFTNKQLTELEKEFYFNKYLTRARRIEIANALGLNETQVKIWFQNRRMKQKKRM	Hox1/lab protein (Fragment) (hoxA)
DH_7656	Perionyx excavatus	-----TNFTNKQLTELEKEFHFNKYLTRARRIEIAAALGLNETQVKIWFQNRRMKQKKR-	Homeobox protein lab03 (Fragment)

DH_7260	Ilyanassa obsoleta	-----------QLEELEKEFHFNKYLTRARRIEIAASLGLNETQVKIWFQNRRA------	Hox1-like protein variant A (Fragment)
DH_7259	Ilyanassa obsoleta	-----------QLEELEKEFHFNKYLTRARRIEIAAALGLNETQVKIWFQNRRA------	Hox1-like protein variant B (Fragment)
DH_9495	Lingula unguis	PNMGRTNFSDKQLTELEKEFHFNKYLTRARRIEIAAALGLNETQVKIWFQNRRMKQKKRM	Labial homeodomain protein (Fragment)
DH_7672	Ptychodera flava	PNNGRTNFTNKQLTELEKEFHFNKYLTRARRVEIAAMLGLNETQVKIWFQNRRMKQKKRY	Transcription factor Hox1
DH_7929	Saccoglossus kowalevskii	PANGRTNFTNKQLTELEKEFHFNKYLTRARRVEIAAMLGLNETQVKIWFQNRRMKQKKRF	Hox1
DH_5579	Halocynthia roretzi	AGNGRTNFTTKQLTELEKEFHFNKYLTRARRVEIAAALLLNETQVKIWFQNRRMKQKKRD	HrHox-1 protein (HrHox-1)
DH_5702	Polyandrocarpa misakiensis	--------------------HFNKYLTRARRVEIAAALMLNETQVKIWFQNRRMKQKKRD	Homeodomain protein PmHox1 (Fragment) (PmHox1)
DH_6283	Ciona intestinalis	GNNGRTNFTTKQLTELEKEFHFNKYLTRARRVEIAAALRLNETQVKIWFQNRRMKQKKRD	Transcription factor protein (Ci-Hox1)
DH_8361	Ciona intestinalis	GNNGRTNFTTKQLTELEKEFHFNKYLTRARRVEIAAALRLNETQVKIWFQNRRMKQKKRD	Putative homeobox protein hox1 (Fragment) (hox1)
DH_9512	Branchiostoma floridae	PNNGRTNFTTKQLTELEKEFHYNKYLTRARRVEIAAALNLNETQVKIWFQNRRMKQKKRE	AmphiHox1 protein (AmphiHox1)
DH_12677	Branchiostoma floridae	PNNGRTNFTTKQLTELEKEFHYNKYLTRARRVEIAAALNLNETQVKIWFQNRRMKQKKRE	AmphiHox1
DH_11611	Branchiostoma floridae	PNNGRTNFTTKQLTELEKEFHYNKYLTRARRVEIAAALNLNETQVKIWFQNRRMKQKKRE	estExt_fgenesh2_kg.C_4020003|Brafl1
DH_5288	Branchiostoma floridae	PNNGRTNFTTKQLTELEKEFHYNKYLTRARRVEIAAALNLNETQVKIWFQNRRMKQKKRE	Hox-1 homeodomain protein (Fragment)
DH_11719	Gasterosteus aculeatus	HNVIRTNFTTKQLTELEKEFHFNKYLTRGRRVEVAASLELNETQVKIWFQNRRMKQKKRE	groupV
DH_1869	Morone saxatilis	HNVIRTNFTTKQLTELEKEFHFNKYLTRARRVEVAASLELNETQVKVWFQNRRMKQKKRE	Hoxb1b
DH_2160	Haplochromis burtoni	HNVIRTNFTTKQLTELEKEFHFNKYLSRARRVEVAASLELNETQVKIWFQNRRMKQKKRE	Hoxb1b (hoxb1b)
DH_6717	Oreochromis niloticus	HNVIRTNFTTKQLTELEKEFHFNKYLSRARRVEVAASLELNETQVKIWFQNRRMKQKKRE	Hox protein (Fragment)
DH_472	Fugu rubripes	HNVIRTNFTTKQLTELEKEFHFNKYLTRARRVEVAASLELNETQVKIWFQNRRMKQKKRE	Homeobox protein Hox-B1b (hoxb1b)
DH_6525	Tetraodon nigroviridis	HNAIRTNFTTKQLTELEKEFHFNKYLTRARRVEVAASLELNETQVKIWFQNRRMKQKKRE	Chromosome 2 SCAF14604, whole genome shotgun sequence (GSTENG00019145001)
DH_2427	Cynolebias cheradophilus	--------------ELEKEFHFNKYLTRARRVEVAASLELNETQVKIWFQNR--------	Hox1 (Fragment)
DH_2428	Cynolebias viarius	--------------ELEKEFHFNKYLTRARRVEVAASLELNETQVKIWFQNR--------	Hox1 (Fragment)
DH_2424	Cynolebias gymnoventris	--------------ELEKEFHFNKYLTRARRVEVAVSLELNETQVKIWFQNR--------	Hox1 (Fragment)
DH_471	Danio rerio	QNIIRTNFTTKQLTELEKEFHFNKYLTRARRVEVAATLELNETQVKIWFQNRRMKQKKRE	Homeobox protein Hox-B1b (hoxb1b)
DH_1677	Danio rerio	QNIIRTNFTTKQLTELEKEFHFNKYLTRARRVEVAATLELNETQVKIWFQNRRMKQKKRE	Homeo box B1b (hoxb1b)
DH_1533	Megalobrama amblycephala	QNIIRTNFTTKQLTELDKDFHFNKYFTRARSVEVAATLDLNETQVKMWFQNRRMKQKKRE	Homeodomain protein (Fragment) (HoxB1b)
DH_9347	Xenopus laevis	QNIIRTNFTTKQLTELEKEFHFNKYLTRARRVEIAATLELNETQVKIWFQNRRMKQKKRE	XHOX2.9 homeodomain protein (Fragment)
DH_458	Ambystoma mexicanum	QNSIRTNFTTKQLSELEKEFHFNKYLTRARRVEIAATLELNETQVKIWFQNRRMKQKKRE	Homeobox protein Hox-B1 (Fragment)
DH_461	Cyprinus carpio	QNTIRTNFTTKQLTELEKEFHFSKYLTRARRVEIAATLELNETQVKIWFQNRRMKQKKRE	Homeobox protein Hox-B1 (hoxb1)
DH_1532	Megalobrama amblycephala	QNTIRTNFTTKQLTELEKEFHFSKYLTRARRVEIAATLELNETQVKIWFQNRRMKQKKRE	Homeodomain protein (Fragment) (HoxB1a)
DH_469	Danio rerio	QNTIRTNFTTKQLTELEKEFHFSKYLTRARRVEIAATLELNETQVKIWFQNRRMKQKKRE	Homeobox protein Hox-B1a (hoxb1a)
DH_2917	Danio rerio	QNTIRTNFTTKQLTELEKEFHFSKYLTRARRVEIAATLELNETQVKIWFQNRRMKQKKRE	Hoxb1a protein (hoxb1a)
DH_3314	Salmo salar	QNTIRTNFTTKQLTELEKEFHFSKYLTRARRVEIAATLELNETQVKIWFQNRRMKQKKRE	Homeobox protein HoxB1aa (HoxB1aa)
DH_3323	Salmo salar	QNTIRTNFTTKQLTELEKEFHFSKYLTRARRVEIAATLELNETQVKIWFQNRRMKQKKRE	Homeobox protein HoxB1ab (Homeobox protien HoxB1ab) (HoxB1ab)
DH_460	Gallus gallus	PNTIRTNFTTKQLTELEKEFHFNKYLTRARRVEIAATLELNETQVKIWFQNRRMKQKKRE	Homeobox protein Hox-B1 (HOXB1)
DH_4560	Gallus gallus	PNTIRTNFTTKQLTELEKEFHFNKYLTRARRVEIAATLELNETQ----------------	Homeobox-containing transcription factor Hoxb-1 (Fragment) (Hoxb-1)
DH_8019	Latimeria menadoensis	QNTIRTNFTTKQLTELEKEFHFNKYLTRARRVEIAATLELNETQVKIWFQNRRMKQKKRE	HoxB1 (Fragment)
DH_470	Fugu rubripes	NSAIRTNFSTRQLTELEKEFHFSKYLTRARRVEIAATLELNETQVKIWFQNRRMKQKKRE	Homeobox protein Hox-B1a (hoxb1a)
DH_7485	Fugu rubripes	NSAIRTNFSTRQLTELEKEFHFSKYLTRARRVEIAATLELNETQVKIWFQNRRMKQKKRE	Hoxb1a
DH_4425	Fugu rubripes	NSAIRTNFSTRQLTELEKEFHFSKYLTRARRVEIAATLELNETQVKIWFQNRRMKQKKRE	Homeobox protein HOXB-1 (FrHOXB-1)
DH_6613	Tetraodon nigroviridis	NSAIRTNFSTRQLTELEKEFHFSKYLTRARRVEIAATLELNETQVKIWFQNRRMKQKKRE	Chromosome undetermined SCAF7711, whole genome shotgun sequence. (Fragment) (GSTENG00005035001)
DH_6002	Oryzias latipes	NSAMRTNFTTRQLTELEKEFHFSKYLTRARRVEIAATLELNETQVKIWFQNRRMKQKKRE	HoxB1a (hoxB1a)
DH_6175	Oryzias latipes	NSAMRTNFTTRQLTELEKEFHFSKYLTRARRVEIAATLELNETQVKIWFQNRRMKQKKRE	HoxB1a (hoxB1a)
DH_2425	Cynolebias luteoflammulatus	--------------EFEKEFHFSKYLTRARRVEIAATLELNETQVKIWFQNR--------	Hox1 (Fragment)
DH_11824	Gasterosteus aculeatus	SNAMRTNFSTRQLTELEKEFHFSKYLTRARRVEIAATLELNETQVKIWFQNRRMKQKKRE	groupXI
DH_6850	Oreochromis niloticus	-----TKFSTRQLTELEKEFHFSKYLTRGPRVEIAATLELKGTQVKIWFQNRSMKQKKRD	Hox protein (Fragment)
DH_459	Ateles geoffroyi	PSGLRTNFTTRQLTELEKEFHFNKYLSRARRVEIAATLELNETQVKIWFQNRRMKQKKRE	Homeobox protein Hox-B1 (HOXB1)
DH_463	Macaca mulatta	PSGLRTNFTTRQLTELEKEFHFNKYLSRARRVEIAATLELNETQVKIWFQNRRMKQKKRE	Homeobox protein Hox-B1 (HOXB1)
DH_464	Macaca nemestrina	PSGLRTNFTTRQLTELEKEFHFNKYLSRARRVEIAATLELNETQVKIWFQNRRMKQKKRE	Homeobox protein Hox-B1 (HOXB1)
DH_466	Pan paniscus	PSGLRTNFTTRQLTELEKEFHFNKYLSRARRVEIAATLELNETQVKIWFQNRRMKQKKRE	Homeobox protein Hox-B1 (HOXB1)
DH_467	Pan troglodytes	PSGLRTNFTTRQLTELEKEFHFNKYLSRARRVEIAATLELNETQVKIWFQNRRMKQKKRE	Homeobox protein Hox-B1 (HOXB1)
DH_462	Homo sapiens	PSGLRTNFTTRQLTELEKEFHFNKYLSRARRVEIAATLELNETQVKIWFQNRRMKQKKRE	Homeobox protein Hox-B1 (HOXB1)
DH_468	Pongo pygmaeus	PSGLRTNFTTRQLTELEKEFHFNKYLSRARRVEIAATLELNETQVKIWFQNRRMKQKKRE	Homeobox protein Hox-B1 (HOXB1)
DH_9200	Homo sapiens	PSGLRTNFTTRQLTELEKEFHFNKYLSRARRVEIAATLELNETQVKIWFQNRRMKQKKRE	Homeobox B1 (Fragment) (HOXB1)
DH_1358	Gorilla gorilla gorilla	PSGLRTNFTTRQLTELEKEFHFNKYLSRARRVEIAATLELNETQVKIWFQNRRMKQKKRE	HOXB1 (Fragment) (HOXB1)
DH_1494	Lagothrix lagotricha	PSGLRTNFTTRQLTELEKEFHFNKYLSRARRVEIAATLELNETQVKIWFQNRRMKQKKRE	HOXB1 (Fragment) (HOXB1)
DH_465	Mus musculus	PGGLRTNFTTRQLTELEKEFHFNKYLSRARRVEIAATLELNETQVKIWFQNRRMKQKKRE	Homeobox protein Hox-B1 (Hoxb1)
DH_2718	Mus musculus	PGGLRTNFTTRQLTELEKEFHFNKYLSRARRVEIAATLELNETQVKIWFQNRRMKQKKRE	Homeo box B1 (RP23-9G13.5-001)
DH_8768	Notophthalmus viridescens	-----------------KEFHFNKYLTRARRCEIAATLELNETQVKIWFQN---------	Homeoprotein (Fragment) (NvHoxB1)
DH_1641	Carassius auratus	-------------------IHFNSYLTRARRLEVAATLELNETQVKIWFQNRRM------	HoxB1b protein (Fragment) (Hox)
DH_3354	Salmo salar	QNVIRTNFNTKQLTELEKEFHFNRYLTRARRVEVAAILELNETQVKIWFQNRRMKQKKRE	Homeobox protein HoxB1ba (HoxB1ba)
DH_6166	Oryzias latipes	INVIRTNFTTKQLTELEKEFHFNKYLTRARRIEVAASLDLNETQVKIWFQNRRMKQKKRE	HoxB1b (hoxB1b)
DH_9401	Oryzias latipes	--------------------HFNKYLTRARRIEVAASLDLNETQVKIWFQNRRMKQKKRE	HOXB1B (Fragment) (hoxb1b)
DH_3276	Salmo salar	GAIPRTNFTTKQLTELEKEFHFNKYLTRSRRVEIAHGLHLNETQVKIWFQNRRMKQKKRE	Homeobox protein HoxD1aa (HoxD1aa)
DH_8011	Latimeria menadoensis	GATARTNFTTKQLTELEKEFHFNKYLTRARRVEIASALQLNETQVKIWFQNRRMKQKKRE	HoxC1 (Fragment)
DH_608	Homo sapiens	SSAIRTNFSTKQLTELEKEFHFNKYLTRARRIEIANCLHLNDTQVKIWFQNRRMKQKKRE	Homeobox protein Hox-D1 (HOXD1)
DH_2836	Homo sapiens	SSAIRTNFSTKQLTELEKEFHFNKYLTRARRIEIANCLHLNDTQVKIWFQNRRMKQKKRE	cDNA, FLJ94811, Homo sapiens homeo box D1 (HOXD1), mRNA (Homeobox D1) (HOXD1)
DH_9007	Homo sapiens	SSAIRTNFSTKQLTELEKEFHFNKYLTRARRIEIANCLHLNDTQVKIWFQNRRMKQKKRE	Homeobox D1 (HOXD1)
DH_609	Mus musculus	PSAIRTNFSTKQLTELEKEFHFNKYLTRARRIEIANCLQLNDTQVKIWFQNRRMKQKKRE	Homeobox protein Hox-D1 (Hoxd1)
DH_5027	Mus musculus	PSAIRTNFSTKQLTELEKEFHFNKYLTRARRIEIANCLQLNDTQVKIWFQNRRMKQKKRE	Homeo box D1 (Hoxd1)
DH_610	Xenopus laevis	PCNVRTNFTTKQLTELEKEFHFNKYLTRARRIEIANSLQLNDTQVKIWFQNRRMKQKKRE	Homeobox protein Hox-D1 (hoxd1)
DH_5728	Xenopus tropicalis	PCTVRTNFTTKQLTELEKEFHFNKYLTRARRIEIANSLQLNDTQVKIWFQNRRMKQKKRE	Homeo box D1 (hoxd1)
DH_11822	Gasterosteus aculeatus	NGPPRTSFSTKQLTELEKEFHFHKYLTRARRVEVAGALQLSETQVKVWFQNRRMKQKKLQ	groupXII
DH_11528	Haplochromis burtoni	NGAPRISYSTKQLTELEKEFHFNKYLTRARRVEVAGALQLSETQVKVWFQNRRMKEKKLQ	HoxC1a
DH_3251	Salmo salar	NGVLRTSFTTKQLTELEKEFHFNKYLTRARRVEIASALQLSETQVKIWFQNRRMKQKKLM	Homeobox protein HoxC1ab (HoxC1ab)

```
DH_543   Danio rerio                          SGGSRTNFTTKQLTELEKEFHFNKYLTRARRIEIANPLQLSETQVKIWFQNRRMKQKKML Homeobox protein Hox-C1a (hoxc1a)
DH_2909  Danio rerio                          SGGSRTNFTTKQLTELEKEFHFNKYLTRARRIEIANPLQLSETQVKIWFQNRRMKQKKML Homeo box C1a (hoxc1a)
DH_5043  Glomeris marginata                   ---------TKQLTELEKEFHFNKYLTRARRIEIATALQLNETQVKIWFQNRRMKQKKRM Labial protein (Fragment) (lab)
DH_8647  Lithobius atkinsoni                  --------------------HFNKYLTRARRIEIATALQLNETQVKIWFQNRRMKQKKRL Labial (Fragment) (lab)
DH_5630  Polycelis nigra                      NITGRTNFTNKQLTELEKEFHFNQYLTRARRIEIAKSMTLSETQIKIWFQNRRMKQKRRQ Homeodomain protein (Fragment) (Pnox3)
DH_8635  Euprymna scolopes                    NSTGRTNFTNKQLTELEKEFHFNKYLTRARRIEIAAA----------------------- Labial homeodomain protein (Fragment) (Lab)
DH_7862  Anopheles gambiae                    NSTGRTNFTNKQLTELEKEFHFNKYLTRARRIEIANALHLNETQVKIWFQNRRMKQKKRV AGAP004650-PA (Fragment) (AGAP004650)
DH_9247  Anopheles gambiae                    NSTGRTNFTNKQLTELEKEFHFNKYLTRARRIEIANALHLNETQVKIWFQNRRMKQK--- Labial homeotic protein (Fragment) (lab)
DH_5327  Aedes aegypti                        TSTGRTNFTNKQLTELEKEFHFNKYLTRARRIEIANSLHLNETQVKIWFQNRRMKQKKRI Homeobox protein (AAEL005195)
DH_2600  Culex quinquefasciatus               VSTGRTNFTNKQLTELEKEFHFNKYLTRARRIEIANTLQLNETQVKIWFQNRRMKQKKRI Homeobox protein (CpipJ_CPIJ002050)
DH_3570  Drosophila sechellia                 NNSGRTNFTNKQLTELEKEFHFNRYLTRARRIEIANTLQLNETQVKIWFQNRRMKQKKRV GM10536 (GM10536)
DH_4226  Drosophila simulans                  NNSGRTNFTNKQLTELEKEFHFNRYLTRARRIEIANTLQLNETQVKIWFQNRRMKQKKRV GD19533 (GD19533)
DH_687   Drosophila melanogaster              NNSGRTNFTNKQLTELEKEFHFNRYLTRARRIEIANTLQLNETQVKIWFQNRRMKQKKRV Homeotic protein labial (lab)
DH_3166  Drosophila erecta                    NNSGRTNFTNKQLTELEKEFHFNRYLTRARRIEIANTLQLNETQVKIWFQNRRMKQKKRV GG10324 (GG10324)
DH_4149  Drosophila yakuba                    NNSGRTNFTNKQLTELEKEFHFNRYLTRARRIEIANTLQLNETQVKIWFQNRRMKQKKRV GE25839 (GE25839)
DH_3466  Drosophila persimilis                NNSGRTNFTNKQLTELEKEFHFNRYLTRARRIEIANTLQLNETQVKIWFQNRRMKQKKRV GL22053 (GL22053)
DH_4331  Drosophila pseudoobscura pseudoobscura NNSGRTNFTNKQLTELEKEFHFNRYLTRARRIEIANTLQLNETQVKIWFQNRRMKQKKRV Lab (Dpse\lab)
DH_3906  Drosophila virilis                   NNSGRTNFTNKQLTELEKEFHFNRYLTRARRIEIANTLQLNETQVKIWFQNRRMKQKKRV Lab (Dvir\lab)
DH_8948  Drosophila virilis                   NNSGRTNFTNKQLTELEKEFHFNRYLTRARRIEIANTLQLNETQVKIWFQNRRMKQKKRV Homeodomain transcription factor labial (Fragment) (lab)
DH_7702  Drosophila virilis                   NNSGRTNFTNKQLTELEKEFHFNRYLTRARRIEIANTLQLNETQVKIWFQNRRMKQKKRV Homeodomain transcription factor labial (Fragment) (lab)
DH_3687  Drosophila grimshawi                 NNSGRTNFTNKQLTELEKEFHFNRYLTRARRIEIANTLQLNETQVKIWFQNRRMKQKKRV GH19490 (GH19490)
DH_7700  Drosophila buzzatii                  NNSGRTNFTNKQLTELEKEFHFNRYLTRARRIEIANTLQLNETQVKIWFQNRRMKQKKRV Homeodomain transcription factor labial (lab)
DH_7701  Drosophila buzzatii                  NNSGRTNFTNKQLTELEKEFHFNRYLTRARRIEIANTLQLNETQVKIWFQNRRMKQKKRV Homeodomain transcription factor labial (Fragment) (lab)
DH_6661  Drosophila buzzatii                  NNSGRTNFTNKQLTELEKEFHFNRYLTRARRIEIANTLQLNETQVKIWFQNRRMKQKKRV Dbuz\lab-PA (lab)
DH_3767  Drosophila mojavensis                NNSGRTNFTNKQLTELEKEFHFNRYLTRARRIEIANTLQLNETQVKIWFQNRRMKQKKRV GI23760 (GI23760)
DH_8950  Drosophila mercatorum                NNSGRTNFTNKQLTELEKEFHFNRYLTRARRIEIANTLQLNETQVKIWFQNRRMKQKKRV Labial (Fragment) (lab)
DH_8949  Drosophila hydei                     NNSGRTNFTNKQLTELEKEFHFNRYLTRARRIEIANTLQLNETQVKIWFQNRRMKQKKRV Labial (Fragment) (lab)
DH_6664  Drosophila repleta                   NNSGRTNFTNKQLTELEKEFHFNRYLTRARRIEIANTLQLNETQVKIWFQNRRMKQKKRV Labial (Fragment) (lab)
DH_8951  Drosophila repleta                   NNSGRTNFTNKQLTELEKEFHFNRYLTRARRIEIANTLQLNETQVKIWFQNRRMKQKKRV Labial (Fragment) (lab)
DH_4010  Drosophila willistoni                NNSGRTNFTNKQLTELEKEFHFNRYLTRARRIEIANTLQLNETQVKIWFQNRRMKQKKRV GK10836 (GK10836)
DH_8106  Chymomyza amoena                     ------------------EFHFNRYLTRARRIEIANTLQLNETQVRLWFQNRRM------ Lab protein (Fragment) (lab)
DH_8104  Zaprionus vittiger                   ------------------EFHFNRYLTRARRIEIANTLQLNETQVNLWFQHRRM------ Lab protein (Fragment) (lab)
DH_3005  Drosophila ananassae                 NNSGRTNFTNKQLTELEKEFHFNRYLTRARRIEIANTLQLNETQVKIWFQNRRMKQKKRQ GF17803 (GF17803)
DH_12439 Helobdella robusta                   SNLGRTNFTNKQLTELEKEFHFNKYLTRARRIEIASTLGLNETQVKIWFQNRRMKHKKRL e_gw1.18.96.1|Helro1
DH_4498  Helobdella triserialis               NNLGRTNFTNKQLTELEKEFHFNKYLTRARRIEIASTLGLNETQVKIWFQNRRMKHKKRL Lox7 protein (Lox7)
DH_9504  Thermobia domestica                  SNAGRTNFTNKQLTELEKEFHFNKYLTRARRIEIASALQLNETQVKIWFQNRR------- Labial protein (Fragment) (lab)
DH_1323  Metacrinus rotundus                  NNNGRTNFTNKQLTELEKEFHFNKYLTRARRVEIASQLGLNETQVKIWFQNRRMKEKKKM Transcription factor Hox1 (MrHox1)
DH_12609 Strongylocentrotus purpuratus        NNNGRTNFTNKQLTELEKEFHFNKYLTRARRIEIAAMLGLNETQVKIWFQNRRMKEKKKM GLEAN3_17352|Sp-Hox1
DH_8921  Tribolium castaneum                  LNTGRTNFTNKQLTELEKEFHFNKYLTRARRIEIASALQLNETQVKIWFQNRRMKQKKRM Homeodomain transcription factor Labial (labial)
DH_9236  Tribolium castaneum                  LNTGRTNFTNKQLTELEKEFHFNKYLTRARRIEIASALQLNETQVKIWFQNRRMKQKKRM Labial protein (Fragment) (lab)
DH_7494  Oncopeltus fasciatus                 ---GRTNFTNKQLTELEKEFHFNKYLTRARRIEIASALQLNETQVKIWFQNRRMKQKKRM Labial (Fragment)
DH_7776  Bombyx mori                          NNTGRTNFTNKQLTELEKEFHFNKYLTRARRIEIASALQLNETQVKIWFQNRRMKQKKRI Hox 1 (Fragment) (Bmlab)
DH_11446 Daphnia pulex                        NNTGRTNFTTKQLTELEKEFHFNKYLTRARRIEIAAALQLNETQVKIWFQNRRMKQKKRM gw1.7.522.1|Dappu1
DH_11516 Daphnia pulex                        NNTGRTNFTTKQLTELEKEFHFNKYLTRARRIEIAAALQLNETQVKIWFQNRRMKQKKRM YAS_Hox_Lab|Dappu1
DH_2757  Flaccisagitta enflata                NNTGRTNFTTKQLTELEKEFHFNKYLTRARRIEIAGALQLNETQVKIWFQNRRMKQKKRM Hox1 (Fragment)
DH_6016  Sacculina carcini                    NNTGRTNFTTKQLTELEKEFHFNKYLTRARRIEIATALALNETQVKIWFQNRRMKQKKRI Labial
DH_9469  Porcellio scaber                     GGTGRTNFTTKQLTELEKEFHFNKYLTRARRIEIASALQLNETQVKIWFQNRR------- Labial protein (Fragment)
DH_4292  Archegozetes longisetosus            PNTGRTNFTTNQLTELEKEFHFNKYLTRARRIEIATALQLNETQVK-------------- Labial hox protein (Fragment)
DH_8998  Folsomia candida                     GATGRTNFTTKQLTELEKEFHFNKYLTRARRIEIASSLQLNETQ---------------- Labial (Fragment)
DH_2229  Brugia malayi                        TNAIRTNFTTHQLTELEKEYYTSKYLNRTRRAEIASILQLNETQIIEVKVLVVKE----- Homeobox domain containing protein (Bm1_05825)
```

Save alignment [fasta format] - Save complete sequences [fasta format]

PG2

ID	organism	10 20 30 40 50 60	description
DH_2135	Haplochromis burtoni	SRRLRTAYTNTQLLELEKEFHFNKYLCRPRRVEIAALLDLTEKQVKVWFQNRRMKHKRQT	Hoxa2b (hoxa2b)
DH_2457	Oreochromis niloticus	SRRLRTAYTNTQLLELEKEFHFNKYLCRPRRVEIAALLDLTEKQVKVWFQNRRMKHKRQT	Hoxa2b (Fragment)
DH_6725	Oreochromis niloticus	SRRLRTAYTNTQLLELEKEFHFNKYLCRPRRVEIAALLDLTEKQVKVWFQNRRMKHKRQT	Hox protein (Fragment)
DH_385	Fugu rubripes	SRRLRTAYTNTQLLELEKEFHFNKYLCRPRRVEIAALLDLTEKQVKVWFQNRRMKHKRQT	Homeobox protein Hox-A2b (hoxa2b)
DH_4828	Morone saxatilis	SRRLRTAYTNTQLLELEKEFHFNKYLCRPRRVEIAALLDLTEKQVKVWFQNRRMKHKR--	HoxA2b (Fragment)
DH_11841	Gasterosteus aculeatus	SRRLRTAYTNTQLLELEKEFHFNKYLCRPRRVEIASLLDLTEKQVKVWFQNRRMKHKRQN	Homeobox protein Hox-A2 [Source:UniProtKB/Swiss-Prot;Acc:O43364]
DH_6136	Mus musculus	SRRLRTAYTNTQLLELEKEFHFNKYLCRPRRVEIAALLDLTERQVKVWFQNRRMKHKRQT	Putative uncharacterized protein (Fragment) (Hoxa2)
DH_379	Mus musculus	SRRLRTAYTNTQLLELEKEFHFNKYLCRPRRVEIAALLDLTERQVKVWFQNRRMKHKRQT	Homeobox protein Hox-A2 (Hoxa2)
DH_8830	Mus spicilegus	SRRLRTAYTNTQLLELEKEFHFNKYLCRPRRVEIAALLDLTERQVKVWFQNRRMKHKRQT	Homeobox protein (Fragment) (HOX1.11)
DH_8832	Mus musculus	SRRLRTAYTNTQLLELEKEFHFNKYLCRPRRVEIAALLDLTERQVKVWFQNRRMKHKRQT	Homeobox protein (Fragment) (Hoxa2)
DH_8833	Mus musculus	SRRLRTAYTNTQLLELEKEFHFNKYLCRPRRVEIAALLDLTERQVKVWFQNRRMKHKRQT	Homeobox protein (Fragment) (Hoxa2)
DH_8028	Latimeria menadoensis	SRRLRTAYTNTQLLELEKEFHFNKYLCRPRRVEIAALLDLTGRQVRVWFQNRRMKHKRQT	HoxA2 (Fragment)
DH_377	Heterodontus francisci	SRRLRTAYTNTQLLELEKEFHFNKYLCRPRRVEIAALLDLTERQVKVWFQNRRMKHKRQT	Homeobox protein Hox-A2 (HOXA2)
DH_3290	Salmo salar	SRRLRTAYTNTQLLELEKEFHFNKYLCRPRRVEIAALLDLTERQVKVWFQNRRMKHKRQT	Homeobox protein HoxA2aa (HoxA2aa)
DH_4829	Morone saxatilis	SRRLRTAYTNTQLLELEKEFHFNKYLCRPRRVEIAALLDLTERQVKVWFQNRRMKHKRQT	HoxA2a (Fragment)
DH_376	Gallus gallus	SRRLRTAYTNTQLLELEKEFHFNKYLCRPRRVEIAALLDLTERQVKVWFQNRRMKHKRQT	Homeobox protein Hox-A2 (HOXA2)
DH_8835	Mus musculus	SRRLRTAYTNTQLLELEKEFHFNKYLCRPRRVEIAALLDLTERQVKVWFQNRRMKHKRQT	Homeobox protein (Fragment) (Hoxa2)
DH_382	Rattus norvegicus	SRRLRTAYTNTQLLELEKEFHFNKYLCRPRRVEIAALLDLTERQVKVWFQNRRMKHKRQT	Homeobox protein Hox-A2 (Hoxa2)
DH_8834	Mus musculus	SRRLRTAYTNTQLLELEKEFHFNKYLCRPRRVEIAALLDLTERQVKVWFQNRRMKHKRQT	Homeobox protein (Fragment) (Hoxa2)
DH_8837	Mus musculus	SRRLRTAYTNTQLLELEKEFHFNKYLCRPRRVEIAALLDLTERQVKVWFQNRRMKHKRQT	Homeobox protein (Fragment) (Hoxa2)
DH_8836	Mus musculus	SRRLRTAYTNTQLLELEKEFHFNKYLCRPRRVEIAALLDLTERQVKVWFQNRRMKHKRQT	Homeobox protein (Fragment) (Hoxa2)
DH_8831	Mus musculus	SRRLRTAYTNTQLLELEKEFHFNKYLCRPRRVEIAALLDLTERQVKVWFQNRRMKHKRQT	Homeobox protein (Fragment) (Hoxa2)
DH_374	Bos taurus	SRRLRTAYTNTQLLELEKEFHFNKYLCRPRRVEIAALLDLTERQVKVWFQNRRMKHKRQT	Homeobox protein Hox-A2 (HOXA2)
DH_2808	Rhinolophus ferrumequinum	SRRLRTAYTNTQLLELEKEFHFNKYLCRPRRVEIAALLDLTERQVKVWFQNRRMKHKRQT	Homeobox A2 (Predicted) (HOXA2)
DH_3189	Sorex araneus	SRRLRTAYTNTQLLELEKEFHFNKYLCRPRRVEIAALLDLTERQVKVWFQNRRMKHKRQT	Homeobox A2 (Predicted) (HOXA2)
DH_375	Callithrix jacchus	SRRLRTAYTNTQLLELEKEFHFNKYLCRPRRVEIAALLDLTERQVKVWFQNRRMKHKRQT	Homeobox protein Hox-A2 (HOXA2)
DH_2744	Callicebus moloch	SRRLRTAYTNTQLLELEKEFHFNKYLCRPRRVEIAALLDLTERQVKVWFQNRRMKHKRQT	Homeobox A2 (Predicted) (HOXA2)
DH_381	Papio anubis	SRRLRTAYTNTQLLELEKEFHFNKYLCRPRRVEIAALLDLTERQVKVWFQNRRMKHKRQT	Homeobox protein Hox-A2 (HOXA2)
DH_378	Homo sapiens	SRRLRTAYTNTQLLELEKEFHFNKYLCRPRRVEIAALLDLTERQVKVWFQNRRMKHKRQT	Homeobox protein Hox-A2 (HOXA2)
DH_2847	Homo sapiens	SRRLRTAYTNTQLLELEKEFHFNKYLCRPRRVEIAALLDLTERQVKVWFQNRRMKHKRQT	Homeobox A2 (HOXA2)
DH_380	Notophthalmus viridescens	SRRLRTAYTNTQLLELEKEFHFNKYLCRPRRVEIAALLDLTERQVKVWFQNRRMKHKRQT	Homeobox protein Hox-A2 (Fragment)
DH_9105	Xenopus laevis	SRRLRTAYTNTQLLELEKEFHFNKYLCRPRRVEIAALLDLTERQVKVWFQNRRMKHKRQT	Transcription factor Hoxa2b (hoxa2)
DH_9106	Xenopus laevis	SRRLRTAYTNTQLLELEKEFHFNKYLCRPRRVEIAALLDLTERQVKVWFQNRRMKHKRQT	Transcription factor Hoxa2 (Hoxa2 protein) (Hoxa2)
DH_2155	Haplochromis burtoni	SRRLRTAYTNTQLLELEKEFHFNKYLCRPRRVEIAALLDLTERQVKVWFQNRRMKHKRQT	Hoxa2a (hoxa2a)
DH_2456	Oreochromis niloticus	SRRLRTAYTNTQLLELEKEFHFNKYLCRPRRVEIAALLDLTERQVKVWFQNRRMKHKRQT	Hoxa2a (Fragment)
DH_6189	Oryzias latipes	SRRLRTAYTNTQLLELEKEFHFNKYLCRPRRVEIAALLDLTERQVKVWFQNRRMKHKRQT	HoxA2a (hoxA2a)
DH_3336	Salmo salar	SRRLRTAYTNTQLLELEKEFHFNKYLCRPRRVEIAALLDLTERQVKVWFQNRRMKHKRQT	Homeobox protein HoxA2aa (HoxA2aa)
DH_3300	Salmo salar	SRRLRTAYTNTQLLELEKEFHFNKYLCRPRRVEIAALLDLTERQVKVWFQNRRMKHKRQT	Homeobox protein HoxA2ab (HoxA2ab)
DH_6540	Tetraodon nigroviridis	SRRLRTAYTNTQLLELEKEFHFNKYLCRPRRVEIAALLDLTERQVKVWFQNRRMKHKRQT	Chromosome 21 SCAF14577, whole genome shotgun sequence (GSTENG00017482001)
DH_11805	Gasterosteus aculeatus	SRRLRTAYTNTQLLELEKEFHFNKYLCRPRRVEIAALLDLTERQVKVWFQNRRMKHKRQT	groupXI
DH_8290	Morone saxatilis	SRRLRTAYTNTQLLELEKEFHFNKYLCRPRRVEIAALLDLTERQVKVWFQNRRMKHKRQT	Hoxb2a protein (Hoxb2a)
DH_477	Fugu rubripes	SRRLRTAYTNTQLLELEKEFHFNKYLCRPRRVEIAALLDLTERQVKVWFQNRRMKHKRQT	Homeobox protein Hox-B2a (hoxb2a)
DH_6513	Tetraodon nigroviridis	SRRLRTAYTNTQLLELEKEFHFNKYLCRPRRVEIAALLDLTERQVKVWFQNRRMKHKRQT	Chromosome undetermined SCAF14653, whole genome shotgun sequence. (Fragment) (GSTENG00020451001)
DH_6174	Oryzias latipes	SRRLRTAYTNTQLLELEKEFHFNKYLCRPRRVEIAALLDLTERQVKVWFQNRRMKHKRQT	HoxB2a (hoxB2a)
DH_476	Danio rerio	SRRLRTAYTNTQLLELEKEFHFNKYLCRPRRVEIAALLDLTERQVKVWFQNRRMKHKRQT	Homeobox protein Hox-B2a (hoxb2a)
DH_1534	Megalobrama amblycephala	SRRLRTAYTNTQLLELEKEFHFNKYLCRPRRVEIAALLDLTERQVKVWFQNRRMKHKRQT	Homeodomain protein (Fragment) (HoxB2a)
DH_3313	Salmo salar	SRRLRTAYTNTQLLELEKEFHFNKYLCRPRRVEIAALLDLTERQVKVWFQNRRMKHKRQT	Homeobox protein HoxB2aa (Homeobox protien HoxB2ab) (HoxB2aa)
DH_3322	Salmo salar	SRRLRTAYTNTQLLELEKEFHFNKYLCRPRRVEIAALLDLTERQVKVWFQNRRMKHKRQT	Homeobox protein HoxB2ab (Homeobox protein HoxB2ab2) (HoxB2ab)
DH_11763	Gasterosteus aculeatus	SRRLRTAYTNTQLLELEKEFHFNKYLCRPRRVEIAALLDLTERQVKVWFQNRRMKHKRQT	Homeobox protein Hox-A2 [Source:UniProtKB/Swiss-Prot;Acc:O43364]
DH_383	Fugu rubripes	SRRLRTAYTNTQLLELEKEFHFNKYLCRPRRVEIAALLDLTERQVKVWFQNRRMKHKRQT	Homeobox protein Hox-A2a (hoxa2a)
DH_8838	Mus musculus	SRRLRTAYTNTQLLELEKEFHFNKYLCRPRRVEIAALLDLTERQVKVWFQNRRMKHKRQT	Homeobox protein (Fragment) (Hoxa2)
DH_3349	Salmo salar	SRRLRTAYTNTQLLELEKEFHFNKYLCRPRRVEIAALLDLTERQVKVWFQNRRMKHKRQT	Homeobox protein HoxB2aa (HoxB2aa)
DH_8018	Latimeria menadoensis	SRRLRTAYTNTQLLELEKEFHFNKYLCRPRRVEIAALLDLTERQVKVWFQNRRMKHKRQT	HoxB2 (Fragment)
DH_7289	Mus musculus	SRRLRTAYTNTQLLELEKEFHFNKYLCRPRRVEIAALLDLTERQVKVWFQNRRMKHKRQT	Homeobox protein gene Hox-2.8, partial cds. (Fragment) (Hoxb2)
DH_474	Mus musculus	SRRLRTAYTNTQLLELEKEFHFNKYLCRPRRVEIAALLDLTERQVKVWFQNRRMKHKRQT	Homeobox protein Hox-B2 (Hoxb2)
DH_3305	Salmo salar	SRRLRTAYTNTQLLELEKEFHFNKYLCRPRRVEIAALLNLTERQVKVWFQNRRMKHKRQT	Homeobox protein HoxA2b (HoxA2b)
DH_6794	Oncorhynchus mykiss	SRRLRTAYTNTQLLELEKEFHFNKYLCRPRRVEIAALLNLTERQVKVWFQNRRMKHKRQT	HoxA2bi (Fragment) (HoxA2bi)
DH_5381	Endeis spinosa	PRRLRTAYTNTQLLELEKEFHFNKYLCRPRRIEIAASLDLTERQVKVWFQNRRMRHKRQS	Homeobox protein proboscipedia (Fragment) (Pb)
DH_5001	Platynereis dumerilii	PRRLRTAYTNTQLLELEKEFHFNKYLCRPRRIEIAASL----------------------	Hox2 homeobox protein (Fragment) (Hox2)
DH_9503	Thermobia domestica	PRRLRTAYTNTQLLELEKEFHFNKYLCRPRRIEIAASLDLTERQVKV-------------	Proboscipedia protein (Fragment) (pb)
DH_9464	Chaetopterus variopedatus	PRRLRTAYTNTQLLELEKEFHFNKYLCRPRRIEIAASLDLTERQVKVWFQNRRMKFKRQS	Homeoprotein CH-Hox2 (Hox2)

```
DH_9182  Oncopeltus fasciatus                     PRRLRTAYTNTQLLELEKEFHFNKYLCRPRRIEIAASLDLTERQVKVWFQ---------- Proboscipedia (Fragment)
DH_12107 Capitella sp. I Grassle and Grassle, 1976 PRRLRTAYTNTQLLELEKEFHFNKYLCRPRRIEIAASLDLTERQVKVWFQNRRMKFKRQ- e_gw1.70.61.1|Capca1
DH_9468  Porcellio scaber                         PRRLRTAYTNTQLLELEKEFHFNKXLCRPRRIXXAASLDLTERQVKVWF----------- Proboscipedia protein (Fragment)
DH_9485  Nereis virens                            PRRLRTAYTNTQLLELEKEFHFNKYLCRPRRIEIAASLDLTERQVKVWFQNRRMKFKRQT Probiscipedia homeobox protein (Fragment) (pb)
DH_9246  Anopheles gambiae                        PRRLRTAYTNTQLLELEKEFHFNKYLCRPRRIEIAASLDLTERQVKVWFQNRR------- Transcription factor proboscipedia (Fragment)
DH_11517 Daphnia pulex                            PRRLRTAYTNTQLLELEKEFHFNKYLCRPRRIEIAASLDLTERQVKVWFQNRRMKHKRQA YAS_NCBI_GNO_0700045|Dappu1
DH_9293  Achaearanea tepidariorum                 PRRLRTAYTNTQLLELEKEFHFNKYLCRPXRIEIAASLDLTXRQVKVWFQNRR------- Proboscipedia (Fragment)
DH_2467  Nymphon gracile                          PRRLRTAYTNNQLLELEKEFHFNKYLCRPRRIEIAASLELTERQVKVWFQNRRMKHKRQS Homeobox protein proboscipedia (Fragment) (Pb)
DH_8935  Artemia sanfranciscana                   ---------NSQLLELEKEFHFNKYLCRPRRIEIATSLSLTERQVKV------------- Proboscipedia (Fragment)
DH_4608  Archegozetes longisetosus                PRRLRTAYTNTQLLELEKEFHFNKYLCRPRRIEIAASLDLTERQVKVWFQNRRMKHKRQS Proboscipedia (Fragment)
DH_6665  Drosophila buzzatii                      PRRLRTAYTNTQLLELEKEFHFNKYLCRPRRIEIAASLDLTERQVKVWSQNRRMK----- Proboscipedia (Fragment) (pb)
DH_11452 Daphnia pulex                            PRRLRTAYTNTQLLELEKEFHFNKYLCRPRRIEIAASLDLTERQVKVWFQNRRMKHKRQ- e_gw1.7.143.1|Dappu1
DH_8922  Tribolium castaneum                      PRRLRTAYTNTQLLELEKEFHFNKYLCRPRRIEIAASLDLTERQVKVWFQNRRMKHKRQT Homeodomain transcription factor Maxillopedia (Fragment) (mxp)
DH_9458  Tribolium castaneum                      PRRLRTAYTNTQLLELEKEFHFNKYLCRPRRIEIAASLDLTERQVKVWFQNRRMKHKRQT Proboscipedia ortholog (Fragment) (maxillopedia)
DH_3571  Drosophila sechellia                     PRRLRTAYTNTQLLELEKEFHFNKYLCRPRRIEIAASLDLTERQVKVWFQNRRMKHKRQT GM10530 (GM10530)
DH_4228  Drosophila simulans                      PRRLRTAYTNTQLLELEKEFHFNKYLCRPRRIEIAASLDLTERQVKVWFQNRRMKHKRQT GD19525 (GD19525)
DH_305   Drosophila melanogaster                  PRRLRTAYTNTQLLELEKEFHFNKYLCRPRRIEIAASLDLTERQVKVWFQNRRMKHKRQT Homeotic protein proboscipedia (pb)
DH_4148  Drosophila yakuba                        PRRLRTAYTNTQLLELEKEFHFNKYLCRPRRIEIAASLDLTERQVKVWFQNRRMKHKRQT GE25834 (GE25834)
DH_3167  Drosophila erecta                        PRRLRTAYTNTQLLELEKEFHFNKYLCRPRRIEIAASLDLTERQVKVWFQNRRMKHKRQT GG10269 (GG10269)
DH_3004  Drosophila ananassae                     PRRLRTAYTNTQLLELEKEFHFNKYLCRPRRIEIAASLDLTERQVKVWFQNRRMKHKRQT GF17798 (GF17798)
DH_3465  Drosophila persimilis                    PRRLRTAYTNTQLLELEKEFHFNKYLCRPRRIEIAASLDLTERQVKVWFQNRRMKHKRQT GL22046 (GL22046)
DH_4330  Drosophila pseudoobscura pseudoobscura   PRRLRTAYTNTQLLELEKEFHFNKYLCRPRRIEIAASLDLTERQVKVWFQNRRMKHKRQT Pb (Dpse\pb)
DH_3685  Drosophila grimshawi                     PRRLRTAYTNTQLLELEKEFHFNKYLCRPRRIEIAASLDLTERQVKVWFQNRRMKHKRQT GH19484 (GH19484)
DH_3905  Drosophila virilis                       PRRLRTAYTNTQLLELEKEFHFNKYLCRPRRIEIAASLDLTERQVKVWFQNRRMKHKRQT GJ23937 (GJ23937)
DH_6658  Drosophila buzzatii                      PRRLRTAYTNTQLLELEKEFHFNKYLCRPRRIEIAASLDLTERQVKVWFQNRRMKHKRQT Dbuz\pb-PB (pb)
DH_6660  Drosophila buzzatii                      PRRLRTAYTNTQLLELEKEFHFNKYLCRPRRIEIAASLDLTERQVKVWFQNRRMKHKRQT Dbuz\pb-PD (pb)
DH_6657  Drosophila buzzatii                      PRRLRTAYTNTQLLELEKEFHFNKYLCRPRRIEIAASLDLTERQVKVWFQNRRMKHKRQT Dbuz\pb-PA (pb)
DH_6659  Drosophila buzzatii                      PRRLRTAYTNTQLLELEKEFHFNKYLCRPRRIEIAASLDLTERQVKVWFQNRRMKHKRQT Dbuz\pb-PC (pb)
DH_3752  Drosophila mojavensis                    PRRLRTAYTNTQLLELEKEFHFNKYLCRPRRIEIAASLDLTERQVKVWFQNRRMKHKRQT GI24721 (GI24721)
DH_4011  Drosophila willistoni                    PRRLRTAYTNTQLLELEKEFHFNKYLCRPRRIEIAASLDLTERQVKVWFQNRRMKHKRQT GK10831 (GK10831)
DH_1699  Cupiennius salei                         PRRLRTAYTNTQLLELEKEFHFNKYLCRPRRIEIAASLDLTERQVKVWFQNRRMKHKRQT Proboscipedia (Fragment) (pb)
DH_7499  Oncopeltus fasciatus                     PRRLRTAYTNTQLLELEKEFHFNKYLCRPRRIEIAASLDLTERQVKVWFQNRRMKHKRQT Proboscipedia (Fragment)
DH_2097  Anopheles gambiae                        PRRLRTAYTNTQLLELEKEFHFNKYLCRPRRIEIAASLDLTERQVKVWFQNRRMKHKRQT AGAP004648-PA (AGAP004648)
DH_2583  Culex quinquefasciatus                   PRRLRTAYTNTQLLELEKEFHFNKYLCRPRRIEIAASLDLTERQVKVWFQNRRMKHKRQT Putative uncharacterized protein (CpipJ_CPIJ001002)
DH_5236  Aedes aegypti                            PRRLRTAYTNTQLLELEKEFHFNKYLCRPRRIEIAASLDLTERQVKVWFQNRRMKHKRQT Putative uncharacterized protein (Fragment) (AAEL015111)
DH_8988  Lithobius forficatus                     ------------NLELEKEFHFNKYLCRPRRIEIAVSLDLTERQVKVWFQNRRMKHKRQT Proboscipedia 1 (Fragment) (Pb1)
DH_5042  Glomeris marginata                       --------------------HFNKYLCRPRRIEIAASLDLTERQVKVWFQNRRMKHKRQT Proboscipedia protein (Fragment) (pb)
DH_8646  Lithobius atkinsoni                      ------------LLELEKEFHFNKYLCRPRRIEIAASLDLAERQVKVWFQNRRMKHKRQT Proboscipedia (Fragment)
DH_7258  Ilyanassa obsoleta                       -----------QLEELEKEFHFNKYLCRPRRIEIAASLDLTERQVKVWFQNRRA------ Proboscipedia-like protein (Fragment)
DH_8997  Folsomia candida                         ---------------LEKEFHFNKYLCRPRRIEIAASLDLTERQVKVWFQNR-------- Proboscipedia (Fragment)
DH_12207 Lottia gigantea                          SRRLRTAYTNTQLLELEKEFHFNKYLCRPRRIEIAASLDLTERQVKVWFQNRRMKYKRQS e_gw1.12.69.1|Lotgi1
DH_12664 Strongylocentrotus purpuratus            GRRIRTAFTTTQLLELEQEFRLNHYLCRPRRIQIAAYLELSERQVKIWFQNRRMKQRRLE GLEAN3_12252|Sp-Hox2
DH_1324  Metacrinus rotundus                      PRRARTAFTTTQLLELEKEFHYNKYLCRPRRIEIAAMLELTERQVKVWFQNRRMKQKRVA Transcription factor Hox2 (MrHox2)
DH_9513  Branchiostoma floridae                   SRRLRTVFTNTQLLELEKEFHYNKYVCKPRRKEIASYLDLNERQVKIWFQNRRMRQKRRD AmphiHox2 protein (AmphiHox2)
DH_12678 Branchiostoma floridae                   SRRLRTVFTNTQLLELEKEFHYNKYVCKPRRKEIASYLDLNERQVKIWFQNRRMRQKRRD AmphiHox2
DH_5289  Branchiostoma floridae                   SRRLRTVFTNTQLLELEKEFHYNKYVCKPRRKEIASYLDLNERQVKIWFQNRRMRQKRRD Hox-2 homeodomain protein (Fragment)
DH_11583 Branchiostoma floridae                   SRRLRTVFTNTQLLELEKEFHYNKYVCKPRRKEIASYLDLNERQVKIWFQNRRMRQKRRD e_gw.402.17.1|Brafl1
DH_1261  Saccoglossus kowalevskii                 HRRVRTAFTNTQLLELEKEFHYNKYLCRPRRIEIASMLDLSERQVKVWFQNRRMKHKRQI Hox 2
DH_6279  Ciona intestinalis                       SRRLRTAYTNTQLLELEKEFHYNKYLCRPRRIEIATLLDLTERQVKVWFQNRRMKHKRQQ Transcription factor protein (Fragment) (Ci-Hox2)
DH_8360  Ciona intestinalis                       SRRLRTAYTNTQLLELEKEFHYNKYLCRPRRIEIATRLDLTERQVKVWFQNRRMKHKRQQ Putative homeobox protein hox2 (Fragment) (hox2)
DH_6677  Oikopleura dioica                        SRRLRTAYTNTQLIELEKEFHFNKYLCRPRRIEIASMLDLTERQVKVWFQNRRMKYKREQ Hox2 (Fragment) (Hox2)
DH_1299  Haliotis asinina                         SRRLRTAYTNTQLLKLEKEFHFNKYLCRP------------------------------- Hox2 (Fragment)
DH_7655  Perionyx excavatus                       PRRLRTAYTNTQLLELEKEFHFNKYLCRPRRIEIAATLNLTERQVKIWFQNRR------- Homeobox protein pb (Fragment)
DH_2458  Oreochromis niloticus                    SRRLRTAYTNTQLLELEKEFHFNKYLCRPRRVEIAALLDLTERQVKVWFQNRRMKHKRQN Hoxb2a (Fragment)
DH_6722  Oreochromis niloticus                    SRRLRTAYTNTQLLELEKEFHFNKYLCRPRRVEIAALLDLTERQVKVWFQNRRMKHKRQN Hox protein (Fragment)
DH_7775  Bombyx mori                              -----TAYTNTQLLELEKEFHFNKYLCRPRRVEIAALLDLTERQVKVWFQNRRMKHKRQT Hox 2 (Fragment) (Bmhox2)
DH_475   Salmo salar                              PGRLRTAYTNTQLLELEKEFHFNKYLCRPRRVEIAALLDLTERQVKLWFQNRRMKHKRQT Homeobox protein Hox-B2 (Fragment) (hoxb2)
DH_7502  Lampetra japonica                        SKRLRTAYTNTQLLELEKEFHFNKYLCRPRRVEIAALLDLTERQVKVWFQNRRMKHKRQT Transcription factor Hox2 (Hox2)
DH_8510  Petromyzon marinus                       SKRLRTAYANTQLLELEKEFHFNKYLCRPRRVEIAALLDLTERQVKVWFQNRRMKHKRQT HoxE2 homeobox (Fragment)
DH_384   Danio rerio                              TRRLRTAYTNTQLLELEKEFHFNKYLCRPRRVEIAALLDLTERQVKVWFQNRRMKHKRQT Homeobox protein Hox-A2b (hoxa2b)
DH_2897  Danio rerio                              TRRLRTAYTNTQLLELEKEFHFNKYLCRPRRVEIAALLDLTERQVKVWFQNRRMKHKRQT Homeo box A2b (hoxa2b)
DH_2895  Danio rerio                              TRRLRTAYTNTQLLELEKEFHFNKYLCRPRRVEIAALLDLTERQVKVWFQNRRMKHKRQT Homeo box A2b (hoxa2b)
DH_2122  Penaeus vannamei                         ------------ALEFEKEFHFNKYLCRPRRVEIAALLNLTERQVKVWFQNRRDP----- Proboscipedia (Fragment)
DH_4658  Echinococcus granulosus                  --------------ELEKEFHFNKYLCRPRWVEIAALLDLTERRVKVWFQNR-------- Proboscipedia-like protein (Fragment) (Hox2)
DH_1524  Megalobrama amblycephala                 TRRLRTAYTNTQLLELEKEFHFNKYLCRPRRVEIAALLDLTERQVKVWFQNRRMKRKRQT Homeodomain protein (Fragment) (HoxA2b)
DH_473   Homo sapiens                             ARRLRTAYTNTQLLELEKEFHFNKYLCRPRRVEIAALLDLTERQVKVWFQNRRMKHKRQT Homeobox protein Hox-B2 (HOXB2)
DH_5512  Rattus norvegicus                        ARRLRTAYTNTQLLELEKEFHFNKYLCRPRRVEIAALLDLTERQVKVWFQNRRMKHKRQT Androgen-dependent epididymal homeobox-like protein (Hoxb2)
DH_5967  Xenopus laevis                           CRRLRTAYTNTQLLELEKEFHFNKYLCRPRRVEIAALLDLTERQVKVWFQNRRMKHKRQT Transcription factor Hoxb2 (Fragment) (Hoxb2)
DH_6390  Tetraodon nigroviridis                   ----RTAYTNTQLLELEKEFHFNKYLCRPRRVEIAALLDLTEKQVKVWFQNRRMKHKRQT Chromosome 8 SCAF15044, whole genome shotgun sequence. (Fragment) (GSTENG00034116001)
DH_1841  Fundulus heteroclitus                    SKRLRTAYTNTQLLELEKEFHFNKYLCRPRRVEIAALLDLTEKQVKVWFQNRRMKHKRQS Homeobox a2x (Hoxa2x)
DH_5408  Fundulus heteroclitus                    SKRLRTAYTNTQLLELEKEFHFNKYLCRPRRVEIAALLDLTEKQVKVWFQNRRMKHKRQS Homeobox A2x (Hoxa2x)
DH_1842  Fundulus majalis                         SKRLRTAYTNTQLLELEKEFHFNKYLCRPRRVEIAALLDLTEKQVKVWFQNRRMKHKRQS Homeobox a2x (Hoxa2x)
DH_5407  Fundulus majalis                         SKRLRTAYTNTQLLELEKEFHFNKYLCRPRRVEIAALLDLTEKQVKVWFQNRRMKHKRQS Homeobox A2x (Hoxa2x)
DH_9399  Oryzias latipes                          --------------------HFNKYLCRPRRVEIAALLDLTERQVKVWFQNRRMKHKRQT HOX2A (Fragment) (HOX2A)
```

```
DH_5195 Homo sapiens        GPQAAHGLHQHAAAGTGEGFTFNKYLCRPRRVEIAALLDLTERQVKVWFQNRRMKHKRQT HOX2.8 protein (Fragment) (HOX2.8)
DH_9398 Oryzias latipes     --------------------HFNKYLCRPRRVEIAALLDLSEKQVKVWFQNRRMKHKRQS HOXA2B (Fragment) (hoxa2b)
DH_6180 Oryzias latipes     SKRLRTAYTNNQLLELEKEFHFNKYLCRPRRVEIAALLDLSEKQVKVWFQNRRMKHKRQS HoxA2b (hoxA2b)
```

Save alignment [fasta format] - Save complete sequences [fasta format]

PG3

ID	organism	10 20 30 40 50 60	description
DH_2003	Nematostella vectensis	TKRYRTSYTNRQLLELEKEFHYNKYLCGTRRRELANAMKLTERQVKVWFQNRRMKLKKDE	Predicted protein (v1g205636)
DH_9160	Nematostella vectensis	TKRYRTSYTNRQLLELEKEFHYNKYLCGTRRRELANAMKLTERQVKVWFQNRRMKLKKDE	Hox type homeodomain protein (Fragment) (anthox7)
DH_5096	Nematostella vectensis	TKRYRTSYTNRQLLELEKEFHYNKYLCGTRRRELANAMKLTERQVKVWFQNRRMKLKKDE	Anthox7-ANTP class homeobox protein (Fragment)
DH_2004	Nematostella vectensis	SKRHRTSYTNKQLLELEKEFHFNKYLCSSRRREISKALQLTERQVKI-------------	Predicted protein (Fragment) (v1g101730)
DH_9159	Nematostella vectensis	SKRHRTSYTNKQLLELEKEFHFNKYLCSSRRREISKALQLTERQVKI-------------	Hox type homeodomain protein (Fragment) (anthox8)
DH_4983	Nematostella vectensis	SKRHRTSYTNKQLLELEKEFHFNKYLCSSRRREISKALQLTERQVKIWFQNRRMKWKKDE	HOXDb (HOXDa) (Fragment)
DH_5055	Nematostella vectensis	SKRHRTSYTNKQLLELEKEFHFNKYLCSSRRREISKALQLTERQVKIWFQNRRMKWKKDE	Anthox8b-ANTP class homeobox protein (Fragment)
DH_5108	Nematostella vectensis	SKRHRTSYTNKQLLELEKEFHFNKYLCSSRRREISKALQLTERQVKIWFQNRRMKWKKDE	Anthox8a-ANTP class homeobox protein (Fragment)
DH_2005	Nematostella vectensis	SKRHRTSYTNKQLLELEKEFHFNKYLCSSRRREISKALQLTERQVKIWFQNRRMKWKKDE	Predicted protein (Fragment) (v1g101611)
DH_12498	Helobdella robusta	SKRPRTAFTSAQLIELEKEFHYNKYLTRPRRVEIANALQLTEKQVKIWFQNRRMKAKKNR	e_gw1.23.267.1|Helro1
DH_1975	Nematostella vectensis	SDKNRTIYSTRQLVELEKEFHYNRYLCRPRRIEIAQSLELTEKQVKIWFQNRRMKWKKEN	Predicted protein (Fragment) (v1g90956)
DH_4985	Nematostella vectensis	SDKNRTIYSTRQLVELEKEFHYNRYLCRPRRIEIAQSLELTEKQVKIWFQNRRMKWKKEN	HOXB (Fragment)
DH_5086	Nematostella vectensis	SDKNRTIYSTRQLVELEKEFHYNRYLCRPRRIEIAQSLELTEKQVKIWFQNRRMKWKKEN	NVHD060-ANTP class homeobox protein (Fragment)
DH_1291	Anopheles gambiae	TKRSRTAFTSSQLVELEKEFHSNRYLCRPRRIELTRKLALTERQIKIWFQNRRMKHKKES	AGAP004647-PA (Fragment) (AGAP004647)
DH_5242	Aedes aegypti	TKRSRTAFTSQQLVELEKEFRLNRYLCRPRRIEIATKLALTERQIKIWFQNRRMKHKKDI	Putative uncharacterized protein (AAEL014277)
DH_8648	Lithobius atkinsoni	---------SAQLVELEKEFHFNRYLCRPRRVELAKQLGLTDRQIKIWFQNRRMKYKKEL	Hox3 (Fragment) (Hox3)
DH_2159	Haplochromis burtoni	SKRTRTAYTSAQLVELEKEFHFSRYLCRPRRVEMAGLLNLHERQIKIWFQNRRMKQKKDQ	Hoxb3b (hoxb3b)
DH_6716	Oreochromis niloticus	SKRTRTAYTSAQLVELEKEFHFSRYLCRPRRVEMAGLLNLHERQIKIWFQNRRMKQKKDQ	Hox protein (Fragment)
DH_11743	Gasterosteus aculeatus	SKRTRTAYTSAQLVELEKEFHFSRYLCKQRRVEMAGLLNLHERQIKIWFQNRRMKQKKDE	groupV
DH_5404	Fugu rubripes	SKRTRTAYTSAQLVELEKEFHFSRYLCRPRRVEMASLLNLQERQIKIWFQNRRMKQKKDE	Homeobox protein HoxB3b (HoxB3b)
DH_7712	Sphoeroides nephelus	SKRTRTAYTSAQLVELEKEFHFSRYLCRPRRVEMASLLNLQERQIKIWFQNRRMKQKKDE	HoxB3b (Fragment) (Hoxb3b)
DH_6526	Tetraodon nigroviridis	SKRTRTAYTSAQLVELEKEFHFSRYLCRPRRVEMAGLLNLQERQIKIWFPHRRLKPKKGE	Chromosome 2 SCAF14604, whole genome shotgun sequence (GSTENG00019144001)
DH_6165	Oryzias latipes	SKRTRTAYTSAQLVELEKEFHFSRYLCRPRRVEMASLLNLNERQIKIWFQNRRMKQKKDQ	HoxB3b (hoxB3b)
DH_7007	Urechis unicinctus	-------------LELEKEFHFNRYLCRPRRIEMAALLNLTERQIKIWFQNRR-------	Homeobox protein Hox3 (Fragment)
DH_8133	Urechis caupo	-----------------KEFHFNRYLCRPRRIEMAALLNLTERQIKIWF-----------	Hox-type homeodomain-containing protein (Fragment)
DH_7190	Thermobia domestica	---------SAQLVELEKEFHFNRYLCRPRRIEMAALLNLTERQIKIWFQNRRMKYKKEQ	Homeodomain transcription factor Hox3/zen (Fragment) (Hox3/zen)
DH_1672	Thermobia domestica	AKRARTAYTSAQLVELEKEFHFNRYLCRPRRIEMAALLNLTERQIKIWFQNRRMKYKKEQ	Homeodomain transcription factor Hox3 (Fragment)
DH_9463	Chaetopterus variopedatus	SKRARTAYTSAQLVELEKEFHFNRYLCRPRRIEMAALLNLTERQIKIWFQNRRMKYKKEQ	Homeoprotein CH-Hox3 (Hox3)
DH_11536	Branchiostoma floridae	GKRARTAYTSAQLVELEKEFHFNRYLCRPRRVEMAAMLNLTERQIKIWFQNRRMKYKKEQ	fgenesh2_pg.scaffold_402000006|Brafl1
DH_350	Branchiostoma floridae	GKRARTAYTSAQLVELEKEFHFNRYLCRPRRVEMAAMLNLTERQIKIWFQNRRMKYKKEQ	Homeobox protein HOX3
DH_12679	Branchiostoma floridae	GKRARTAYTSAQLVELEKEFHFNRYLCRPRRVEMAAMLNLTERQIKIWFQNRRMKYKKEQ	AmphiHox3
DH_9633	Priapulus caudatus	----------AQLVELEKEFHFNRYLCRPRRIEMAAMLNLTERQIKI-------------	Hox 3 homeodomain protein (Fragment)
DH_1673	Folsomia candida	TKRARTAYTSAQLVELEKEFHYNRYLCRPRRIEMASLLSLTERQIKIWFQNRRMKYKKEL	Homeodomain transcription factor Hox3
DH_8996	Folsomia candida	TKRARTAYTSAQLVELEKEFHYNRYLCRPRRIEMASLLSLTERQIKIWFQNRRMKYKKEL	Hox3 (Fragment)
DH_9291	Achaearanea tepidariorum	-FRARTAYTSAQLVELEKEFHFNRYLCRPRRIEMASLLNLTERQIKIWFQNRR-------	Hox-3 (Fragment) (hox-3)
DH_7695	Euprymna scolopes	AKRARTAYTSAQLVELEKEFHFNQYLCRPRRIEMAALLNLSERQIKIWFQNRRMRFKKEK	Hox class homeodomain-containing protein Hox3 (Fragment)
DH_8633	Euprymna scolopes	AKRARTAYTSAQLVELEKEFHFNQYLCRPRRIEMAALLNLSERQIKIWFQNRRMRFKKEK	HOX3 homeodomain protein (Fragment) (Hox3)
DH_12162	Lottia gigantea	AKRARTAYTSAQLVELEKEFHFNRYLCRPRRIEMAALLNLSERQ----------------	gw1.12.35.1|Lotgi1
DH_9494	Lingula unguis	TKRARTAYTSAQLVELEKEFHFNRYLCRPRRIEMAALLNLTERQIKIWFQNRRMKHAKRN	Hox3 homeodomain protein (Fragment)
DH_9046	Haliotis asinina	TKRARTAYTSAQLVELEKEFHFNRYLCRPRRIEMAALLNLTERQ----------------	Hox3 (Fragment) (Hox3)
DH_11383	Daphnia pulex	AKRARTAYTSAQLVELEKEFHFNRYLCRPRRIEMATLLNLTERQIKIWFQNRRMKFKKEQ	YAS_fgenesh1_pg.C_scaffold_7000048|Dappu1
DH_11470	Daphnia pulex	AKRARTAYTSAQLVELEKEFHFNRYLCRPRRIEMATLLNLTERQIKIWFQNRRMKFKKEQ	gw1.7.157.1|Dappu1
DH_4544	Cupiennius salei	AKRARTAYTSAQLVELEKEFHFNRYLCRPRRIEMANLLNLSERQIKIWFQNRRMKYKKEQ	Hox3 protein (hox3)
DH_9484	Nereis virens	SKRARTAYNSAQLVELEKEFHFNRYLCRPRRIEMAALLSLSERQIKIWFQNRRMKYKKDQ	Hox3 homeobox protein (Fragment) (Hox3)
DH_4548	Lineus sanguineus	PKRSRTAYTSAQLVELEKEFHFNRYLCRPRRIEMAALLNLSERQIKIWFQNRRMKYKKDQ	Homeodomain protein (Fragment) (Hox-3)
DH_12081	Capitella sp. I Grassle and Grassle, 1976	SKRARTAYTSAQLVELEKEFHFNRYLCRPRRIEMAALLNLTERQIKIWFQNRRMKYKKDQ	estExt_fgenesh1_pg.C_700024|Capca1
DH_8509	Petromyzon marinus	SKRARTAYTSAQLVELEKEFHFNRYLCRPRRVEMANLLNLTERQIKIWFQNRRMKYKKDH	Hox3 homeobox (Fragment)
DH_7520	Lampetra japonica	SKRARTAYTSAQLVELEKEFHFNRYLCRPRRVEMANLLNLTERQIKIWFQNRRMKYKKDH	LjHox3d Homeobox (Fragment) (LjHox3d)
DH_12640	Strongylocentrotus purpuratus	PKRNRTAFTSAQLVELEKEFHFNRYLCRPRRVEMAKSLNLTERQIKIWFQNRRMKYKRDM	GLEAN3_27568|Sp-Hox3
DH_8174	Echinococcus granulosus	--------------EFEKEFHFNRYLCRPRRVEIANLLNLTERQIKIWFQNRR-------	Homeodomain-containing protein Hox3 (Fragment)
DH_2426	Cynolebias bellottii	------------------EFHFNRYLCRPRRVEMANLLNLTERQVKIWFQNR--------	Hox3 (Fragment)
DH_3339	Salmo salar	SKRARTAYTSAQLVELEKEFHFNRYLCRPRRVEMANLLNLTERQIKIWFQNRRMKYKEDQ	Homeobox protein HoxA3ab (HoxA3ab)
DH_6152	Oryzias latipes	SKRVRTAYTSAQLVELEKEFHFNRYLCRPRRVEMANLLNLTERQIKIWFQNRRMKYKKDQ	HoxD3a (hoxD3a)
DH_6706	Oreochromis niloticus	SKRVRTAYTSAQLVELEKEFHFNRYLCRPRRVEMANLLNLTERQIKIWFQNRRMKYKKDQ	Hox protein (Fragment)
DH_11727	Gasterosteus aculeatus	SKRVRTAYTSAQLVELEKEFHFNRYLCRPRRVEMANLLNLTERQIKIWFQNRRMKYKKDQ	Homeobox protein Hox-D3 (Hox-4A) [Source:UniProtKB/Swiss-Prot;Acc:P31249]
DH_616	Fugu rubripes	SKRVRTAYTSAQLVELEKEFHFNRYLCRPRRVEMANLLNLTERQIKIWFQNRRMKYKKDQ	Homeobox protein Hox-D3a (hoxd3a)
DH_1556	Megalobrama amblycephala	SKRVRTAYTSAQLVELEKEFHFNRYLCRPRRVEMANLLNLTERQIKIWFQNRRMKYKKDQ	Homeodomain protein (Fragment) (HoxD3a)
DH_2554	Danio rerio	SKRVRTAYTSAQLVELEKEFHFNRYLCRPRRVEMANLLNLTERQIKIWFQNRRMKYKKDQ	Homeo box D3a (hoxd3a)
DH_3282	Salmo salar	SKRVRTAYTSAQLVELEKEFHFNRYLCRPRRVEMANLLNLTERQIKIWFQNRRMKYKKDQ	Homeobox protein HoxD3ab (HoxD3ab)
DH_3369	Salmo salar	SKRVRTAYTSAQLVELEKEFHFNRYLCRPRRVEMANLLNLTERQIKIWFQNRRMKYKKDQ	Homeobox protein HoxD3ab (HoxD3ab)
DH_3275	Salmo salar	SKRVRTAYTSAQLVELEKEFHFNRYLCRPRRVEMANLLNLTERQIKIWFQNRRMKYKKDQ	Homeobox protein HoxD3aa (HoxD3aa)
DH_1344	Xenopus laevis	SKRVRTAYTSAQLVELEKEFHFNRYLCRPRRVEMANLLNLTERQIKIWFQNRRMKYKKDQ	LOC100036911 protein (hoxd3)
DH_8003	Latimeria menadoensis	SKRVRTAYTSAQLVELEKEFHFNRYLCRPRRVEMANLLNLTERQIKIWFQNRRMKYKKDQ	HoxD3 (Fragment)

DH_612	Mus musculus	SKRVRTAYTSAQLVELEKEFHFNRYLCRPRRVEMANLLNLTERQIKIWFQNRRMKYKKDQ	Homeobox protein Hox-D3 (Hoxd3)
DH_6140	Mus musculus	SKRVRTAYTSAQLVELEKEFHFNRYLCRPRRVEMANLLNLTERQIKIWFQNRRMKYKKDQ	Putative uncharacterized protein (Hoxd3)
DH_611	Homo sapiens	SKRVRTAYTSAQLVELEKEFHFNRYLCRPRRVEMANLLNLTERQIKIWFQNRRMKYKKDQ	Homeobox protein Hox-D3 (HOXD3)
DH_8044	Rattus sp	SKRVRTAYTSAQLVELEKEFHFNRYLCRPRRVEMANLLNLTERQIKIWFQNRRMKYKKDQ	Hox-D|Hox-4 (Fragment) (Hox-D|Hox-4)
DH_9397	Oryzias latipes	--------------------HFNRYLCRPRRVEMANLLNLTERQIKIWLQNRRMKYKKDQ	HOXA3A (Fragment) (hoxa3a)
DH_613	Rattus norvegicus	HPRGCTAYTSAQLVELEKEFHFNRYLCRRRRVEMANLLNLTERQIKIWFQNRRMKYKKDQ	Homeobox protein Hox-D3 (Fragment) (Hoxd3)
DH_9396	Oryzias latipes	--------------------HFNRYLCRPRRVEMANLLNLTERQIKIWFQNRRMKYKKDQ	HOXD3A (Fragment) (hoxd3a)
DH_2167	Haplochromis burtoni	SKRVKTAYTSAQLVELEKEFHFNRYLCRPRRVEMANLLNLTERQIKIWFQNRRMKYKKDQ	Hoxd3a (hoxd3a)
DH_6188	Oryzias latipes	SKRARTAYTSAQLVELEKEFHFNRYLCRPRRVEMANLLNLTERQIKIWFQNRRMKYKKDQ	HoxA3a (hoxA3a)
DH_11793	Gasterosteus aculeatus	SKRARTAYTSAQLVELEKEFHFNRYLCRPRRVEMANLLNLTERQIKIWFQNRRMKYKKDQ	Homeobox protein Hox-A3 (Hox-1E) [Source:UniProtKB/Swiss-Prot;Acc:O43365]
DH_392	Fugu rubripes	SKRARTAYTSAQLVELEKEFHFNRYLCRPRRVEMANLLNLTERQIKIWFQNRRMKYKKDQ	Homeobox protein Hox-A3a (hoxa3a)
DH_3289	Salmo salar	SKRARTAYTSAQLVELEKEFHFNRYLCRPRRVEMANLLNLTERQIKIWFQNRRMKYKKDQ	Homeobox protein HoxA3aa (HoxA3aa)
DH_391	Danio rerio	SKRARTAYTSAQLVELEKEFHFNRYLCRPRRVEMANLLNLTERQIKIWFQNRRMKYKKDQ	Homeobox protein Hox-A3a (hoxa3a)
DH_389	Homo sapiens	SKRARTAYTSAQLVELEKEFHFNRYLCRPRRVEMANLLNLTERQIKIWFQNRRMKYKKDQ	Homeobox protein Hox-A3 (HOXA3)
DH_2809	Rhinolophus ferrumequinum	SKRARTAYTSAQLVELEKEFHFNRYLCRPRRVEMANLLNLTERQIKIWFQNRRMKYKKDQ	Homeobox A3 isoform a (Predicted) (HOXA3)
DH_3190	Sorex araneus	SKRARTAYTSAQLVELEKEFHFNRYLCRPRRVEMANLLNLTERQIKIWFQNRRMKYKKDQ	Homeobox A3 (Predicted) (HOXA3)
DH_388	Heterodontus francisci	SKRARTAYTSAQLVELEKEFHFNRYLCRPRRVEMANLLNLTERQIKIWFQNRRMKYKKDQ	Homeobox protein Hox-A3 (HOXA3)
DH_387	Gallus gallus	SKRARTAYTSAQLVELEKEFHFNRYLCRPRRVEMANLLNLTERQIKIWFQNRRMKYKKDQ	Homeobox protein Hox-A3 (HOXA3)
DH_8274	Xenopus laevis	SKRARTAYTSAQLVELEKEFHFNRYLCRPRRVEMANLLNLTERQIKIWFQNRRMKYKKDQ	Hoxa3a-prov protein (hoxa3)
DH_2805	Xenopus tropicalis	SKRARTAYTSAQLVELEKEFHFNRYLCRPRRVEMANLLNLTERQIKIWFQNRRMKYKKDQ	Hoxa3 protein (hoxa3)
DH_386	Bos taurus	SKRARTAYTSAQLVELEKEFHFNRYLCRPRRVEMANLLNLTERQIKIWFQNRRMKYKKDQ	Homeobox protein Hox-A3 (HOXA3)
DH_390	Mus musculus	SKRARTAYTSAQLVELEKEFHFNRYLCRPRRVEMANLLNLTERQIKIWFQNRRMKYKKDQ	Homeobox protein Hox-A3 (Hoxa3)
DH_2745	Callicebus moloch	SKRARTAYTSAQLVELEKEFHFNRYLCRPRRVEMANLLNLTERQIKIWFQNRRMKYKKDQ	Homeobox A3 isoform a (Predicted) (HOXA3)
DH_2574	Callithrix jacchus	SKRARTAYTSAQLVELEKEFHFNRYLCRPRRVEMANLLNLTERQIKIWFQNRRMKYKKDQ	Homeobox A3 isoform a (Predicted) (HOXA3)
DH_1356	Gorilla gorilla gorilla	SKRARTAYTSAQLVELEKEFHFNRYLCRPRRVEMANLLNLTERQIKIWFQNRRMKYKKDQ	HOXA3 (Fragment) (HOXA3)
DH_2444	Papio anubis	SKRARTAYTSAQLVELEKEFHFNRYLCRPRRVEMANLLNLTERQIKIWFQNRRMKYKKDQ	Homeobox A3, isoform 2 (Predicted) (HOXA3)
DH_1509	Macaca mulatta	SKRARTAYTSAQLVELEKEFHFNRYLCRPRRVEMANLLNLTERQIKIWFQNRRMKYKKDQ	HOXA3 (Fragment) (HOXA3)
DH_1389	Pan paniscus	SKRARTAYTSAQLVELEKEFHFNRYLCRPRRVEMANLLNLTERQIKIWFQNRRMKYKKDQ	HOXA3 (Fragment) (HOXA3)
DH_1688	Homo sapiens	SKRARTAYTSAQLVELEKEFHFNRYLCRPRRVEMANLLNLTERQIKIWFQNRRMKYKKDQ	Homeo box A3 (cDNA FLJ36881 fis, clone BGGI12001247, highly similar to HOMEOBOX PROTEIN HOX-A3) (HOXA3)
DH_2966	Homo sapiens	SKRARTAYTSAQLVELEKEFHFNRYLCRPRRVEMANLLNLTERQIKIWFQNRRMKYKKDQ	cDNA FLJ32005 fis, clone NT2RP7009439, highly similar to HOMEOBOX PROTEIN HOX-A3
DH_1687	Homo sapiens	SKRARTAYTSAQLVELEKEFHFNRYLCRPRRVEMANLLNLTERQIKIWFQNRRMKYKKDQ	Homeo box A3 (Homeobox A3, isoform CRA_a) (HOXA3)
DH_3299	Salmo salar	SKRARTAYTSAQLVELEKEFHFNRYLCRPRRVEMANLLNLTERQIKIWFQNRRMKYKKDQ	Homeobox protein HoxA3ab (HoxA3ab)
DH_3335	Salmo salar	SKRARTAYTSAQLVELEKEFHFNRYLCRPRRVEMANLLNLTERQIKIWFQNRRMKYKKDQ	Homeobox protein HoxA3aa (Fragment) (HoxA3aa)
DH_2154	Haplochromis burtoni	SKRARTAYTSAQLVELEKEFHFNRYLCRPRRVEMANLLNLTERQIKIWFQNRRMKYKKDQ	Hoxa3a (hoxa3a)
DH_9343	Notophthalmus viridescens	SKRAATAYTSAQLVELEKEFHFNRYLCRPRRVEMANLLSLTERQIKIWFQNRRMKYKKDQ	Homeobox NVHBOX-2.7 protein (Fragment)
DH_9321	Notophthalmus viridescens	SKRAATAYTSAQLVELEKEFHFNRYLCRPRRVEMANLLSLTERQIKIWFQNRRMKYKKDQ	HBOX-2.7 (Fragment)
DH_7679	Pleurodeles waltlii	SKRARTAYTSAQLVELEKEFHFNRYLCRPRRVEMANLLSLSERQIKIWFQNRRMKYKKDQ	Transcription factor HoxB3
DH_9339	Gallus gallus	------------------EFHFNRYLCRPRRVEMANLLNLSERQIKIWFQNRRMKYKKDE	HOXB-3 protein (Fragment) (HOXB-3)
DH_1643	Carassius auratus	-------------------FHFNRYLCRPRRVEMANLLNLSERQIKIWFQNRRM------	HoxB3a protein (Fragment) (Hox)
DH_2962	Homo sapiens	SERARTAYTSAQLVELEKEFHFNRYLCRPRRVEMANLLNLSERQIKIWFQNRRMKYKKDQ	cDNA FLJ14703 fis, clone NT2RP3000512, highly similar to Homeobox protein Hox-B3
DH_1579	Macaca nemestrina	SKRARTAYTSXQLVELEKEFHFNRYLCRPRRVEMANLLNLSERQIKIWFQNRRMKYKKDQ	HOXB3 (Fragment) (HOXB3)
DH_1642	Carassius auratus	-------------------IHFNKYLCRPRRVEMANLLNLSERQIKIWFQNRRM------	HoxB3a protein (Fragment) (Hox)
DH_11817	Gasterosteus aculeatus	SKRARTAYTSAQLVELEKEFHFNRYLCRPRRVEMANLLNLSERQIKIWFQNRRMKYKKDQ	Homeobox protein Hox-B3 (Hox-2G) (Hox-2.7) [Source:UniProtKB/Swiss-Prot;Acc:P14651]
DH_6721	Oreochromis niloticus	SKRARTAYTSAQLVELEKEFHFNRYLCRPRRVEMANLLNLSERQIKIWFQNRRMKYKKDQ	Hox protein (Fragment)
DH_483	Fugu rubripes	SKRARTAYTSAQLVELEKEFHFNRYLCRPRRVEMANLLNLSERQIKIWFQNRRMKYKKDQ	Homeobox protein Hox-B3a (hoxb3a)
DH_7484	Fugu rubripes	SKRARTAYTSAQLVELEKEFHFNRYLCRPRRVEMANLLNLSERQIKIWFQNRRMKYKKDQ	Hoxb3a
DH_8291	Morone saxatilis	SKRARTAYTSAQLVELEKEFHFNRYLCRPRRVEMANLLNLSERQIKIWFQNRRMKYKKDQ	Hoxb3a protein (Hoxb3a)
DH_6173	Oryzias latipes	SKRARTAYTSAQLVELEKEFHFNRYLCRPRRVEMANLLNLSERQIKIWFQNRRMKYKKDQ	HoxB3a (hoxB3a)
DH_481	Xenopus laevis	SKRARTAYTSAQLVELEKEFHFNRYLCRPRRVEMANLLNLSERQIKIWFQNRRMKYKKDQ	Homeobox protein Hox-B3 (Fragment) (hoxb3)
DH_478	Gallus gallus	SKRARTAYTSAQLVELEKEFHFNRYLCRPRRVEMANLLNLSERQIKIWFQNRRMKYKKDQ	Homeobox protein Hox-B3 (HOXB3)
DH_8017	Latimeria menadoensis	SKRARTAYTSAQLVELEKEFHFNRYLCRPRRVEMANLLNLSERQIKIWFQNRRMKYKKDQ	HoxB3 (Fragment)
DH_482	Danio rerio	SKRARTAYTSAQLVELEKEFHFNRYLCRPRRVEMANLLNLSERQIKIWFQNRRMKYKKDQ	Homeobox protein Hox-B3a (hoxb3a)
DH_1535	Megalobrama amblycephala	SKRARTAYTSAQLVELEKEFHFNRYLCRPRRVEMANLLNLSERQIKIWFQNRRMKYKKDQ	Homeodomain protein (Fragment) (HoxB3a)
DH_3312	Salmo salar	SKRARTAYTSAQLVELEKEFHFNRYLCRPRRVEMANLLNLSERQIKIWFQNRRMKYKKDQ	Homeobox protein HoxB3aa (HoxB3aa)
DH_3329	Salmo salar	SKRARTAYTSAQLVELEKEFHFNRYLCRPRRVEMANLLNLSERQIKIWFQNRRMKYKKDQ	Homeobox protein HoxB3aa3 (HoxB3aa)
DH_3328	Salmo salar	SKRARTAYTSAQLVELEKEFHFNRYLCRPRRVEMANLLNLSERQIKIWFQNRRMKYKKDQ	Homeobox protein HoxB3aa2 (HoxB3aa)
DH_3321	Salmo salar	SKRARTAYTSAQLVELEKEFHFNRYLCRPRRVEMANLLNLSERQIKIWFQNRRMKYKKDQ	Homeobox protein HoxB3ab (Homeobox protien HoxB3ab) (HoxB3ab)
DH_5724	Xenopus tropicalis	SKRARTAYTSAQLVELEKEFHFNRYLCRPRRVEMANLLNLSERQIKIWFQNRRMKYKKDQ	Homeo box B3 (hoxb3)
DH_479	Homo sapiens	SKRARTAYTSAQLVELEKEFHFNRYLCRPRRVEMANLLNLSERQIKIWFQNRRMKYKKDQ	Homeobox protein Hox-B3 (HOXB3)
DH_1359	Gorilla gorilla gorilla	SKRARTAYTSAQLVELEKEFHFNRYLCRPRRVEMANLLNLSERQIKIWFQNRRMKYKKDQ	HOXB3 (Fragment) (HOXB3)
DH_1606	Pongo pygmaeus	SKRARTAYTSAQLVELEKEFHFNRYLCRPRRVEMANLLNLSERQIKIWFQNRRMKYKKDQ	HOXB3 (Fragment) (HOXB3)
DH_1812	Bos taurus	SKRARTAYTSAQLVELEKEFHFNRYLCRPRRVEMANLLNLSERQIKIWFQNRRMKYKKDQ	HOXB3 protein (HOXB3)
DH_480	Mus musculus	SKRARTAYTSAQLVELEKEFHFNRYLCRPRRVEMANLLNLSERQIKIWFQNRRMKYKKDQ	Homeobox protein Hox-B3 (Hoxb3)
DH_1372	Saguinus labiatus	SKRARTAYTSAQLVELEKEFHFNRYLCRPRRVEMANLLNLSERQIKIWFQNRRMKYKKDQ	HOXB3 (Fragment) (HOXB3)
DH_2717	Mus musculus	SKRARTAYTSAQLVELEKEFHFNRYLCRPRRVEMANLLNLSERQIKIWFQNRRMKYKKDQ	Homeo box B3 (Hoxb3)
DH_9035	Mesocricetus auratus	SKRARTAYTSAQLVELEKEFHFNRYLCRPRRVEMANLLNLSERQIKIWFQNRRMKYKKDQ	Homeobox B3 (hox b3)
DH_2869	Rattus norvegicus	SKRARTAYTSAQLVELEKEFHFNRYLCRPRRVEMANLLNLSERQIKIWFQNRRMKYKKDQ	Hoxb3 protein (Hoxb3)
DH_3326	Salmo salar	SKRARTAYTSAQLVELEKEFHFNRYLCRPRRLEMASQLSLKERQIKIWFQNRRMKCKKDG	Homeobox protein HoxB3ba (HoxB3ba)
DH_5006	Platynereis dumerilii	SKRARTAYTSAQLVELEKEFHFNRYLCRPR------------------------------	Hox3 homeobox protein (Fragment) (Hox3)
DH_7653	Perionyx excavatus	SKRARTAYTSAQLVELEKEFHFNRYLCRPRRVELATHLSLTERQIKIWFQNRR-------	Homeobox protein Hox3b (Fragment)
DH_9346	Xenopus laevis	SKRARTAYTNSQLVELEKEFHFNRYLCRPRRLEMAKLLNLSERQIKIWFQNRRMKFKKDH	XHOX2.7 homeodomain protein (Fragment)
DH_6278	Ciona intestinalis	SKRERTAYTNSQLVELEKEFHFSHYLCRPRRIELAQGLGLTERQIKIWFQNRRMKFKKEQ	Transcription factor protein (Ci-Hox3)
DH_9611	Ciona intestinalis	SKRERTAYTNSQLVELEKEFHFSHYLCRPRRIELAQGLGLTERQIKIWFQNRRMKFKKEQ	Homeoprotein (Hox3)
DH_4611	Archegozetes longisetosus	AKRARTAYTSSQLVELEKEFHTSRYLCRPRRIEMASLLKLSERQIKIWFQNRRMKFKKEQ	Hox 3 (Fragment)
DH_7654	Perionyx excavatus	SKRTRTAYSSSQLVELEKEFHFNRYLCRPRRIEMASMLKLSERQIKIWFQNRR-------	Homeobox protein Hox3a (Fragment)
DH_5847	Oncopeltus fasciatus	AKRARTAYTSIQLVELEKEFHFNRYLCRPRRIEMATQLRLSERQIKIWFQNRRMKYKKER	Predicted zerknuellt protein (zen)

DH_1670	Strigamia maritima	NKRSRTAYTQSQLLELEKEFHFNRYLCRPRRLELASLLNLTERQIKIWFQNRRMKIKKIK	Homeodomain transcription factor Hox3a (Fragment)
DH_1671	Strigamia maritima	NKRSRTAYTQSQLVELEKEFHFNRYLCRPRRVELASMLNLTERQIKIWFQNRRMKNKKIK	Homeodomain transcription factor Hox3b
DH_9259	Pachymerium ferrugineum	--------TQQQLVELEKEFHFNRYLCRPRRVELANMLQLSERQIKIW------------	Putative Hox3 / zerknuellt (Fragment) (hox3/zen)
DH_2145	Haplochromis burtoni	VKRERTAFTNNQLLELEKEFHFSPYLCRPRRLEMAAGLQLTDRQVKIWFQNRRMRYKKEQ	Hoxc3a (hoxc3a)
DH_6713	Oreochromis niloticus	VKRERTAFTNNQLLELEKEFHFSPYLCRPRRLEMAAGLQLTDRQVKIWFQNRRMRYKKEQ	Hox protein (Fragment)
DH_6162	Oryzias latipes	VKRERTAFTNSQLLELEKEFHFSPYLCRRRRLEMAAGLQLTDQQVKIWFQNRRMRHKNKN	HoxC3a (hoxC3a)
DH_9395	Oryzias latipes	--------------------HFSPYLCRRRRLEMAAGLQLTDQQVKIWFQNRRMRHKNKN	HOXC3A (Fragment) (hoxc3a)
DH_11777	Gasterosteus aculeatus	VRRERTAFTKSQLLELEKEFHFSPYLCRSRRLEMAAGLRLTDRQVKIWFQNRRMRHKKEQ	groupXII
DH_3357	Salmo salar	GRRARTAFTSTQLVELEKEFHFSPYLCRPRRLEMAAGLRLTDRQIKIWFQNRRMKYKKDH	Homeobox protein HoxC3aa (Fragment) (HoxC3aa)
DH_544	Danio rerio	SKRARVAFTSSQLLELEKEFHFSAYLCRNRRLEMAELLKLTDRQIKIWFQNRRMKYKKDH	Homeobox protein Hox-C3a (hoxc3a)
DH_1460	Danio rerio	SKRARVAFTSSQLLELEKEFHFSAYLCRNRRLEMAELLKLTDRQIKIWFQNRRMKYKKDH	Homeo box C3a (hoxc3a)
DH_6683	Danio rerio	SKRARVAFTSSQLLELEKEFHFSAYLCRNRRLEMAELLKLTDRQIKIWFQNRRMKYKKDH	LOC100151318 protein (hoxc3a)
DH_1545	Megalobrama amblycephala	SKRARAAFTSAQLLELEKEFHFSAYLCRPRRLEMAALLKLTDRQIKIWFQNRRMKYKKDH	Homeodomain protein (Fragment) (HoxC3a)

Save alignment [fasta format] - Save complete sequences [fasta format]

PG4

ID	organism	10 20 30 40 50 60	description
DH_9391	Oryzias latipes	--------------------HFNRYLTRRRRIEIAHTLCLSERQIKIWFQNRRMKWKKDH	HOXD4A (Fragment) (hoxd4a)
DH_618	Gallus gallus	PKRSRTAYTRQQVLELEKEFHFNRYLTRRRRIEIAHTLCLSERQIKIWFQNRRMKWKKDH	Homeobox protein Hox-D4 (HOXD4)
DH_1577	Macaca nemestrina	PKRSRTAYTRQQVLELEKEFHFNRYLTRRRRIEIAHTLCLSERQIKIWFQNRRMKWKKDH	HOXD4 (Fragment) (HOXD4)
DH_1508	Macaca mulatta	PKRSRTAYTRQQVLELEKEFHFNRYLTRRRRIEIAHTLCLSERQIKIWFQNRRMKWKKDH	HOXD4 (Fragment) (HOXD4)
DH_1939	Bos taurus	PKRSRTAYTRQQVLELEKEFHFNRYLTRRRRIEIAHTLCLSERQIKIWFQNRRMKWKKDH	HOXD4 protein (HOXD4)
DH_1446	Mus musculus	PKRSRTAYTRQQVLELEKEFHFNRYLTRRRRIEIAHTLCLSERQIKIWFQNRRMKWKKDH	Homeo box D4 (Hoxd4)
DH_622	Mus musculus	PKRSRTAYTRQQVLELEKEFHFNRYLTRRRRIEIAHTLCLSERQIKIWFQNRRMKWKKDH	Homeobox protein Hox-D4 (Hoxd4)
DH_625	Saguinus labiatus	PKRSRTAYTRQQVLELEKEFHFNRYLTRRRRIEIAHTLCLSERQIKIWFQNRRMKWKKDH	Homeobox protein Hox-D4 (HOXD4)
DH_619	Gorilla gorilla gorilla	PKRSRTAYTRQQVLELEKEFHFNRYLTRRRRIEIAHTLCLSERQIKIWFQNRRMKWKKDH	Homeobox protein Hox-D4 (HOXD4)
DH_624	Pan troglodytes	PKRSRTAYTRQQVLELEKEFHFNRYLTRRRRIEIAHTLCLSERQIKIWFQNRRMKWKKDH	Homeobox protein Hox-D4 (HOXD4)
DH_623	Pan paniscus	PKRSRTAYTRQQVLELEKEFHFNRYLTRRRRIEIAHTLCLSERQIKIWFQNRRMKWKKDH	Homeobox protein Hox-D4 (HOXD4)
DH_2832	Homo sapiens	PKRSRTAYTRQQVLELEKEFHFNRYLTRRRRIEIAHTLCLSERQIKIWFQNRRMKWKKDH	cDNA, FLJ94520, highly similar to Homo sapiens homeo box D4 (HOXD4), mRNA
DH_620	Homo sapiens	PKRSRTAYTRQQVLELEKEFHFNRYLTRRRRIEIAHTLCLSERQIKIWFQNRRMKWKKDH	Homeobox protein Hox-D4 (HOXD4)
DH_621	Lagothrix lagotricha	PKRSRTAYTRQQVLELEKEFHFNRYLTRRRRIEIAHTLCLSERQIKIWFQNRRMKWKKDH	Homeobox protein Hox-D4 (HOXD4)
DH_617	Ateles geoffroyi	PKRSRTAYTRQQVLELEKEFHFNRYLTRRRRIEIAHTLCLSERQIKIWFQNRRMKWKKDH	Homeobox protein Hox-D4 (HOXD4)
DH_2555	Danio rerio	PKRSRTAYTRQQVLELEKEFHFNRYLTRRRRIEIAHTLCLSERQIKIWFQNRRMKWKKDH	Homeo box D4a (Hoxd4a protein) (hoxd4a)
DH_6705	Oreochromis niloticus	PKRSRTAYTRQQVLELEKEFHFNRYLTRRRRIEIAHTLCLSERQIKIWFQNRRMKWKKDH	Hox protein (Fragment)
DH_11737	Gasterosteus aculeatus	PKRSRTAYTRQQVLELEKEFHFNRYLTRRRRIEIAHTLCLSERQIKIWFQNRRMKWKKDH	Homeobox protein Hox-D4 (Hox-4B) (Hox-5.1) (HHO.C13) [Source:UniProtKB/Swiss-Prot;Acc:P09016]
DH_6151	Oryzias latipes	PKRSRTAYTRQQVLELEKEFHFNRYLTRRRRIEIAHTLCLSERQIKIWFQNRRMKWKKDH	HoxD4a (hoxD4a)
DH_9580	Paralichthys olivaceus	PKRSRTAYTRQQVLELEKEFHFNRYLTRRRRIEIAHTLCLSERQIKIWFQNRRMKWKKDH	Hoxd-4 (Hoxd-4)
DH_2166	Haplochromis burtoni	PKRSRTAYTRQQVLELEKEFHFNRYLTRRRRIEIAHTLCLSERQIKIWFQNRRMKWKKDH	Hoxd4a (hoxd4a)
DH_628	Fugu rubripes	PKRSRTAYTRQQVLELEKEFHFNRYLTRRRRIEIAHTLCLSERQIKIWFQNRRMKWTKDH	Homeobox protein Hox-D4a (hoxd4a)
DH_8002	Latimeria menadoensis	PKRSRTAYTRQQVLELEKEFHFNRYLTRRRRIEIAHTLCLSERQIKIWFQNRRMKWEKDH	HoxD4 (Fragment)
DH_3274	Salmo salar	PKRSRTAYTRQQVLELEKEFHFNRYLTRRRRIEIAHTLVLSERQIKIWFQNRRMKWKKDH	Homeobox protein HoxD4aa (HoxD4aa)
DH_6777	Oncorhynchus mykiss	PKRSRTAYTRQQVLELEKEFHFNRYLTRRRRIEIAHTLVLSERQ----------------	HoxD4ai (Fragment) (Hox)
DH_3281	Salmo salar	PKRSRTAYTRQQVLELEKEFHFNRYLTRRRRIEIAHTLVLSERQIKIWFQNRRMKWKKDH	Homeobox protein HoxD4ab (HoxD4ab)
DH_627	Danio rerio	PKRSRTAYTRQQVLELEKEFHFNRYLTRRRRIESAHTLSLSERQIKIWFQNRRMKWKKDH	Homeobox protein Hox-D4a (hoxd4a)
DH_6512	Tetraodon nigroviridis	PKRSRTAYTRQQVLELEKEFHYNRYLTRRRRVEIAHTLCLSERQIKIWFQNRRMKWKKDH	Chromosome undetermined SCAF14653, whole genome shotgun sequence (GSTENG00020452001)
DH_11830	Gasterosteus aculeatus	PKRSRTAYTRQQVLELEKEFHYNRYLTRRRRVEIAHTLCLSERQIKIWFQNRRMKWKKDH	Homeobox protein Hox-B4 (Hox-2F) (Hox-2.6) [Source:UniProtKB/Swiss-Prot;Acc:P17483]
DH_490	Fugu rubripes	PKRSRTAYTRQQVLELEKEFHYNRYLTRRRRVEIAHTLCLSERQIKIWFQNRRMKWKKDH	Homeobox protein Hox-B4a (hoxb4a)
DH_6172	Oryzias latipes	PKRSRTAYTRQQVLELEKEFHYNRYLTRRRRVEIAHTLCLSERQIKIWFQNRRMKWKKDH	HoxB4a (hoxB4a)
DH_6720	Oreochromis niloticus	PKRSRTAYTRQQVLELEKEFHYNRYLTRRRRVEIAHTLCLSERQIKIWFQNRRMKWKKDH	Hox protein (Fragment)
DH_489	Danio rerio	PKRSRTAYTRQQVLELEKEFHYNRYLTRRRRVEIAHTLCLSERQIKIWFQNRRMKWKKDH	Homeobox protein Hox-B4a (hoxb4a)
DH_2914	Danio rerio	PKRSRTAYTRQQVLELEKEFHYNRYLTRRRRVEIAHTLCLSERQIKIWFQNRRMKWKKDH	Homeo box B4a (hoxb4a)
DH_4805	Danio rerio	PKRSRTAYTRQQVLELEKEFHYNRYLTRRRRVEIAHTLCLSERQIKIWFQNRRMKWKKDH	Hoxb4a variant 2 (hoxb4a)
DH_6788	Oncorhynchus mykiss	PKRSRTAYTRQQVLELEKEFHYNRYLTRRRRVEIAHTLVLSERQIKIWFQNR--------	HoxB4aii (Fragment) (Hox)
DH_6789	Oncorhynchus mykiss	PKRSRTAYTRQQVLELEKEFHYNRYLTRRRRVEIAHTLVLSERQIKIWFQNRRMK-----	HoxB4ai (Fragment) (Hox)
DH_3311	Salmo salar	PKRSRTAYTRQQVLELEKEFHYNRYLTRRRRVEIAHTLVLSERQIKIWFQNRRMKWKKDH	Homeobox protein HoxB4aa (HoxB4aa)
DH_3320	Salmo salar	PKRSRTAYTRQQVLELEKEFHYNRYLTRRRRVEIAHTLVLSERQIKIWFQNRRMKWKKDH	Homeobox protein HoxB4ab (Homeobox protien HoxB4ab) (HoxB4ab)
DH_2780	Xenopus tropicalis	AKRSRTAYTRQQVLELEKEFHYNRYLTRRRRVEIAHTLRLSERQIKIWFQNRRMKWKKDH	Hoxb4 protein (hoxb4)
DH_6997	Xenopus tropicalis	AKRSRTAYTRQQVLELEKEFHYNRYLTRRRRVEIAHTLRLSERQIKIWFQNRRMKWKKDH	Hoxb4 protein (Fragment) (hoxb4)
DH_488	Xenopus laevis	AKRSRTAYTRQQVLELEKEFHYNRYLTRRRRVEIAHTLRLSERQIKIWFQNRRMKWKKDH	Homeobox protein Hox-B4 (hoxb4)
DH_485	Gallus gallus	PKRSRTAYTRQQVLELEKEFHYNRYLTRRRRVEIAHSLCLSERQIKIWFQNRRMKWKKDH	Homeobox protein Hox-B4 (HOXB4)
DH_8016	Latimeria menadoensis	PKRSRTAYTRQQVLELEKEFHYNRYLTRRRRVEIAHSLCLSERQIKIWFQNRRMKWKKDH	HoxB4
DH_484	Bos taurus	PKRSRTAYTRQQVLELEKEFHYNRYLTRRRRVEIAHALCLSERQIKIWFQNRRMKWKKDH	Homeobox protein Hox-B4 (HOXB4)
DH_486	Homo sapiens	PKRSRTAYTRQQVLELEKEFHYNRYLTRRRRVEIAHALCLSERQIKIWFQNRRMKWKKDH	Homeobox protein Hox-B4 (HOXB4)
DH_487	Mus musculus	PKRSRTAYTRQQVLELEKEFHYNRYLTRRRRVEIAHALCLSERQIKIWFQNRRMKWKKDH	Homeobox protein Hox-B4 (Hoxb4)
DH_2716	Mus musculus	PKRSRTAYTRQQVLELEKEFHYNRYLTRRRRVEIAHALCLSERQIKIWFQNRRMKWKKDH	Homeo box B4 (Hoxb4)
DH_2784	Rattus norvegicus	PKRSRTAYTRQQVLELEKEFHYNRYLTRRRRVEIAHALCLSERQIKIWFQNRRMKWKKDH	Hoxb4 protein (Similar to homeotic protein Hox B4-mouse) (Predicted) (Hoxb4)
DH_7287	Mus musculus	PKRSRTAYTRQQVLELEKEFHYNRYLTRRRRVEIAHALCLSERQIKIWFQNRRMKWKKDH	Homeobox protein (Hox-2.6) (Fragment)
DH_9393	Oryzias latipes	--------------------HYNRYLTRRRRVEIAHTLCLSERQIKIWFQNRRMKWKKDH	HOXB4A (Fragment) (hoxb4a)
DH_8724	Petromyzon marinus	LNGARTAYTRHQALELEKEFHFNRYLTRRRRVEVANALCLSERQIKIWFQNRRMKWKKDH	Homeobox protein hox4x (Fragment)
DH_397	Morone saxatilis	PKRSRTAYTRQQALELEKEFHFNRYLTRRRRVEIAHTMCLSERQVKIWFQNRRMKWKKEH	Homeobox protein Hox-A4 (hoxa4)
DH_9394	Oryzias latipes	--------------------HFNRYLTRRRRVEIAHTMCLSERQVKIWFQNRRMKWKKEH	HOXA4A (Fragment) (hoxa4a)
DH_6187	Oryzias latipes	PKRSRTAYTRQQALELEKEFHFNRYLTRRRRVEIAHTMCLSERQVKIWFQNRRMKWKKEH	HoxA4a (hoxA4a)
DH_402	Fugu rubripes	PKRSRTAYTRQQALELEKEFHFNRYLTRRRRVEIAHTMCLSERQVKIWFQNRRMKWKKEH	Homeobox protein Hox-A4a (hoxa4a)
DH_2153	Haplochromis burtoni	PKRSRTAYTRQQALELEKEFHFNRYLTRRRRVEIAHTMCLSERQVKIWFQNRRMKWKKEH	Hoxa4a (hoxa4a)
DH_1526	Megalobrama amblycephala	PKRSRTAYTRQQALELEKEFHFNRYLTRRRRVEIAHTMCLSERQVKIW------------	Homeodomain protein (Fragment) (HoxA4a)
DH_11762	Gasterosteus aculeatus	PKRSRTAYTRQQALELEKEFHFNRYLTRRRRVEIAHTMCLSERQVKIWFQNRRMKWKKEH	groupX
DH_401	Danio rerio	PKRSRTAYTRQQALELEKEFHFNRYLTRRRRVEIAHTMCLSERQVKIWFQNRRMKWKKDH	Homeobox protein Hox-A4a (hoxa4a)

```
DH_3298  Salmo salar                       PKRSRTAYTRQQALELEKEFHFNRYLTRRRRVEIAHTMCLSERQVKIWFQNRRMKWKKDH Homeobox protein HoxA4an (Homeobox protein HoxA4ab) (HoxA4ab)
DH_6791  Oncorhynchus mykiss               PKRSRTAYTRQQALELEKEFHFNRYLTRRRRVEIAHTMCLSERQVKIWFQNRRMKWKKDH HoxA4ai (Fragment) (Hox)
DH_3288  Salmo salar                       PKRSRTAYTRQQALELEKEFHFNRYLTRRRRVEIAHTMCLTERQVKIWFQNRRMKWKKDH Homeobox protein HoxA4aa (HoxA4aa)
DH_6790  Oncorhynchus mykiss               PKRSRTAYTRQQALELEKEFHFNRYLTRRRRVEIAHTMCLTERQVKIWFQNRRMKWKKDH HoxA4aii (Fragment) (Hox)
DH_2169  Haplochromis burtoni              PKRLRTAYTRQQVLELEKEFHFSRYLTRRRRVEVAHALCLSERQVKVWFQNRRMRWKKDN Hox4b (hoxd4b)
DH_9392  Oryzias latipes                   --------------------HFSRYLTRRRRVEVAHALCLSERQVKVWFQNRRMRWKKDN HOXD4B (Fragment) (hoxd4b)
DH_6146  Oryzias latipes                   PKRLRTAYTRQQVLELEKEFHFSRYLTRRRRVEVAHALCLSERQVKVWFQNRRMRWKKDN HoxD4b (hoxD4b)
DH_7716  Sphoeroides nephelus              PKRLRTAYTRQQVLELEKEFHFSRYLTRRRRVEVAHTLCLSERQVKVWFQNRRMRWKKDN HoxD4b
DH_6530  Tetraodon nigroviridis            PKRLRTAYTRQQVLELEKEFHFSRYLTRRRRVEVAHTLCLSERQVKVWFQNRRMRWKKDN Chromosome 17 SCAF14597, whole genome shotgun sequence. (Fragment) (GSTENG00018723001)
DH_629   Fugu rubripes                     PKRLRTAYTRQQVLELEKEFHFSRYLTRRRRVEVAHTLCLSERQVKVWFQNRRMRWKKDH Homeobox protein Hox-D4b (hoxd4b)
DH_11941 Gasterosteus aculeatus            PKRLRTAYTRPQVLELEKEFHFSRYLTRRRRVEVAHALYLSERQVKVWFQNRRMRWKKDH groupVI
DH_6029  Bos taurus                        PKRSRTAYTRQQVLELEKEFHFNRYLTRRRRIEIAHKLCLSERQVKIWFQNRRMKWKKDH Homeobox A4 (HOXA4)
DH_3191  Sorex araneus                     PKRSRTAYTRQQVLELEKEFHFNRYLTRRRRIEIAHALCLSERQVKIWFQNRRMKWKKDH Homeobox A4 (Predicted) (HOXA4)
DH_7292  Rattus norvegicus                 ----------------------FNRYLTRRRRIEIAHTLCLSERQVKIWFQNRRMKWKKDH Hox1.4 protein (Fragment) (Hoxa4)
DH_399   Rattus norvegicus                 ------------------EFHFNRYLTRRRRIEIAHTLCLSERQVKIWFQNRRMKWKKDH Homeobox protein Hox-A4 (Fragment) (Hoxa4)
DH_7527  Rattus sp                         ------------------EFHFNRYLTRRRRIEIAHTLCLSERQVKIWFQNRRMKWKKDH Hox-1|Hox-A (Fragment) (Hox-A|Hox-1)
DH_6277  Ciona intestinalis                GKRPRTAYTRHQVLELEKEFHYNRYLTRRRRIEIAHGLCLSERQVKIWFQNRRMKWKKDH Transcription factor protein (Ci-Hox4)
DH_400   Ovis aries                        ------AYTRQQVLELEKEFHFNRYLTRRRRIEIAHTLCLSEAQVKIWFQNRRMKWKKDH Homeobox protein Hox-A4 (Fragment) (HOXA4)
DH_395   Homo sapiens                      PKRSRTAYTRQQVLELEKEFHFNRYLTRRRRIEIAHTLCLSERQVKIWFQNRRMKWKKDH Homeobox protein Hox-A4 (HOXA4)
DH_1686  Homo sapiens                      PKRSRTAYTRQQVLELEKEFHFNRYLTRRRRIEIAHTLCLSERQVKIWFQNRRMKWKKDH Homeo box A4 (HOXA4)
DH_2445  Papio anubis                      PKRSRTAYTRQQVLELEKEFHFNRYLTRRRRIEIAHTLCLSERQVKIWFQNRRMKWKKDH Homeobox A4 (Predicted) (HOXA4)
DH_2575  Callithrix jacchus                PKRSRTAYTRQQVLELEKEFHFNRYLTRRRRIEIAHTLCLSERQVKIWFQNRRMKWKKDH Homeobox A4 (Predicted) (HOXA4)
DH_2746  Callicebus moloch                 PKRSRTAYTRQQVLELEKEFHFNRYLTRRRRIEIAHTLCLSERQVKIWFQNRRMKWKKDH Homeobox A4 (Predicted) (HOXA4)
DH_1337  Homo sapiens                      PKRSRTAYTRQQVLELEKEFHFNRYLTRRRRIEIAHTLCLSERQVKIWFQNRRMKWKKDH HOXA4 protein (HOXA4)
DH_398   Mus musculus                      PKRSRTAYTRQQVLELEKEFHFNRYLTRRRRIEIAHTLCLSERQVKIWFQNRRMKWKKDH Homeobox protein Hox-A4 (Hoxa4)
DH_6053  Mus musculus                      PKRSRTAYTRQQVLELEKEFHFNRYLTRRRRIEIAHTLCLSERQVKIWFQNRRMKWKKDH Hoxa4 protein (Hoxa4)
DH_393   Gallus gallus                     PKRSRTAYTRQQVLELEKEFHFNRYLTRRRRIEIAHTLCLSERQVKIWFQNRRMKWKKDH Homeobox protein Hox-A4 (HOXA4)
DH_8027  Latimeria menadoensis             PKRSRTAYTRQQVLELEKEFHFNRYLTRRRRIEIAHTLCLSERQVKIWFQNRRMKWKKDH HoxA4 (Fragment)
DH_394   Heterodontus francisci            PKRSRTAYTRQQVLELEKEFHFNRYLTRRRRIEIAHTLCLSERQVKIWFQNRRMKWKKDH Homeobox protein Hox-A4 (HOXA4)
DH_7288  Mus musculus                      PKRSRTAYTRQQVLELEKEFHFNRYLTRRRRIEIAHTLCLSERQVKIWFQNRRMKWKKDH Homeobox protein (Hox1-3) (Fragment) (Hoxa4)
DH_7678  Pleurodeles waltlii               PKRSRTAYTRQQVLELEKEFHFNRYLTRRRRIEIAHTLCLSERQVKIWFQNRRMKWKKDH Transcription factor HoxA4 (Fragment)
DH_5970  Xenopus laevis                    PKRSRTAYTRQQVLELEKEFHFNRYLTRRRRIEIAHSLCLSERQIKIWFQNRRMKWKKDH Putative uncharacterized protein
DH_7675  Xenopus laevis                    PKRSRTAYTRQQVLELEKEFHFNRYLTRRRRIEIAHSLCLSECQIKIWFQNRRMKWKKDH HOXD4 (Fragment)
DH_549   Danio rerio                       PKRSRTAYTRQQVLELEKEFHYNRYLTRRRRIEIAHSLVLSERQIKIWFQNRRMKWKKDH Homeobox protein Hox-C4a (hoxc4a)
DH_1546  Megalobrama amblycephala          PKRSRTAYTRQQVLELEKEFHYNRYLTRRRRIEIAHSLVLSERQIKIWFQNRRMK----- Homeodomain protein (Fragment) (HoxC4a)
DH_550   Fugu rubripes                     PKRSRTAYTRQQVLELEKEFHYNRYLTRRRRIEIAHALVLSERQIKIWFQNRRMKWKKDH Homeobox protein Hox-C4a (hoxc4a)
DH_3241  Salmo salar                       SKRSRTAYTRQQVLELEKEFHYNRYLTRRRRIEIAHTLVLSERQIKIWFQNRRMKWKKDH Homeobox protein HoxC4aa (HoxC4aa)
DH_6785  Oncorhynchus mykiss               SKRSRTAYTRQQVLELEKEFHYNRYLTRRRRIEIAHTLVLPERQIKIWFQNRRMK----- HoxC4a-2 (Fragment) (Hox)
DH_3250  Salmo salar                       SKRSRTAYTRQQVLELEKEFHYNRYLTRRRRIEIAHTLVLSERQIKIWFQNRRMKWKKDH Homeobox protein HoxC4ab (HoxC4ab)
DH_3361  Salmo salar                       SKRSRTAYTRQQVLELEKEFHYNRYLTRRRRIEIAHTLVLSERQIKIWFQNRRMKWKKDH Homeobox protein HoxC4ab (HoxC4ab)
DH_6783  Oncorhynchus mykiss               TKRSRTAYTRQQVLELEKEFHYNRYLTRRRRIEIAHTLVLSERQIKIWFQNRRMK----- HoxC4bii (Fragment) (Hox)
DH_3260  Salmo salar                       AKRSRTAYTRQQVLELEKEFHYNRYLTRRRRIEIAHTLVLSERQIKIWFQNRRMKWKKDH Homeobox protein HoxC4ba (HoxC4ba)
DH_3267  Salmo salar                       AKRSRTAYTRQQVLELEKEFHYNRYLTRRRRIEIAHTLVLSERQIKIWFQNRRMKWKKDH Homeobox protein HoxC4bb (HoxC4bb)
DH_2144  Haplochromis burtoni              PKRSRTAYTRQQVLELEKEFHYNRYLTRRRRIEIAHTLVLSERQIKIWFQNRRMKWKKDH Hoxc4a (hoxc4a)
DH_11773 Gasterosteus aculeatus            PKRSRTAYTRQQVLELEKEFHYNRYLTRRRRIEIAHTLVLSERQIKIWFQNRRMKWKKDH Homeobox protein Hox-C4 (Hox-3E) (CP19) [Source:UniProtKB/Swiss-Prot;Acc:P09017]
DH_547   Oryzias latipes                   PKRSRTAYTRQQVLELEKEFHYNRYLTRRRRIEIAHTLVLSERQIKIWFQNRRMKWKKDH Homeobox protein Hox-C4 (hoxc4)
DH_6161  Oryzias latipes                   PKRSRTAYTRQQVLELEKEFHYNRYLTRRRRIEIAHTLVLSERQIKIWFQNRRMKWKKDH HoxC4a (hoxC4a)
DH_6712  Oreochromis niloticus             PKRSRTAYTRQQVLELEKEFHYNRYLTRRRRIEIAHTLVLSERQIKIWFQNRRMKWKKDH Hox protein (Fragment)
DH_7445  Bugula turrita                    NKRTRTAYTRQQVLELEKEFHYNRYLTQRRRIEIAHTLTLSERQIKIWFQNRRMKWKKDH Hox class homeodomain-containing protein Dfdb (Fragment)
DH_8267  Homo sapiens                      PKRSRTAYTRQQVLELEKEFHYNRYLTRRRRIEIAHSLCLSERQIKIWFQNRRMKWKKDH Homeobox C4 (HOXC4)
DH_1704  Bos taurus                        PKRSRTAYTRQQVLELEKEFHYNRYLTRRRRIEIAHSLCLSERQIKIWFQNRRMKWKKDH HOXC4 protein (HOXC4)
DH_546   Mus musculus                      PKRSRTAYTRQQVLELEKEFHYNRYLTRRRRIEIAHSLCLSERQIKIWFQNRRMKWKKDH Homeobox protein Hox-C4 (Hoxc4)
DH_2864  Mus musculus                      PKRSRTAYTRQQVLELEKEFHYNRYLTRRRRIEIAHSLCLSERQIKIWFQNRRMKWKKDH Homeo box C4 (Hoxc4)
DH_8010  Latimeria menadoensis             PKRSRTAYTRQQVLELEKEFHYNRYLTRRRRIEIAHSLCLSERQIKIWFQNRRMKWKKDH HoxC4 (Fragment)
DH_545   Homo sapiens                      PKRSRAAYTRQQVLELEKEFHYNRYLTRRRRIEIAHSLCLSERQIKIWFQNRRMKWKKDH Homeobox protein Hox-C4 (HOXC4)
DH_9467  Porcellio scaber                  PKRQRTANTRHQILELEKEFHFNRYLTRRRRIEIAHSLCLSXXQIKIWFQNRR------- Deformed protein (Fragment) (Dfd)
DH_9507  Thermobia domestica               PKRQRTAYTRHQILELEKEFHFNRYLTRRRRIEIAHSLCLSERQIKIWFQNRR------- Deformed protein (Fragment) (dfd)
DH_11380 Daphnia pulex                     PKRQRTAYTRHQILELEKEFHFNRYLTRRRRIEIAHSLCLSERQIKIWFQNRRMKWKKDN YAS_Hox_Dfd1|Dappu1
DH_11415 Daphnia pulex                     PKRQRTAYTRHQILELEKEFHFNRYLTRRRRIEIAHSLCLSERQIKIWFQNRRMKWKKDN e_gw1.7.430.1|Dappu1
DH_4605  Archegozetes longisetosus         PKRQRTAYTRHQILELEKEFHFNRYLTRRRRIEIAHSLCLSERQIKIWFQNRRMKWKKDN Deformed (Fragment) (Dfd)
DH_4758  Artemia sanfranciscana            PKRQRTAYTRHQILELEKEFHFNRYLTRRRRIEIAHSLCLSERQIKIWFQNRRMKWKKDN Dfd protein (Fragment) (Dfd)
DH_5812  Artemia urmiana                   PKRQRTAYTRHQILELEKEFHFNRYLTRRRRIEIAHSLCLSERQIKIWFQNRRMKWKKDN Deformed (Fragment) (Dfd)
DH_5813  Artemia sp. Qi Xiang Cuo          PKRQRTAYTRHQILELEKEFHFNRYLTRRRRIEIAHSLCLSERQIKIWFQNRRMKWKKDN Deformed (Fragment) (Dfd)
DH_5814  Artemia sinica                    PKRQRTAYTRHQILELEKEFHFNRYLTRRRRIEIAHSLCLSERQIKIWFQNRRMKWKKDN Deformed (Fragment) (Dfd)
DH_5815  Artemia sp. MaDuo                 PKRQRTAYTRHQILELEKEFHFNRYLTRRRRIEIAHSLCLSERQIKIWFQNRRMKWKKDN Deformed (Fragment) (Dfd)
DH_5817  Artemia sanfranciscana            PKRQRTAYTRHQILELEKEFHFNRYLTRRRRIEIAHSLCLSERQIKIWFQNRRMKWKKDN Deformed (Fragment) (Dfd)
DH_5818  Artemia sp. BaiYanNao             PKRQRTAYTRHQILELEKEFHFNRYLTRRRRIEIAHSLCLSERQIKIWFQNRRMKWKKDN Deformed (Fragment) (Dfd)
DH_5816  Artemia sp. Kazakhstan HL-2005    PKRQRTAYTRHQILELEKEFHFNRYLTRRRRIEIAHSLCLSERQIKIWFQNRRMKWKKDN Deformed (Fragment) (Dfd)
DH_5819  Artemia persimilis                PKRQRTAYTRHQILELEKEFHFNRYLTRRRRIEIAHSLCLSERQIKIWFQNRRMKWKKDN Deformed (Fragment) (Dfd)
DH_1702  Cupiennius salei                  PKRQRTAYTRHQILELEKEFHFNRYLTRRRRIEIAHALCLSERQIKIWFQNRRMKWKKDN Deformed-2 (Fragment) (dfd-2)
DH_9294  Achaearanea tepidariorum          PKRQRTAYTRHQILELEKEFHFNRYLTRRRRIEIAHALCLSERQIKIWFQNRR------- Deformed-2 (Fragment) (dfd-2)
DH_4592  Cupiennius salei                  PKRQRTAYTRHQILELEKEFHFNRYLTRRRRIEIAHALCLSERQIKIWFQNRRMKWKKDN Homeobox protein (Fragment) (deformed)
DH_4684  Sacculina carcini                 PKRQRTAYTRHQILELEKEFHFNRYLTRRRRIEIAHSLCLTERQIKI------------- Deformed (Fragment) (Dfd)
```

```
DH_9632  Priapulus caudatus                        ------AYTRHQVLELEKEFHFNRYLTRRRRIEIAHSLCLTERQIKI------------- Deformed homeodomain protein (Fragment) (Dfd)
DH_9462  Chaetopterus variopedatus                 SKRTRTAYTRHQVLELEKEFHFNRYLTRRRRIEIAHSLCLSERQIKI------------- Homeoprotein CH-Hox4 (Fragment) (Hox4)
DH_9257  Pachymerium ferrugineum                   PKRQRTAYTRQQILELEKEFHFNRYLTRRRRIEIAHSLCLAERQIKIW------------ Putative Deformed (Fragment) (dfd)
DH_9260  Pachymerium ferrugineum                   AKRQRTAYTRQQILELEKEFHFNRYLTRRRRIEIAHSLCLSERQIKIW------------ Putative Deformed (Fragment) (dfd)
DH_8987  Lithobius forficatus                      PKRQRTAYTRQQILELEKEFHFNRYLTRRRRIEIAHSLCLSERQIKIWFQNRRMKWKKDN Deformed (Fragment) (Dfd)
DH_3575  Drosophila sechellia                      PKRQRTAYTRHQILELEKEFHYNRYLTRRRRIEIAHTLVLSERQIKIWFQNRRMKWKKDN GM10914 (GM10914)
DH_7791  Tribolium castaneum                       PKRQRTAYTRHQILELEKEFHYNRYLTRRRRIEIAHTLVLSERQIKIWFQN---------- TC Deformed protein (Fragment) (TC Deformed)
DH_4232  Drosophila simulans                       PKRQRTAYTRHQILELEKEFHYNRYLTRRRRIEIAHTLVLSERQIKIWFQNRRMKWKKDN GD19895 (GD19895)
DH_104   Drosophila melanogaster                   PKRQRTAYTRHQILELEKEFHYNRYLTRRRRIEIAHTLVLSERQIKIWFQNRRMKWKKDN Homeotic protein deformed (Dfd)
DH_3171  Drosophila erecta                         PKRQRTAYTRHQILELEKEFHYNRYLTRRRRIEIAHTLVLSERQIKIWFQNRRMKWKKDN GG13676 (GG13676)
DH_4144  Drosophila yakuba                         PKRQRTAYTRHQILELEKEFHYNRYLTRRRRIEIAHTLVLSERQIKIWFQNRRMKWKKDN GE24883 (GE24883)
DH_3000  Drosophila ananassae                      PKRQRTAYTRHQILELEKEFHYNRYLTRRRRIEIAHTLVLSERQIKIWFQNRRMKWKKDN GF17792 (GF17792)
DH_3461  Drosophila persimilis                     PKRQRTAYTRHQILELEKEFHYNRYLTRRRRIEIAHTLVLSERQIKIWFQNRRMKWKKDN GL22041 (GL22041)
DH_5757  Drosophila pseudoobscura pseudoobscura    PKRQRTAYTRHQILELEKEFHYNRYLTRRRRIEIAHTLVLSERQIKIWFQNRRMKWKKDN Dfd (Dfd)
DH_3681  Drosophila grimshawi                      PKRQRTAYTRHQILELEKEFHYNRYLTRRRRIEIAHTLVLSERQIKIWFQNRRMKWKKDN GH19479 (GH19479)
DH_3748  Drosophila mojavensis                     PKRQRTAYTRHQILELEKEFHYNRYLTRRRRIEIAHTLVLSERQIKIWFQNRRMKWKKDN GI24717 (GI24717)
DH_3900  Drosophila virilis                        PKRQRTAYTRHQILELEKEFHYNRYLTRRRRIEIAHTLVLSERQIKIWFQNRRMKWKKDN GJ23932 (GJ23932)
DH_4015  Drosophila willistoni                     PKRQRTAYTRHQILELEKEFHYNRYLTRRRRIEIAHTLVLSERQIKIWFQNRRMKWKKDN GK12253 (GK12253)
DH_2098  Anopheles gambiae                         PKRQRTAYTRHQILELEKEFHYNRYLTRRRRIEIAHTLVLSERQIKIWFQNRRMKWKKDN AGAP004646-PA (AGAP004646)
DH_5270  Aedes aegypti                             PKRQRTAYTRHQILELEKEFHYNRYLTRRRRIEIAHTLVLSERQIKIWFQNRRMKWKKDN Homeotic deformed protein, putative (AAEL009950)
DH_6074  Mamestra brassicae                        PKRQRTAYTRHQILELEKEFHYNRYLTRRRRIEIAHTLVLSERQIKIWFQNRRMKWKKDN Deformed (dfd)
DH_4742  Tribolium castaneum                       PKRQRTAYTRHQILELEKEFHYNRYLTRRRRIEIAHTLVLSERQIKIWFQNRRMKWKKDN Transcription factor Deformed (Dfd) (dfd)
DH_2585  Culex quinquefasciatus                    PKRQRTAYTRHQILELEKEFHYNRYLTRRRRIEIAHTLVLSERQIKIWFQNRRMKWKKDN Homeotic deformed protein (CpipJ_CPIJ001004)
DH_9614  Bombyx mori                               PKRQRTGYTRHQILELEKEFHYNRYLTRRRRIEIAHTLVLSERQIKIWFQNRRMKWKKDN Dfd protein (Dfd)
DH_9245  Anopheles gambiae                         PKRQRTAYTRHQILELEKEFHYNXYLTRRRRIEIAHTLVLSERQIKIWFQNRRMKWKKD- Transcription factor Deformed (Fragment) (Dfd)
DH_7106  Oncopeltus fasciatus                      PKRQRTAYTRHQILELEKEFHYNKYLTRRRRIEIAHTLCLSERQIKIWFQNRRMKWKKD- Deformed (Fragment) (Dfd)
DH_11989 Capitella sp. I Grassle and Grassle, 1976 SKRTRTAYTRHQILELEKEFHFNRYLTRRRRIEIAHTLCLSERQIKIWFQNRRMKWKKEH estExt_Genewise1.C_700045|Capca1
DH_9060  Haliotis asinina                          ----------------------NRYLTRRRRIEIAHTLCLSERQIKIWFQNRRMKWKKEH Homeobox protein Hox4 (Fragment) (Hox4)
DH_12201 Lottia gigantea                           SKRNRTAYTRHQVLELEKEFHFNRYLTRRRRIEIAHTLCLSERQIKIWFQNRRMKWKKEH e_gw1.12.439.1|Lotgi1
DH_8645  Lithobius atkinsoni                       --------------------HFNRYLTRRRRIEIAHSLCLSERQIKIWFQNRRMRWKKEH Sex combs reduced (Fragment) (Scr)
DH_396   Lineus sanguineus                         SKRSRTAYTRHQILELEKEFHFNRYLTRRRRIEIAHALDLSERQIKIWFQNRRMKWKKEH Homeobox protein Hox-A4 (Fragment) (HOXA4)
DH_7446  Bugula turrita                            PKRARTAYTRHQILELEKEFHFNRYLTRRRRIEIAHTLDLSERQIKIWFQNRRMKWKKEH Hox class homeodomain-containing protein Dfda (Fragment)
DH_743   Caenorhabditis elegans                    EKRQRTAYTRNQVLELEKEFHTHKYLTRKRRIEVAHSLMLTERQVKIWFQNRRMKHKKEN Homeobox protein lin-39 (lin-39)
DH_9199  Caenorhabditis briggsae                   EKRQRTAYTRNQVLELEKEFHTHKYLTRKRRIEVAHSLMLTERQVKIWFQNRRMKHKKEN LIN-39 (CBR-LIN-39 protein) (Cbr-lin-39)
DH_4531  Pristionchus pacificus                    EKRQRTAYTRNQVLELEKEFHFNKYLTRKRRIEISHSLMLSERQVKIWFQNRRMKHKKEH Homeotic protein (lin-39)
DH_4729  Dugesia tigrina                           SKRNRTAYTRQQILELEKEFHFNKYLTRKRRNEIAQSLQLSESQVKIWFQNRRMKWKKDH Homeodomain protein (Fragment) (DthoxA)
DH_9593  Dugesia japonica                          SKRNRTAYTRQQILELEKEFHFNKYLTRKRRIEIAQSLQLSERQVKIWFQNRRMKWKKDH PLOX3-Dj (Fragment) (Plox3-Dj)
DH_9139  Discocelis tigrina                        NKRARTAYSAQQILELEKEFHFNKYLTRRRRIEIAQALFLTERQVKIWFQNRRMKWKKEH Hox4/Dfd protein (Fragment) (hoxD)
DH_6676  Oikopleura dioica                         NKRIRTAYTRHQTFELEKEFHFKRYLTRKRRIEIATTLQLSERQVKIWFQNRRMKWKKEH Hox4 (Fragment) (Hox4)
DH_7690  Schistosoma mansoni                       PKRTRTAYTRQQILELEKEFHYNKYLTRKRRLEIAHTLTLSERQIKIWFQNRRMKWKKEH Hox protein Dfd
DH_7521  Lampetra japonica                         LKRSRTAYTRQQVLELEKEFHFNRYLTRRRRIEIAHALCLTERQIKIWFQNRRMKWKKDH LjHox4w Homeobox (Fragment) (LjHox4w)
DH_8604  Petromyzon marinus                        LKRSRTAYTRQQVLELEKEFHFNRYLTRRRRIEIAHALCLTERQIKIWFQNRRMKWKKDH Hox4w (Fragment) (hox4w)
DH_2759  Flaccisagitta enflata                     PKRARTAYTRHQVLELEKEFHFNRYLTRRRRIEIAHALCLTERQIKIWFQNRRMKWKKDH Hox4 (Fragment)
DH_8507  Petromyzon marinus                        SKRSRTAYTRQQVLELEKEFHFNRYLTRRRRIEIAHSLCLSERQIKIWFQNRRMKWKKDH HoxG4 homeobox (Fragment)
DH_11537 Branchiostoma floridae                    TKRSRTAYTRQQVLELEKEFHFNRYLTRRRRIEIAHSLGLTERQIKIWFQNRRMKWKKDN estExt_fgenesh2_pg.C_4020005|Brafl1
DH_12680 Branchiostoma floridae                    TKRSRTAYTRQQVLELEKEFHFNRYLTRRRRIEIAHSLGLTERQIKIWFQNRRMKWKKDN AmphiHox4
DH_9514  Branchiostoma floridae                    TKRSRTAYTRQQVLELEKEFHFNRYLTRRRRIEIAHSLGLTERQIKIWFQNRRMKWKKDN AmphiHox4 protein (AmphiHox4)
DH_5290  Branchiostoma floridae                    TKRSRTAYTRQQVLELEKEFHFNRYLTRRRRIEIAHSLGLTERQIKIWFQNRRMKWKKDN Hox-4 homeodomain protein (Fragment)
DH_1325  Metacrinus rotundus                       AKRSRTSYTRQQILELEKEFHFNRYLTRRRRIEIAQALGLSERQIKIWFQNRRMKWKKDN Transcription factor Hox4 (MrHox4)
DH_7708  Patiriella exigua                         --------------------HFNRYLTRRRRIEIAQSLGLSERQIKIWFQNRRMKWKKDH Hox4 (Fragment) (Hox4)
DH_548   Rattus norvegicus                         GPARGVANCRQQVLELEKEFHYNRYLTRRRRIEIAHSLCLSERQIKIWFQNRRMKWKKDH Homeobox protein Hox-C4 (Fragment) (Hoxc4)
DH_6330  Tubifex tubifex                           --------------------HYNRYLTRRRRIEVAHQLSLSERQIKIWFQNRRMKWKKDY Homeodomain (Fragment) (Ttu-tox28)
DH_8652  Lithobius atkinsoni                       ---------RQQILELEKEFHFNRYLTRRRRIEIAHSLCLSERQIKIWFQNRRMKWKKDN Deformed (Fragment) (Dfd)
DH_1647  Carassius auratus                         -------------------FHFNHYLTRRRRIEIAHTLCLSERQIKIWFQNRRM------ HoxA5a protein (Fragment) (Hox)
DH_4599  Dugesia tigrina                           YKRCRQAYSRQQTLELEKEFYYNQYLTRRRRIEIANSVCLSERQIKIWFQNRRMKYKKDV Homeodomain protein (DthoxD)
DH_5552  Dugesia tigrina                           YKRCRQAYSRQQTLELEKEFYYNQYLTRRRRIEIANSVCLSERQIKIWFQNRRMKYKKDV DNA-Binding Protein (Fragment) (Homeobox)
DH_2766  Schmidtea mediterranea                    YKRSRQAYSRQQTLELEKEFYYNQYLTRRRRIEIANSVCLSERQIKIWFQNRRMKYKKDA HoxD-like protein (hoxD)
DH_9594  Dugesia japonica                          FKRSRQAYSRQQTLELEKEFYYNQYLTRRRRIEIANSVCLSERQIKIWFQNRRMKYKKDV PLOX4-Dj (Fragment) (Plox4-Dj)
DH_2588  Culex quinquefasciatus                    SKRTRQSYSRQQTLVLEKEFHMNKYLTRRRRIEIANILKLSERQVKIWFQNRRMKAKKDQ Segmentation protein fushi tarazu (CpipJ_CPIJ001007)
```

Save alignment [fasta format] - Save complete sequences [fasta format]

PG5

ID	organism	10 20 30 40 50 60	description
DH_11385	Daphnia pulex	TKRQRTSYTRYQTLELEKEFHFNRYLTRRRRIEIAHSLCLSERQIKIWFQNRRMKWKKEH	YAS_YAS_Hox_Scr|Dappu1
DH_8994	Folsomia candida	TKRQRTSYTRYQTLELEKEFHFNRYLTRRRRIEIAHSLCFSERQIKIWFQNRRMKWKKEH	Sex combs reduced (Fragment)
DH_11403	Daphnia pulex	TKRQRTSYTRYQTLELEKEFHFNRYLTRRRRIEIAHSLCLSERQIKIWFQNRRMKWKKEH	e_gw1.7.149.1|Dappu1
DH_8985	Lithobius forficatus	TKRQRTSYTRYQTLELEKEFHFNRYLTRRRRIEIAHSLCLSERQIKIWFQNRRMKWKKEH	Sex combs reduced 2 (Fragment) (Scr2)
DH_4609	Archegozetes longisetosus	TKRQRTSYTRYQTLXLEKEFHFNRYLTRRRRIEIAHSLCLSERQIKIWFQNRRMKWKKEH	Sex combs reduced (Fragment) (Scr)
DH_4760	Artemia sanfranciscana	TKRQRTSYTRYQTLELEKEFHFNRYLTRRRRIEIAHTLCLSERQIKIWFQNRRMKWKKEH	Scr protein (Fragment) (Scr)
DH_2469	Nymphon gracile	TKRQRTSYTRYQTLELEKEFHFNRYLTRRRRIEIAHALCLSERQIKIWFQNRRMKWKKEH	Homeobox protein sex comb reduced (Scr)
DH_5379	Endeis spinosa	TKRQRTSYTRYQTLELEKEFHFNRYLTRRRRIEIAHALCLSERQIKIWFQNRRMKWKKEH	Homeobox protein sex comb reduced (Fragment) (Scr)
DH_1700	Cupiennius salei	TKRQRTSYTRYQTLELEKEFHFNRYLTRRRRIEIAHALCLSERQIKIWFQNRRMKWKKEH	Sex comb reduced-1 (Fragment) (scr-1)
DH_1701	Cupiennius salei	TKRQRTSYTRYQTLELEKEFHFNRYLTRRRRIEIAHALCLSERQIKIWF-----------	Sex comb reduced-2 (Fragment) (scr-2)
DH_1262	Saccoglossus kowalevskii	AKRSRTAYTRYQTLELEKEFHFNRYLTRRRRIEIAHALGLSERQIKIWFQNRRMKWKKEH	Hox 5
DH_7670	Ptychodera flava	AKRSRTAYTRYQTLELEKEFHFNRYLTRRRRIEIAHALGLSERQIKIWFQNRRMKWKKEH	Transcription factor Hox5
DH_1326	Metacrinus rotundus	AKRSRTAYTRYQTLELEKEFHFNRYLTRRRRIEIAHALGLTERQIKIWFQNRRMKWKKEH	Transcription factor Hox5 (MrHox5)
DH_8868	Heliocidaris erythrogramma	SKRSRTAYTRYQTLELEKEFHFNRYLTRRRRIEIAHALGLTERQIKIWFQNRRMKWKKEH	HEHBOX9 (Fragment) (HeHbox9)
DH_12676	Strongylocentrotus purpuratus	SKRSRTAYTRYQTLELEKEFHFNRYLTRRRRIEIAHALGLTERQIKIWFQNRR-------	GLEAN3_05169|Sp-Hox5
DH_7671	Ptychodera flava	HKRTRTAYTRYQVLELEKEFHFNRYLTRRRRIEIAHALGLTERQVKIWFQNRRMKWKKDH	Transcription factor Hox4
DH_7928	Saccoglossus kowalevskii	HKRTRTAYTRYQVLELEKEFHFNRYLTRRRRIEIAHALGLTERQIKIWFQNRRMKWKKDH	Hox4
DH_8535	Oscheius tipulae	EKRQRTAYTRVQVLELEKEFHFNKYLTRKRRLEIAHALTLTERQVKIWFQNRRMKHKKEN	HOM-C transcription factor (lin-39)
DH_8536	Oscheius tipulae	EKRQRTAYTRVQVLELEKEFHFNKYLTRKRRLEIAHALTLTERQVKIWFQNRRMKHKKEN	HOM-C transcription factor (lin-39)
DH_9483	Nereis virens	SKRTRTAYTRHQVLELEKEFHFNRYLTRRRRIEIAHALCLTERQIKIWFQNRRMKWKKEN	Deformed homeobox protein (Fragment) (Dfd)
DH_6452	Tetraodon nigroviridis	PKRSRTAYTRQQVLELEKEFHYNRYLTRRRRIEIAHALVLSERQIKIWFQNRRMKWKKDH	Chromosome 9 SCAF14991, whole genome shotgun sequence (GSTENG00028038001)
DH_8995	Folsomia candida	TKRQRTAYTRHQILELEKEFHYNRYLTRRRRIEIAHSLVLSERQIKIWFQNRRMKYKKDN	Deformed (Fragment)
DH_8359	Ciona intestinalis	-KRPRTAYTRHQVLELEKEFHYNRYLTRRRRIEIALGLCLSERQVKIWFQNRRMKWKKDH	Putative homeobox protein 4 (hox4)
DH_7334	Oikopleura dioica	NKRIRTAYTRHQTLELEKEFHFNRYLTRKRRIEIATTLQLSERQVKIWFQNRRMKWKKEH	Hox4.1 (007-10)
DH_12358	Helobdella robusta	NKRTRTSYTRHQTLELEKEFHFNRYLSRRRRIEIAHVLNLSERQIKIWFQNRRMKWKKDH	e_gw1.90.8.1|Helro1
DH_4438	Helobdella triserialis	NKRTRTSYTRHQTLELEKEFHFNRYLSRRRRIEIAHVLNLSERQIKIWFQNRRMKWKKDH	LOX20 homeodomain protein (Fragment)
DH_7650	Perionyx excavatus	RKRKRTLYTRHQTLELEKEFHFNRYLVRRRRIEIAHYLNLSERQIKIWFQNRR-------	Homeobox protein Scr (Fragment)
DH_12317	Helobdella robusta	SKRLRTSYTRHQTLELEKEFYFNRYLARRRRIEIAHALSLSERQIKIWFQNRRMKWKKDH	e_gw1.63.157.1|Helro1
DH_7005	Urechis unicinctus	SKRTRTSYTRHQTLELEKEFHFNRYLTRRRRIEIAHLLNLSERQIKIWFQNRR-------	Homeobox protein Scr (Fragment)
DH_12320	Helobdella robusta	SKRTRTSYTRHQTLELEKEFHFNRYLTRRRRIEVAHMLNLTERQIKIWFQNRRMKWKKDY	gw2.22.102.1|Helro1
DH_9482	Nereis virens	SKRTRTSYTRHQTLELEKEFHFNRYLTRRRRIEIAHALNLTERQIKIWFQNRRMKWKKEH	Sex combs reduced homeobox protein (Fragment) (Scr)
DH_9493	Lingula unguis	SKRTRTSYTRHQTLELEKEFHFNRYLTRRRRIEIAHALNLTERQIKIWFQNRRMKWKKEQ	Sex combs reduced homeodomain protein (Fragment)
DH_11995	Capitella sp. I Grassle and Grassle, 1976	NKRTRTSYTRHQTLELEKEFHFNRYLTRRRRIEIAHSLNLTERQIKIWFQNRRMKWKKEH	estExt_Genewise1Plus.C_700047|Capca1
DH_9243	Haliotis rufescens	TKRSRTSYTRHQTLELEKEFHYNKYLTRRRRIEIAHALNLTERQIKIWFQNRRMKWKKEH	Hox5
DH_8632	Euprymna scolopes	SKRSRTSYTRHQTLELEKEFHYNKYLTRRRRIEIAHALNLTERQIKIWFQNRRMKWKKEH	Sec-combs reduced protein (Fragment) (Scr)
DH_12163	Lottia gigantea	SKRSRTSYTRHQTLELEKEFHYNKYLTRRRRIEIAHALNLTERQIKIWFQNRRMKWKKDH	gw1.12.429.1|Lotgi1
DH_5578	Hirudo medicinalis	NKRLRTSYSRQQTLELEKEFHFNRYLTKRRRVEIAQMLKLSERQIKIWFQNRRMKWKKEN	Lox1 protein (Fragment) (Lox1)
DH_8082	Diplosoma listerianum	NKRRRTSYSRYQTLELEKEFHYNRYLTRRRRVEIAHILSLSERQIKIWFQNRRMRWKKEN	Hox5 (Fragment)
DH_12319	Helobdella robusta	DKRARTSYSRYQTLELEKEFHFNRYLNGRRRIEIAHSLGLTERQIKIWFQNRRMKWKKDN	e_gw1.108.1.1|Helro1
DH_4405	Helobdella sp. MS-2000	DKRARTSYSRYQTLELEKEFHFNRYLNGRRRIEIAHSLGLTERQIKIWFQNRRMKWKKDN	LOX6 (Fragment) (Lox6)
DH_4444	Hirudo medicinalis	DKRARTSYSRYQTLELEKEFHFNRYLNGRRRIEIAHSLGLTERQIKIWFQNRRMKWKKDN	LOX6 (Fragment) (Lox6)
DH_7652	Perionyx excavatus	EKRARTSYSRYQTLELEKEFHFNRYLTGRRRLEIAHSLGLTERQIKIWFQNRR-------	Homeobox protein Lox6 (Fragment)
DH_7006	Urechis unicinctus	EKRSRTSYSRYQTLELEKEFHFNRYLTGRRRLEIAHTLGLTERQIKIWFQNRR-------	Homeobox protein Lox6 (Fragment)
DH_12480	Helobdella robusta	EKRLRTTYAKFQTFELEKEFHYNRYITRNKRIELSSRLELTQRQIKIWFQNRRMKFKKDS	gw2.9.567.1|Helro1
DH_9285	Anopheles gambiae	SKRTRQSYSRHQTIELEKEFHFNRYLNRRRRIEIASMLKLTERQIKIWFQNRRMKAKKDN	Homeobox transcription factor (Fragment) (ftz)
DH_11533	Branchiostoma floridae	NKRTRTAYTRYQTLELEKEFHFNRYLTRRRRIEIAHALCLTERQIKIWFQNRRMKWKKEN	e_gw.260.11.1|Brafl1
DH_12681	Branchiostoma floridae	NKRTRTAYTRYQTLELEKEFHFNRYLTRRRRIEIAHALCLTERQIKIWFQNRRMKWKKEN	AmphiHox5
DH_11625	Branchiostoma floridae	NKRTRTAYTRYQTLELEKEFHFNRYLTRRRRIEIAHALCLTERQIKIWFQNRRMKWKKEN	e_gw.260.11.1|Brafl1
DH_5291	Branchiostoma floridae	NKRTRTAYTRYQTLELEKEFHFNRYLTRRRRIEIAHALCLTERQIKIWFQNRRMKWKKEN	Hox-5 homeodomain protein (Fragment)
DH_4454	Ciona intestinalis	SKRTRTAYTRYQTLELEKEFHYNRYLTRRRRIEVAHTLCLTERQIKIWFQNRRMKWKKEN	Hox 5 (hox5)
DH_6276	Ciona intestinalis	SKRTRTAYTRYQTLELEKEFHYNRYLTRRRRIEVAHTLCLTERQIKIWFQNRRMKWKKEN	Transcription factor protein (Ci-Hox5)
DH_7962	Symsagittifera roscoffensis	CKRTRTAYTRFQTLELEKEFHFNRYLTRRRRIEIANLLALTERQIKIWFQNRRMKWKKDN	Homeobox protein SrHox4/5 (Fragment) (Hox4/5)
DH_1394	Pan paniscus	GKRARTAYTRYQTLELEKEFHFNRYLTRRRRIEIAHALCLSXRQIKIWFQNRRMKWKKDN	HOXB5 (Fragment) (HOXB5)
DH_7466	Homo sapiens	GKRARTAYTRYQTLELEKEFHFNHYLTRRRRIEIAHALCLSERQIKIWFQNRRMKWKKDN	HOXA5 protein (Fragment) (HOXA5)
DH_3310	Salmo salar	GKRARTAYTRYQTLELEKEFHFNRYLTRRRRIEIAHALCLSERQIKIWFQNRRMKWKKDN	Homeobox protein HoxB5aa (HoxB5aa)
DH_4745	Dicentrarchus labrax	GKRARTAYTRYQTLELEKEFHFNRYLTRRRRIEIAHALCLSERQIKIWFQNRRMKWKKDN	HOX-A5
DH_491	Gallus gallus	GKRARTAYTRYQTLELEKEFHFNRYLTRRRRIEIAHALCLSERQIKIWFQNRRMKWKKDN	Homeobox protein Hox-B5 (Fragment) (HOXB5)
DH_1360	Gorilla gorilla gorilla	GKRARTAYTRYQTLELEKEFHFNRYLTRRRRIEIAHALCLSERQIKIWFQNRRMKWKKDN	HOXA5 (Fragment) (HOXA5)
DH_416	Fugu rubripes	GKRARTAYTRYQTLELEKEFHFNRYLTRRRRIEIAHALCLSERQIKIWFQNRRMKWKKDN	Homeobox protein Hox-A5a (hoxa5a)

DH_3287	Salmo salar	GKRARTAYTRYQTLELEKEFHFNRYLTRRRRIEIAHALCLSERQIKIWFQNRRMKWKKDN	Homeobox protein HoxA5aa (HoxA5aa)
DH_7146	Xenopus tropicalis	GKRARTAYTRYQTLELEKEFHFNRYLTRRRRIEIAHALCLSERQIKIWFQNRRMKWKKDN	Homeobox A5 (hoxa5)
DH_3297	Salmo salar	GKRARTAYTRYQTLELEKEFHFNRYLTRRRRIEIAHALCLSERQIKIWFQNRRMKWKKDN	Homeobox protein HoxA5ab (HoxA5ab)
DH_404	Bos taurus	GKRARTAYTRYQTLELEKEFHFNRYLTRRRRIEIAHALCLSERQIKIWFQNRRMKWKKDN	Homeobox protein Hox-A5 (HOXA5)
DH_2576	Callithrix jacchus	GKRARTAYTRYQTLELEKEFHFNRYLTRRRRIEIAHALCLSERQIKIWFQNRRMKWKKDN	Homeobox A5 (Predicted) (HOXA5)
DH_409	Lemur catta	GKRARTAYTRYQTLELEKEFHFNRYLTRRRRIEIAHALCLSERQIKIWFQNRRMKWKKDN	Homeobox protein Hox-A5 (HOXA5)
DH_492	Homo sapiens	GKRARTAYTRYQTLELEKEFHFNRYLTRRRRIEIAHALCLSERQIKIWFQNRRMKWKKDN	Homeobox protein Hox-B5 (HOXB5)
DH_493	Mus musculus	GKRARTAYTRYQTLELEKEFHFNRYLTRRRRIEIAHALCLSERQIKIWFQNRRMKWKKDN	Homeobox protein Hox-B5 (Hoxb5)
DH_6813	Gallus gallus	GKRARTAYTRYQTLELEKEFHFNRYLTRRRRIEIAHALCLSERQIKIWFQNRRMKWKKDN	Hoxb-5 (Fragment) (Hoxb-5)
DH_1758	Xenopus tropicalis	GKRARTAYTRYQTLELEKEFHFNRYLTRRRRIEIAHALCLSERQIKIWFQNRRMKWKKDN	Hoxb5 protein (hoxb5)
DH_1377	Saguinus labiatus	GKRARTAYTRYQTLELEKEFHFNRYLTRRRRIEIAHALCLSERQIKIWFQNRR-------	HOXB5 (Fragment) (HOXB5)
DH_412	Rattus norvegicus	GKRARTCYTRYQTLELEKEFHFNRYLTRRRRIEIAHALCLSERQIKIWFQNRRMKWKKDK	Homeobox protein Hox-A5 (Fragment) (Hoxa5)
DH_2158	Haplochromis burtoni	GKRARTAYTRYQTLELEKEFHFNRYLTRRRRIEIAHALCLSERQIKIWFQNRRMKWKKDN	Hoxb5b (hoxb5b)
DH_11733	Gasterosteus aculeatus	GKRARTAYTRYQTLELEKEFHFNRYLTRRRRIEIAHALCLSERQIKIWFQNRRMKWKKDN	Homeobox protein Hox-B5 (Hox-2A) (HHO.C10) (HU-1) [Source:UniProtKB/Swiss-Prot;Acc:P09067]
DH_498	Danio rerio	GKRARTAYTRYQTLELEKEFHFNRYLTRRRRIEIAHALCLSERQIKIWFQNRRMKWKKDN	Homeobox protein Hox-B5b (hoxb5b)
DH_9387	Oryzias latipes	GKRARTAYTRYQTLELEKEFHFNRYLTRRRRIEIAHALCLSERQIKIWFQNRRMKWKKDN	HOXB5B (Fragment) (hoxb5b)
DH_410	Morone saxatilis	GKRARTAYTRYQTLELEKEFHFNRYLTRRRRIEIAHALCLSERQIKIWFQNRRMKWKKDN	Homeobox protein Hox-A5 (hoxa5)
DH_496	Danio rerio	GKRARTAYTRYQTLELEKEFHFNRYLTRRRRIEIAHALCLSERQIKIWFQNRRMKWKKDN	Homeobox protein Hox-B5a (hoxb5a)
DH_407	Homo sapiens	GKRARTAYTRYQTLELEKEFHFNRYLTRRRRIEIAHALCLSERQIKIWFQNRRMKWKKDN	Homeobox protein Hox-A5 (HOXA5)
DH_6186	Oryzias latipes	GKRARTAYTRYQTLELEKEFHFNRYLTRRRRIEIAHALCLSERQIKIWFQNRRMKWKKDN	HoxA5a (hoxA5a)
DH_6541	Tetraodon nigroviridis	GKRARTAYTRYQTLELEKEFHFNRYLTRRRRIEIAHALCLSERQIKIWFQNRRMKWKKDN	Chromosome 21 SCAF14577, whole genome shotgun sequence (GSTENG00017481001)
DH_405	Gallus gallus	GKRARTAYTRYQTLELEKEFHFNRYLTRRRRIEIAHALCLSERQIKIWFQNRRMKWKKDN	Homeobox protein Hox-A5 (HOXA5)
DH_3334	Salmo salar	GKRARTAYTRYQTLELEKEFHFNRYLTRRRRIEIAHALCLSERQIKIWFQNRRMKWKKDN	Homeobox protein HoxA5aa (HoxA5aa)
DH_1607	Pongo pygmaeus	GKRARTAYTRYQTLELEKEFHFNRYLTRRRRIEIAHALCLSERQIKIWFQNRRMKWKKDN	HOXA5 (Fragment) (HOXA5)
DH_408	Lagothrix lagotricha	GKRARTAYTRYQTLELEKEFHFNRYLTRRRRIEIAHALCLSERQIKIWFQNRRMKWKKDN	Homeobox protein Hox-A5 (HOXA5)
DH_411	Mus musculus	GKRARTAYTRYQTLELEKEFHFNRYLTRRRRIEIAHALCLSERQIKIWFQNRRMKWKKDN	Homeobox protein Hox-A5 (Hoxa5)
DH_1685	Homo sapiens	GKRARTAYTRYQTLELEKEFHFNRYLTRRRRIEIAHALCLSERQIKIWFQNRRMKWKKDN	Homeo box A5 (HOXA5)
DH_5960	Macaca fascicularis	GKRARTAYTRYQTLELEKEFHFNRYLTRRRRIEIAHALCLSERQIKIWFQNRRMKWKKDN	Putative uncharacterized protein
DH_1422	Mus musculus	GKRARTAYTRYQTLELEKEFHFNRYLTRRRRIEIAHALCLSERQIKIWFQNRRMKWKKDN	Homeo box B5 (Hoxb5)
DH_8015	Latimeria menadoensis	GKRARTAYTRYQTLELEKEFHFNRYLTRRRRIEIAHALCLSERQIKIWFQNRRMKWKKDN	HoxB5
DH_7104	Gallus gallus	GKRARTAYTRYQTLELEKEFHFNRYLTRRRRIEIAHALCLSERQIKIWFQNRRMKWKKDN	Homeodomain transcription factor (HOXB5)
DH_9080	Mus musculus	GKRARTAYTRYQTLELEKEFHFNRYLTRRRRIEIAHALCLSERQIKIWFQNRRMKWKKDN	Putative uncharacterized protein (Hoxb5)
DH_2842	Homo sapiens	GKRARTAYTRYQTLELEKEFHFNRYLTRRRRIEIAHALCLSERQIKIWFQNRRMKWKKDN	cDNA, FLJ95879, Homo sapiens homeo box B5 (HOXB5), mRNA
DH_3192	Sorex araneus	GKRARTAYTRYQTLELEKEFHFNRYLTRRRRIEIAHALCLSERQIKIWFQNRRMKWKKDN	Homeobox A5 (Predicted) (HOXA5)
DH_6116	Mus musculus	GKRARTAYTRYQTLELEKEFHFNRYLTRRRRIEIAHALCLSERQIKIWFQNRRMKWKKDN	Putative uncharacterized protein (Fragment) (Hoxa5)
DH_2751	Callicebus moloch	GKRARTAYTRYQTLELEKEFHFNRYLTRRRRIEIAHALCLSERQIKIWFQNRRMKWKKDN	Homeobox A5 (Predicted) (HOXA5)
DH_403	Ambystoma mexicanum	GKRARTAYTRYQTLELEKEFHFNRYLTRRRRIEIAHALCLSERQIKIWFQNRRMKWKKDN	Homeobox protein Hox-A5 (Fragment) (HOXA5)
DH_1510	Macaca mulatta	GKRARTAYTRYQTLELEKEFHFNRYLTRRRRIEIAHALCLSERQIKIWFQNRRMKWKKDN	HOXA5 (Fragment) (HOXA5)
DH_1580	Macaca nemestrina	GKRARTAYTRYQTLELEKEFHFNRYLTRRRRIEIAHALCLSERQIKIWFQNRRMKWKKDN	HOXA5 (Fragment) (HOXA5)
DH_2446	Papio anubis	GKRARTAYTRYQTLELEKEFHFNRYLTRRRRIEIAHALCLSERQIKIWFQNRRMKWKKDN	Homeobox A5 (Predicted) (HOXA5)
DH_11834	Gasterosteus aculeatus	GKRARTAYTRYQTLELEKEFHFNRYLTRRRRIEIAHALCLSERQIKIWFQNRRMKWKKDN	Homeobox protein Hox-A5 (Hox-1C) [Source:UniProtKB/Swiss-Prot;Acc:P20719]
DH_2152	Haplochromis burtoni	GKRARTAYTRYQTLELEKEFHFNRYLTRRRRIEIAHALCLSERQIKIWFQNRRMKWKKDN	Hoxa5a (hoxa5a)
DH_3351	Salmo salar	GKRARTAYTRYQTLELEKEFHFNRYLTRRRRIEIAHALCLSERQIKIWFQNRRMKWKKDN	Homeobox protien HoxB5ab (HoxB5ab)
DH_3348	Salmo salar	GKRARTAYTRYQTLELEKEFHFNRYLTRRRRIEIAHALCLSERQIKIWFQNRRMKWKKDN	Homeobox protein HoxB5aa (HoxB5aa)
DH_1538	Megalobrama amblycephala	GKRARTAYTRYQTLELEKEFHFNRYLTRRRRIEIAHALCLSERQIKIWFQNRRMKWKKDN	Homeodomain protein (Fragment) (HoxB5b)
DH_6715	Oreochromis niloticus	GKRARTAYTRYQTLELEKEFHFNRYLTRRRRIEIAHALCLSERQIKIWFQNRRMKWKKDN	Hox protein (Fragment)
DH_6164	Oryzias latipes	GKRARTAYTRYQTLELEKEFHFNRYLTRRRRIEIAHALCLSERQIKIWFQNRRMKWKKDN	HoxB5b (hoxB5b)
DH_7713	Sphoeroides nephelus	GKRARTAYTRYQTLELEKEFHFNRYLTRRRRIEIAHALCLSERQIKIWFQNRRMKWKKDN	Hoxb5b (Hoxb5b)
DH_499	Fugu rubripes	GKRARTAYTRYQTLELEKEFHFNRYLTRRRRIEIAHALCLSERQIKIWFQNRRMKWKKDN	Homeobox protein Hox-B5b (hoxb5b)
DH_9388	Oryzias latipes	--------------------HFNRYLTRRRRIEIAHALCLSERQIKIWFQNRRMKWKKDN	HOXA5A (Fragment) (hoxa5a)
DH_9279	Herdmania curvata	SKRTRTAYTRYQTLELEKEFHFNRYLTRRRRIEIAHALCLSERQIKIWFQNRRMKWKKDN	Hox5 (Hox5)
DH_3386	Paralabrax maculatofasciatus	-------------------EFHFNRYLTRRRRIEIAHALCLSERQIKIWFQNRRMKWKKDN	HOXa5 (Fragment)
DH_6808	Trionyx sinensis	GKRARTAYTRYRTLELEKEFHFNRYLTRRRRIEIAHALCLSERQIKIWFQNRRMKWKKDN	Hoxb-5 (Fragment) (Hoxb-5)
DH_414	Ovis aries	-----------QTLELEKEFHFNRYLTRRRRIEIAHALCLSERQIKIWFQNRRMKWKKDN	Homeobox protein Hox-A5 (Fragment) (HOXA5)
DH_497	Fugu rubripes	GKRARTAYTRYQTLELEKEFHFNRYLTRRRRIEIAHALCLTERQIKIWFQNRRMKWKKDN	Homeobox protein Hox-B5a (hoxb5a)
DH_6511	Tetraodon nigroviridis	GKRARTAYTRYQTLELEKEFHFNRYLTRRRRIEIAHALCLTERQIKIWFQNRRMKWKKDN	Chromosome undetermined SCAF14653, whole genome shotgun sequence (GSTENG00020455001)
DH_2130	Haplochromis burtoni	GKRARTAYTRYQTLELEKEFHFNRYLTRRRRIEIAHALCLTERQIKIWFQNRRMKWKKDN	Hoxb5a (hoxb5a)
DH_11765	Gasterosteus aculeatus	GKRARTAYTRYQTLELEKEFHFNRYLTRRRRIEIAHALCLTERQIKIWFQNRRMKWKKDN	groupXI
DH_6171	Oryzias latipes	GKRARTAYTRYQTLELEKEFHFNRYLTRRRRIEIAHALCLTERQIKIWFQNRRMKWKKDN	HoxB5a (hoxB5a)
DH_6846	Oreochromis niloticus	GKRARTAYTRYQTLELEKEFHFNRYLTRRRRIEIAHALCLTERQIKIWFQNRRMKWKKDN	Hox protein
DH_9390	Oryzias latipes	GKRARTAYTRYQTLELEKEFHFNRYLTRRRRIEIAHALCLTERQIKIWFQNRRMKWKKDN	HOXB5A (Fragment) (hoxb5a)
DH_406	Heterodontus francisci	GKRARTAYTRYQTLELEKEFHFNRYLTRRRRIEIAHALCLTERQIKIWFQNRRMKWKKDN	Homeobox protein Hox-A5 (HOXA5)
DH_7514	Lampetra japonica	GKRSRTAYTRYQTLELEKEFHFNRYLTRRRRIEIAHALCLTERQIKIWFQNRRMKWKKDN	LjHox5i Homeobox (Fragment) (LjHox5i)
DH_8503	Petromyzon marinus	GKRSRTAYTRYQTLELEKEFHFNRYLTRRRRIEIAHALCLTERQIKIWFQNRRMRWKKDN	HoxN5 homeobox (Fragment)
DH_630	Heterodontus francisci	GKRTRTAYTRYQTLELEKEFHYNRYLTRRRRIEIAHALCLTERQIKIWFQNRRMKWKKDN	Homeobox protein Hox-D5 (HOXD5)
DH_3356	Salmo salar	GKRARTAYTRYQTLELEKEFHFNRYLTRRRRIEIAHTLCLTERQIKIWFQNRRMKWKKDN	Homeobox protein HoxB5bb (HoxB5bb)
DH_6787	Oncorhynchus mykiss	GKRARTAYTRYQTLELEKEFHFNRYLTRRRRIEIAHTLCLTERQIKIWFQNRRMKWKKDN	HoxB5bi (Fragment) (Hox)
DH_3353	Salmo salar	GKRARTAYTRYQTLELEKEFHFNRYLTRRRRIEIAHTLCLTERQIKIWFQNRRMKWKKDN	Homeobox protein HoxB5ba (HoxB5ba)
DH_9579	Paralichthys olivaceus	GKRARTAYTRYQTLELEKEFHFNRYLTRRRRIEIAHTLCLTERQIKIWFQNRRMKWKKDN	Hoxb-5 (Fragment) (Hoxb-5)
DH_495	Xenopus laevis	GKRARTAYTRYQTLELEKEFHFNRYLTRRRRIEIAHTLCLSERQIKIWFQNRRMKWKKDN	Homeobox protein Hox-B5 (Fragment) (hoxb5)
DH_415	Danio rerio	GKRPRTAYTRYQTLELEKEFHFNRYLTRRRRIEIAHTLCLSERQIKIWFQNRRMKWKKDN	Homeobox protein Hox-A5a (hoxa5a)
DH_2918	Danio rerio	GKRPRTAYTRYQTLELEKEFHFNRYLTRRRRIEIAHTLCLSERQIKIWFQNRRMKWKKDN	Homeo box A5a (hoxa5a)

DH_1527	Megalobrama amblycephala	GKRPRTAYTRFQTLELEKEFHFNRYLTRRRRIEIAHTLCLSERQIKIWFQNRRMKWKKDN	Homeodomain protein (Fragment) (HoxA5a)
DH_4475	Ethmostigmus rubripes	--------------------HFNRYLTRRRRIEIAHSLCLSERQIKIWFQNRRMKWKKDN	Deformed (Fragment) (Dfd)
DH_5040	Glomeris marginata	--------------------HFNRYLTRRRRIEIAHSLCLSERQIKIWFQNRRMKWKKDN	Deformed (Fragment) (dfd)
DH_5701	Polyandrocarpa misakiensis	--------------------HFNRYLTRRRRIEIAQSLCLSERQIKIWFQNRRMKWKKDN	Homeodomain protein PmHox5 (Fragment) (PmHox5)
DH_7513	Lampetra japonica	GKRSRTAYTRYQTLELEKEFHFNRYLTRRRRIEVANALCLSERQIKIWFQNRRMKWKKDN	LjHox5w Homeobox (Fragment) (LjHox5w)
DH_9404	Petromyzon marinus	GKRSRTAYTRYQTLELEKEFHFNRYLTRRRRIEVANALCLSERQIKIWFQNRRMKWKKDN	Homeobox protein (Fragment) (hoxw5)
DH_8506	Petromyzon marinus	GKRSRTAYTRYQTLELEKEFHFNRYLTRRRRIEVANALCLSERQIKIWFQNRRMKWKKDN	HoxJ5 homeobox (Fragment)
DH_2143	Haplochromis burtoni	GKRSRTSYTRYQTLELEKEFHFNRYLSRRRRIEIAHTLCLNERQIKIWFQNRRMKWKKDS	Hoxc5a (hoxc5a)
DH_6160	Oryzias latipes	GKRSRTSYTRYQTLELEKEFHFNRYLSRRRRIEIAHTLCLNERQIKIWFQNRRMKWKKDS	HoxC5a (hoxC5a)
DH_6836	Oreochromis niloticus	GKRSRTSYTRYQTLELEKEFHFNRYLSRRRRIEIAHTLCLNERQIKIWFQNRRMKWKKDS	Hox protein (Fragment)
DH_11757	Gasterosteus aculeatus	GKRSRTSYTRYQTLELEKEFHFNRYLSRRRRVEIAHTLCLNERQIKIWFQNRRMKWKKDS	Homeobox protein Hox-C5 (Hox-3D) (CP11) [Source:UniProtKB/Swiss-Prot;Acc:Q00444]
DH_556	Fugu rubripes	GKRSRTSYTRYQTLELEKEFHFNRYLSRRRRIEIAHNLCLNERQIKIWFQNRRMKWKKDS	Homeobox protein Hox-C5a (hoxc5a)
DH_3259	Salmo salar	GKRSRTSYTRYQTLELEKEFHFNRYLTRRRRIEIAHTLCLNERQIKIWFQNRRMKWKKDS	Homeobox protein HoxC5ba (HoxC5ba)
DH_3266	Salmo salar	GKRSRTSYTRYQTLELEKEFHFNRYLTRRRRIEIAHTLCLNERQIKIWFQNRRMKWKKDS	Homeobox protein HoxC5bb (HoxC5bb)
DH_551	Homo sapiens	GKRSRTSYTRYQTLELEKEFHFNRYLTRRRRIEIANNLCLNERQIKIWFQNRRMKWKKDS	Homeobox protein Hox-C5 (HOXC5)
DH_5192	Homo sapiens	------------------EFHFNRYLTRRRRIEIANNLCLNERQIKIWFQNRRMKWNKDS	Homeodomain protein (50 AA) (Fragment)
DH_552	Mus musculus	GKRSRTSYTRYQTLELEKEFHFNRYLTRRRRIEIANNLCLNERQIKIWFQNRRMKWKKDS	Homeobox protein Hox-C5 (Hoxc5)
DH_1461	Danio rerio	GKRSRTSYTRYQTLELEKEFHFNRYLTRRRRIEIANNLCLNERQIKIWFQNRRMKWKKDS	Homeo box C5a (hoxc5a)
DH_2923	Danio rerio	GKRSRTSYTRYQTLELEKEFHFNRYLTRRRRIEIANNLCLNERQIKIWFQNRRMKWKKDS	Hoxc5a protein (hoxc5a)
DH_555	Danio rerio	GKRSRTSYTRYQTLELEKEFHFNRYLTRRRRIEIANNLCLNERQIKIWFQNRRMKWKKDS	Homeobox protein Hox-C5a (hoxc5a)
DH_553	Notophthalmus viridescens	GKRSRTSYTRYQTLELEKEFHFNRYLTRRRRIEIANNLCLNERQIKIWFQNRRMKWKKDS	Homeobox protein Hox-C5 (Fragment)
DH_8009	Latimeria menadoensis	GKRSRTSYTRYQTLELEKEFHFNRYLTRRRRIEIANNLCLNERQIKIWFQNRRMKWKKDS	HoxC5 (Fragment)
DH_554	Xenopus laevis	GKRSRTSYTRYQTLELEKEFHFNRYLTRRRRIEIANNLCLNERQIKIWFQNRRMKWKKDT	Homeobox protein Hox-C5 (Fragment) (hoxc5)
DH_4799	Xenopus laevis	GKRSRTSYTRYQTLELEKEFHFNRYLTRRRRIEIANNLCLNERQIKIWFQNRRMKWKKDT	Hoxc5-A protein (hoxc5)
DH_3240	Salmo salar	GKRSRTSYTRYQTLELEKEFHFNRYLARRRRIEIANTLCLNERQIKIWFQNRRMKWKKDS	Homeobox protein HoxC5aa (HoxC5aa)
DH_3249	Salmo salar	GKRSRTSYTRYQTLELEKDFHFNHYLTRRRRIELANSLCLNERQIKIWFQNRRMKWKKDS	Homeobox protein HoxC5ab (HoxC5ab)
DH_5380	Endeis spinosa	PKRHRTNYNKIQSLELEKEFHFNRYVTRRRRIEIAHALCLPERQIKIWFQNRRMRYKKDN	Homeobox protein deformed (Dfd)
DH_6828	Oreochromis niloticus	--------------------HFSRYLTRRRRVEVAHALCLFERQVKVWFQNRRMRWKKDN	Hox protein (Fragment)
DH_7512	Lampetra japonica	LKRARTAYTRYQALELEKEFHFNRYLTRRRRVEVAGALCLSERQIKIWFQNRRMKWKKDH	LjHox4x Homeobox (Fragment) (LjHox4x)
DH_12315	Helobdella robusta	NKRARTAYTKHQILELEKEFHFNRYLTRRRRIEIAHCLCLSERQIKIWFQNRRMKWKKEN	e_gw1.18.37.1|Helro1
DH_9470	Helobdella triserialis	NKRARTRYTKHQILELEKEFHFNRYLTRRRRIEIAHCLCLSERQIKIWFQNRRVKWKKEN	LOX18 homeodomain protein (Fragment) (Lox18)
DH_7651	Perionyx excavatus	SKRARTAYTKHQILELEKEFHFNRYLTRRRRIEIAHSLCLSERQIKIWFQNRR-------	Homeobox protein Lox18 (Fragment)
DH_8470	Sacculina carcini	XKRQRTSYTRYQTLELEKEFHFNRYLTRRRRIEIAHALCLTERQIKIWFQNRRMKWKKEH	Sex comb reduced (Fragment) (Scr)
DH_1086	Apis mellifera	VKRQRTSYTRYQTLELEKEFHFNRYLTRRRRIEIAHALCLTERQIKIWFQNRRMKWKKEH	Homeobox protein H55 (Fragment)
DH_4485	Acanthokara kaputensis	AKRQRTSYTRYQTLELEKEFHFNRYLTRRRRIEIAHALCLTERQIKIWFQNRRMKWKKEH	Sex combs reduced (Fragment) (Scr)
DH_4685	Sacculina carcini	TKRQRTSYTRYQTLELEKEFHFNRYLTRRRRIEIAHALCLTERQVKIWFQNRRMKWKKEH	Sex comb reduced (Fragment) (Scr)
DH_9613	Bombyx mori	TKRQRTSYTRYQTLELEKEFHFNRYLTRRRRIEIAHALCLTERQIKIWFQNRRMKWKKEH	Scr protein (Scr)
DH_8892	Tribolium castaneum	TKRQRTSYTRYQTLELEKEFHFNRYLTRRRRIEIAHALCLTERQIKIWFQNRRMKWKKEH	Cephalothorax (Cx)
DH_8904	Tribolium castaneum	TKRQRTSYTRYQTLELEKEFHFNRYLTRRRRIEIAHALCLTERQIKIWFQNRRMKWKKEH	Cephalothorax (Cx)
DH_9051	Tribolium castaneum	TKRQRTSYTRYQTLELEKEFHFNRYLTRRRRIEIAHALCLTERQIKIWFQNRRMKWKKEH	Scr (scr)
DH_9288	Tribolium castaneum	TKRQRTSYTRYQTLELEKEFHFNRYLTRRRRIEIAHALCLTERQIKIWFQNRRMKWKKEH	Sex combs reduced Scr (Cephalothorax) (Cx)
DH_4016	Drosophila willistoni	TKRQRTSYTRYQTLELEKEFHFNRYLTRRRRIEIAHALCLTERQIKIWFQNRRMKWKKEH	GK10826 (GK10826)
DH_3747	Drosophila mojavensis	TKRQRTSYTRYQTLELEKEFHFNRYLTRRRRIEIAHALCLTERQIKIWFQNRRMKWKKEH	GI24715 (GI24715)
DH_3680	Drosophila grimshawi	TKRQRTSYTRYQTLELEKEFHFNRYLTRRRRIEIAHALCLTERQIKIWFQNRRMKWKKEH	GH19477 (GH19477)
DH_5758	Drosophila pseudoobscura pseudoobscura	TKRQRTSYTRYQTLELEKEFHFNRYLTRRRRIEIAHALCLTERQIKIWFQNRRMKWKKEH	Scr (Scr)
DH_3460	Drosophila persimilis	TKRQRTSYTRYQTLELEKEFHFNRYLTRRRRIEIAHALCLTERQIKIWFQNRRMKWKKEH	GL22040 (GL22040)
DH_2999	Drosophila ananassae	TKRQRTSYTRYQTLELEKEFHFNRYLTRRRRIEIAHALCLTERQIKIWFQNRRMKWKKEH	GF17790 (GF17790)
DH_4143	Drosophila yakuba	TKRQRTSYTRYQTLELEKEFHFNRYLTRRRRIEIAHALCLTERQIKIWFQNRRMKWKKEH	GE25827 (GE25827)
DH_3576	Drosophila sechellia	TKRQRTSYTRYQTLELEKEFHFNRYLTRRRRIEIAHALCLTERQIKIWFQNRRMKWKKEH	GM10524 (GM10524)
DH_4233	Drosophila simulans	TKRQRTSYTRYQTLELEKEFHFNRYLTRRRRIEIAHALCLTERQIKIWFQNRRMKWKKEH	GD19518 (GD19518)
DH_3172	Drosophila erecta	TKRQRTSYTRYQTLELEKEFHFNRYLTRRRRIEIAHALCLTERQIKIWFQNRRMKWKKEH	GG10180 (GG10180)
DH_1769	Drosophila melanogaster	TKRQRTSYTRYQTLELEKEFHFNRYLTRRRRIEIAHALCLTERQIKIWFQNRRMKWKKEH	CG1030-PB, isoform B (CG1030-PC, isoform C) (Scr)
DH_1087	Drosophila melanogaster	TKRQRTSYTRYQTLELEKEFHFNRYLTRRRRIEIAHALCLTERQIKIWFQNRRMKWKKEH	Homeotic protein Sex combs reduced (Scr)
DH_5676	Schistocerca gregaria	TKRQRTSYTRYQTLELEKEFHFNRYLTRRRRIEIAHALCLTERQIKIWFQNRRMKWKKEH	Sex combs reduced homeodomain protein homologue (Fragment)
DH_9184	Tribolium castaneum	TKRQRTSYTRYQTLELEKEFHFNRYLTRRRRIEIAHALCLTERQIKIWFQNRRMKWKKEH	Cephalothorax (Fragment) (Cx)
DH_3899	Drosophila virilis	TKRQRTSYTRYQTLELEKEFHFNRYLTRRRRIEIAHALCLTERQIKIWFQNRRMKWKKEH	GJ23929 (GJ23929)
DH_5271	Aedes aegypti	TKRQRTSYTRYQTLELEKEFHFNRYLTRRRRIEIAHALCLTERQIKIWFQNRRMKWKKEH	Homeotic antennapedia protein, putative (AAEL009949)
DH_2587	Culex quinquefasciatus	TKRQRTSYTRYQTLELEKEFHFNRYLTRRRRIEIAHALCLTERQIKIWFQNRRMKWKKEH	Homeotic antennapedia protein (CpipJ_CPIJ001006)
DH_4589	Anopheles gambiae	TKRQRTSYTRYQTLELEKEFHFNRYLTRRRRIEIAHALCLTERQIKIWFQNRRMKWKKEH	Sex combs reduced homeotic protein (AGAP004659-PA) (Scr)
DH_8986	Lithobius forficatus	TKRQRTSYTRYQTLELEKEFHFNRYLTRRRRIEIAHALCLTERQIKIW------------	Sex combs reduced 1 (Fragment) (Scr1)
DH_8969	Procambarus clarkii	TKRQRTSYTRYQTLELEKEFHFNRYLTRRRRIEIAHALCLTERQIKIW------------	Scr protein (Fragment)
DH_4640	Trypetesa lampas	TKRQRTSYTRYQTLELEKEFHFNRYLTRRRRIEIAHALCLTERQIKI-------------	Homeobox sex comb reduce protein (Fragment) (Scr)
DH_9597	Porcellio scaber	TKRQRTSYTRYQTLELEKEFHFNRYLTRRRRIEIAHALCLTERQIKI-------------	Homeotic sex combs reduced protein (Fragment) (Scr)
DH_7804	Tribolium castaneum	TKRQRTSYTRYQTLELEKEFHFNRYLTRRRRIEIAHALCLTNKNLVSKPPHEVEKRAQDG	Cephalothorax (Cx)
DH_494	Ovis aries	GKRARTAYTRYQTLELEKEFHFNRYLTRRRRIEIAHA-----------------------	Homeobox protein Hox-B5 (Fragment) (HOXB5)
DH_9461	Chaetopterus variopedatus	NKRTRTSYTRHQTLELEKEFHFNRYLTRRRRIEIAHALNLTERQIKI-------------	Homeoprotein CH-Hox5 (Fragment) (Hox5)
DH_12388	Helobdella robusta	NKRTRTSYTRHQTLELEKEFHFNRYLNRRRRIEIAHNLILSERQVFRICRRRCCLCCCCC	e_gw1.231.3.1|Helro1
DH_9502	Thermobia domestica	TKRQRTSYTRYQTLELEKEFHFNRYLTRRRRIEIAHALCLTERQIKI-------------	Sex combs reduced protein (Fragment) (scr)

Save alignment [fasta format] - Save complete sequences [fasta format]

PG6

ID	organism	10 20 30 40 50 60	description
DH_7189	Thermobia domestica	---------RFQTLELEKEFHFNKYLTRRRRIEIAHSLGLSERQIKIWFQNRRMKAKKEI	Homeodomain transcription factor fushi tarazu (Fragment) (ftz)
DH_11648	Branchiostoma floridae	----------------XKEFHFNKYLTRRRRIEIAHALGLTERQIKIWFQNRRMKLKKEA	e_gw.260.61.1|Brafl1
DH_12345	Helobdella robusta	RKRGRQTYTRFQTLELEKEFHFNKYLTRRRRIEISHQLCLTERQVKIWFQNRRMKWKKEA	e_gw1.63.77.1|Helro1
DH_752	Caenorhabditis elegans	SKRTRQTYSRSQTLELEKEFHYHKYLTRKRRQEISETLHLTERQVKIWFQNRRMKHKKEA	Homeobox protein mab-5 (mab-5)
DH_9198	Caenorhabditis briggsae	SKRTRQTYSRSQTLELEKEFHYHKYLTRKRRQEISETLHLTERQVKIWFQNRRMKHKKEA	MAB-5 (CBR-MAB-5 protein) (Cbr-mab-5)
DH_2312	Caenorhabditis briggsae	SKRTRQTYSRSQTLELEKEFHYHKYLTRKRRQEISETLHLTERQVKIWFQNRRMKHKKEA	Putative uncharacterized protein (CBG00043)
DH_9609	Pristionchus pacificus	SKRTRQTYSRTQTLELEKEFHFNKYLTRKRRQEISESLHLTERQVKIWFQNRRMKHKKES	MAB-5
DH_4759	Artemia sanfranciscana	QKRTRQTYTKYQTLELEKEFLYNRYLTRVRRMDISSKLQLTERQIKIWFQNRRMKAKKEN	Hx1 protein (Fragment) (Hx1)
DH_5675	Schistocerca gregaria	AKRTRQTYTRFQTLELEKEFHYNRYLTRRRRVEISQALGLTERQIKIWFQNRRMKLKKEN	Divergent antennapedia-class homeodomain protein (Fragment)
DH_2476	Nymphon gracile	KKKGRQTYTRFQTLELEKEFRFNNYLTSRRRQDLSGMLELSERQIKIWFQNRRMKMKREI	Homeobox protein abdominalA (Fragment) (AbdA)
DH_8993	Folsomia candida	---TRQTYTRCQTLELEKEFHFNKYLTRRRRLDLAKMLTLSERQIKIWFQNRRMKAKKEV	Fushi tarazu (Fragment)
DH_2765	Flaccisagitta enflata	HRERRQTYTRHQTAELEREYVTNRYLTRRRRIEISQSLHLSERQIKIWFQNRRMKEKREK	MedPost (Fragment)
DH_8362	Spadella cephaloptera	HRKRRQTYSRQQTAELEREYVSNRYLTRRRRIEISQHLDLSERQIKIWFQNRRMKERREK	Hox protein MedPost (Fragment) (MedPost)
DH_8907	Haplophilus subterraneus	-------YTRFQTLELEKEFHFNRYLTRRRRIEIAHALGLTERQIKI-------------	Fushi tarazu (Fragment) (ftz)
DH_8905	Strigamia maritima	-------YTRFQTLELEKEFHFNRYLTRRRRIEIAHALGLTERQIKI-------------	Fushi tarazu (Fragment) (ftz)
DH_8909	Geophilus easoni	-------YTRFQTLELEKEFHFNRYLTRRRRIEIAHALGLTERQIKI-------------	Fushi tarazu (Fragment) (ftz)
DH_8910	Geophilus carpophagus	-------YTRFQTLELEKEFHFNRYLTRRRRIEIAHALGLTERQIKI-------------	Fushi tarazu (Fragment) (ftz)
DH_8906	Necrophloeophagus flavus	-------YTRFQTLELEKEFHFNRYLTRRRRIEIAHALGLTERQIKI-------------	Fushi tarazu (Fragment) (ftz)
DH_8908	Geophilus insculptus	-------YTRFQTLELEKEFHFNRYLTRRRRIEIAHTLGLTERQIKI-------------	Fushi tarazu (Fragment) (ftz)
DH_8911	Brachygeophilus truncorum	-------YTRFQTLELEKEFHFNRYLTRRRRIEIAHTLGLTERQIKI-------------	Fushi tarazu (Fragment) (ftz)
DH_4683	Sacculina carcini	AKRTRQSYTRYQTLELEKEFRTNRYLTRRRRIEIARTVALTERQIKI-------------	Diva (Fragment) (diva)
DH_8471	Sacculina carcini	AKRTRQSYTRYQTLELEKEFRTNRYLTRRRRIEIARTVALTERQIKIWFQNRRMKAKKEH	Diva
DH_8923	Tribolium castaneum	NKRTRQTYTRYQTLELEKEFHFNKYLTRRRRIEIAESLRLTERQIKIWFQNRRMKAKKDT	Homeodomain transcription factor Fushi tarazu (fushi tarazu)
DH_9052	Tribolium castaneum	NKRTRQTYTRYQTLELEKEFHFNKYLTRRRRIEIAESLRLTERQIKIWFQNRRMKAKKDT	Ftz (ftz)
DH_5694	Tribolium castaneum	NKRTRQTYTRYQTLELEKEFHFNKYLTRRRRIEIAESLRLTERQIKIWFQNRRMKAKKDT	Fushi-tarazu (ftz)
DH_8980	Lithobius forficatus	--------TRFQTLELEKVFHYNRYLTRRRRIEIAHTLVLSERQIKIWFQNRRMKLKKEM	Abdominal-A1 (Fragment) (AbdA1)
DH_11993	Capitella sp. I Grassle and Grassle, 1976	RRRGRQTYTRYQTLELEKEFKFNRYLTRRRRIELSHMLCLTERQIKIWFQNRRMKEKKEI	estExt_fgenesh1_pg.C_2920001|Capca1
DH_7003	Urechis unicinctus	RRRGRQTYTRYQTLELEKEFKFNRYLTRRRRIELSHMLCLTERQIKIWFQNRR-------	Homeobox protein Lox2 (Fragment)
DH_9481	Nereis virens	RRRGRQTYTRYQTLELEKEFKFNRYLTRKRRIELSHMLCLTERQIKIWFQNRRMKEKKEI	Lox2 homeobox protein (Fragment) (Lox2)
DH_12312	Helobdella robusta	RRRGRQTYTRYQTLELEKEFKFNRYLTRRRRIELSHTLYLTERQIKIWFQNRRMKEKKEV	e_gw1.22.27.1|Helro1
DH_221	Helobdella robusta	RRRGRQTYTRYQTLELEKEFKFNRYLTRRRRIELSHTLYLTERQIKIWFQNRRMKEKKEV	Homeobox protein LOX2 (Fragment) (LOX2)
DH_222	Hirudo medicinalis	RRRGRQTYTRYQTLELEKEFKFNRYLTRRRRIELSHTLYLTERQIKIWFQNRRMKEKKEV	Homeobox protein LOX2 (Fragment) (LOX2)
DH_7647	Perionyx excavatus	RRRGRQTYTRFQTLELEKEFKFNRYLTRRRRIELSHTLFLTERQIKIWFQNRR-------	Homeobox protein Lox2 (Fragment)
DH_12285	Lottia gigantea	RRRGRQTYTRFQTLELEKEFKFNRYLTRRRRIELSHMLCLTERQIKIWFQNRRMKEKKEL	fgenesh2_pg.C_sca_12000242|Lotgi1
DH_9628	Priapulus caudatus	RRRGRQTYTRYQTLELEKEFRFNHYLTRRRRIEMSQALCLTERQIKIWFQNRRMKLKKET	Ultrabithorax homeodomain protein (Fragment) (Ubx)
DH_8181	Tetraponera aethiops	--------TRYQTLELEKEFHTNHYLTRRRRIEMAHALCLTERQIKIWFQN---------	Ultrabithorax (Fragment) (Ubx)
DH_8182	Pseudomyrmex termitarius	--------TRYQTLELEKEFHTNHYLTRRRRIEMAHALCLTERQIKIWFQN---------	Ultrabithorax (Fragment) (Ubx)
DH_8184	Platythyrea sinuata	--------TRYQTLELEKEFHTNHYLTRRRRIEMAHALCLTERQIKIWFQN---------	Ultrabithorax (Fragment) (Ubx)
DH_8183	Ponera coarctata	--------TRYQTLELEKEFHTNHYLTRRRRIEMAHALCLTERQIKIWFQN---------	Ultrabithorax (Fragment) (Ubx)
DH_8180	Mutilla sp. HN-2002	--------TRYQTLELEKEFHTNHYLTRRRRIEMAHALCLTERQIKIWFQN---------	Ultrabithorax (Fragment) (Ubx)
DH_8185	Pachycondyla obscuricornis	--------TRYQTLELEKEFHTNHYLTRRRRIEMAHALCLTERQIKIWFQN---------	Ultrabithorax (Fragment) (Ubx)
DH_8179	Myrmecia tarsata	--------TRYQTLELEKEFHTNHYLTRRRRIEMAHALCLTERQIKIWFQN---------	Ultrabithorax (Fragment) (Ubx)
DH_8186	Odontomachus haematodus	--------TRYQTLELEKEFHTNHYLTRRRRIEMAHALCLTERQIKIWFQN---------	Ultrabithorax (Fragment) (Ubx)
DH_8187	Leptogenys sp. HN-2002	--------TRYQTLELEKEFHTNHYLTRRRRIEMAHALCLTERQIKIWFQN---------	Ultrabithorax (Fragment) (Ubx)
DH_8188	Gnamptogenys striatula	--------TRYQTLELEKEFHTNHYLTRRRRIEMAHALCLTERQIKIWFQN---------	Ultrabithorax (Fragment) (Ubx)
DH_8189	Ectatomma ruidum	--------TRYQTLELEKEFHTNHYLTRRRRIEMAHALCLTERQIKIWFQN---------	Ultrabithorax (Fragment) (Ubx)
DH_8190	Amblyopone australis	--------TRYQTLELEKEFHTNHYLTRRRRIEMAHALCLTERQIKIWFQN---------	Ultrabithorax (Fragment) (Ubx)
DH_8191	Tetramorium bicarinatum	--------TRYQTLELEKEFHTNHYLTRRRRIEMAHALCLTERQIKIWFQN---------	Ultrabithorax (Fragment) (Ubx)
DH_8192	Myrmica rubra	--------TRYQTLELEKEFHTNHYLTRRRRIEMAHALCLTERQIKIWFQN---------	Ultrabithorax (Fragment) (Ubx)
DH_8193	Messor capitatus	--------TRYQTLELEKEFHTNHYLTRRRRIEMAHALCLTERQIKIWFQN---------	Ultrabithorax (Fragment) (Ubx)
DH_8194	Manica rubida	--------TRYQTLELEKEFHTNHYLTRRRRIEMAHALCLTERQIKIWFQN---------	Ultrabithorax (Fragment) (Ubx)
DH_8195	Crematogaster limata parabiotica	--------TRYQTLELEKEFHTNHYLTRRRRIEMAHALCLTERQIKIWFQN---------	Ultrabithorax (Fragment) (Ubx)
DH_8196	Cyphomyrmex salvini	--------TRYQTLELEKEFHTNHYLTRRRRIEMAHALCLTERQIKIWFQN---------	Ultrabithorax (Fragment) (Ubx)
DH_8197	Crematogaster brasiliensis	--------TRYQTLELEKEFHTNHYLTRRRRIEMAHALCLTERQIKIWFQN---------	Ultrabithorax (Fragment) (Ubx)
DH_8198	Acromyrmex subterraneus	--------TRYQTLELEKEFHTNHYLTRRRRIEMAHALCLTERQIKIWFQN---------	Ultrabithorax (Fragment) (Ubx)
DH_8199	Polyrhachis laboriosa	--------TRYQTLELEKEFHTNHYLTRRRRIEMAHALCLTERQIKIWFQN---------	Ultrabithorax (Fragment) (Ubx)
DH_8200	Oecophylla longinoda	--------TRYQTLELEKEFHTNHYLTRRRRIEMAHALCLTERQIKIWFQN---------	Ultrabithorax (Fragment) (Ubx)
DH_8201	Lasius alienus	--------TRYQTLELEKEFHTNHYLTRRRRIEMAHALCLTERQIKIWFQN---------	Ultrabithorax (Fragment) (Ubx)
DH_8202	Formica selysi	--------TRYQTLELEKEFHTNHYLTRRRRIEMAHALCLTERQIKIWFQN---------	Ultrabithorax (Fragment) (Ubx)
DH_8203	Dendromyrmex sp. HN-2002	--------TRYQTLELEKEFHTNHYLTRRRRIEMAHALCLTERQIKIWFQN---------	Ultrabithorax (Fragment) (Ubx)
DH_8204	Camponotus vagus	--------TRYQTLELEKEFHTNHYLTRRRRIEMAHALCLTERQIKIWFQN---------	Ultrabithorax (Fragment) (Ubx)
DH_8205	Camponotus sp. HN-2002	--------TRYQTLELEKEFHTNHYLTRRRRIEMAHALCLTERQIKIWFQN---------	Ultrabithorax (Fragment) (Ubx)
DH_8206	Camponotus femoratus	--------TRYQTLELEKEFHTNHYLTRRRRIEMAHALCLTERQIKIWFQN---------	Ultrabithorax (Fragment) (Ubx)

```
DH_8207  Eciton burchelli                          --------TRYQTLELEKEFHTNHYLTRRRRIEMAHALCLTERQIKIWFQN--------- Ultrabithorax (Fragment) (Ubx)
DH_8208  Dorylus nigricans                         --------TRYQTLELEKEFHTNHYLTRRRRIEMAHALCLTERQIKIWFQN--------- Ultrabithorax (Fragment) (Ubx)
DH_8209  Tapinoma erraticum                        --------TRYQTLELEKEFHTNHYLTRRRRIEMAHALCLTERQIKIWFQN--------- Ultrabithorax (Fragment) (Ubx)
DH_8210  Dolichoderus sp. HN-2002                  --------TRYQTLELEKEFHTNHYLTRRRRIEMAHALCLTERQIKIWFQN--------- Ultrabithorax (Fragment) (Ubx)
DH_8211  Dolichoderus bidens                       --------TRYQTLELEKEFHTNHYLTRRRRIEMAHALCLTERQIKIWFQN--------- Ultrabithorax (Fragment) (Ubx)
DH_8212  Azteca sp. HN-2002                        --------TRYQTLELEKEFHTNHYLTRRRRIEMAHALCLTERQIKIWFQN--------- Ultrabithorax (Fragment) (Ubx)
DH_8213  Cerapachys sp. HN-2002                    --------TRYQTLELEKEFHTNHYLTRRRRIEMAHALCLTERQIKIWFQN--------- Ultrabithorax (Fragment) (Ubx)
DH_5205  Porcellio scaber                          RRRGRQTYTRYQTLELEKEFHTNHYLTRRRRIEMAHALCLTERQIKIWFQNRRMKLKKEI Ultrabithorax (Fragment) (Ubx)
DH_11382 Daphnia pulex                             RRRGRQTYTRYQTLELEKEFHTNHYLTRRRRIEMAHALCLTERQIKIWFQNRRMKLKKEI YAS_NCBI_GNO_0700054|Dappu1
DH_5210  Daphnia magna                             RRRGRQTYTRYQTLELEKEFHTNHYLTRRRRIEMAHALCLTERQIKIWFQNRRMKLKKEI Ultrabithorax (Ubx)
DH_5202  Procambarus clarkii                       RRRGRQTYTRYQTLELEKEFHTNHYLTRRRRIEMAHAPCLTERQIKIWFQNRRMKLKKEI Ultrabithorax (Fragment) (Ubx)
DH_9067  Porcellio scaber                          RRRGRQTYTRYQTLELEKEFHTNHYLTRRRRIEMAHALCLTERQIKIWFQNRRMKLKKEI Ubx1 (Fragment)
DH_11405 Daphnia pulex                             RRRGRQTYTRYQTLELEKEFHTNHYLTRRRRIEMAHALCLTERQIKIWFQNRRMKLKKEI gw1.7.150.1|Dappu1
DH_2590  Culex quinquefasciatus                    RRRGRQTYTRYQTLELEKEFHTNHYLTRRRRIEMAHALCLTERQIKIWFQNRRMKLKKEI Putative uncharacterized protein (CpipJ_CPIJ001011)
DH_4594  Cupiennius salei                          RRRGRQTYTRYQTLELEKEFHTNHYLTRRRRIEMAHALCLTERQIKIWFQNRRMKLKKEI Ultrabithorax-1 (Fragment) (ubx-1)
DH_1161  Drosophila melanogaster                   RRRGRQTYTRYQTLELEKEFHTNHYLTRRRRIEMAHALCLTERQIKIWFQNRRMKLKKEI Homeotic protein ultrabithorax (Ubx)
DH_1163  Drosophila simulans                       RRRGRQTYTRYQTLELEKEFHTNHYLTRRRRIEMAHALCLTERQIKIWFQNRRMKLKKEI Homeotic protein ultrabithorax (Ubx)
DH_3155  Drosophila erecta                         RRRGRQTYTRYQTLELEKEFHTNHYLTRRRRIEMAHALCLTERQIKIWFQNRRMKLKKEI GG16812 (GG16812)
DH_4125  Drosophila yakuba                         RRRGRQTYTRYQTLELEKEFHTNHYLTRRRRIEMAHALCLTERQIKIWFQNRRMKLKKEI GE26131 (GE26131)
DH_3008  Drosophila ananassae                      RRRGRQTYTRYQTLELEKEFHTNHYLTRRRRIEMAHALCLTERQIKIWFQNRRMKLKKEI GF17924 (GF17924)
DH_3429  Drosophila persimilis                     RRRGRQTYTRYQTLELEKEFHTNHYLTRRRRIEMAHALCLTERQIKIWFQNRRMKLKKEI GL23004 (GL23004)
DH_4049  Drosophila willistoni                     RRRGRQTYTRYQTLELEKEFHTNHYLTRRRRIEMAHALCLTERQIKIWFQNRRMKLKKEI GK13855 (GK13855)
DH_3896  Drosophila virilis                        RRRGRQTYTRYQTLELEKEFHTNHYLTRRRRIEMAHALCLTERQIKIWFQNRRMKLKKEI Ultrabithorax (Dvir\Ubx)
DH_7692  Drosophila virilis                        RRRGRQTYTRYQTLELEKEFHTNHYLTRRRRIEMAHALCLTERQIKIWFQNRRMKLKKEI Ultrabithorax (Ubx)
DH_3677  Drosophila grimshawi                      RRRGRQTYTRYQTLELEKEFHTNHYLTRRRRIEMAHALCLTERQIKIWFQNRRMKLKKEI GH19471 (GH19471)
DH_3744  Drosophila mojavensis                     RRRGRQTYTRYQTLELEKEFHTNHYLTRRRRIEMAHALCLTERQIKIWFQNRRMKLKKEI GI24708 (GI24708)
DH_4224  Drosophila simulans                       RRRGRQTYTRYQTLELEKEFHTNHYLTRRRRIEMAHALCLTERQIKIWFQNRRMKLKKEI Ultrabithorax (Dsim\Ubx)
DH_9066  Porcellio scaber                          RRRGRQTYTRYQTLELEKEFHTNHYLTRRRRIEMAHALCLTERQIKIWFQNRRMKLKKEI Ubx (Fragment)
DH_2530  Bombyx mori                               RRRGRQTYTRYQTLELEKEFHTNHYLTRRRRIEMAHALCLTERQIKIWFQNRRMKLKKEI Homeobox protein (Ubx)
DH_2531  Bombyx mori                               RRRGRQTYTRYQTLELEKEFHTNHYLTRRRRIEMAHALCLTERQIKIWFQNRRMKLKKEI Homeobox protein (Ubx)
DH_1165  Junonia coenia                            RRRGRQTYTRYQTLELEKEFHTNHYLTRRRRIEMAHALCLTERQIKIWFQNRRMKLKKEI Homeotic protein ultrabithorax (Ubx)
DH_4735  Manduca sexta                             RRRGRQTYTRYQTLELEKEFHTNHYLTRRRRIEMAHALCLTERQIKIWFQNRRMKLKKEI UBX (Fragment) (Ubx)
DH_8590  Tribolium castaneum                       RRRGRQTYTRYQTLELEKEFHTNHYLTRRRRIEMAHALCLTERQIKIWFQNRRMKLKKEI Ultrabithorax (Ubx)
DH_9621  Tribolium castaneum                       RRRGRQTYTRYQTLELEKEFHTNHYLTRRRRIEMAHALCLTERQIKIWFQNRRMKLKKEI Ultrathorax (Fragment)
DH_4588  Anopheles gambiae                         RRRGRQTYTRYQTLELEKEFHTNHYLTRRRRIEMAHALCLTERQIKIWFQNRRMKLKKEI Ultrabithorax homeotic protein IVa (AGAP004661-PB) (Ubx)
DH_7825  Anopheles gambiae                         RRRGRQTYTRYQTLELEKEFHTNHYLTRRRRIEMAHALCLTERQIKIWFQNRRMKLKKEI AGAP004661-PA (AGAP004661)
DH_4587  Anopheles gambiae                         RRRGRQTYTRYQTLELEKEFHTNHYLTRRRRIEMAHALCLTERQIKIWFQNRRMKLKKEI Ultrabithorax homeotic protein IIa (Ubx)
DH_8991  Folsomia candida                          RRRGRQTYTRYQTLELEKEFHTNHYLTRRRRIEMAHALCLTERQIKIWFQNRRMKLKKEI Ultrabithorax (Fragment)
DH_4478  Ethmostigmus rubripes                     RRRGRQTYTRYQTLELEKEFHTNHYLTRRRRIEMAHALCLTERQIKIWFQNRRMKLKKEI Ultrabithorax (Fragment) (Ubx)
DH_5412  Strigamia maritima                        RRRGRQTYTRYQTLELEKEFHTNHYLTRRRRIEMAHALCLTERQIKIWFQNRRMKLKKEI Ultrabithorax (ubx)
DH_2473  Endeis spinosa                            RRRGRQTYTRYQTLELEKEFHTNHYLTRRRRIEMAHALCLTERQIKIWFQNRRMKLKKEI Homeobox protein ultrabithorax (Ubx)
DH_8972  Procambarus clarkii                       RRRGRQTYTRYQTLELEKEFHTNHYLTRRRRIEMAHALCLTERQIKIWFQNRRMKLKKEI Ubx protein (Fragment)
DH_3596  Drosophila sechellia                      RRRGRQTYTRYQTLELEKEFHTNHYLTRRRRIEMAHALCLTERQIKIWFQNRRMKLKKEI GM15411 (GM15411)
DH_8638  Porcellio scaber                          --------------------HTNHYLTRRRRIEMAHALCLTERQIKIWFQNRRMKLKKEI Ultrabithorax (Fragment) (Ubx)
DH_8919  Pachymerium ferrugineum                   ---------------------TNHYLTRRRRIEMAHALCLTERQIKIWFQNRRMKLKKEI Ultrabithorax (Fragment) (ubx)
DH_5036  Glomeris marginata                        --------------------HTNHYLTRRRRIEMAHALCLTERQIKIWFQNRRMKLKKEI Ultrabithorax (Fragment) (ubx)
DH_6968  Gryllus bimaculatus                       --------------------HTNHYLTRRRRIEMAHALCLTERQIKIWFQNRRMKLKKEI Ultrabithorax (Fragment) (Ubx)
DH_8639  Archispirostreptus gigas                  --------------------HTNHYLTRRRRIEMAHALCLTERQIKIWFQNRRMKLKKEI Ultrabithorax (Fragment) (Ubx)
DH_8640  Folsomia candida                          --------------------HTNHYLTRRRRIEMAHALCLTERQIKIWFQNRRMKLKKEI Ultrabithorax (Fragment) (Ubx)
DH_8644  Lithobius atkinsoni                       --------------------HTNHYLTRRRRIEMAHALCLTERQIKIWFQNRRMKLKKEI Ultrabithorax (Fragment) (Ubx)
DH_8641  Tigriopus californicus                    --------------------HTNHYLTRRRRIEMAHQLCLTERQIKIWFQNRRMKLKKEI Ultrabithorax (Fragment) (Ubx)
DH_5209  Moina macrocopa                           RRRGRQTYTRYQTLELEKEFHTNHYLTRRRRIEMAHSLCLTERQIKIWFQNRRMKLKKEI Ultrabithorax (Ubx)
DH_5206  Artemia sanfranciscana                    RRRGRQTYTRYQTLELEKEFHTNHYLTRRRRIEMAHSLCLTERQIKIWFQNRRMKLKKEI Ultrabithorax (Ubx)
DH_8642  Artemia sanfranciscana                    RRRGRQTYTRYQTLELEKEFHTNHYLTRRRRIEMAHSLCLTERQIKIWFQNRRMKLKKEI Ultrabithorax (Ubx)
DH_4761  Artemia sanfranciscana                    RRRGRQTYTRYQTLELEKEFHTNHYLTRRRRIEMAHSLCLTERQIKIWFQNRRMKLKKEI Ubx protein (Fragment) (Ubx)
DH_4293  Archegozetes longisetosus                 RRRGRQTYTRYQTLELEKEFHTNHYLTRRRRIEMAHSLCLTERQIKIWFQNRRMKLKKEI Ultrabithorax (Fragment)
DH_4595  Cupiennius salei                          RRRGRQTYTRYQTLELEKEFHTNHYLTRRRRIEMAHSLCLTERQIKIWFQNRRMKLKKEA Ultrabithorax-2 (Fragment) (ubx-2)
DH_4687  Sacculina carcini                         --RGRQTYTRYQTLELEKEFHANHYLTRRRRIEMAHQLCLTERQIKIWFQNRRMKLKKEI Ultrabithorax (Fragment) (Ubx)
DH_5529  Ctenodrilus serratus                      --------------------QFNHYLTRKRRIEIAHALCLTERQIKIWFQNRRMKLKKER Homeobox protein (Fragment) (CTsx-2)
DH_5655  Ctenodrilus serratus                      --------------------QFNHYLTRKRRIEIAHALCLTERQIKIWFQNRRMKLKKER CTsx-2 protein (Fragment) (CTsx-2)
DH_12209 Lottia gigantea                           RRRGRQTYSRYQTLELEKEFQFNHYLTRKRRIEIAHTLCLTERQIKIWFQNRRMKMKKER estExt_Genewise1.C_sca_120441|Lotgi1
DH_9496  Patella vulgata                           --------------------QFNHYLTRKRRIEIAHTLCLTERQIKIWFQNRRMKMKRER Lox4 homeodomain protein (Fragment)
DH_12006 Capitella sp. I Grassle and Grassle, 1976 RRRGRQTYSRYQTLELEKEFQFNHYLTRKRRIEIAHALCLTERQIKIWFQNRRMKLKKER e_gw1.70.60.1|Capca1
DH_9489  Lingula unguis                            RRRGRQTYSRFQTLELEKEFQFNHYLTRKRRIEVAHALCLTERQIKIWFQNRRMKLKKER Lox4 homeodomain protein (Fragment)
DH_7002  Urechis unicinctus                        RRRGRQTYSRYQTLELEKEFQFNRYLTRKRRIEIAHCLCLTERQIKIWFQNRR------- Homeobox protein Lox4 (Fragment)
DH_7646  Perionyx excavatus                        MRRGRQTYSRYQTLELEKEFQFNRYLTRKRRIEIAHCLCLTERQIKIWFQNRR------- Homeobox protein Lox4 (Fragment)
DH_12313 Helobdella robusta                        RRRGRQTYSRYQTLELEKEFQFNRYLTRKRRIEIAHCLCLTERQIKIWFQNRRMKVKKEK e_gw1.18.97.1|Helro1
DH_5667  Hirudo medicinalis                        RRRGRQTYSRYQTLELEKEFQFNRYLTRKRRIEIAHCLCLTERQIKIWFQNRRMKVKKEK Lox4 protein (Lox4)
DH_12502 Helobdella robusta                        RRRGRQTYSRYQTLELEKEFQFNRYLTRKRRIEIAHSLCLTERQIKIWFQNRRMKSKKEI fgenesh4_pg.C_scaffold_108000001|Helro1
DH_4731  Dugesia tigrina                           KRRGRQTYSRHQTLELEKEFQFNHYLTRRRRIEIAHNLCLSERQIKIWFQNRRMKLKKER Homeodomain protein (DthoxF)
DH_5628  Polycelis nigra                           KRRGRQTYSRHQTLELEKEFQFNHYLTRRRRIEIAHNLCLSERQI--------------- Homeodomain protein (Fragment) (Pnox1 b)
DH_5627  Polycelis nigra                           KRRGRQTYSRHQTLELEKEFQFNHYLTRRRRIEIAHNLCLSERQIKIWFQNRRMKLKKER Homeodomain protein (Fragment) (Pnox1 a)
DH_1352  Taenia asiatica                           ------------ALELEKEFQYSHYLTRRRRIEIAHSLCLTERQIKIWFQN--------- Abdominal-A like protein Hox5 (Fragment)
DH_7689  Schistosoma mansoni                       RRRGRQTYSRYQTLELEKEFQYSHYLTRRRRIEIAHNLCLTERQIKIWFQNRRMKLKKER Abd-A-like protein
DH_8234  Ectatomma ruidum                          ---------RFQTLELEKEFHFNHYLTRRRRIEIAHALCLTERQIKIWFQNRRMKLKKEL Abdominal-A (Fragment) (abd-A)
DH_8233  Gnamptogenys striatula                    ---------RFQTLELEKEFHFNHYLTRRRRIEIAHALCLTERQIKIWFQNRRMKLKKEL Abdominal-A (Fragment) (abd-A)
```

DH_8246	Leptanilla sp. HN-2002	---------RFQTLELEKEFHFSHYLTRRRRIEIAHALCLTERQIKIWFQNRRMKLKKEL	Abdominal-A (Fragment) (abd-A)
DH_8217	Mutilla sp. HN-2002	---------RFQTLELEKEFHFNHYLTRRRRIEIAHALCLTERQIKIWFENRRMKLKKEL	Abdominal-A (Fragment) (abd-A)
DH_8252	Dendromyrmex sp. HN-2002	---------RFQTLELEKEFHFNHYLTRRRRIEIAHALCLTERQIKIWFQNRRMKLKKEL	Abdominal-A (Fragment) (abd-A)
DH_8229	Pachycondyla apicalis	---------RFQTLELEKEFHFNHYLTRRRRIEIAHALCLTERQIKIWFQNRRMKLKKEL	Abdominal-A (Fragment) (abd-A)
DH_8226	Pachycondyla obscuricornis	---------RFQTLELEKEFHFNHYLTRRRRIEIAHALCLTERQIKIWFQNRRMKLKKEL	Abdominal-A (Fragment) (abd-A)
DH_8222	Typhlomyrmex sp. HN-2002	---------RFQTLELEKEFHFNHYLTRRRRIEIAHALCLTERQIKIWFQNRRMKLKKEL	Abdominal-A (Fragment) (abd-A)
DH_8262	Dolichoderus bidens	---------RFQTLELEKEFHFNHYLTRRRRIEIAHALCLTERQIKIWFQNRRMKLKKEL	Abdominal-A (Fragment) (abd-A)
DH_8219	Pseudomyrmex termitarius	---------RFQTLELEKEFHFNHYLTRRRRIEIAHALCLTERQIKIWFQNRRMKLKKEL	Abdominal-A (Fragment) (abd-A)
DH_8253	Camponotus vagus	---------RFQTLELEKEFHFNHYLTRRRRIEIAHALCLTERQIKIWFQNRRMKLKKEL	Abdominal-A (Fragment) (abd-A)
DH_8244	Atta sexdens	---------RFQTLELEKEFHFNHYLTRRRRIEIAHALCLTERQIKIWFQNRRMKLKKEL	Abdominal-A (Fragment) (abd-A)
DH_8258	Dorylus nigricans	---------RFQTLELEKEFHFNHYLTRRRRIEIAHALCLTERQIKIWFQNRRMKLKKEL	Abdominal-A (Fragment) (abd-A)
DH_8255	Camponotus femoratus	---------RFQTLELEKEFHFNHYLTRRRRIEIAHALCLTERQIKIWFQNRRMKLKKEL	Abdominal-A (Fragment) (abd-A)
DH_8251	Formica selysi	---------RFQTLELEKEFHFNHYLTRRRRIEIAHALCLTERQIKIWFQNRRMKLKKEL	Abdominal-A (Fragment) (abd-A)
DH_8257	Eciton burchelli	---------RFQTLELEKEFHFNHYLTRRRRIEIAHALCLTERQIKIWFQNRRMKLKKEL	Abdominal-A (Fragment) (abd-A)
DH_8247	Polyrhachis laboriosa	---------RFQTLELEKEFHFNHYLTRRRRIEIAHALCLTERQIKIWFQNRRMKLKKEL	Abdominal-A (Fragment) (abd-A)
DH_8249	Lasius alienus	---------RFQTLELEKEFHFNHYLTRRRRIEIAHALCLTERQIKIWFQNRRMKLKKEL	Abdominal-A (Fragment) (abd-A)
DH_8242	Cyphomyrmex salvini	---------RFQTLELEKEFHFNHYLTRRRRIEIAHALCLTERQIKIWFQNRRMKLKKEL	Abdominal-A (Fragment) (abd-A)
DH_8245	Acromyrmex subterraneus	---------RFQTLELEKEFHFNHYLTRRRRIEIAHALCLTERQIKIWFQNRRMKLKKEL	Abdominal-A (Fragment) (abd-A)
DH_8239	Messor capitatus	---------RFQTLELEKEFHFNHYLTRRRRIEIAHALCLTERQIKIWFQNRRMKLKKEL	Abdominal-A (Fragment) (abd-A)
DH_8240	Manica rubida	---------RFQTLELEKEFHFNHYLTRRRRIEIAHALCLTERQIKIWFQNRRMKLKKEL	Abdominal-A (Fragment) (abd-A)
DH_8237	Tetramorium bicarinatum	---------RFQTLELEKEFHFNHYLTRRRRIEIAHALCLTERQIKIWFQNRRMKLKKEL	Abdominal-A (Fragment) (abd-A)
DH_8238	Myrmica rubra	---------RFQTLELEKEFHFNHYLTRRRRIEIAHALCLTERQIKIWFQNRRMKLKKEL	Abdominal-A (Fragment) (abd-A)
DH_8235	Ectatomma quadridens	---------RFQTLELEKEFHFNHYLTRRRRIEIAHALCLTERQIKIWFQNRRMKLKKEL	Abdominal-A (Fragment) (abd-A)
DH_8236	Amblyopone australis	---------RFQTLELEKEFHFNHYLTRRRRIEIAHALCLTERQIKIWFQNRRMKLKKEL	Abdominal-A (Fragment) (abd-A)
DH_8230	Odontomachus mayi	---------RFQTLELEKEFHFNHYLTRRRRIEIAHALCLTERQIKIWFQNRRMKLKKEL	Abdominal-A (Fragment) (abd-A)
DH_8231	Odontomachus haematodus	---------RFQTLELEKEFHFNHYLTRRRRIEIAHALCLTERQIKIWFQNRRMKLKKEL	Abdominal-A (Fragment) (abd-A)
DH_8227	Paraponera clavata	---------RFQTLELEKEFHFNHYLTRRRRIEIAHALCLTERQIKIWFQNRRMKLKKEL	Abdominal-A (Fragment) (abd-A)
DH_8228	Pachycondyla goeldii	---------RFQTLELEKEFHFNHYLTRRRRIEIAHALCLTERQIKIWFQNRRMKLKKEL	Abdominal-A (Fragment) (abd-A)
DH_8221	Myrcidris epicharis	---------RFQTLELEKEFHFNHYLTRRRRIEIAHALCLTERQIKIWFQNRRMKLKKEL	Abdominal-A (Fragment) (abd-A)
DH_8220	Pseudomyrmex gracilis	---------RFQTLELEKEFHFNHYLTRRRRIEIAHALCLTERQIKIWFQNRRMKLKKEL	Abdominal-A (Fragment) (abd-A)
DH_8214	Myrmecia tarsata	---------RFQTLELEKEFHFNHYLTRRRRIEIAHALCLTERQIKIWFQNRRMKLKKEL	Abdominal-A (Fragment) (abd-A)
DH_8218	Tetraponera aethiops	---------RFQTLELEKEFHFNHYLTRRRRIEIAHALCLTERQIKIWFQNRRMKLKKEL	Abdominal-A (Fragment) (abd-A)
DH_8243	Crematogaster brasiliensis	---------RFQTLELEKEFHFNHYLTRRRRIEIAHALCLTERQIKIWFQNRRMKLKKEL	Abdominal-A (Fragment) (abd-A)
DH_8241	Crematogaster limata parabiotica	---------RFQTLELEKEFHFNHYLTRRRRIEIAHALCLTERQIKIWFQNRRMKLKKEL	Abdominal-A (Fragment) (abd-A)
DH_8259	Tapinoma melanocephalum	---------RFQTLELEKEFHFNHYLTRRRRIEIAHALCLTERQIKIWFQNRRMKLKKEL	Abdominal-A (Fragment) (abd-A)
DH_8250	Gigantiops destructor	---------RFQTLELEKEFHFNHYLTRRRRIEIAHALCLTERQIKIWFQNRRMKLKKEL	Abdominal-A (Fragment) (abd-A)
DH_8261	Dolichoderus sp. HN-2002	---------RFQTLELEKEFHFNHYLTRRRRIEIAHALCLTERQIKIWFQNRRMKLKKEL	Abdominal-A (Fragment) (abd-A)
DH_8260	Tapinoma erraticum	---------RFQTLELEKEFHFNHYLTRRRRIEIAHALCLTERQIKIWFQNRRMKLKKEL	Abdominal-A (Fragment) (abd-A)
DH_8263	Azteca sp. HN-2002	---------RFQTLELEKEFHFNHYLTRRRRIEIAHALCLTERQIKIWFQNRRMKLKKEL	Abdominal-A (Fragment) (abd-A)
DH_8215	Myrmecia nigriceps	---------RFQTLELEKEFHFNHYLTRRRRIEIAHALCLTERQIKIWFQNRRMKLKKEL	Abdominal-A (Fragment) (abd-A)
DH_8225	Platythyrea sinuata	---------RFQTLELEKEFHFNHYLTRRRRIEIAHALCLTERQIKIWFQNRRMKLKKEL	Abdominal-A (Fragment) (abd-A)
DH_8232	Leptogenys sp. HN-2002	---------RFQTLELEKEFHFNHYLTRRRRIEIAHALCLTERQIKIWFQNRRMKLKKEL	Abdominal-A (Fragment) (abd-A)
DH_8248	Oecophylla longinoda	---------RFQTLELEKEFHFNHYLTRRRRIEIAHALCLTERQIKIWFQNRRMKLKKEL	Abdominal-A (Fragment) (abd-A)
DH_8254	Camponotus sp. HN-2002	---------RFQTLELEKEFHFNHYLTRRRRIEIAHALCLTERQIKIWFQNRRMKLKKEL	Abdominal-A (Fragment) (abd-A)
DH_8256	Neivamyrmex pilosus	---------RFQTLELEKEFHFNHYLTRRRRIEIAHALCLTERQIKIWFQNRRMKLKKEL	Abdominal-A (Fragment) (abd-A)
DH_8264	Cerapachys sp. HN-2002	---------RFQTLELEKEFHFNHYLTRRRRIEIAHALCLTERQIKIWFQNRRMKLKKEL	Abdominal-A (Fragment) (abd-A)
DH_8224	Ponera coarctata	---------RFQTLELEKEFHFNHYLTRRRRIEIAHALCLTERQIKIWFQNRRMKLKKEL	Abdominal-A (Fragment) (abd-A)
DH_8223	Prionopelta sp. HN-2002	---------RFQTLELEKEFHFNHYLTRRRRIEIAHALCLTERQIKIWFQNRRMKLKKEL	Abdominal-A (Fragment) (abd-A)
DH_3430	Drosophila persimilis	RRRGRQTYTRFQTLELEKEFHFNHYLTRRRRIEIAHALCLTERQIKIWFQNRRMKLKKEL	GL23002 (GL23002)
DH_4328	Drosophila pseudoobscura pseudoobscura	RRRGRQTYTRFQTLELEKEFHFNHYLTRRRRIEIAHALCLTERQIKIWFQNRRMKLKKEL	GA26453 (Dpse\GA26453)
DH_6662	Drosophila buzzatii	RRRGRQTYTRFQTLELEKEFHFNHYLTRRRRIEIAHALCLTERQIKIWFQNRRMKLKKEL	Dbuz\abd-A-PA (abd-A)
DH_6663	Drosophila buzzatii	RRRGRQTYTRFQTLELEKEFHFNHYLTRRRRIEIAHALCLTERQIKIWFQNRRMKLKKEL	Dbuz\abd-A-PB (abd-A)
DH_3768	Drosophila mojavensis	RRRGRQTYTRFQTLELEKEFHFNHYLTRRRRIEIAHALCLTERQIKIWFQNRRMKLKKEL	GI23753 (GI23753)
DH_3688	Drosophila grimshawi	RRRGRQTYTRFQTLELEKEFHFNHYLTRRRRIEIAHALCLTERQIKIWFQNRRMKLKKEL	GH18074 (GH18074)
DH_3922	Drosophila virilis	RRRGRQTYTRFQTLELEKEFHFNHYLTRRRRIEIAHALCLTERQIKIWFQNRRMKLKKEL	Abd-A (Dvir\abd-A)
DH_4048	Drosophila willistoni	RRRGRQTYTRFQTLELEKEFHFNHYLTRRRRIEIAHALCLTERQIKIWFQNRRMKLKKEL	GK13853 (GK13853)
DH_5	Drosophila melanogaster	RRRGRQTYTRFQTLELEKEFHFNHYLTRRRRIEIAHALCLTERQIKIWFQNRRMKLKKEL	Homeobox protein abdominal-A (abd-A)
DH_3594	Drosophila sechellia	RRRGRQTYTRFQTLELEKEFHFNHYLTRRRRIEIAHALCLTERQIKIWFQNRRMKLKKEL	GM15405 (GM15405)
DH_4124	Drosophila yakuba	RRRGRQTYTRFQTLELEKEFHFNHYLTRRRRIEIAHALCLTERQIKIWFQNRRMKLKKEL	GE26127 (GE26127)
DH_3154	Drosophila erecta	RRRGRQTYTRFQTLELEKEFHFNHYLTRRRRIEIAHALCLTERQIKIWFQNRRMKLKKEL	GG16810 (GG16810)
DH_4223	Drosophila simulans	RRRGRQTYTRFQTLELEKEFHFNHYLTRRRRIEIAHALCLTERQIKIWFQNRRMKLKKEL	GD20268 (GD20268)
DH_3007	Drosophila ananassae	RRRGRQTYTRFQTLELEKEFHFNHYLTRRRRIEIAHALCLTERQIKIWFQNRRMKLKKEL	GF17922 (GF17922)
DH_9059	Myrmica rubra	RRRGRQTYTRFQTLELEKEFHFNHYLTRRRRIEIAHALCLTERQIKIWFQNRRMKLKKEL	Abdominal-A protein (abd-A)
DH_2873	Bombyx mori	RRRGRQTYTRFQTLELEKEFHFNHYLTRRRRIEIAHALCLTERQIKIWFQNRRMKLKKEL	Abdominal A (abd-A)
DH_2533	Bombyx mori	RRRGRQTYTRFQTLELEKEFHFNHYLTRRRRIEIAHALCLTERQIKIWFQNRRMKLKKEL	Homeobox protein (abd-A)
DH_5035	Glomeris marginata	--------------------HFNHYLTRRRRIEIAHAFCLTERQIKIWFQNRRMKLKKEL	Abdominal-A (Fragment) (abd-A)
DH_5600	Junonia coenia	------------------EFHFNHYLTRRRRIEIAHALCLTERQIKIWFQNRRMKLKKEL	Abdominal-A homeodomain protein (Fragment) (abd-a)
DH_5811	Artemia sp. Qi Xiang Cuo	RRRGAQTYTRYQSLELEKEFHFNHYLTRRRRIEIAHALCLTERQIKIWFQNRRMKLKKEL	Abdominal-A (Fragment) (abdA)
DH_5832	Artemia sp. Kazakhstan HL-2005	RRRGRQTYTRYQSLELEKEFHFNHYLTRRRRIEIAHALCLTERQIKIWFQNRRMKLKKEL	Abdominal-A (Fragment) (abdA)
DH_8093	Zaprionus vittiger	------------------EFHFNHYLTRRRRIEIAHALCLTERQIKIWFQNRRM------	AbdA protein (Fragment) (abd-A)
DH_8094	Scaptodrosophila pattersoni	------------------EFHFNHYLTRRRRIEIAHALCLTERQIKIWFQNRRM------	AbdA protein (Fragment) (abd-A)
DH_8097	Chymomyza amoena	------------------EFHFNHYLTRRRRIEIAHALCLTERQIKIWFQNRRM------	AbdA protein (Fragment) (abd-A)
DH_8098	Drosophila adiastola	------------------EFHFNHYLTRRRRIEIAHALCLTERQIKIWFQNRRM------	AbdA protein (Fragment) (abd-A)
DH_8096	Drosophila heteroneura	------------------EFHFNHYLTRRRRIEIAHALCLTERQIKIWFQNRRM------	AbdA protein (Fragment) (abd-A)
DH_8095	Hirtodrosophila pictiventris	------------------EFHFNHYLTRRRRIEIAHALCLTERQIKICFQNRRM------	AbdA protein (Fragment) (abd-A)
DH_6	Manduca sexta	RRRGRQTYTRFQTLELEKEFHFNHYLTRRRRIEIAHALCLTERQIKIWFQNRRMKLKKEL	Homeobox protein abdominal-A homolog (Fragment) (ABD-A)

DH_2532	Bombyx mori	RRRGRQTYTRFQTLELEKEFHFNHYLTRRRRIEIAHALCLTERQIKIWFQNRRMKLKKEL	Homeobox protein (abd-A)
DH_8	Tribolium castaneum	RRRGRQTYTRFQTLELEKEFHFNHYLTRRRRIEIAHALCLTERQIKIWFQNRRMKLKKEL	Homeobox protein abdominal-A homolog (ABD-A)
DH_7	Schistocerca gregaria	RRRGRQTYTRFQTLELEKEFHFNHYLTRRRRIEIAHALCLTERQIKIWFQNRRMKLKKEL	Homeobox protein abdominal-A homolog (Fragment) (ABD-A)
DH_1	Anopheles gambiae	RRRGRQTYTRFQTLELEKEFHFNHYLTRRRRIEIAHALCLTERQIKIWFQNRRMKLKKEL	Homeobox protein abdominal-A homolog (abd-A)
DH_4	Culex quinquefasciatus	RRRGRQTYTRFQTLELEKEFHFNHYLTRRRRIEIAHALCLTERQIKIWFQNRRMKLKKEL	Homeobox protein abdominal-A homolog (abd-A)
DH_5248	Aedes aegypti	RRRGRQTYTRFQTLELEKEFHFNHYLTRRRRIEIAHALCLTERQIKIWFQNRRMKLKKEL	Putative uncharacterized protein (AAEL013832)
DH_8216	Polistes sp. HN-2002	---------RFQTLELEKEFHYNHYLTRRRRIEIAHALCLTERHIKIWFQNRRMKLKKEM	Abdominal-A (Fragment) (abd-A)
DH_2	Apis mellifera	RRRGRQTYTRFQTLELEKEFHYNHYLTRRRRIEIAHALCLTERQIKIWFQNRRMKLKKEL	Homeobox protein abdominal-A homolog (Fragment) (ABD-A)
DH_4596	Cupiennius salei	RRRGRQTYTRFQTLELEKEFHFNHYLTRRRRIEIAHALCLTERQIKIWFQNRRMKLKKEM	Homeobox protein (Fragment) (abdominal-A)
DH_9264	Pachymerium ferrugineum	--------TRFQTLELEKEFHFNHYLTRRRRIEIAHALCLTERQIKIW------------	Putative abdominal-A (Fragment) (abd-A)
DH_4473	Ethmostigmus rubripes	RRRGRQTYTRFQTLELEKEFHFNHYLTRRRRIEIAHALCLTERQIKIWFQNRRMKLKKEM	Abdominal-A (Fragment) (abd-A)
DH_5411	Strigamia maritima	RRRGRQTYTRFQTLELEKEFHFNHYLTRRRRIEIAHALCLTERQIKIWFQNRRMKLKKEM	Abdominal-A (abdA)
DH_8971	Procambarus clarkii	RRRGRQTYTRFQTLELEKEFHFNHYLTRRRRIEIAHALCLTERQVKIWFQNRRMKLKKEL	Abd-a protein (Fragment)
DH_9068	Porcellio scaber	RRRGRQTYTRFQTLELEKEFHFNHYLTRRRRIEIAHALCLTERQVKI-------------	Abd-A3 (Fragment)
DH_9069	Porcellio scaber	RRRGRQTYTRFQTLELEKEFHFNHYLTRRRRIEIAHALCLTERQVKIWFQNRRMKLKKEL	Abd-A2 (Fragment)
DH_9070	Porcellio scaber	RRRGRQTYTRFQTLELEKEFHFNHYLTRRRRIEIAHALCLTERQVKIWFQNRRMKLKKEL	Abd-A1 (Fragment)
DH_4295	Asellus aquaticus	-----QTYTRFQTLELEKEFHFNHYLTRRRRIEIAHALCLTERQVKIWFQNRRMKLKKEL	Abdominal-A (Fragment) (abd-A)
DH_5823	Artemia parthenogenetica	RRRGRQTYTRYQTLELEKEFHFNHYLTRRRRIEIAHAXCLTERQIKIWFQXRRMKLKKEL	Abdominal-A (Fragment) (abdA)
DH_5824	Artemia parthenogenetica	RRRGRQTYTRYQTLELEKEFHFNHYLTRRRRIEIAHAXCLTERQIKIWFQXRRMKLKKEL	Abdominal-A (Fragment) (abdA)
DH_8990	Folsomia candida	RRRGRQTYTRFQTLELEKEFHFNHYLTRRRRIEIAHAXCLTERQIKIWFQNRRMKLKKEL	Abdominal-A (Fragment) (AbdA)
DH_11384	Daphnia pulex	RRRGRQTYTRYQTLELEKEFHFNHYLTRRRRIEIAHALCLTERQIKIWFQNRRMKLKKEL	YAS_Hox_Abd-A|Dappu1
DH_4479	Acanthokara kaputensis	RRRGRQTYTRYQTLELEKEFHFNHYLTRRRRIEIAHVLCLTERQIKIWFQNRRMKLKKEL	Abdominal-A (Fragment) (abd-A)
DH_11432	Daphnia pulex	RRRGRQTYTRYQTLELEKEFHFNHYLTRRRRIEIAHALCLTERQIKIWFQNRRMKLKKEL	SNAP_00004289|Dappu1
DH_11401	Daphnia pulex	RRRGRQTYTRYQTLELEKEFHFNHYLTRRRRIEIAHALCLTERQIKIWFQNRRMKLKKEL	gw1.7.452.1|Dappu1
DH_3	Artemia sanfranciscana	RRRGRQTYTRYQTLELEKEFHFNHYLTRRRRIEIAHALCLTERQIKIWFQNRRMKLKKEL	Homeobox protein abdominal-A homolog (Fragment) (ABDA)
DH_5825	Artemia parthenogenetica	RRRGRQTYTRYQTLELEKEFHFNHYLTRRRRIEIAHALCLTERQIKIWFQNRRMKLKKEL	Abdominal-A (Fragment) (abdA)
DH_5826	Artemia urmiana	RRRGRQTYTRYQTLELEKEFHFNHYLTRRRRIEIAHALCLTERQIKIWFQNRRMKLKKEL	Abdominal-A (Fragment) (abdA)
DH_5827	Artemia sinica	RRRGRQTYTRYQTLELEKEFHFNHYLTRRRRIEIAHALCLTERQIKIWFQNRRMKLKKEL	Abdominal-A (Fragment) (abdA)
DH_5820	Artemia parthenogenetica	RRRGRQTYTRYQTLELEKEFHFNHYLTRRRRIEIAHALCLTERQIKIWFQNRRMKLKKEL	Abdominal-A (Fragment) (abdA)
DH_5821	Artemia parthenogenetica	RRRGRQTYTRYQTLELEKEFHFNHYLTRRRRIEIAHALCLTERQIKIWFQNRRMKLKKEL	Abdominal-A (Fragment) (abdA)
DH_5822	Artemia parthenogenetica	RRRGRQTYTRYQTLELEKEFHFNHYLTRRRRIEIAHALCLTERQIKIWFQNRRMKLKKEL	Abdominal-A (Fragment) (abdA)
DH_5833	Artemia sanfranciscana	RRRGRQTYTRYQTLELEKEFHFNHYLTRRRRIEIAHALCLSERQIKIWFQNRRMKLKKGL	Abdominal-A (Fragment) (abdA)
DH_5835	Artemia persimilis	RRRGRQTSTRYQTLQLEKEFHFNHSLTRRRRIEIAHALCLSERQIKIWFQNRRMKLKKEL	Abdominal-A (Fragment) (abdA)
DH_5829	Artemia sinica	RRRGRETYTRYQTLELEKEFDFNHYLTRRRRIEIAHALCLTERQIKIWFQNRRMKLKKEL	Abdominal-A (Fragment) (abdA)
DH_5830	Artemia sinica	RRRGRETYTRYQTLELEKEFDFNHYLTRRRRIEIAHALCLTERQIKIWFQNRRMKLKKEL	Abdominal-A (Fragment) (abdA)
DH_5828	Artemia sinica	RRRGRQTYTRYQTLELEKEFDFNHYLTRRRRIEIAHALCLTERQIKIWFQNRRMKLKKEL	Abdominal-A (Fragment) (abdA)
DH_419	Homo sapiens	GRRGRQTYTRYQTLELEKEFHFNRYLTRRRRIEIANALCLTERQIKIWFQNRRMKWKKEN	Homeobox protein Hox-A6 (HOXA6)
DH_1693	Homo sapiens	GRRGRQTYTRYQTLELEKEFHFNRYLTRRRRIEIANALCLTERQIKIWFQNRRMKWKKEN	Homeo box A6 (Homeobox A6, isoform CRA_b) (HOXA6)
DH_2752	Callicebus moloch	GRRGRQTYTRYQTLELEKEFHFNRYLTRRRRIEIANALCLTERQIKIWFQNRRMKWKKEN	Homeobox A6 (Predicted) (HOXA6)
DH_2810	Rhinolophus ferrumequinum	GRRGRQTYTRYQTLELEKEFHFNRYLTRRRRIEIANALCLTERQIKIWFQNRRMKWKKEN	Homeobox A6 (Predicted) (HOXA6)
DH_420	Mus musculus	GRRGRQTYTRYQTLELEKEFHFNRYLTRRRRIEIANALCLTERQIKIWFQNRRMKWKKEN	Homeobox protein Hox-A6 (Hoxa6)
DH_5031	Mus musculus	GRRGRQTYTRYQTLELEKEFHFNRYLTRRRRIEIANALCLTERQIKIWFQNRRMKWKKEN	Homeo box A6 (Hoxa6)
DH_417	Gallus gallus	GRRGRQTYTRYQTLELEKEFHFNRYLTRRRRIEIANALCLTERQIKIWFQNRRMKWKKEN	Homeobox protein Hox-A6 (HOXA6)
DH_8026	Latimeria menadoensis	GRRGRQTYTRYQTLELEKEFHFNRYLTRRRRIEIANALCLTGRQIGIWFQNRRMKWKKES	HoxA6 (Fragment)
DH_418	Heterodontus francisci	GRRGRQTYTRFQTLELEKEFHFNRYLTRRRRIEIANALCLTERQIKIWFQNRRMKWKKEN	Homeobox protein Hox-A6 (HOXA6)
DH_9629	Priapulus caudatus	RRRGRQTYTRFQTLELEKEFHFNRYLTRRRRIEIANMLCLTERQIKIWFQNRRMKWKKEK	HB3 homeodomain protein (Fragment) (HB3)
DH_8508	Petromyzon marinus	GRKGRRSYSRHQSLELEKEFHFNRYLARRRRVEIAHSLCLSERQVKIWFQNRRMKWKKER	HoxF5 homeobox (Fragment)
DH_7518	Lampetra japonica	--------------------HFNRYLTRRRRVEIAHSLCLTERQIKIWFQNRRMKWKKEN	LjHox7m Homeobox (Fragment) (LjHox7m)
DH_6344	Tubifex tubifex	--------------------HFNRYLTRRRRIEIAHTLCLSERQIKIWFQNRRMKWKKEN	Homeodomain (Fragment) (Ttu-tox14)
DH_8505	Petromyzon marinus	GKRGRQTYSRYQTLELEKEFHFNRYLTRRRRVEIAHSLCLTERQIKIWFQNRRMKWKKEN	HoxK6 homeobox (Fragment)
DH_8504	Petromyzon marinus	SRRARTAYSRYQTLELEKEFHFNRYLTRRRRVEIAHSLCLTERQIKIWFQNRRMKWKKET	HoxL5/6 homeobox (Fragment)
DH_6353	Tubifex tubifex	--------------------HFNRYLTRRRRIEIAHALALTERQIKIWFQNRRMKWKKEN	Homeodomain (Fragment) (Ttu-tox5)
DH_8649	Lithobius atkinsoni	--------------------HFNRYLTRRRRIEIAHALGLTERQIKIWFQNRRMKAKKEN	Fushi tarazu (Fragment) (ftz)
DH_8653	Lithobius atkinsoni	--------------------HFNRYLTRRRRIEIAHALCLTERQIKIWFQNRRMKWKKEN	Antennapedia (Fragment) (Antp)
DH_5037	Glomeris marginata	--------------------HFNRYLTRRRRIEIAHALCLTERQIKIWFQNRRMKWKKEN	Antennapedia (Fragment) (antp)
DH_1402	Pan paniscus	----------------EKEFHYNRYLTRRRRIEIAHALCLTERQIKIWFXXRRMKWKKEN	HOXB7 (Fragment) (HOXB7)
DH_1602	Pan troglodytes	--------------------HYNRYLTRRRRIEIAHALCLTERQIKIWFXXRRMKWKKEN	HOXB7 (Fragment) (HOXB7)
DH_5413	Strigamia maritima	--------------ELEKEFHFNRYLTRRRRIEIAHALCLTERQIKIWFQNRRMKWKKEN	Antennapedia (Fragment) (antP)
DH_6845	Oreochromis niloticus	------------TLELEKEFHFNRYLTRRRRIETAHALCLTERQIKIWFQNRRMKWKKEN	Hox protein (Fragment)
DH_8358	Ciona intestinalis	RRRGRQTYSRHQTLELEKEFHYNRYLTRRRRIEVAHTKCLTERQIKIWFQNRRMKWKKEN	Putative homeobox protein Hox6/7 (Fragment) (hox6/7)
DH_9283	Milnesium tardigradum	SKRTRQTYTRYQTLELEKEFLYNRYLTRRRRIEIASSLGLSERQIKINENTRIESFUNDN	Fushi tarazu (Fragment) (ftz)
DH_6781	Oncorhynchus mykiss	RRRGRQIYSRYQTLELEKEFHFNRYLTRRRRI----------------------------	HoxC6bii (Fragment) (Hox)
DH_6809	Trionyx sinensis	RRRGRQIYSRYQTLELEKEFHFNRYLTRRRRIEIANALCLTERQIKIWFQ----------	Hoxc-6 (Fragment) (Hoxc-6)
DH_6814	Gallus gallus	RRRGRQIYSRYQTLELEKEFHFNRYLTRRRRIEIANALCLTERQIKIWFQN---------	Hoxc-6 (Fragment) (Hoxc-6)
DH_1548	Megalobrama amblycephala	RRRGRQIYSRYQTLELEKEFHFNRYLTRRRRIEIANALCLTERQIKIWFQNRRMKSMKDT	Homeodomain protein (Fragment) (HoxC6a)
DH_5713	Bos taurus	RSRGRQIYSRYQTLELEKEFHFNRYLTRRRRIEIANALCLTERQIKIWFQNR---------	Antennapedia-type homeotic factor (Fragment)
DH_562	Danio rerio	RRRGRQIYSRYQTLELEKEFHFNRYLTRRRRIEIANALCLTERQIKIWFQNRRMKWKKET	Homeobox protein Hox-C6a (hoxc6a)
DH_1462	Danio rerio	RRRGRQIYSRYQTLELEKEFHFNRYLTRRRRIEIANALCLTERQIKIWFQNRRMKWKKET	Homeo box C6a (hoxc6a)
DH_4303	Danio rerio	RRRGRQIYSRYQTLELEKEFHFNRYLTRRRRIEIANALCLTERQIKIWFQNRRMKWKKET	Putative uncharacterized protein
DH_9386	Oryzias latipes	--------------------HFNRYLTRRRRIEIANALCLTERQIKIWFQNRRMKWKKES	HOX6A (Fragment) (hox6a)
DH_9631	Priapulus caudatus	-------------------FHFNRYLTRRRRIEIANVLRLTERQIKIWFQNRRMKWKKEH	HB1 homeodomain protein (Fragment) (HB1)
DH_8050	Rattus sp	------------------EFHFNRYLTRRRRIEIANALCLTERQIKIWFQNRRMKWKKEN	Hox-A|Hox-1 (Fragment) (Hox-A|Hox-1)
DH_2142	Haplochromis burtoni	RRRGRQIYSRYQTLELEKEFHFNRYLTRRRRIEIANALCLTERQIKIWFQNRRMKWKKES	Hoxc6a (hoxc6a)
DH_6159	Oryzias latipes	RRRGRQIYSRYQTLELEKEFHFNRYLTRRRRIEIANALCLTERQIKIWFQNRRMKWKKES	HoxC6a (hoxC6a)
DH_559	Notophthalmus viridescens	RRRGRQIYSRYQTLELEKEFHFNRYLTRRRRIEIANASCLTERQIKIWFQNRRMKWKKES	Homeobox protein Hox-C6 (HOXC6)
DH_563	Fugu rubripes	RRRGRQIYSRYQTLELEKEFHFNRYLTRRRRIEIANALCLTERQIKIWFQNRRMKWKKES	Homeobox protein Hox-C6a (hoxc6a)

DH_11810	Gasterosteus aculeatus	RRRGRQIYSRYQTLELEKEFHFNRYLTRRRRIEIANALCLTERQIKIWFQNRRMKWKKES	HoxC6 (Fragment). [Source:UniProtKB/TrEMBL;Acc:Q4VQD3]
DH_11778	Gasterosteus aculeatus	RRRGRQIYSRYQTLELEKEFHFNRYLTRRRRIEIANALCLTERQIKIWFQNRRMKWKKES	HoxC6 (Fragment). [Source:UniProtKB/TrEMBL;Acc:Q4VQD3]
DH_6669	Gasterosteus aculeatus	RRRGRQIYSRYQTLELEKEFHFNRYLTRRRRIEIANALCLTERQIKIWFQNRRMKWKKES	HoxC6 (Fragment)
DH_6711	Oreochromis niloticus	RRRGRQIYSRYQTLELEKEFHFNRYLTRRRRIEIANALCLTERQIKIWFQNRRMKWKKES	Hox protein (Fragment)
DH_557	Homo sapiens	RRRGRQIYSRYQTLELEKEFHFNRYLTRRRRIEIANALCLTERQIKIWFQNRRMKWKKES	Homeobox protein Hox-C6 (HOXC6)
DH_558	Mus musculus	RRRGRQIYSRYQTLELEKEFHFNRYLTRRRRIEIANALCLTERQIKIWFQNRRMKWKKES	Homeobox protein Hox-C6 (Hoxc6)
DH_2863	Mus musculus	RRRGRQIYSRYQTLELEKEFHFNRYLTRRRRIEIANALCLTERQIKIWFQNRRMKWKKES	Homeo box C6 (Hoxc6)
DH_7483	Homo sapiens	RRRGRQIYSRYQTLELEKEFHFNRYLTRRRRIEIANALCLTERQIKIWFQNRRMKWKKES	HOXC6 protein (HOXC6)
DH_2840	Homo sapiens	RRRGRQIYSRYQTLELEKEFHFNRYLTRRRRIEIANALCLTERQIKIWFQNRRMKWKKES	cDNA, FLJ95712, Homo sapiens homeo box C6 (HOXC6), transcript variant 2, mRNA (Homeobox C6, isoform CRA_
DH_6748	Mus musculus	RRRGRQIYSRYQTLELEKEFHFNRYLTRRRRIEIANALCLTERQIKIWFQNRRMKWKKES	Putative uncharacterized protein (Hoxc6)
DH_560	Ovis aries	RRRGRQIYSRYQTLELEKEFHFNRYLTRRRRIEIANALCLTERQIKIWFQNRRMKWKKES	Homeobox protein Hox-C6 (HOXC6)
DH_561	Xenopus laevis	RRRGRQIYSRYQTLELEKEFHFNRYLTRRRRIEIANALCLTERQIKIWFQNRRMKWKKES	Homeobox protein Hox-C6 (hoxc6)
DH_6693	Xenopus laevis	RRRGRQIYSRYQTLELEKEFHFNRYLTRRRRIEIANALCLTERQIKIWFQNRRMKWKKES	Putative uncharacterized protein
DH_3258	Salmo salar	RRRGRQIYSRYQTLELEKEFHFNRYLTRRRRIEIANALCLTERQIKIWFQNRRMKWKKES	Homeobox protein HoxC6ba (HoxC6ba)
DH_3239	Salmo salar	RRRGRQIYSRYQTLELEKEFHFNRYLTRRRRIEIANALCLTERQIKIWFQNRRMKWKKES	Homeobox protein HoxC6aa (HoxC6aa)
DH_3248	Salmo salar	RRRGRQIYSRYQTLELEKEFHFNRYLTRRRRIEIANALCLTERQIKIWFQNRRMKWKKES	Homeobox protein HoxC6ab (HoxC6ab)
DH_9338	Gallus gallus	RRRGRQIYSRYQTLELEKEFHFNRYLTRRRRIEIANALCLTERQIKIWFQNRRMKWKKES	HOXC-6 protein (Fragment) (HOXC-6)
DH_564	Danio rerio	RRRGRQIYSRYQTLELEKEFHYNRYLTRRRRIEIANTLCLSERQIKIWFQNRRMKWKKES	Homeobox protein Hox-C6b (hoxc6b)
DH_2922	Danio rerio	RRRGRQIYSRYQTLELEKEFHYNRYLTRRRRIEIANTLCLSERQIKIWFQNRRMKWKKES	Hoxc6b protein (hoxc6b)
DH_1549	Megalobrama amblycephala	RRRGRQTYSRYQTLELEKEFHYNRYLTRRRRIEIANALCLSERQIKIWFQNRRMKWKKES	Homeodomain protein (Fragment) (HoxC6b)
DH_6782	Oncorhynchus mykiss	RRRGRQIYSRYQTLELEKEFHFNRYLTRRRRIEIANALCLSERQIKIWFQNRRMK-----	HoxC6bi (Fragment) (Hox)
DH_3265	Salmo salar	RRRGRQIYSRYQTLELEKEFHFNRYLTRRRRIEIANALCLSERQIKIWFQNRRMKWKKES	Homeobox protein HoxC6bb (HoxC6bb)
DH_7974	Diplosoma listerianum	RRRSRQTYSRYQTLELEKEFHYNRYLTRRRRIEIANALCLSERQIKIWFQNRRMKWKREN	HOX6/7 (Fragment) (Hox6/7)
DH_506	Danio rerio	GRRGRQTYTRFQTLELEKEFHFNRYLTRRRRIEISHALCLTERQIKIWFQNRRMKWKKEN	Homeobox protein Hox-B6b (hoxb6b)
DH_9385	Oryzias latipes	--------------------HFNRYLTRRRRIEISHALCLTERQIKIWFQNRRMKWKKEN	HOXB6B (Fragment) (hoxb6b)
DH_2915	Danio rerio	GRRGRQTYTRFQTLELEKEFHFNRYLTRRRRIEISHALCLTERQIKIWFQNRRMKWKKEN	Homeo box B6b (hoxb6b)
DH_3325	Salmo salar	GRRGRQTYTRYQTLELEKEFHFNRYLTRRRRIEISHALCLTERQIKTWFQNRRMKWKKEN	Homeobox protein HoxB6ba (HoxB6ba)
DH_507	Fugu rubripes	GRRGRQTYTRYQTLELEKEFHFNRYLTRRRRIEISHALCLTERQIKIWFQNRRMKWKKEN	Homeobox protein Hox-B6b (hoxb6b)
DH_7714	Sphoeroides nephelus	GRRGRQTYTRYQTLELEKEFHFNRYLTRRRRIEISHALCLTERQIKIWFQNRRMKWKKEN	HoxB6b (Hoxb6b)
DH_2157	Haplochromis burtoni	GRRGRQTYTRYQTLELEKEFHFNRYLTRRRRIEISHALCLTERQIKIWFQNRRMKWKKEN	Hoxb6b (hoxb6b)
DH_6163	Oryzias latipes	GRRGRQTYTRYQTLELEKEFHFNRYLTRRRRIEISHALCLTERQIKIWFQNRRMKWKKEN	HoxB6b (hoxB6b)
DH_6714	Oreochromis niloticus	GRRGRQTYTRYQTLELEKEFHFNRYLTRRRRIEISHALCLTERQIKIWFQNRRMKWKKEN	Hox protein (Fragment)
DH_3352	Salmo salar	GRRGRQTYTRYQTLELEKEFHFNRYLTRRRRIEISHALCLTERQIKIWFQNRRMKWKKEN	Homeobox protein HoxB6ba (HoxB6ba)
DH_3355	Salmo salar	GRRGRQTYTRYQTLELEKEFHFNRYLTRRRRIEISHALCLTERQIKIWFQNRRMKWKKEN	Homeobox protein HoxB6bb (HoxB6bb)
DH_8014	Latimeria menadoensis	GRRGRQTYTRYQTLELEKEFHFNRYVTRRRRIEIAHALCLTERQIKIWFQNRRMKWKKEN	HoxB6 (Fragment)
DH_505	Fugu rubripes	GRRGRQTYTRYQTLELEKEFHFNRYLTRRRRIEIAHALCLTERQIKIWFQNRRMKWKKEN	Homeobox protein Hox-B6a (hoxb6a)
DH_2129	Haplochromis burtoni	GRRGRQTYTRYQTLELEKEFHFNRYLTRRRRIEIAHALCLTERQIKIWFQNRRMKWKKEN	Hoxb6a (hoxb6a)
DH_11809	Gasterosteus aculeatus	GRRGRQTYTRYQTLELEKEFHFNRYLTRRRRIEIAHALCLTERQIKIWFQNRRMKWKKEN	groupXI
DH_6510	Tetraodon nigroviridis	GRRGRQTYTRYQTLELEKEFHFNRYLTRRRRIEIAHALCLTERQIKIWFQNRRMKWKKEN	Chromosome undetermined SCAF14653, whole genome shotgun sequence (GSTENG00020456001)
DH_6170	Oryzias latipes	GRRGRQTYTRYQTLELEKEFHFNRYLTRRRRIEIAHALCLTERQIKIWFQNRRMKWKKEN	HoxB6a (hoxB6a)
DH_504	Danio rerio	GRRGRQTYTRYQTLELEKEFHFNRYLTRRRRIEIAHALCLTERQIKIWFQNRRMKWKKEN	Homeobox protein Hox-B6a (hoxb6a)
DH_2913	Danio rerio	GRRGRQTYTRYQTLELEKEFHFNRYLTRRRRIEIAHALCLTERQIKIWFQNRRMKWKKEN	Homeo box B6a (hoxb6a)
DH_3309	Salmo salar	GRRGRQTYTRYQTLELEKEFHFNRYLTRRRRIEIAHALCLTERQIKIWFQNRRMKWKKEN	Homeobox protein HoxB6aa (HoxB6aa)
DH_3347	Salmo salar	GRRGRQTYTRYQTLELEKEFHFNRYLTRRRRIEIAHALCLTERQIKIWFQNRRMKWKKEN	Homeobox protein HoxB6aa (HoxB6aa)
DH_3319	Salmo salar	GRRGRQTYTRYQTLELEKEFHFNRYLTRRRRIEIAHALCLTERQIKIWFQNRRMKWKKEN	Homeobox protein HoxB6ab (Homeobox protien HoxB6ab) (HoxB6ab)
DH_8066	Zaprionus vittiger	------------------EFHFNRYLTRRRRIEIAHALCLTERQIKIWFQNRRM------	Antp protein (Scr protein) (Fragment) (Antp)
DH_8067	Scaptodrosophila pattersoni	------------------EFHFNRYLTRRRRIEIAHALCLTERQIKIWFQNRRM------	Antp protein (Scr protein) (Fragment) (Antp)
DH_8068	Hirtodrosophila pictiventris	------------------EFHFNRYLTRRRRIEIAHALCLTERQIKIWFQNRRM------	Antp protein (Scr protein) (Fragment) (Antp)
DH_8069	Drosophila heteroneura	------------------EFHFNRYLTRRRRIEIAHALCLTERQIKIWFQNRRM------	Antp protein (Scr protein) (Fragment) (Antp)
DH_8070	Drosophila adiastola	------------------EFHFNRYLTRRRRIEIAHALCLTERQIKIWFQNRRM------	Ftz protein (Scr protein) (Antp protein) (Fragment) (Scr)
DH_8071	Chymomyza amoena	------------------EFHFNRYLTRRRRIEIAHALCLTERQIKIWFQNRRM------	Antp protein (Scr protein) (Fragment) (Antp)
DH_8177	Mesocestoides vogae	--------------ELEKEFHFNRYLTRRRRIEIAHALCLTERQIKIWFQNRR-------	Homeodomain-containing protein Hox5 (Fragment)
DH_501	Homo sapiens	GRRGRQTYTRYQTLELEKEFHYNRYLTRRRRIEIAHALCLTERQIKIWFQNRRMKWKKES	Homeobox protein Hox-B6 (HOXB6)
DH_2212	Homo sapiens	GRRGRQTYTRYQTLELEKEFHYNRYLTRRRRIEIAHALCLTERQIKIWFQNRRMKWKKES	cDNA FLJ77171, highly similar to Homo sapiens homeo box B6 (HOXB6), transcript variant 2, mRNA (HCG20006
DH_502	Mus musculus	GRRGRQTYTRYQTLELEKEFHYNRYLTRRRRIEIAHALCLTERQIKIWFQNRRMKWKKES	Homeobox protein Hox-B6 (Hoxb6)
DH_1421	Mus musculus	GRRGRQTYTRYQTLELEKEFHYNRYLTRRRRIEIAHALCLTERQIKIWFQNRRMKWKKES	Homeo box B6 (Hoxb6)
DH_5229	Homo sapiens	-------------SELEKEFHYNRYLTRRRRIEIAHALCLTERQRKIWFQNP--------	HOX B6 protein (Fragment) (HOX B6)
DH_6527	Tetraodon nigroviridis	GRRGRQTYTRYQTLELEKEFHFNRYLTRRRRIEISHALCLTERQIKIWFQNRRMKLGVYG	Chromosome 2 SCAF14604, whole genome shotgun sequence (GSTENG00019143001)
DH_9263	Pachymerium ferrugineum	--------TRYQTLELEKEFHFNRYLTRRRRIEIAHALCLTERQIKIW------------	Putative Antennapedia (Fragment) (antp)
DH_9434	Acanthokara kaputensis	----------YQTLELEKEFHFNRYLTRRRRIEIAHALCLTERQIK-------------	Antennapedia (Fragment) (Antp)
DH_4764	Xenopus laevis	GRRGRQTYTRYQTLELEKEFHFNRYLTRRRRIEIAHSLCLTERQIKIWFQNRRMKWKKES	Hoxb6 protein (hoxb6)
DH_500	Gallus gallus	ARRGRQTYTRYQTLELEKEFHFNRYLTRRRRIEIAHSLCLTERQIKIWFQNRRMKWKKEN	Homeobox protein Hox-B6 (Fragment) (HOXB6)
DH_4477	Ethmostigmus rubripes	------SYTRYQTLELEKEFHFNRYLTRRRRIEIAHSLCLSERQIKIWFQNRRMKWKKEH	Sex combs reduced (Fragment) (Scr)
DH_5039	Glomeris marginata	------SYTRYQTLELEKEFHFNRYLTRRRRIEIAHSLCLTERQIKIWFQNRRMKWKKEH	Sex combs reduced (Fragment) (scr)
DH_9261	Pachymerium ferrugineum	--------TRYQTLELEKEFHFNRYLTRRRRIEIAHALCLSERQIKIW------------	Putative Sex combs reduced (Fragment) (scr)
DH_8084	Spadella cephaloptera	-------------LELEKEFHFNRYLTRRRRIEIAHALCLTERQIKI-------------	Hox scmedian 1 (Fragment)
DH_8083	Spadella cephaloptera	-------------LELEKEFHFNRYLTRRRRIEMAHALCLTERQIKI-------------	Hox scmedian 3 (Fragment)

Save alignment [fasta format] – Save complete sequences [fasta format]

PG7

ID	organism	10 20 30 40 50 60	description
DH_12318	Helobdella robusta	QKRTRQTYTRYQTLELEKEFYSNRYLTRRRRIEIAHSLALSERQIKIWFQNRRMKWKKEN	estExt_Genewise1Plus.C_900048|Helro1
DH_4406	Helobdella sp. MS-2000	QKRTRQTYTRYQTLELEKEFYSNRYLTRRRRIEIAHSLALSERQIKIWFQNRRMKWKKEN	LOX5 (Fragment) (Lox5)
DH_8631	Euprymna scolopes	QKRTRQTYTRFQTLELEKEFHFNRYLTRRRRIEIAHSLGLSERQIKIWFQNRRMKWKKEN	Lox5 homeodomain protein (Fragment) (Lox5)
DH_7794	Ctenodrilus serratus	--------------------HYNRYLTRRRRIEIAHALGLSERQIKIWFQNRRMKWKKEN	CTs-Lox5 protein (Fragment) (CTs-Lox5)
DH_5700	Ctenodrilus serratus	--------------------HYNRYLTRRRRIEIAHALGLSERQIKIWFQNRRMKWKKEN	Lox5 ortholog homeobox (Fragment) (CTs-Lox5)
DH_413	Salmo salar	GRGRGQTYTRYQTLELEKEFHFNRYLTRRRRIEMAHALCLSERQIKIWFQNRRMKWKKDN	Homeobox protein Hox-A5 (Fragment) (hoxa5)
DH_5805	Holopneustes purpurescens	-----------QTLELEKEFHFNRYLTRRRRIEIAHALGLTERQIKIWFQNRRMKWKKEH	Homeoprotein (Fragment) (Hox5)
DH_8079	Spadella cephaloptera	--------------------HFNRYLTRRRRIEIAHALGLAERQIKIWFQNRRMKWKKEH	Hox median 4 protein (Fragment)
DH_1263	Saccoglossus kowalevskii	QRRGRQTYTRYQTLELEKEFHFNRYLTRRRRIEIAHTLGLTERQIKIWFQNRRMKWKKEQ	Hox 6
DH_7669	Ptychodera flava	QRRGRQTYTRYQTLELEKEFHFNRYLTRRRRIEIAHALGLTERQIKIWFQNRRMKWKKEQ	Transcription factor Hox6
DH_9138	Discocelis tigrina	RRRGRQTYSRNQTLELEKEFQYSRYLTRRRRIEIAHMLQLTERQIKIWFQNRRMKQKKDK	Ubd-A protein (Fragment) (hoxF)
DH_6348	Tubifex tubifex	--------------------HYNRYLTRRRRIEVAHQLSLTERQIKIWFQNRRMKWKKDY	Homeodomain (Fragment) (Ttu-tox10)
DH_7718	Sphoeroides nephelus	RQRGRQSYTRQQSLELEKEFHSGRYLTRRRRGEIAHALRLTERQVKIWFQNRRMKWKKEK	HoxB7a (Fragment)
DH_6719	Oreochromis niloticus	RRRGRQTYTRHQSLELEKEFHFNRYLTRKRQIEVAHALGLTERQIKIWFQNRRMKWKKEN	Hox protein (Fragment)
DH_513	Danio rerio	RKRGRQTYSRYQTLELEKEFHFNRYLSRRRRIEIAHALCLTERQIKIWFQNRRMKWKKEN	Homeobox protein Hox-B7a (hoxb7a)
DH_2926	Danio rerio	RKRGRQTYSRYQTLELEKEFHFNRYLSRRRRIEIAHALCLTERQIKIWFQNRRMKWKKEN	Homeo box B7a (hoxb7a)
DH_1541	Megalobrama amblycephala	RKRGRQTYSRYQTLELEIEFHFNRYLSRRRRIEIAHALCLTERQIKIWFQNRRMKW----	Homeodomain protein (Fragment) (HoxB7a)
DH_425	Morone saxatilis	RKRGRQTYSRYQTLELEKEFHFNRYLTRRRRIEIAHALCLSERQIKIWFQNRRMKWKKDH	Homeobox protein Hox-A7 (hoxa7)
DH_2151	Haplochromis burtoni	RKRGRQTYSRHQTLELEKEFHFNRYLTRRRRIEIAHALCLSERQIKIWFQNRRMKWKKDH	Hoxa7a (hoxa7a)
DH_11790	Gasterosteus aculeatus	RKRGRQTYSRYQTLELEKEFHFNRYLTRRRRIEIAHALCLTERQIKIWFQNRRMKWKKDQ	groupX
DH_9492	Lingula unguis	RKRGRHTYSRHQTLELEKEFHFNRYLTRRRRIEIAHALCLTERQIKIWFQNRRMKWKKEN	Antennapedia-like homeodomain protein (Fragment)
DH_11379	Daphnia pulex	RKRGRQTYTRFQTLELEKEFHFNRYLTRRRRIEIAHALCLTERQIKIWFQNRRMKWKKEN	fgenesh1_pm.C_scaffold_7000012|Dappu1
DH_11381	Daphnia pulex	RKRGRQTYTRFQTLELEKEFHFNRYLTRRRRIEIAHALCLTERQIKIWFQNRRMKWKKEN	YAS_fgenesh1_pm.C_scaffold_7000012|Dappu1
DH_8940	Daphnia magna	RKRGRQTYTRFQTLELEKEFHFNRYLTRRRRIEIAHALCLTERQIKIWFQNRRMKWKKEN	Homeotic protein (Antennapedia) (Antp)
DH_11400	Daphnia pulex	RKRGRQTYTRFQTLELEKEFHFNRYLTRRRRIEIAHALCLTERQIKIWFQNRRMKWKKEN	e_gw1.7.449.1|Dappu1
DH_5208	Moina macrocopa	RKRGRQTYTRFQTLELEKEFHFNRYLTRRRRIEIAHALCLTERQIKIWFQNRRMKWKKEN	Antennapedia (Antp)
DH_5207	Artemia sanfranciscana	RKRGRQTYTRFQTLELEKEFHFNRYLTRRRRIEIAHALCLTERQIKIWFQNRRMKWKKEN	Antennapedia (Antp)
DH_8643	Artemia sanfranciscana	RKRGRQTYTRFQTLELEKEFHFNRYLTRRRRIEIAHALCLTERQIKIWFQNRRMKWKKEN	Antennapedia (Antp)
DH_4757	Artemia sanfranciscana	RKRGRQTYTRFQTLELEKEFHFNRYLTRRRRIEIAHALCLTERQIKIWFQNRRMKWKKEN	Antp protein (Fragment) (Antp)
DH_8167	Calanus helgolandicus	RKRGRQTYTRYQTLELEKEFHFNRYLTRRRRIEIAHALCLTERQIKIWFQNRRMKWKKEN	Putative antennapedia protein 1 (Fragment) (antp1)
DH_4593	Cupiennius salei	RKRGRQTYTRYQTLELEKEFHFNRYLTRRRRIEIAHALCLTERQIKIWFQNRRMKWKKEN	Homeobox protein (Fragment) (antennapedia)
DH_11990	Capitella sp. I Grassle and Grassle, 1976	RKRGRQTYTRYQTLELEKEFHFNRYLTRRRRIEIAHALCLTERQIKIWFQNRRMKWKKEN	e_gw1.70.47.1|Capca1
DH_8630	Euprymna scolopes	RKRGRQTYTRYQTLELEKEFHFNRYLTRRRRIEIAHALCLTERQIKIWFQNRRMKWKKEN	Antennapedia homeodomain protein (Fragment) (Antp)
DH_29	Drosophila melanogaster	RKRGRQTYTRYQTLELEKEFHFNRYLTRRRRIEIAHALCLTERQIKIWFQNRRMKWKKEN	Homeotic protein antennapedia (Antp)
DH_1770	Drosophila melanogaster	RKRGRQTYTRYQTLELEKEFHFNRYLTRRRRIEIAHALCLTERQIKIWFQNRRMKWKKEN	CG1028-PJ, isoform J (CG1028-PL, isoform L) (CG1028-PM, isoform M) (Antp)
DH_3577	Drosophila sechellia	RKRGRQTYTRYQTLELEKEFHFNRYLTRRRRIEIAHALCLTERQIKIWFQNRRMKWKKEN	GM10522 (GM10522)
DH_4235	Drosophila simulans	RKRGRQTYTRYQTLELEKEFHFNRYLTRRRRIEIAHALCLTERQIKIWFQNRRMKWKKEN	GD19515 (GD19515)
DH_7812	Drosophila melanogaster	RKRGRQTYTRYQTLELEKEFHFNRYLTRRRRIEIAHALCLTERQIKIWFQNRRMKWKKEN	CG1028-PD, isoform D (CG1028-PN, isoform N) (Antp)
DH_4141	Drosophila yakuba	RKRGRQTYTRYQTLELEKEFHFNRYLTRRRRIEIAHALCLTERQIKIWFQNRRMKWKKEN	GE25826 (GE25826)
DH_7814	Drosophila melanogaster	RKRGRQTYTRYQTLELEKEFHFNRYLTRRRRIEIAHALCLTERQIKIWFQNRRMKWKKEN	CG1028-PG, isoform G (CG1028-PK, isoform K) (Antp)
DH_3174	Drosophila erecta	RKRGRQTYTRYQTLELEKEFHFNRYLTRRRRIEIAHALCLTERQIKIWFQNRRMKWKKEN	Antp (Dere\Antp)
DH_7813	Drosophila melanogaster	RKRGRQTYTRYQTLELEKEFHFNRYLTRRRRIEIAHALCLTERQIKIWFQNRRMKWKKEN	CG1028-PE, isoform E (CG1028-PF, isoform F) (Antp)
DH_3458	Drosophila persimilis	RKRGRQTYTRYQTLELEKEFHFNRYLTRRRRIEIAHALCLTERQIKIWFQNRRMKWKKEN	GL22036 (GL22036)
DH_4329	Drosophila pseudoobscura pseudoobscura	RKRGRQTYTRYQTLELEKEFHFNRYLTRRRRIEIAHALCLTERQIKIWFQNRRMKWKKEN	Antp (Dpse\Antp)
DH_30	Drosophila subobscura	RKRGRQTYTRYQTLELEKEFHFNRYLTRRRRIEIAHALCLTERQIKIWFQNRRMKWKKEN	Homeotic protein antennapedia (Antp)
DH_2997	Drosophila ananassae	RKRGRQTYTRYQTLELEKEFHFNRYLTRRRRIEIAHALCLTERQIKIWFQNRRMKWKKEN	GF17789 (GF17789)
DH_3897	Drosophila virilis	RKRGRQTYTRYQTLELEKEFHFNRYLTRRRRIEIAHALCLTERQIKIWFQNRRMKWKKEN	Antennapedia (Dvir\Antp)
DH_7693	Drosophila virilis	RKRGRQTYTRYQTLELEKEFHFNRYLTRRRRIEIAHALCLTERQIKIWFQNRRMKWKKEN	Antennapedia (Antp)
DH_3678	Drosophila grimshawi	RKRGRQTYTRYQTLELEKEFHFNRYLTRRRRIEIAHALCLTERQIKIWFQNRRMKWKKEN	GH19475 (GH19475)
DH_4018	Drosophila willistoni	RKRGRQTYTRYQTLELEKEFHFNRYLTRRRRIEIAHALCLTERQIKIWFQNRRMKWKKEN	GK10824 (GK10824)
DH_7800	Drosophila pseudoobscura pseudoobscura	RKRGRQTYTRYQTLELEKEFHFNRYLTRRRRIEIAHALCLTERQIKIWFQNRRMKWKKEN	Antp-PA (Fragment) (Antp)
DH_2589	Culex quinquefasciatus	RKRGRQTYTRYQTLELEKEFHFNRYLTRRRRIEIAHALCLTERQIKIWFQNRRMKWKKEN	Homeotic antennapedia protein (CpipJ_CPIJ001008)
DH_4590	Anopheles gambiae	RKRGRQTYTRYQTLELEKEFHFNRYLTRRRRIEIAHALCLTERQIKIWFQNRRMKWKKEN	Antennapedia homeotic protein (AGAP004660-PA) (Antp)

DH_5563	Drosophila virilis	RKRGRQTYTRYQTLELEKEFHFNRYLTRRRRIEIAHALCLTERQIKIWFQNRRMKWKKEN	Homeobox protein (Fragment) (Antp)
DH_2700	Culex quinquefasciatus	RKRGRQTYTRYQTLELEKEFHFNRYLTRRRRIEIAHALCLTERQIKIWFQNRRMKWKKEN	Homeotic antennapedia protein (CpipJ_CPIJ019078)
DH_7801	Drosophila erecta	RKRGRQTYTRYQTLELEKEFHFNRYLTRRRRIEIAHALCLTERQIKIWFQNRRMKWKKEN	Antp-PA (Fragment) (Antp)
DH_5300	Bombyx mori	RKRGRQTYTRYQTLELEKEFHFNRYLTRRRRIEIAHALCLTERQIKIWFQNRRMKWKKEN	Antennapedia homologue protein
DH_8924	Tribolium castaneum	RKRGRQTYTRYQTLELEKEFHFNRYLTRRRRIEIAHALCLTERQIKIWFQNRRMKWKKEN	Homeodomain transcription factor Prothoraxless (ptl)
DH_9286	Tribolium castaneum	RKRGRQTYTRYQTLELEKEFHFNRYLTRRRRIEIAHALCLTERQIKIWFQNRRMKWKKEN	Prothoraxless
DH_8992	Folsomia candida	RKRGRQTYTRYQTLELEKEFHFNRYLTRRRRIEIAHALCLTERQIKIWFQNRRMKWKKEN	Antennapedia (Fragment)
DH_3308	Salmo salar	RKRGRQTYTRYQTLELEKEFHFNRYLTRRRRIEIAHALCLTERQIKIWFQNRRMKWKKEN	Homeobox protein Hox-B7a (HoxB7aa)
DH_8983	Lithobius forficatus	RKRGRQTYTRYQTLELEKEFHFNRYLTRRRRIEIAHALCLTERQIKIWFQNRRMKWKKEN	Antennapedia 1 (Fragment) (Antp1)
DH_4474	Ethmostigmus rubripes	RKRGRQTYTRYQTLELEKEFHFNRYLTRRRRIEIAHALCLTERQIKIWFQNRRMKWKKEN	Antennapedia (Fragment) (Antp)
DH_4686	Sacculina carcini	RKRGRQTYTRYQTLELEKEFHFNRYLTRRRRIEIAHALCLTERQIKIWFQNRRMKWKKEN	Antennapedia (Antp)
DH_3318	Salmo salar	RKRGRQTYTRYQTLELEKEFHFNRYLTRRRRIEIAHALCLTERQIKIWFQNRRMKWKKEN	Homeobox protein HoxB7ab (Homeobox protien HoxB7ab) (HoxB7ab)
DH_5672	Schistocerca americana	RKRGRQTYTRYQTLELEKEFHFNRYLTRRRRIEIAHALCLTERQIKIWFQNRRMKWKKEN	Homeotic protein (Antp)
DH_9065	Porcellio scaber	RKRGRQTYTRYQTLELEKEFHFNRYLTRRRRIEIAHALCLTERQIKIWFQNRRMKWKKEN	Antp (Fragment)
DH_2472	Nymphon gracile	RKRGRQTYTRYQTLELEKEFHFNRYLTRRRRIEIAHALCLTERQIKIWFQNRRMKWKKEN	Homeobox protein antennapedia (Fragment) (Ant)
DH_2471	Endeis spinosa	RKRGRQTYTRYQTLELEKEFHFNRYLTRRRRIEIAHALCLTERQIKIWFQNRRMKWKKEN	Homeobox protein antennapedia (Fragment) (Ant)
DH_5272	Aedes aegypti	RKRGRQTYTRYQTLELEKEFHFNRYLTRRRRIEIAHALCLTERQIKIWFQNRRMKWKKEN	Homeotic antennapedia protein, putative (AAEL009947)
DH_3745	Drosophila mojavensis	RKRGRQTYTRYQTLELEKEFHFNRYLTRRRRIEIAHALCLTERQIKIWFQNRRMKWKKEN	GI24713 (GI24713)
DH_9196	Apis mellifera	RKRGRQTYTRYQTLELEKEFHYNRYLTRRRRIEIAHALCLTERQIKIWFQNRRMKWKKEN	Antennapedia protein (antennapedia)
DH_509	Gorilla gorilla gorilla	RKRGRQTYTRYQTLELEKEFHYNRYLTRRRRIEIAHALCLTERQIKIWFQNRRMKWKKEN	Homeobox protein Hox-B7 (HOXB7)
DH_508	Bos taurus	RKRGRQTYTRYQTLELEKEFHYNRYLTRRRRIEIAHALCLTERQIKIWFQNRRMKWKKEN	Homeobox protein Hox-B7 (HOXB7)
DH_512	Rattus norvegicus	RKRGRQTYTRYQTLELEKEFHYNRYLTRRRRIEIAHALCLTERQIKIWFQNRRMKWKKEN	Homeobox protein Hox-B7 (Hoxb7)
DH_6815	Gallus gallus	RKRGRQTYTRYQTLELEKEFHYNRYLTRRRRIEIAHALCLTERQIKIWFQNRRMKWKKEN	Hoxb-7 (Fragment) (Hoxb-7)
DH_8013	Latimeria menadoensis	RKRGRQTYTRYQTLELEKEFHYNRYLTRRRRIEIAHALCLTERQIKIWFQNRRMKWKKEN	HoxB7 (Fragment)
DH_263	Apis mellifera	RKRGRQTYTRYQTLELEKEFHYNRYLTRRRRIEIAHALCLTERQIKIWFQNRRMKWKKEN	Homeobox protein H90 (Fragment)
DH_8048	Rattus sp	RKRGRQTYTRYQTLELEKEFHYNRYLTRRRRIEIAHALCLTERQIKIWFQNRRMKWKKEN	Hox-B|Hox-2 (Fragment) (Hox-B|Hox-2)
DH_7649	Perionyx excavatus	RKRGRQTYTRYQTLELEKEFHYNRYLTRRRRIEIAHALALTERQIKIWFQNRR-------	Homeobox protein Antp (Fragment)
DH_4736	Manduca sexta	RKRGRQTYTRYQTLELEKEFHFNRYLTRRRRIEIAH-LPLTERQIKIWFQNRRMKWKKEN	ANTP (Fragment) (Antp)
DH_5005	Nereis virens	RKRGRQTYTRYQTLELEKEFHFNRYLTRRRRIEIAHSLCLTERQIKIWFQNRRMKWKKEN	Hox7 homeobox protein (Fragment) (Hox7)
DH_514	Xenopus laevis	RKRGRQTYTRYQTLELEKEFHFNRYLTRRRRIEIAHVLCLTERQIKIWFQNRRMKWKKEN	Homeobox protein Hox-B7-A (hoxb7-A)
DH_6729	Homo sapiens	RKRGRQTYTRYQTLELEKEFHYNRYLTRRRRIEIAHTLCLTERQIKIWFQNRRMKWKKEN	Homeo box B7 variant (Fragment)
DH_9006	Homo sapiens	RKRGRQTYTRYQTLELEKEFHYNRYLTRRRRIEIAHTLCLTERQIKIWFQNRRMKWKKEN	Homeobox B7 (HOXB7)
DH_510	Homo sapiens	RKRGRQTYTRYQTLELEKEFHYNRYLTRRRRIEIAHTLCLTERQIKIWFQNRRMKWKKEN	Homeobox protein Hox-B7 (HOXB7)
DH_2198	Homo sapiens	RKRGRQTYTRYQTLELEKEFHYNRYLTRRRRIEIAHTLCLTERQIKIWFQNRRMKWKKEN	cDNA FLJ76026, highly similar to Homo sapiens homeo box B7 (HOXB7), mRNA
DH_511	Mus musculus	RKRGRQTYTRYQTLELEKEFHYNRYLTRRRRIEIAHTLCLTERQIKIWFQNRRMKWKKEN	Homeobox protein Hox-B7 (Hoxb7)
DH_1420	Mus musculus	RKRGRQTYTRYQTLELEKEFHYNRYLTRRRRIEIAHTLCLTERQIKIWFQNRRMKWKKEN	Homeo box B7 (Hoxb7)
DH_515	Xenopus laevis	RKRGRQTYTRYQTLELEKEFHFNRYLTRRRRIEIAHTLCLTERQIKIWFQNRRMKWKKEN	Homeobox protein Hox-B7-B (hoxb7-B)
DH_6366	Xenopus laevis	RKRGRQTYTRYQTLELEKEFHFNRYLTRRRRIEIAHTLCLTERQIKIWFQNRRMKWKKEN	Hoxb7 protein (Hoxb7)
DH_5201	Procambarus clarkii	RKRGRQTYTRYQTLELEKEFHFNRYLTRRRRIEIAHALCLTERQIKIWFQDRRMKWKKEN	Antennapedia (Antp)
DH_5203	Procambarus clarkii	RKRGRQTYTRYQTLELEKEFHFNRYLTRRRRIEIAHALCLTERQIKIWFQDRRMKWKKEN	Antennapedia (Antp)
DH_6810	Trionyx sinensis	RKRGRQTYTRYQTLKLEKEFHFNRYLTRRRRIEIAHALCLTERQIKIWFQKA--------	Hoxa-7 (Fragment) (Hoxa-7)
DH_8973	Procambarus clarkii	RKRGRQTYTRYQTLELEKEFHFNRYLTRRRRIEIAHALCLTERQIKIW------------	Antp protein (Fragment)
DH_9508	Thermobia domestica	RKRGRQTYTRYQTLELEKEFHFNRYLTRRRRIEIAHALCLTERQIKI-------------	Antennapedia protein (Fragment) (antp)
DH_11531	Branchiostoma floridae	RKRGRQTYTRYQTLELEKEFHFNKYLTRRRRIEIAHALCLTERQIKIWFQNRRMKWKKEN	e_gw.260.32.1|Brafl1
DH_11589	Branchiostoma floridae	RKRGRQTYTRYQTLELEKEFHFNKYLTRRRRIEIAHALCLTERQIKIWFQNRRMKWKKEN	e_gw.260.32.1|Brafl1
DH_2432	Branchiostoma floridae	RKRGRQTYTRYQTLELEKEFHFNKYLTRRRRIEIAHALCLTERQIKIWFQNRRMKWKKEN	AmphiHox7 (AmphiHox7)
DH_12683	Branchiostoma floridae	RKRGRQTYTRYQTLELEKEFHFNKYLTRRRRIEIAHALCLTERQIKIWFQNRRMKWKKEN	AmphiHox7
DH_11545	Branchiostoma floridae	RKRGRQTYTRYQTLELEKEFHFNKYLTRRRRIEIAHALCLTERQIKIWFQNRRMKWKKEN	e_gw.402.27.1|Brafl1
DH_5293	Branchiostoma floridae	RKRGRQTYTRYQTLELEKEFHFNKYLTRRRRIEIAHALCLTERQIKIWFQNRRMKWKKEN	Hox-7 homeodomain protein (Fragment)
DH_4550	Lineus sanguineus	RKRGRQTYTRYQTLELEKEFHFNKYLTRRRRIEIAHALCLTERQIKIWFQNRRMKWKKKE	Homeodomain protein (Fragment) (Hox-7)
DH_5597	Junonia coenia	RKRGRQTYTRYQTLELEKEFHFNRYLTRRRRIEIAHALCLTERQIKIWFQNRRMKWKK--	Antennapedia protein (Fragment) (antennapedia)
DH_2431	Branchiostoma floridae	RRRGRQTYSRYQTLELEKEFHFNKYLTRRRRIEIAHALGLTERQIKIWFQNRRMKLKKEA	AmphiHox8 (AmphiHox8)
DH_12684	Branchiostoma floridae	RRRGRQTYSRYQTLELEKEFHFNKYLTRRRRIEIAHALGLTERQIKIWFQNRRMKLKKEA	AmphiHox8
DH_5294	Branchiostoma floridae	RRRGRQTYSRYQTLELEKEFHFNKYLTRRRRIEIAHALGLTERQIKIWFQNRRMKLKKEA	Hox-8 homeodomain protein (Fragment)
DH_11639	Branchiostoma floridae	RRRGRQTYSRYQTLELEKEFHFNKYLTRRRRIEIAHALGLTERQIKIWFQNRRMKLKKEA	e_gw.402.19.1|Brafl1
DH_9480	Nereis virens	EERGRQTYSRYQTLELEKEFRFNHYLTRKRRMEIAHVLCLTEHQIKIWFQNRR-------	Lox4 homeobox protein (Fragment) (Lox4)
DH_9510	Thermobia domestica	RRRGRQTYTRFQTLELEKEFHFNHYLTRRRRIEIAHALCLPERQIKI-------------	Abdominal-A protein (Fragment) (abd-A)
DH_5834	Artemia sanfranciscana	RRRGRQTSTRHQTLELEKEFHFNHYLTRRRRIEIAHALCLSERQIKIWFQNRRMKLKKEL	Abdominal-A (Fragment) (abdA)
DH_5831	Artemia sp. MaDuo	RRRGAQTYTRYQSLELEKEFHFIHYLTRRRRIEIAHALCLTERQIKIWFQNRRMKLKKEL	Abdominal-A (Fragment) (abdA)
DH_4486	Acanthokara kaputensis	RKRGRQTYTRYQTLELEKEFHTNHYLTRRRRIEMAHALCLTERQIKIWFQNRRMKLKKEM	Ultrabithorax (Ubx)
DH_2762	Flaccisagitta enflata	RKRGRQTYTRYQTLELEKEFHFNRYLTRRRRIEMAHALCLTERQIKIWFQNRRMKEKKEK	Hox8
DH_5204	Porcellio scaber	RKRGRQTYTRYQTLELEKEFHFNRYLTRRRRIEIAHALCLTERQIKIWFQNRRMKC----	Antennapedia (Fragment) (Antp)
DH_7004	Urechis unicinctus	RKRGRQTYTRYQTLELEKEFHFNRYLTRRRRIEIAHALCLTERQIKIWFQNRR-------	Homeobox protein Antp (Fragment)
DH_12161	Lottia gigantea	RKRGRQTYTRYQTLELEKEFHFNRYLTRRRRIEIAHALCLTERQIKIWFQNRRMKWKKEA	fgenesh2_pm.C_sca_12000048|Lotgi1
DH_4604	Archegozetes longisetosus	RKRGRQTYTRYQTLELEKEFHFNRYLTRRRRIEIAHALCLTERQIKIWFQNRRMKWKKEI	Antennapedia (Fragment) (Antp)
DH_423	Heterodontus francisci	RKRGRQTYTRYQTLELEKEFHFNRYLTRRRRIEIAHALCLTERQIKIWFQNRRMKWKKET	Homeobox protein Hox-A7 (HOXA7)

```
DH_2761  Flaccisagitta enflata                     RKRGRQTYTRYQTLELEKEFHFNRYLTRRRRIDIAHALCLTERQIKIWFQNRRMKWKKEQ Hox6
DH_3296  Salmo salar                               RKRGRQTYSRYQTLELEKEFHFNRYLNRRRRVEIAHVLCLTERQIKIWFQNRRMKWKKDH Homeobox protein HoxA7ab (HoxA7ab)
DH_3338  Salmo salar                               RKRGRQTYPRYQTLELEKEFHFNRYLNRRRRVEIAHVLCLTERQIKIWFQNRRMKWKKDH Homeobox protein HoxA7ab (HoxA7ab)
DH_3286  Salmo salar                               RKRGRQTYSRYQTLEMEKEFHFNRYLTRRRRVEIAQVLCLTERQIKIWFQNRRMKWKKDH Homeobox protein HoxA7aa (HoxA7aa)
DH_6185  Oryzias latipes                           RKRGRQTYSRYQTLELEKEFHFNRYLSRRRRVEIAHALTLTERQIKIWFQNRRMKWKKDH HoxA7a (hoxA7a)
DH_430   Salmo salar                               RKRGSQTYTRYQTLELEKEFHFNRYLTRRRRVEIAHVLCLTERQIKIWFQNRRMKWKKDH Homeobox protein Hox-A7 (Fragment) (hoxa7)
DH_6864  Pecten maximus                            RKRGRRTYTRYQTLELEKEFHFNKYLTRRRRVEIAHVLCLTERQIKIWFQSRRMKWKKEA Homeodomain protein Antp (Fragment) (antp)
DH_429   Rattus norvegicus                         GKRGRQTYTRYQTLELEKEFHFNRYLTRRRAVEIAHALCLTERQIKIWFQNRRMKWKKEH Homeobox protein Hox-A7 (Fragment) (Hoxa7)
DH_8502  Petromyzon marinus                        RRRGRQTYSRYQTLELEKEFHFNRYLTRRRRIEIAHALCLTERQIKIWFQNRRMKWKKEH HoxN6 homeobox (Fragment)
DH_8487  Lampetra fluviatilis                      RRRGRQTYSRYQTLELEKEFHFNRYLTRRRRIEIAHALCLTERQIKIWFQNRRMKWKKEH Homeobox protein HoxL6 (Fragment)
DH_9405  Petromyzon marinus                        RRRGRQTYSRYQTLELEKEFHFNRYLTRRRRIEIAHALCLTERQIKIWFQNRRMKWKKEH Homeobox protein (Fragment) (hoxw6)
DH_7515  Lampetra japonica                         RRRGRQTYSRYQTLELEKEFHFNRYLTRRRRIEIAHALCLTERQIKIWFQNRRMKWKKEH LjHox6w Homeobox (Fragment) (LjHox6w)
DH_8501  Petromyzon marinus                        RRRGRQTYSRYQTLELEKEFHFNRYLTRRRRIEIAHALCLTERQIKIWFQNRRMKWKKEH HoxN7 homeobox (Fragment)
DH_5528  Ctenodrilus serratus                      --------------------HFNRYLTRRRRIEIAHALCLTERQIKIWFQNRRMKWKKEH Deformed ortholog homeobox (Fragment) (CTs-Dfd)
DH_5664  Ctenodrilus serratus                      --------------------HFNRYLTRRRRIEIAHALCLTERQIKIWFQNRRMKWKKEH CTs-Dfd protein (Fragment) (CTs-Dfd)
DH_5598  Junonia coenia                            ------------------EFHFNRYLTRRRRIEIAHALCLTERQIKIWFQNRRMKWKKEH Sex combs reduced homeodomain protein (Fragment)
DH_7360  Nasonia vitripennis                       ------------------EFHFNRYLTRRRRIEIAHALCLTERQIKIWFQNRRMKWKKEH Sex combs reduced (Fragment) (scr)
DH_8025  Latimeria menadoensis                     KKRGRQTYTRYQTLELEKEFHFNRYLTRRRRIEIAHALCLTERQIKIWFQNRRMKWKKEH HoxA7 (Fragment)
DH_421   Gallus gallus                             RKRGRQTYTRYQTLELEKEFHFNRYLTRRRRIEIAHALCLTERQIKIWFQNRRMKWKKEH Homeobox protein Hox-A7 (HOXA7)
DH_424   Homo sapiens                              RKRGRQTYTRYQTLELEKEFHFNRYLTRRRRIEIAHALCLTERQIKIWFQNRRMKWKKEH Homeobox protein Hox-A7 (HOXA7)
DH_1692  Homo sapiens                              RKRGRQTYTRYQTLELEKEFHFNRYLTRRRRIEIAHALCLTERQIKIWFQNRRMKWKKEH Homeo box A7 (HOXA7)
DH_428   Pan troglodytes                           RKRGRQTYTRYQTLELEKEFHFNRYLTRRRRIEIAHALCLTERQIKIWFQNRRMKWKKEH Homeobox protein Hox-A7 (HOXA7)
DH_2447  Papio anubis                              RKRGRQTYTRYQTLELEKEFHFNRYLTRRRRIEIAHALCLTERQIKIWFQNRRMKWKKEH Homeobox A7 (Predicted) (HOXA7)
DH_422   Coturnix coturnix japonica                RKRGRQTYTRYQTLELEKEFHFNRYLTRRRRIEYAHALCLTERQIKIWFQNRRMKWKKEH Homeobox protein Hox-A7 (HOXA7)
DH_427   Pan paniscus                              RKRGRQTYTRYQTLELEKEFHFNRYLTRRRRIEXXHALCLTERQIXXWFQNRRMKWKKEH Homeobox protein Hox-A7 (HOXA7)
DH_2753  Callicebus moloch                         RKRGRQTYTRYQTLELEKEFHFNRYLTRRRRIEIAHALCLTERQIKIWFQNRRMKWKKEH Homeobox A7 (Predicted) (HOXA7)
DH_2811  Rhinolophus ferrumequinum                 RKRGRQTYTRYQTLELEKEFHFNRYLTRRRRIEIAHALCLTERQIKIWFQNRRMKWKKEH Homeobox protein Hox-A7 (Predicted) (HOXA7)
DH_2577  Callithrix jacchus                        RKRGRQTYTRYQTLELEKEFHFNRYLTRRRRIEIAHALCLTERQIKIWFQNRRMKWKKEH Homeobox A7 (Predicted) (HOXA7)
DH_432   Xenopus laevis                            RKRGRQTYTRYQTLELEKEFHFNRYLTRRRRIEIAHALCLTERQIKIWFQNRRMKWKKEH Homeobox protein Hox-A7 (hoxa7)
DH_431   Ovis aries                                RKRGRQTYTRYQTLELEKEFHFNRYLTRRRRIEIAHALCLTERQIKIWFQNRRMKWKKEH Homeobox protein Hox-A7 (Fragment) (HOXA7)
DH_426   Mus musculus                              RKRGRQTYTRYQTLELEKEFHFNRYLTRRRRIEIAHALCLTERQIKIWFQNRRMKWKKEH Homeobox protein Hox-A7 (Hoxa7)
DH_1567  Mus musculus                              RKRGRQTYTRYQTLELEKEFHFNRYLTRRRRIEIAHALCLTERQIKIWFQNRRMKWKKEH Homeo box A7 (Hoxa7)
DH_8406  Mus musculus                              RKRGRQTYTRYQTLELEKEFHFNRYLTRRRRIEIAHALCLTERQIKIWFQNRRMKWKKEH Hoxa7 protein (Hoxa7)
DH_8051  Rattus sp                                 RKRGRQTYTRYQTLELEKEFHFNRYLTRRRRIEIAHALCLTERQIKIWFQNRRMKWKKEH Hox-A|Hox-1 (Fragment) (Hox-A|Hox-1)
DH_9630  Priapulus caudatus                        RKRGRQTYTRYQTLELEKEFHFNRYLTRRRRIEIANMLCLTERQIKIWFQNRRMKWKKEC HB2 homeodomain protein (Fragment) (HB2)
DH_12574 Strongylocentrotus purpuratus             RKRGRQTYTRAQTLELEKEFHYNRYLTRKRRIEIAQAVCLSERQIKIWFQNRRMKWKKER GLEAN3_02630|Sp-Hox8
DH_8867  Heliocidaris erythrogramma                RKRGRQTYTRAQTLELEKEFHYNRYLTRKRRIEIAQAVCLSERQIKIWFQNRRMKWKKER HEHBOX6 (Fragment) (HeHbox6)
DH_12606 Strongylocentrotus purpuratus             RKRGRQTYTRAQTLELEKEFHYNRYLTRKRRIEIAQAVCLSERQIKIWFQNRRMKWKKER GLEAN3_21309|Sp-Hox8
DH_7822  Tripneustes gratilla                      RKRGRQTYTRAQTLELEKEFHYNRYLTRKRRIEIAQAVCLSERQIKIWFQNRRMKWKKER Homeotic protein TgHbox 6 (Fragment)
DH_1328  Metacrinus rotundus                       KKRGRQTYTRYQTLELEKEFHFNRYLTRRRRIEIAQAVCLSERQIKIWFQNRRMKAKKET Transcription factor Hox8 (MrHox8)
DH_11535 Branchiostoma floridae                    KKRGRQTYTRYQTLELEKEFHFNKYLTRKRRIEIAHLLGLTERQIKIWFQNRRMKWKKEN fgenesh2_pg.scaffold_260000039|Brafl1
DH_12682 Branchiostoma floridae                    KKRGRQTYTRYQTLELEKEFHFNKYLTRKRRIEIAHLLGLTERQIKIWFQNRRMKWKKEN AmphiHox6
DH_2433  Branchiostoma floridae                    KKRGRQTYTRYQTLELEKEFHFNKYLTRKRRIEIAHLLGLTERQIKIWFQNRRMKWKKEN AmphiHox6 (AmphiHox6)
DH_5292  Branchiostoma floridae                    KKRGRQTYTRYQTLELEKEFHFNKYLTRKRRIEIAHLLGLTERQIKIWFQNRRMKWKKEN Hox-6 homeodomain protein (Fragment)
DH_265   Strongylocentrotus purpuratus             RKRCRQTYTRYQTLELEKEFHFNRYLTRRRRIELSHLLGLTERQIKIWFQNRRMKYKKES Homeobox protein HB1
DH_266   Tripneustes gratilla                      RKRCRQTYTRYQTLELEKEFHFNRYLTRRRRIELSHLLGLTERQIKIWFQNRRMKYKKES Homeobox protein HB1 (Fragment)
DH_8866  Heliocidaris erythrogramma                RKRCRQTYTRYQTLELEKEFHFNRYLTRRRRIELSHLLGLTERQIKIWFQNRRMKYKKES Hox-type homeodomain protein (Fragment) (HeHbox1)
DH_12572 Strongylocentrotus purpuratus             RKRCRQTYTRYQTLELEKEFHFNRYLTRRRRIELSHLLGLTERQIKIWFQNRRMKYKKES GLEAN3_05170|Sp-Hox7
DH_7935  Saccoglossus kowalevskii                  KKRCRQTYTRYQTLELEKEFHYNRYLTRRRRIELSHLLGLTERQIKIWFQNRRMKYKKES Hox 7
DH_1327  Metacrinus rotundus                       RKRCRQTYTRYQTLELEKEFHFNRYLTRRRRIELSHLLGLTERQIKIWFQNRRMKYKKEN Transcription factor Hox7 (MrHox7)
DH_1775  Xenoturbella bocki                        RKRGRQTYTRYQTLELEKEFHYNQYLTRRRRIEIATGLGLTERQI--------------- HoxM2 protein (Fragment) (hoxM2)
DH_11527 Daphnia pulex                             PKRTRQTYTRYQTLELEKEFHFNRYLTRRRRVEIAHALCLTERQIKIWFQNRRMKAKKET YAS_Hox_Ftz|Dappu1
DH_1797  Daphnia pulex                             PKRTRQTYTRYQTLELEKEFHFNRYLTRRRRVEIAHALCLTERQ---------------- Homeodomain transcription factor ftz-like protein (Fragment)
DH_11436 Daphnia pulex                             PKRTRQTYTRYQTLELEKEFHFNRYLTRRRRVEIAHALCLTERQIKIWFQNRRMKAKKET gw1.7.523.1|Dappu1
DH_4476  Ethmostigmus rubripes                     PKRTRQTYTRFQTLELEKEFHFNRYLTRRRRIEIAHSLGLTERQIKI------------- Fushi tarazu (Fragment) (ftz)
DH_9262  Pachymerium ferrugineum                   PKRTRQTYTRFQTLELEKEFHFNRYLTRRRRIEIAHTLGLTERQIKIW------------ Putative fushi-tarazu (Fragment) (ftz)
DH_9284  Archegozetes longisetosus                 PKRTRQTYTRYQTLELEKEFHFNRYLTRRRRIEIAHSLGLTERQIKIWFQNRRMKAKKEN Fushi tarazu-like protein (Fragment) (ftz)
DH_4482  Acanthokara kaputensis                    PKRTRQTYTRYQTLELEKEFHFNRYLTRRRRIEIAHALGLTERQIK-------------- Fushi tarazu (Fragment) (ftz)
DH_2470  Endeis spinosa                            PKRTRQTYTRYQTLELEKEFHFNRYLTRRRRIEIAHALGLSERQIKIWFQNRRMKAKKEN Homeobox protein fushi tarazu (Ftz)
DH_5999  Cupiennius salei                          PKRSRQTYTRYQTLELEKEFHFNQYLTRRRRIEIAHTLGLTERQIKIWFQNRRMKAKKEN Fushi tarazu (Fragment) (ftz)
DH_11992 Capitella sp. I Grassle and Grassle, 1976 QKRTRQTYTRYQTLELEKEFHYNRYLTRRRRIEIAHALQLTERQIKIWFQNRRMKYKKEN estExt_Genewise1Plus.C_700050|Capca1
DH_5008  Platynereis dumerilii                     QKRTRQTYTRYQTLELEKEFHYNRYLTRRRRIEIAHA----------------------- Lox5 homeobox protein (Fragment) (Lox5)
DH_9478  Nereis virens                             QKRTRQTYTRYQTLELEKEFHYNRYLTRRRRIEIAHALGLTERQIKIWFQNRRMKWKKEN Lox5 homeobox protein (Fragment) (Lox5)
DH_9491  Lingula unguis                            QKRTRQTYTRYQTLELEKEFHYNRYLTRRRRIEIAHHLGLTERQIKIWFQNRRMKWKKEN Lox5 homeodomain protein (Fragment)
DH_7444  Bugula turrita                            QKRTRQTYTRYQTLELEKEFHYNRYLTRRRRIEIAHTLGLTERQIKIWFQNRRMKWKKEN Hox class homeodomain-containing protein Lox5 (Fragment)
DH_7648  Perionyx excavatus                        QKRTRQTYTRYQTLELEKEFHHNRYLTRRRRIEIAHALALTERQIKIWFQNRR------- Homeobox protein Lox5 (Fragment)
```

DH_2760	Flaccisagitta enflata	QKRTRQTYTRHQTLELEKEFHFNRYLTRRRRIEIVHALGLTERQIKIWFQNRRMKWKKEN	Hox5 (Fragment)
DH_8080	Spadella cephaloptera	QKRTRQTYTRHQTLELEKEFHFNRYLTRRRRIEIAHALGLTERQIKIWFQNR--------	Hox median 2 protein (Fragment)
DH_4549	Lineus sanguineus	QKRTRQTYTRYQTLELEKEFHFNKYLTRRRRIEIAHALGLTERQIKIWFQNRRMKWKKEN	Homeodomain protein (Fragment) (Hox-6)
DH_12246	Lottia gigantea	QKRTRQTYTRYQTLELEKEFHFNRYLTRRRRIEVAHMLGLTERQIKIWFQNRRMKWKKEN	gw1.12.445.1|Lotgi1
DH_4598	Dugesia tigrina	NKRTRQTYTRHQTLELEKEFHFNKYLTRRRRIEIAHTLILTERQIKIWFQNRRMKWKKEH	Homeodomain protein (DthoxC)
DH_9595	Dugesia japonica	NKRTRQTYTRHQTLELEKEFHFNKYLTRRRRIEIAHSLILTERQIKIWFQNRRMKWKKEH	PLOX5-Dj (Fragment) (Plox5-Dj)
DH_1117	Schistosoma mansoni	QKRTRQTYTRYQTLELEKEFHFNKYLTRRRRIEIAHTLTLTERQIKIWFQNR--------	Homeobox protein SMOX-1 (Fragment) (SMOX-1)
DH_6973	Schistosoma mansoni	QKRTRQTYTRYQTLELEKEFHFNKYLTRRRRIEIAHTLTLTERQIKIWFQNRRMKWKKDH	Hox protein Smox1
DH_4600	Dugesia tigrina	HKRSRQTYTRYQTLELEKEFHFNKYLTRRRRIEIAHALSLTERQIKIWFQNRRMKWKKDH	DthoxE protein (DthoxE)
DH_9596	Dugesia japonica	HKRSRQTYTRYQTLELEKEFHFNKYLTRRRRIEIAHALSLTERQIKIWFQNRRMKWKKDH	PLOX6-Dj (Fragment) (Plox6-Dj)
DH_5554	Dugesia tigrina	HKRSRQTYTRYQTLELEKEFHFNKYLTRRRRIEIAHGLSLTERQIKIWFQNRRMKWKKDH	DNA-binding protein (Fragment) (homeobox)
DH_175	Drosophila hydei	SKRTRQTYSRYQTLELEKEFHFNRYITRRRRMDIAHALNLSERQIKIWFQNRRMKSKKDR	Segmentation protein fushi tarazu (ftz)
DH_3746	Drosophila mojavensis	SKRTRQTYSRYQTLELEKEFHFNRYITRRRRMDIAHALNLSERQIKIWFQNRRMKSKKDR	GI24714 (GI24714)
DH_3898	Drosophila virilis	SKRTRQTYSRYQTLELEKEFHFNRYITRRRRMDIAHALNLSERQIKIWFQNRRMKSKKDR	GJ23928 (GJ23928)
DH_8337	Drosophila virilis	SKRTRQTYSRYQTLELEKEFHFNRYITRRRRMDIAHALNLSERQIKIWFQNRRMKSKKDR	Ftz-PA (ftz)
DH_3679	Drosophila grimshawi	SKRTRQTYSRYQTLELEKEFHFNRYITRRRRIDIAHALSLSERQIKIWFQNRRMKSKKDR	GH19476 (GH19476)
DH_2518	Drosophila mauritiana	SKRTRQTYTRYQTLELEKEFHFNRYITRRRRIDIANALSLSERQIKIWFQNRRMKSKKDR	Ftz (ftz)
DH_2520	Drosophila mauritiana	SKRTRQTYTRYQTLELEKEFHFNRYITRRRRIDIANALSLSERQIKIWFQNRRMKSKKDR	Ftz (ftz)
DH_2521	Drosophila mauritiana	SKRTRQTYTRYQTLELEKEFHFNRYITRRRRIDIANALSLSERQIKIWFQNRRMKSKKDR	Ftz (ftz)
DH_2519	Drosophila mauritiana	SKRTRQTYTRYQTLELEKEFHFNRYITRRRRIDIANALSLSERQIKIWFQNRRMKSKKDR	Ftz (ftz)
DH_2876	Drosophila simulans	SKRTRQTYTRYQTLELEKEFHFNRYITRRRRIDIANALSLSERQIKIWFQNRRMKSKKDR	FTZ (ftz)
DH_2877	Drosophila simulans	SKRTRQTYTRYQTLELEKEFHFNRYITRRRRIDIANALSLSERQIKIWFQNRRMKSKKDR	FTZ (ftz)
DH_4234	Drosophila simulans	SKRTRQTYTRYQTLELEKEFHFNRYITRRRRIDIANALSLSERQIKIWFQNRRMKSKKDR	Ftz (ftz)
DH_2878	Drosophila simulans	SKRTRQTYTRYQTLELEKEFHFNRYITRRRRIDIANALSLSERQIKIWFQNRRMKSKKDR	FTZ (ftz)
DH_2880	Drosophila sechellia	SKRTRQTYTRYQTLELEKEFHFNRYITRRRRIDIANALSLSERQIKIWFQNRRMKSKKDR	FTZ (ftz)
DH_176	Drosophila melanogaster	SKRTRQTYTRYQTLELEKEFHFNRYITRRRRIDIANALSLSERQIKIWFQNRRMKSKKDR	Segmentation protein fushi tarazu (ftz)
DH_3173	Drosophila erecta	SKRTRQTYTRYQTLELEKEFHFNRYITRRRRIDIANALSLSERQIKIWFQNRRMKSKKDR	Ftz (Dere\ftz)
DH_8344	Drosophila erecta	SKRTRQTYTRYQTLELEKEFHFNRYITRRRRIDIANALSLSERQIKIWFQNRRMKSKKDR	Ftz-PA (ftz)
DH_4142	Drosophila yakuba	SKRTRQTYTRYQTLELEKEFHFNRYITRRRRIDIANALSLSERQIKIWFQNRRMKSKKDR	GE24884 (GE24884)
DH_2998	Drosophila ananassae	SKRTRQTYTRYQTLELEKEFHFNRYITRRRRIDIANALSLSERQIKIWFQNRRMKSKKDR	GF17190 (GF17190)
DH_3459	Drosophila persimilis	SKRTRQTYTRYQTLELEKEFHFNRYITRRRRVDIANALSLSERQIKIWFQNRRMKSKKDR	GL21778 (GL21778)
DH_8341	Drosophila pseudoobscura pseudoobscura	SKRTRQTYTRYQTLELEKEFHFNRYITRRRRVDIANALSLSERQIKIWFQNRRMKSKKDR	Ftz-PA (ftz)
DH_5759	Drosophila pseudoobscura pseudoobscura	SKRTRQTYTRYQTLELEKEFHFNRYITRRRRVDIANALSLSERQIKIWFQNRRMKSKKDR	Ftz (ftz)
DH_6765	Drosophila pseudoobscura pseudoobscura	SKRTRQTYTRYQTLELEKEFHFNRYITRRRRVDIANALSLSERQIKIWFQNRRMK-----	Fushi tarazu (Fragment) (ftz)
DH_6766	Drosophila miranda	SKRTRQTYTRYQTLELEKEFHFNRYITRRRRVDIANALSLSERQIKIWFQNRRMK-----	Fushi tarazu (Fragment) (ftz)
DH_6767	Drosophila affinis	SKRTRQTYTRYQTLELEKEFHFNRYITRRRRVDIANALSLSERQIKIWFQNRRMK-----	Fushi tarazu (Fragment) (ftz)
DH_4017	Drosophila willistoni	SKRTRQTYTRYQTLELEKEFHFNRYITRRRRIDIAHALSLTERQIKIWFQNRRMKSKKDR	GK10825 (GK10825)
DH_12589	Strongylocentrotus purpuratus	GKRGRQTYTRQQTLELEKEFHFSRYVTRRRRFEIAQSLGLSERQIKIWFQNRRMKWKREH	GLEAN3_05171|Sp-Hox6
DH_267	Tripneustes gratilla	GKRGRQTYTRQQTLELEKEFHFSRYVTRRRRFEIAQSLGLSERQIKIWFQNRRMKWKREH	Homeobox protein HB3 (Fragment)
DH_5038	Glomeris marginata	RKRTRQTYTRFQTLELEKEFHSNRYLNRRRRIEIATSLTLTERQVKIWFQNRRMKAKREP	Fushi tarazu (ftz)
DH_9472	Dicyema orientale	HKRTRQTYTRSQTLELEKEFHYNKYLSKRRRTEISEVLELSERQVKIWFQNRRMKWKKDN	DoxC (DoxC)

Save alignment [fasta format] - Save complete sequences [fasta format]

PG8

ID	organism	10 20 30 40 50 60	description
DH_3324	Salmo salar	RRRGRQAYSRFQTLELEKEFLFNSYLTRKRKVEVSHALALTERQIKIWFQNRRMKWKKEN	Homeobox protein HoxB8ba (HoxB8ba)
DH_3330	Salmo salar	RRRGRQAYSRFQTLELEKEFLFNSYLTRKRKVEVSHALALTERQIKIWFQNRRMKWKKEN	Homeobox protein HoxB8ba2 (Homeobox protein HoxB8ba) (HoxB8ba)
DH_8001	Latimeria menadoensis	RRRGRQTYSRFQTLELEKEFLFNPYLTRKRRIEVSHSLGLTERQIKIWFQNRRMKWKKEN	HoxD8 (Fragment)
DH_631	Gallus gallus	RRRGRQTYSRFQTLELEKEFLFNPYLTRKRRIEVSHALGLTERQVKIWFQNRRMKWKKEN	Homeobox protein Hox-D8 (HOXD8)
DH_3427	Xenopus tropicalis	RRRGRQTYSRFQTLELEKEFLFNPYLTRKRRIEVSHALGLTERQVKIWFQNRRMKWKKEN	Putative uncharacterized protein
DH_7569	Xenopus laevis	RRRGRQTYSRFQTLELEKEFLFNPYLTRKRRIEVSHALGLTERQVKIWFQNRRMKWKKEN	MGC68588 protein (hoxd8)
DH_632	Heterodontus francisci	RRRGRQTYSRFQTLELEKEFLFNPYLTRKRRIEVSHALGLTERQVKIWFQNRRMKWKKEN	Homeobox protein Hox-D8 (HOXD8)
DH_4621	Ambystoma mexicanum	RRRGRQTYSRFQTLELEKEFLFNPYLTRKRRIEVSHALGLTERQVKIWFQNRRMKWKKEN	Homeobox transcription factor (Fragment) (Hoxd-8)
DH_7516	Lampetra japonica	RRRGRQTYSRFQTLELEKEFLFNPYLTRKRRIEVSHALGLTERQVKIWFQNRRMKWKKEN	LjHoxQ8 Homeobox (Fragment) (LjHoxQ8)
DH_9353	Petromyzon marinus	RRRGRQTYSRFQTLELEKEFLFNPYLTRKRRIEVSHALGLTERQVKIWFQNRRMKWKKEN	Homeobox protein HoxQ8 (HoxQ8)
DH_9354	Petromyzon marinus	RRRGRQTYSRFQTLELEKEFLFNPYLTRKRRIEVSHALGLTERQVKIWFQNRRMKWKKEN	Homeobox protein HoxQ8a (Fragment) (HoxQ8a)
DH_523	Danio rerio	RRRGRQTYSRYQTLELEKEFLFNPYLTRKRRIEVSHALALTERQVKIWFQNRRMKWKKEH	Homeobox protein Hox-B8b (hoxb8b)
DH_2889	Danio rerio	RRRGRQTYSRYQTLELEKEFLFNPYLTRKRRIEVSHALALTERQVKIWFQNRRMKWKKEH	Homeo box B8b (hoxb8b)
DH_1543	Megalobrama amblycephala	RRRGRRTYSRYQTLELEKEFLFNPYLTRKRRIEVSHALALTERQVKIWFQNRRMKWKKEH	Homeodomain protein (Fragment) (HoxB8b)
DH_9074	Mus musculus	RRRGRQTYSRFQTLELEKEFLFNPYLTRKRRIEVSHTLALTERQVKIWFQNRRMKWKKEN	Putative uncharacterized protein (Fragment) (Hoxd8)
DH_9075	Mus musculus	RRRGRQTYSRFQTLELGKEFLFYPYLTRKRRIEVSHTLALTERQVKLWF-----------	Putative uncharacterized protein (Fragment) (Hoxd8)
DH_1445	Mus musculus	RRRGRQTYSRFQTLELEKEFLFNPYLTRKRRIEVSHTLALTERQVKIWFQNRRMKWKKEN	Homeo box D8 (Hoxd8)
DH_1444	Mus musculus	RRRGRQTYSRFQTLELEKEFLFNPYLTRKRRIEVSHTLALTERQVKIWFQNRRMKWKKEN	Homeo box D8 (Hoxd8)
DH_634	Mus musculus	RRRGRQTYSRFQTLELEKEFLFNPYLTRKRRIEVSHTLALTERQVKIWFQNRRMKWKKEN	Homeobox protein Hox-D8 (Hoxd8)
DH_1443	Mus musculus	RRRGRQTYSRFQTLELEKEFLFNPYLTRKRRIEVSHTLALTERQVKIWFQNRRMKWKKEN	Homeo box D8 (Fragment) (Hoxd8)
DH_633	Homo sapiens	RRRGRQTYSRFQTLELEKEFLFNPYLTRKRRIEVSHALALTERQVKIWFQNRRMKWKKEN	Homeobox protein Hox-D8 (HOXD8)
DH_8383	Homo sapiens	RRRGRQTYSRFQTLELEKEFLFNPYLTRKRRIEVSHALALTERQVKIWFQNRRMKWKKEN	Homeobox D8 (HOXD8)
DH_6960	Homo sapiens	RRRGRQTYSRFQTLELEKEFLFNPYLTRKRRIEVSHALALTERQVKIWFQNRRMKWKKEN	HOXD8 protein (HOXD8)
DH_635	Pan troglodytes	RRRGRQTYSRFQTLELEKEFLFNPYLTRKRRIEVSHALALTERQVKIWFQNRRMKWKKEN	Homeobox protein Hox-D8 (HOXD8)
DH_1362	Gorilla gorilla gorilla	RRRGRQTYSRFQTLELEKEFLFNPYLTRKRRIEVSHALALTERQVKIWFQNRRMKWKKEN	HOXD8 (Fragment) (HOXD8)
DH_1374	Saguinus labiatus	RRRGRQTYSRFQTLELEKEFLFNPYLTRKRRIEVSHALALTERQVKIWFQNRRMKWKKEN	HOXD8 (Fragment) (HOXD8)
DH_1392	Pan paniscus	RRRGRQTYSRFQTLELEKEFLFNPYLTRKRRIEVSHALALTERQVKIWFQNRRMKWKKEN	HOXD8 (Fragment) (HOXD8)
DH_9383	Oryzias latipes	--------------------LFNPYLTRKRRIEVSHALALTERQVKIWFQNRRMKWKKEN	HOX8B (Fragment) (hox8b)
DH_6834	Oreochromis niloticus	------------TLELEKEFLFNPYLTRKRRIEVSHALALTERQVKIWFQNRRMKWKKEN	Hox protein (Fragment)
DH_2128	Haplochromis burtoni	RRRGRQTYSRYQTLELEKEFLFNPYLTRKRRIEVSHALALTERQVKIWFQNRRMKWKKEN	Hoxb8a (hoxb8a)
DH_6718	Oreochromis niloticus	RRRGRQTYSRYQTLELEKEFLFNPYLTRKRRIEVSHALALTERQVKIWFQNRRMKWKKEN	Hox protein (Fragment)
DH_522	Fugu rubripes	RRRGRQTYSRYQTLELEKEFLFNPYLTRKRRIEVSHALALTERQVKIWFQNRRMKWKKEN	Homeobox protein Hox-B8a (hoxb8a)
DH_6169	Oryzias latipes	RRRGRQTYSRYQTLELEKEFLFNPYLTRKRRIEVSHALALTERQVKIWFQNRRMKWKKEN	HoxB8a (hoxB8a)
DH_6598	Tetraodon nigroviridis	RRRGRQTYSRYQTLELEKEFLFNPYLTRKRRIEVSHALALTERQVKIWFQNRRMKWKKEN	Chromosome undetermined SCAF10600, whole genome shotgun sequence. (Fragment) (GSTENG00008821001)
DH_516	Gallus gallus	RRRGRQTYSRYQTLELEKEFLFNPYLTRKRRIEVSR------------------------	Homeobox protein Hox-B8 (Fragment) (HOXB8)
DH_9355	Petromyzon marinus	RRRGRQTYSRYQTLELEKEFLFNPYLTRKRRIEVSHVLGLSERQVKIWFQNRRMKWKKEN	Homeobox protein HoxR8 (Fragment) (HoxR8)
DH_1542	Megalobrama amblycephala	RRRGRQTYSRYQTLELEKEFLFNPYLTRKRRIEVSHALGLTERQVKIWFQNRRMKWKKVN	Homeodomain protein (Fragment) (HoxB8a)
DH_1418	Mus musculus	RRRGRQTYSRYQTLELEKEFLFNPYLTRKRRIEVSHALGLTERQVKI-------------	Homeo box B8 (Fragment) (Hoxb8)
DH_3307	Salmo salar	RRRGRQTYSRYQTLELEKEFLFNPYLTRKRRIEVSHALGLTERQVKIWFQNRRMKWKREN	Homeobox protein HoxB8aa (HoxB8aa)
DH_9655	Gallus gallus	RRRGRQTYSRYQTLELEKEFLFNPYLTRKRRIEVSHALGLTERQVKIWFQNRRMKWKKEN	Hoxb-8
DH_517	Homo sapiens	RRRGRQTYSRYQTLELEKEFLFNPYLTRKRRIEVSHALGLTERQVKIWFQNRRMKWKKEN	Homeobox protein Hox-B8 (HOXB8)
DH_8485	Homo sapiens	RRRGRQTYSRYQTLELEKEFLFNPYLTRKRRIEVSHALGLTERQVKIWFQNRRMKWKKEN	cDNA FLJ38903 fis, clone NT2NE2001252, highly similar to HOMEOBOX PROTEIN HOX-B8
DH_518	Mus musculus	RRRGRQTYSRYQTLELEKEFLFNPYLTRKRRIEVSHALGLTERQVKIWFQNRRMKWKKEN	Homeobox protein Hox-B8 (Hoxb8)
DH_1419	Mus musculus	RRRGRQTYSRYQTLELEKEFLFNPYLTRKRRIEVSHALGLTERQVKIWFQNRRMKWKKEN	Homeo box B8 (Hoxb8)
DH_7517	Lampetra japonica	RRRGRQTYSRYQTLELEKEFLFNPHLTRKRRIEVSHALGLTERQVKIWFQNRRMKWKKEN	LjHox8p Homeobox (Fragment) (LjHox8p)
DH_521	Danio rerio	RRRGRQTYSRYQTLELEKEFLFNPYLTRKRRIEVSHALGLTERQVKIWFQNRRMKWKKEN	Homeobox protein Hox-B8a (hoxb8a)
DH_3346	Salmo salar	RRRGRQTYSRYQTLELEKEFLFNPYLTRKRRIEVSHALGLTERQVKIWFQNRRMKWKKEN	Homeobox protein HoxB8aa (HoxB8aa)
DH_3317	Salmo salar	RRRGRQTYSRYQTLELEKEFLFNPYLTRKRRIEVSHALGLTERQVKIWFQNRRMKWKKEN	Homeobox protein HoxB8ab (Homeobox protien HoxB8ab) (HoxB8ab)
DH_8800	Xenopus laevis	RRRGRQTYSRYQTLELEKEFLFNPYLTRKRRIEVSHALGLTERQVKIWFQNRRMKWKKEN	Homeodomain protein (Fragment) (homeobox)
DH_8047	Rattus sp	RRRGRQTYSRYQTLELEKEFLFNPYLTRKRRIEVSHALGLTERQVKIWFQNRRMKWKKEN	Hox-B|Hox-2 (Fragment) (Hox-B|Hox-2)
DH_2202	Homo sapiens	RRSGRQTYSRYQTLELEKEFLFNPYLTRKRRIEVSHALGLTERQVKIWFQNRRMKWKKEN	cDNA FLJ77104, highly similar to Homo sapiens homeo box C8 (HOXC8), mRNA (Homeobox C8) (HOXC8)
DH_4459	Xenopus laevis	RRSGRQTYSRYQTLELEKEFLFNPYLTRKRRIEVSHALGLTERQLKIWFQNRRMKWKKEN	Homeobox protein pXhoxc8 (xhoxc8)
DH_567	Mus musculus	RRSGRQTYSRYQTLELEKEFLFNPYLTRKRRIEVSHALGLTERQVKIWFQNRRMKWKKEN	Homeobox protein Hox-C8 (Hoxc8)
DH_6812	Trionyx sinensis	RRSGRQTYSRYQTLELEKEFLFNPYLTRKRRIEVSHALGLTERQVKIWFQNRRMKWKKEN	Hoxc-8 (Fragment) (Hoxc-8)
DH_8008	Latimeria menadoensis	RRSGRQTYSRYQTLELEKEFLFNPYLTRKRRIEVSHALGLTERQVKIWFQNRRMKWKKEN	HoxC8 (Fragment)

DH_7425	Xenopus tropicalis	RRSGRQTYSRYQTLELEKEFLFNPYLTRKRRIEVSHALGLTERQVKIWFQNRRMKWKKEN	Homeobox C8 (hoxc8)
DH_4800	Xenopus laevis	RRSGRQTYSRYQTLELEKEFLFNPYLTRKRRIEVSHALGLTERQVKIWFQNRRMKWKKEN	LOC100158353 protein (hoxc8)
DH_565	Gallus gallus	RRSGRQTYSRYQTLELEKEFLFNPYLTRKRRIEVSHALGLTERQVKIWFQNRRMKWKKEN	Homeobox protein Hox-C8 (HOXC8)
DH_7819	Felis silvestris catus	RRSGRQTYSRYQTLELEKEFLFNPYLTRKRRIEVSHALGLTERQVKIWFQNRRMKWKKEN	Homeotic protein HOX3A (Fragment)
DH_568	Rattus norvegicus	RRSGRQTYSRYQTLELEKEFLFNPYLTRKRRIEVSHALGLTERQVKIWFQNRRMKWKKEN	Homeobox protein Hox-C8 (Fragment) (Hoxc8)
DH_8310	Mus musculus	RRSGRQTYSRYQTLELEKEFLFNPYLTRKRRIEVSHALGLTERQVKIWFQNRRMKWKKEN	Putative uncharacterized protein (Fragment) (Hoxc8)
DH_8307	Mus musculus	RRSGRQTYSRYQTLELEKEFLFNPYLTRKRRIEVSHALGLTERQVKIWFQNRRMKWKKEN	Putative uncharacterized protein (Fragment) (Hoxc8)
DH_8308	Mus musculus	RRSGRQTYSRYQTLELEKEFLFNPYLTRKRRIEVSHALGLTERQVKIWFQNRRMKWKKEN	Putative uncharacterized protein (Fragment) (Hoxc8)
DH_566	Homo sapiens	RRSGRQTYSRYQTLELEKEFLFNPYLTRKRRIEVSHALGLTERQVKIWFQNRRMKWKKEN	Homeobox protein Hox-C8 (HOXC8)
DH_8046	Rattus sp	RRSGRQTYSRYQTLELEKEFLFNPYLTRKRRIEVSHALGLTERQVKIWFQNRRMKWKKEN	Hox-C|Hox-3 (Fragment) (Hox-C|Hox-3)
DH_3264	Salmo salar	RRSGRQTYSRYQTLELEKEFLFNPYLTRKRRIEVSHALGLTERQVKIWFQNRRMKWKKEN	Homeobox protein HoxC8bb (HoxC8bb)
DH_3257	Salmo salar	RRSGRQTYSRYQTLELEKEFLFNPYLTRKRRIEVSHALGLTERQVKIWFQNRRMKWKKEN	Homeobox protein HoxC8ba (HoxC8ba)
DH_11837	Gasterosteus aculeatus	RRNGRQTYSRYQTLELEKEFLFNPYLTRKRRIEVSHALSLTERQVKIWFQNRRMKWKKEN	Homeobox protein Hox-C8 (Hox-3A) [Source:UniProtKB/Swiss-Prot;Acc:P31273]
DH_570	Fugu rubripes	RRNGRQTYSRYQTLELEKEFLFNPYLTRKRRIEVSHALSLTERQVKIWFQNRRMKWKKEN	Homeobox protein Hox-C8a (hoxc8a)
DH_2141	Haplochromis burtoni	RRNGRQTYSRYQTLELEKEFLFNPYLTRKRRIEVSHALSLTERQVKIWFQNRRMKWKKEN	Hoxc8a (hoxc8a)
DH_6158	Oryzias latipes	RRNGRQTYSRYQTLELEKEFLFNPYLTRKRRIEVSHALSLTERQVKIWFQNRRMKWKKEN	HoxC8a (hoxC8a)
DH_3238	Salmo salar	RRNGRQTYSRYQTLELEKEFLFNPYLTRKRRIEVSHALSLTERQVKIWFQNRRMKWKKEN	Homeobox protein HoxC8aa (HoxC8aa)
DH_3327	Salmo salar	RRNGRQTYSRYQTLELEKEFLFNPYLTRKRRIEVSHALSLTERQVKIWFQNRRMKWKKEN	Homeobox protein HoxC8aa2 (HoxC8aa)
DH_3247	Salmo salar	RRNGRQTYSRYQTLELEKEFLFNPYLTRKRRIEVSHALSLTERQVKIWFQNRRMKWKKEN	Homeobox protein HoxC8an (Homeobox protein HoxC8ab) (HoxC8ab)
DH_569	Danio rerio	RRNGRQTYSRYQTLELEKEFLFNPYLTRKRRIEVSHALSLTERQVKIWFQNRRMKWKKEN	Homeobox protein Hox-C8a (hoxc8a)
DH_6451	Tetraodon nigroviridis	RRNGRQTYSRYQTLELEKEFLFNPYLTRKRRIEVSHALSLTERQVKIWFQNRRMKWKKEN	Chromosome 9 SCAF14991, whole genome shotgun sequence (GSTENG00028039001)
DH_9384	Oryzias latipes	--------------------LFNPYLTRKRRIEVSHALSLTERQVKIWFQNRRMKWKKEN	HOXC8A (Fragment) (hoxc8a)
DH_520	Rattus norvegicus	TQRGRQTYSRYQTLELEKEFLFNPYLTRKRRIEVSHALGLTERQVKIWFQNRRMKWKKEN	Homeobox protein Hox-B8 (Fragment) (Hoxb8)
DH_8629	Euprymna scolopes	RRRGRQTYSRFQTLELEKEFQYNNYLTRKRRIEVAHALNLSERQVKIWFQNRRMKLKKEK	Lox4 homeodomain protein (Fragment) (Lox4)
DH_519	Sus scrofa	HRRGLQTCSRYQTLELEKEFQCNPYLTCKRWIEVSHALGLTERQIKIWFQNRRMKWKKRE	Homeobox protein Hox-B8 (Fragment) (HOXB8)

Save alignment [fasta format] - Save complete sequences [fasta format]

PG9

ID	organism	10 20 30 40 50 60	description
DH_12582	Strongylocentrotus purpuratus	RRTKRRPYSKLQIYELEKEFTTNMYLTRDRRSKLSQALDLTERQVKIWFQNRRMKMKKLN	GLEAN3_02631|Sp-Hox11/13b
DH_4488	Strongylocentrotus purpuratus	RRTKRRPYSKLQIYELEKEFTTNMYLTRDRRSKLSQALDLTERQVKIWFQNRRMKMKKLN	SpHbox7 (Hbox7)
DH_6314	Heliocidaris tuberculata	RRTKRRPYSKLQIYELEKEFQANMYLTRDRRSKLSQALDLTERQVKIWFQNRRMKMKKLN	Homeobox protein Hox11/13 (Hox11/13)
DH_6315	Heliocidaris erythrogramma	RRTKRRPYSKLQIYELEKEFQANMYLTRDRRSKLSQALDLTERQVKIWFQNRRMKMKKLN	Homeobox protein Hox11/13 (Hox11/13)
DH_8869	Heliocidaris erythrogramma	RRTKRRPYSKLQIYELEKEFQANMYLTRDRRSKLSQALDLTERQVKIWFQNRRMKMKKLN	HEHBOX7 (Fragment) (HeHbox7)
DH_7665	Ptychodera flava	RRTKRRPYSKLQIFELEKEFQQNMYLTRDRRTRLAQTLNLTERQVKIWFQNRRMKLKKMT	Transcription factor Hox11/13c
DH_7934	Saccoglossus kowalevskii	RRTKRRPYSKLQIFELEKEFQQNMYLTRDRRSRLSQALNLTERQIKIWFQNRRMKLKKMT	Hox 11/13c
DH_4584	Holothuria glaberrima	RRTKRRPYSKLQIYELEKEFQHNMYLTRDRRAKLSQTLSLTERQVKIWFQNRRMKLKKIT	Homeodomain-containing DNA binding protein 12 (Fragment) (Hbox12)
DH_12653	Strongylocentrotus purpuratus	RRTKRRPYTKLQIFELEKEFQAHQYLTRDRRARLSQSLSLSERQVKIWFQNRRMKQKKMN	GLEAN3_00388|Sp-Hox11/13c
DH_1330	Metacrinus rotundus	RRTKRRPYSKVQIFELEKEFQLHQYLTRDRRARLSQSLTLSERQVKIWFQNRRMKQKKLN	Transcription factor Hox11/13c (MrHox11/13c)
DH_1266	Saccoglossus kowalevskii	RRTKRKPYTKMQIFELEQAFQNNMYLTRERRTKLSQQLSLSERQIKIWFQNRRMKLKKMT	Hox 11/13b
DH_7666	Ptychodera flava	RRTKRRPYSKMQIYELEKAFQQNAYLTRERRQKYSQQLNLTERQVKIWFQNRRMKSKKQV	Transcription factor Hox11/13b (Fragment)
DH_4551	Lineus sanguineus	TRKKRKPYTRYQTMVLENEFLTNSYITRQKRWEISCKLHLTERQVKVWFQNRRMKRKKLN	Homeodomain protein (Fragment) (Hox-9)
DH_8627	Euprymna scolopes	GRKKRKPYTRYQTMVLENEFLNSSYITRQKRWEISCKLQLTERQVKVWFQNRRMKRKKLN	Posterior-2 homeodomain protein (Fragment) (Post2)
DH_7443	Bugula turrita	SRKKRKPYTRYQTMVLETEFINNSYITRQKRWEISCRLRLTERQVKVWFQNRRMKRKKLN	Hox class homeodomain-containing protein Post2 (Fragment)
DH_2763	Flaccisagitta enflata	-RKKRKPYTKHQTFILEQEYLMSTYITRQRRLELARNLSLTERQVKIWFQNRRMKTKKLR	Posterior Hox A (Fragment)
DH_11463	Daphnia pulex	VRKKRKPYSKFQTLELEKEFLFNAYVSKQKRWELARNLNLTERQVKIWFQNRRMKNKKNS	gw1.7.450.1|Dappu1
DH_11514	Daphnia pulex	VRKKRKPYSKFQTLELEKEFLFNAYVSKQKRWELARNLNLTERQVKIWFQNRRMKNKKNS	YAS_Hox_Abd-B|Dappu1
DH_5410	Strigamia maritima	VRKKRKPYSKFQTLELEKEFLFNAYVSKQKRWELARNLNLTERQVKIWFQNRRMKNKKNS	Abdominal-B (abdB)
DH_8989	Folsomia candida	VRKKRKPYSKFQTLELEKEFLFNAYVSKQKRWELARNLNLTERQVKIWFQNRRMKNKKNS	Abdominal-B (Fragment) (AbdB)
DH_5374	Anopheles gambiae	VRKKRKPYSKFQTLELEKEFLFNAYVSKQKRWELARNLNLTERQVKIWFQNRRMKNKKNS	Abdominal-B
DH_7876	Anopheles gambiae	VRKKRKPYSKFQTLELEKEFLFNAYVSKQKRWELARNLNLTERQVKIWFQNRRMKNKKNS	AGAP004664-PA (AGAP004664)
DH_5273	Aedes aegypti	VRKKRKPYSKFQTLELEKEFLFNAYVSKQKRWELARNLNLTERQVKIWFQNRRMKNKKNS	Homeobox protein abdominal-B, putative (AAEL009933)
DH_5274	Aedes aegypti	VRKKRKPYSKFQTLELEKEFLFNAYVSKQKRWELARNLNLTERQVKIWFQNRRMKNKKNS	Homeobox protein abdominal-B, putative (AAEL009743)
DH_9287	Tribolium castaneum	VRKKRKPYSKFQTLELEKEFLFNAYVSKQKRWELARNLNLTERQVKIWFQNRRMKNKKNS	Abdominal-B
DH_1771	Drosophila melanogaster	VRKKRKPYSKFQTLELEKEFLFNAYVSKQKRWELARNLQLTERQVKIWFQNRRMKNKKNS	CG11648-PC, isoform C (CG11648-PD, isoform D) (CG11648-PE, isoform E) (Abd-B)
DH_4222	Drosophila simulans	VRKKRKPYSKFQTLELEKEFLFNAYVSKQKRWELARNLQLTERQVKIWFQNRRMKNKKNS	Abd-B (Abd-B)
DH_9	Drosophila melanogaster	VRKKRKPYSKFQTLELEKEFLFNAYVSKQKRWELARNLQLTERQVKIWFQNRRMKNKKNS	Homeobox protein abdominal-B (Abd-B)
DH_3593	Drosophila sechellia	VRKKRKPYSKFQTLELEKEFLFNAYVSKQKRWELARNLQLTERQVKIWFQNRRMKNKKNS	Abd-B (Abd-B)
DH_4123	Drosophila yakuba	VRKKRKPYSKFQTLELEKEFLFNAYVSKQKRWELARNLQLTERQVKIWFQNRRMKNKKNS	GE26123 (GE26123)
DH_3153	Drosophila erecta	VRKKRKPYSKFQTLELEKEFLFNAYVSKQKRWELARNLQLTERQVKIWFQNRRMKNKKNS	GG16806 (GG16806)
DH_3006	Drosophila ananassae	VRKKRKPYSKFQTLELEKEFLFNAYVSKQKRWELARNLQLTERQVKIWFQNRRMKNKKNS	GF17921 (GF17921)
DH_3431	Drosophila persimilis	VRKKRKPYSKFQTLELEKEFLFNAYVSKQKRWELARNLQLTERQVKIWFQNRRMKNKKNS	GL23001 (GL23001)
DH_5761	Drosophila pseudoobscura pseudoobscura	VRKKRKPYSKFQTLELEKEFLFNAYVSKQKRWELARNLQLTERQVKIWFQNRRMKNKKNS	GA11117 (GA11117)
DH_4047	Drosophila willistoni	VRKKRKPYSKFQTLELEKEFLFNAYVSKQKRWELARNLQLTERQVKIWFQNRRMKNKKNS	GK13851 (GK13851)
DH_3689	Drosophila grimshawi	VRKKRKPYSKFQTLELEKEFLFNAYVSKQKRWELARNLQLTERQVKIWFQNRRMKNKKNS	GH18073 (GH18073)
DH_3769	Drosophila mojavensis	VRKKRKPYSKFQTLELEKEFLFNAYVSKQKRWELARNLQLTERQVKIWFQNRRMKNKKNS	GI23752 (GI23752)
DH_3921	Drosophila virilis	VRKKRKPYSKFQTLELEKEFLFNAYVSKQKRWELARNLQLTERQVKIWFQNRRMKNKKNS	GJ10598 (GJ10598)
DH_2592	Culex quinquefasciatus	VRKKRKPYSKFQTLELEKEFLYNAYVSKQKRWELARNLNLTERQVKIWFQNRRMKNKKNS	Homeobox protein abdominal-B (CpipJ_CPIJ001021)
DH_7233	Porcellio scaber	-RKKRKPYSKFQTLELEKEFLYNAYVSKQKRWELARNLNLTERQVKIWFQNRRMKKKKNS	Abdominal-B (Fragment)
DH_5232	Artemia sanfranciscana	VRKKRKPYSKFQTLELEKEFLFNAYVSKQKRWELARNLNLTERQVKI-------------	AbdB protein (Fragment) (abdB)
DH_9612	Cupiennius salei	VRKKRKPYSKFQTLELEKEFLFNAYVSKQKRWELARNLNLTERQVKIWFQNRRMKSKKTS	Abdominal-B (abd-B)
DH_4294	Archegozetes longisetosus	VRKKRKPYSKFQTLELEKEFLFNAYVSKQKRWELARNLNLTERQVKIWFQNRRMKSKKTS	Abdominal-B (Fragment)
DH_9509	Thermobia domestica	-RKKRKPYSKFQTLELEKEFLFNAYVSKQKRWELARNLNLTERQVKIWFQNRR-------	Abdominal-B protein (Fragment) (abd-B)
DH_2474	Endeis spinosa	IRKKRKPYSKFQTLELEKEFLFNAYVSKQKRWESARNLNLSERQVKIWFQNRRMKNKKNS	Homeobox protein abdominalB (Fragment) (AbdB)
DH_4680	Sacculina carcini	VRKKRKPYSKYQTLELEKEFLFNAYVSKLKRWELARNLNLTERQVKI-------------	AbdominalB (Fragment) (AbdB)
DH_8117	Sacculina carcini	VRKKRKPYSKYQTLELEKEFLFNAYVSKQKRWELARNLNLTERQVKIWFQNRRMKNKKAQ	Abdominal B (AbdB)
DH_9626	Priapulus caudatus	VRKKRKPYTKYQTLELEKEFLFNAYVSKQKRWELARTLNLTERQVKIWFQNRRMKSKKSN	Abdominal-B homeodomain protein (Fragment) (Abd-B)
DH_9474	Caenorhabditis elegans	MRKKRKPYTKAQTLELEKEFLYNTYVSKQKRWELAKYLHLTERQVKIWFQNRRMKDKKQK	Homeodomain protein PHP-3 (Fragment) (php-3)
DH_9588	Caenorhabditis elegans	MRKKRKPYTKAQTLELEKEFLYNTYVSKQKRWELAKYLHLTERQVKIWFQNRRMKDKKQK	Protein Y75B8A.1, confirmed by transcript evidence (php-3)
DH_2417	Caenorhabditis briggsae	MRKKRKPYTKAQTLELEKEFLYNTYVSKQKRWELAKYLHLTERQVKIWFQNRRMKDKKQK	CBR-PHP-3 protein (Cbr-php-3)
DH_2243	Brugia malayi	SRKKRKPYTKYQTLELEKEFLYNTYVSKQKRYELAKNLYLSERQVKIWFQNRRMKDKKQK	Homeobox domain containing protein (Bm1_19850)
DH_5731	Xenopus tropicalis	TRKKRCPYTKYQTLELEKEFLFNMYLTRDRRYEVARVLNLTERQVKIWFQNRRMKMKKMN	Homeo box C9 (hoxc9)
DH_8045	Rattus sp	TRKKRCPYTKYQTLELEKEFLFNMYLTRDRRYEVARVLNLTERQVKIWFQNRRMKMKKMN	Hox-C|Hox-3 (Fragment) (Hox-C|Hox-3)
DH_8007	Latimeria menadoensis	TRKKRCPYTKYQTLELEKEFLFNMYLTRDRRYEVARVLNLTERQVKIWFQNRRMKMKKMN	HoxC9 (Fragment)
DH_4806	Xenopus tropicalis	TRKKRCPYTKYQTLELEKEFLFNMYLTRDRRYEVARVLNLTERQVKIWFQNRRMKMKKMN	Putative uncharacterized protein (Fragment)
DH_574	Ovis aries	TRKKRCPYTKYQTLELEKEFLFNMYLTRDRRYEVARVLNLTERQVKIWFQNRRMKMKKMN	Homeobox protein Hox-C9 (Fragment) (HOXC9)
DH_4807	Capra hircus	TRKKRCPYTKYQTLELEKEFLFNMYLTRDRRYEVARVLNLTERQVKIWFQNRRMKMKKMN	Homeobox C9 (Hoxc9)
DH_572	Mus musculus	TRKKRCPYTKYQTLELEKEFLFNMYLTRDRRYEVARVLNLTERQVKIWFQNRRMKMKKMN	Homeobox protein Hox-C9 (Hoxc9)

DH_8484	Homo sapiens	TRKKRCPYTKYQTLELEKEFLFNMYLTRDRRYEVARVLNLTERQVKIWFQNRRMKMKKMN	HOXC9 protein (Fragment) (HOXC9)
DH_2845	Homo sapiens	TRKKRCPYTKYQTLELEKEFLFNMYLTRDRRYEVARVLNLTERQVKIWFQNRRMKMKKMN	cDNA, FLJ96178, Homo sapiens homeo box C9 (HOXC9), mRNA (Homeobox C9, isoform CRA_a) (HOXC9)
DH_571	Homo sapiens	TRKKRCPYTKYQTLELEKEFLFNMYLTRDRRYEVARVLNLTERQVKIWFQNRRMKMKKMN	Homeobox protein Hox-C9 (HOXC9)
DH_6709	Oreochromis niloticus	TRKKRCPYTKYQTLELEKEFLFNMYLTRDRRYEVARVLNLTERQVKIWFQNRRMKMKKMN	Hox protein (Fragment)
DH_6780	Oncorhynchus mykiss	TRKKRCPYTKYQTLELEKEFLFNMYLTRDRRYEVARVLNLTERQVKIWFQNRRMKMKKMN	HoxC9bi (Fragment) (Hox)
DH_3263	Salmo salar	TRKKRCPYTKYQTLELEKEFLFNMYLTRDRRYEVARVLNLTERQVKIWFQNRRMKMKKMN	Homeobox protein HoxC9bb (HoxC9bb)
DH_1463	Danio rerio	TRKKRCPYTKYQTLELEKEFLFNMYLTRDRRYEVARVLNLTERQVKIWFQNRRMKMKKMN	Homeo box C9a (hoxc9a)
DH_575	Danio rerio	TRKKRCPYTKYQTLELEKEFLFNMYLTRDRRYEVARVLNLTERQVKIWFQNRRMKMKKMN	Homeobox protein Hox-C9a (hoxc9a)
DH_2140	Haplochromis burtoni	TRKKRCPYTKYQTLELEKEFLFNMYLTRDRRYEVARVLNLTERQVKIWFQNRRMKMKKMN	Hoxc9a (hoxc9a)
DH_6157	Oryzias latipes	TRKKRCPYTKYQTLELEKEFLFNMYLTRDRRYEVARVLNLTERQVKIWFQNRRMKMKKMN	HoxC9a (hoxC9a)
DH_573	Oryzias latipes	TRKKRCPYTKYQTLELEKEFLFNMYLTRDRRYEVARVLNLTERQVKIWFQNRRMKMKKMN	Homeobox protein Hox-C9 (hoxc9)
DH_3256	Salmo salar	TRKKRCPYTKYQTLELEKEFLFNMYLTRDRRYEVALVLNLTERQVKIWFQNRRMKMKKMN	Homeobox protein HoxC9ba (HoxC9ba)
DH_3362	Salmo salar	TRKKRCPYTKYQTLELEKEFLFNMYLTRDRRYEVALVLNLTERQVKIWFQNRRMKMKKMN	Homeobox protein HoxC9ba (HoxC9ba)
DH_6779	Oncorhynchus mykiss	TRKKRCPYSKYQTLELEKEFLFNMYLTRDRRYEVARVLNLTERQVKIWFQNRRMKMKKMN	HoxC9a-1-R (Fragment) (Hox)
DH_3359	Salmo salar	TRKKRCPYSKYQTLELEKEFLFNMYLTRDRRYEVARVLNLTERQVKIWFQNRRMKMKKMN	Homeobox protein HoxC9aa (HoxC9aa)
DH_3246	Salmo salar	TRKKRCPYSKYQTLELEKEFLFNMYLTRDRRYEVARVLNLTERQVKIWFQNRRMKMKKMN	Homeobox protein HoxC9ab (HoxC9ab)
DH_3237	Salmo salar	TRKKRCPYSKYQTLELEKEFLFNMYLTRDRRYEVARVLNLTERQVKIWYQNRRMKMKKMN	Homeobox protein HoxC9aa (HoxC9aa)
DH_11839	Gasterosteus aculeatus	TRKKRCPYTKYQTLELEKEFLFNMYLTRDRRFEVARVLNLTERQVKIWFQNRRMKMKKMN	Homeobox protein Hox-C9 (Hox-3B) [Source:UniProtKB/Swiss-Prot;Acc:P31274]
DH_576	Fugu rubripes	TRKKRCPYTKYQTLELEKEFLFNMYLTRDRRFEVARVLNLTERQVKIWFQNRRMKMKKMN	Homeobox protein Hox-C9a (hoxc9a)
DH_8747	Xenopus laevis	TRKKRCPYTKYQTLELEKEFLFNLYLTRDRRYEVARILNLTER-----------------	Homeobox protein, Hoxd9 (Fragment) (hoxd9)
DH_3273	Salmo salar	TRKKRCPYTKYQTLELEKEFLFNTYLTRDRRYEVARILNLTERQVKIWFQNRRMKMKKMN	Homeobox protein HoxD9aa (HoxD9aa)
DH_638	Homo sapiens	TRKKRCPYTKYQTLELEKEFLFNMYLTRDRRYEVARILNLTERQVKIWFQNRRMKMKKMS	Homeobox protein Hox-D9 (HOXD9)
DH_2223	Homo sapiens	TRKKRCPYTKYQTLELEKEFLFNMYLTRDRRYEVARILNLTERQVKIWFQNRRMKMKKMS	Putative uncharacterized protein HOXD9 (HOXD9)
DH_639	Mus musculus	TRKKRCPYTKYQTLELEKEFLFNMYLTRDRRYEVARILNLTERQVKIWFQNRRMKMKKMS	Homeobox protein Hox-D9 (Hoxd9)
DH_1442	Mus musculus	TRKKRCPYTKYQTLELEKEFLFNMYLTRDRRYEVARILNLTERQVKIWFQNRRMKMKKMS	Homeo box D9 (Hoxd9)
DH_6115	Mus musculus	TRKKRCPYTKYQTLELEKEFLFNMYLTRDRRYEVARILNLTERQVKIWFQNRRMKMKKMS	Putative uncharacterized protein (Fragment) (Hoxd9)
DH_4310	Rattus norvegicus	TRKKRCPYTKYQTLELEKEFLFNMYLTRDRRYEVARILNLTERQVKIWFQNRRMKMKKMS	Putative uncharacterized protein
DH_636	Gallus gallus	TRKKRCPYTKYQTLELEKEFLFNMYLTRDRRYEVARILNLTERQVKIWFQNRRMKMKKMS	Homeobox protein Hox-D9 (Fragment) (HOXD9)
DH_6775	Oncorhynchus mykiss	TRKKRCPYTKYQTLELEKEFLFNMYLTRDRRYEVARILNLTERQVKIWFQN---------	HoxD9ai (Fragment) (Hox)
DH_6776	Oncorhynchus mykiss	TRKKRCPYTKYQTLELEKEFLFNMYLTRDRRYEVARILNLTERQVKIWFQN---------	HoxD9a-1 (Fragment) (Hox)
DH_1269	Xenopus laevis	TRKKRCPYTKYQTLELEKEFLFNMYLTRDRRYEVARILNLTERQVKIWFQNRRMKMKKMN	Putative uncharacterized protein
DH_637	Heterodontus francisci	TRKKRCPYTKYQTLELEKEFLFNMYLTRDRRYEVARILNLTERQVKIWFQNRRMKMKKMN	Homeobox protein Hox-D9 (HOXD9)
DH_3366	Salmo salar	TRKKRCPYTKYQTLELEKEFLFNMYLTRDRRYEVARILNLTERQVKIWFQNRRMKMKKMN	Homeobox protein HoxD9aa (HoxD9aa)
DH_8000	Latimeria menadoensis	TRKKRCPYTKYQTLELEKEFLFNMYLTRDRRYEVARILDLTERQVKIWFQNRRMKMKKMN	HoxD9 (Fragment)
DH_11725	Gasterosteus aculeatus	TRKKRCPYTKYQTLELEKEFLYNMYLTRDRRYEVARILSLTERQVKIWFQNRRMKMKKMN	HoxD9a. [Source:UniProtKB/TrEMBL;Acc:Q4VQD4]
DH_6670	Gasterosteus aculeatus	TRKKRCPYTKYQTLELEKEFLYNMYLTRDRRYEVARILSLTERQVKIWFQNRRMKMKKMN	HoxD9a
DH_11715	Gasterosteus aculeatus	TRKKRCPYTKYQTLELEKEFLYNMYLTRDRRYEVARILSLTERQVKIWFQNRRMKMKKMN	HoxD9a. [Source:UniProtKB/TrEMBL;Acc:Q4VQD4]
DH_2165	Haplochromis burtoni	TRKKRCPYTKYQTLELEKEFLYNMYLTRDRRYEVARILSLTERQVKIWFQNRRMKMKKMN	Hoxd9a (hoxd9a)
DH_6704	Oreochromis niloticus	TRKKRCPYTKYQTLELEKEFLYNMYLTRDRRYEVARILSLTERQVKIWFQNRRMKMKKMN	Hox protein (Fragment)
DH_640	Oryzias latipes	TRKKRCPYTKYQTLELEKEFLYNMYLTRDRRFEVARILSLTERQVKIWFQNRRMKMKKMN	Homeobox protein Hox-D9 (hoxd9)
DH_6150	Oryzias latipes	TRKKRCPYTKYQTLELEKEFLYNMYLTRDRRFEVARILSLTERQVKIWFQNRRMKMKKMN	HoxD9a (hoxD9a)
DH_642	Fugu rubripes	TRKKRCPYTKYQTLELEKEFLYNMYLTRDRRYEVARILSLTERQVKIWFQNRRMKMKKQN	Homeobox protein Hox-D9a (hoxd9a)
DH_6416	Tetraodon nigroviridis	TRKKRCPYTKYQTLELEKEFLYNMYLTRDRRYEVARILSLTERQVKIWFQNRRMKMKKLN	Chromosome 2 SCAF15010, whole genome shotgun sequence (GSTENG00031462001)
DH_1558	Megalobrama amblycephala	TRKKRCPYTKYQTLELEKEFLYNMYLTRDRRYEVARILNLTERQVKIWFQNR--------	Homeodomain protein (Fragment) (HoxD9a)
DH_641	Danio rerio	TRKKRCPYTKYQTLELEKEFLYNMYLTRDRRYEVARILNLTERQVKIWFQNRRMKMKKMN	Homeobox protein Hox-D9a (hoxd9a)
DH_2556	Danio rerio	TRKKRCPYTKYQTLELEKEFLYNMYLTRDRRYEVARILNLTERQVKIWFQNRRMKMKKMN	Homeo box D9a (hoxd9a)
DH_3368	Salmo salar	TRKKRCPYTKYQTLELEKEFLYNMYLTRDRRNEMARNLNLTERQVKIWFQNRRMKMKKMN	Homeobox protein HoxD9ab (HoxD9ab)
DH_3280	Salmo salar	TRKKRCPYTKYQTLELEKEFLYNMYLTRDRRNEMARNLNLTERQVKIWFQNRRMKMKKMN	Homeobox protein HoxD9ab (HoxD9ab)
DH_6774	Oncorhynchus mykiss	TRKKRCPYTKYQTLELEKEFLYNVYLTRDRRYEMARNLNLTERQVKIWFQN---------	HoxD9aii (Fragment) (Hox)
DH_438	Morone saxatilis	TRKKRCPYTKHQILELEKEFLFNTYLTRDRRYEVARLLNLTERQVKIWFQNRRMKMKKFN	Homeobox protein Hox-A9 (hoxa9)
DH_2150	Haplochromis burtoni	TRKKRCPYTKHQILELEKEFLFNTYLTRDRRYEVARLLNLTERQVKIWFQNRRMKMKKFN	Hoxa9a (hoxa9a)
DH_6184	Oryzias latipes	TRKKRCPYTKHQILELEKEFLFNTYLTRDRRYEVARLLNLTERQVKIWFQNRRMKMKKFN	HoxA9a (hoxA9a)
DH_9379	Oryzias latipes	TRKKRCPYTKHQILELEKEFLFNTYLTRDRRYEVARLLNLTERQVKIWFQNRRMKMKKFN	HOXA9A (hxoa9a)
DH_441	Fugu rubripes	TRKKRCPYTKHQILELEKEFLFNTYLTRDRRYEVARLLNLTERQVKIWFQNRRMKMKKFN	Homeobox protein Hox-A9a (hoxa9a)
DH_6542	Tetraodon nigroviridis	TRKKRCPYTKHQILELEKEFLFNTYLTRDRRYEVARLLNLTERQVKIWFQNRRMKMKKFN	Chromosome 21 SCAF14577, whole genome shotgun sequence (GSTENG00017478001)
DH_11799	Gasterosteus aculeatus	TRKKRCPYTKHQILELEKEFLFNTYLTRDRRYEVARLLNLTERQVKIWFQNRRMKMKKFN	Homeobox protein Hox-A9 (Hox-1G) [Source:UniProtKB/Swiss-Prot;Acc:P31269]
DH_440	Danio rerio	TRKKRCPYTKHQILELEKEFLFNTYLTRDRRYEVARLLNLTERQVKIWFQNRRMKMKKFN	Homeobox protein Hox-A9a (hoxa9a)
DH_1528	Megalobrama amblycephala	TRKKRCPYTKHQILELEKEFLFNTYLTRDRRYEVPRLLNLTERQVKIWFQNRRMKL----	Homeodomain protein (Fragment) (HoxA9a)
DH_3285	Salmo salar	TRKKRCPYTKHQILELEKEFLFNTYLTRDRRYEVARQLNLTE---------------------	Homeobox protein HoxA9aa (Fragment) (HoxA9aa)
DH_3333	Salmo salar	TRKKRCPYTKHQILELEKEFLFNTYLTRDRRYEVARQLNLTERQVKIWFQNRRMKMKKCN	Homeobox protein HoxA9aa (HoxA9aa)
DH_3295	Salmo salar	TRKKRCPYSKHQILELEKEFLFNTYLTRDRRFEVARQLNLTERQVKIWFQNRRMKMKKFN	Homeobox protein HoxA9ab (HoxA9ab)
DH_11836	Gasterosteus aculeatus	SRKKRCPYTKHQILELEKEFLFNMYLPRDRRYEVARVLSLTERQVKIWFQNRRMKMKKEN	Homeobox protein Hox-A9 (Hox-1G) [Source:UniProtKB/Swiss-Prot;Acc:P31269]
DH_3304	Salmo salar	SRKKRCPYSKHQILELEKEFLFNMYLPRDRRYEVARVLHLTERQIKIWFQNRRMKMKKGN	Homeobox protein HoxA9b (HoxA9b)
DH_2134	Haplochromis burtoni	TRKKRCPYTKHQILELEKEFLFNMYLPRDRRYEIARLLNLTERQVKIWFQNRRMKMKKEN	Hoxa9b (hoxa9b)
DH_6724	Oreochromis niloticus	TRKKRCPYTKHQILELEKEFLFNMYLPRDRRYEIARLLNLTERQVKIWFQNRRMKMKKEN	Hox protein (Fragment)
DH_6179	Oryzias latipes	TRKKRCPYTKHQILELEKEFLFNMYLPRDRRYEVARLLNLTERQVKIWFQNRRMKMKKEN	HoxA9b (hoxA9b)
DH_9380	Oryzias latipes	TRKKRCPYTKHQILELEKEFLFNMYLPRDRRYEVARLLNLTERQVKIWFQNRRMKMKKEN	HOXA9B (hoxa9b)
DH_443	Fugu rubripes	TRKKRCPYTKHQILELEKEFLFNMYLPRDRRYEVARLLHLTERQVKIWFQNRRMKMKKEN	Homeobox protein Hox-A9b (hoxa9b)
DH_442	Danio rerio	TRKKRCPYTKHQTLELEKEFLFNMYLSRDRRYEVARLLNLTERQVKIWFQNRRMKMKKCN	Homeobox protein Hox-A9b (hoxa9b)
DH_1653	Carassius auratus	--KKRVPYTKHQTLELEKEFLFNMYLSRDRRYEVARLLNLTERQVEIWFQNRRM------	HoxA9b protein (Fragment) (Hox)
DH_437	Homo sapiens	TRKKRCPYTKHQTLELEKEFLFNMYLTRDRRYEVARLLNLTERQVKIWFQNRRMKMKKIN	Homeobox protein Hox-A9 (HOXA9)
DH_2448	Papio anubis	TRKKRCPYTKHQTLELEKEFLFNMYLTRDRRYEVARLLNLTERQVKIWFQNRRMKMKKIN	Homeobox A9 (Predicted) (HOXA9)

```
DH_439   Mus musculus                    TRKKRCPYTKHQTLELEKEFLFNMYLTRDRRYEVARLLNLTERQVKIWFQNRRMKMKKIN Homeobox protein Hox-A9 (Hoxa9)
DH_2747  Callicebus moloch               TRKKRCPYTKHQTLELEKEFLFNMYLTRDRRYEVARLLNLTERQVKIWFQNRRMKMKKIN Homeobox protein Hox-A9 (Predicted) (HOXA9)
DH_2812  Rhinolophus ferrumequinum       TRKKRCPYTKHQTLELEKEFLFNMYLTRDRRYEVARLLNLTERQVKIWFQNRRMKMKKIN Homeobox A9 (Predicted) (HOXA9)
DH_3193  Sorex araneus                   TRKKRCPYTKHQTLELEKEFLFNMYLTRDRRYEVARLLNLTERQVKIWFQNRRMKMKKIN Homeobox A9 (Predicted) (HOXA9)
DH_4574  Homo sapiens                    TRKKRCPYTKHQTLELEKEFLFNMYLTRDRRYEVARLLNLTERQVKIWFQNRRMKMKKIN HOXA-9B (HOXA-9)
DH_435   Gallus gallus                   TRKKRCPYTKHQTLELEKEFLFNMYLTRDRRYEVARLLNLTERQVKIWFQNRRMKMKKIN Homeobox protein Hox-A9 (Fragment) (HOXA9)
DH_433   Ambystoma mexicanum             TRKKRCPYTKHQTLELEKEFLFNMYLTRDRRYEVARLLNLTERQVKIWFQNRRMKMKKIN Homeobox protein Hox-A9 (Fragment) (HOXA9)
DH_1457  Xenopus laevis                  TRKKRCPYTKHQTLELEKEFLFNMYLTRDRRYEVARLLNLTERQVKIWFQNRRMKMKKIN LOC100037070 protein (hoxa9)
DH_8742  Xenopus laevis                  TRKKRCPYTKHQTLELEKEFLFNMYLTRDRRYEVARLLNLTERQVKIWFQNRRMKMKKIN Homebox protein Hoxa9 (Fragment) (hoxa9)
DH_434   Cavia porcellus                 TRKKRCPYTKHQTLELEKEFLFNMYLTRDRRYEVARLLNLTERQVKIWFQNRRMKMKKIN Homeobox protein Hox-A9 (Fragment) (HOXA9)
DH_8294  Pleurodeles waltlii             TRKKRCPYTKHQTLELEKEFLFNMYLTRDRRYEVARLLNLTERQVKIWFQNRRMKMKKIN Transcription factor HoxA9 (Fragment)
DH_4573  Homo sapiens                    TRKKRCPYTKHQTLELEKEFLFNMYLTRDRRYEVARLLNLTERQVKIWFQNRRMKMKKIN HOXA-9A (HOXA-9)
DH_8024  Latimeria menadoensis           TRKKRCPYTKHQTLELEKEFLFNMYLTRDRRYEVARLLNLTERQVKIWFQNRRMKMKKIN HoxA9 (Fragment)
DH_7274  Cavia sp                        TRKKRCPYTKHQTLELEKEFLFNMYLTRDRRYEVARLLNLTERQVKIWFQNRRMKMKKIN Guinea pig Hox-1.7 protein (GPK/5)
DH_8790  Xenopus borealis                TRKKRCPYTKHQTLELEKEFLFNMYLTRDRRYEVARLLNLTERQVKIWFQNRRMKMKKIN Homeobox protein (Fragment) (homeobox)
DH_11921 Gasterosteus aculeatus          TRKKRCPYTKHQTLELEKEFLYNMYLTRDRRLEVAGLLNLTERQVKIWFQNRRMKMKKLM groupVI
DH_6145  Oryzias latipes                 TRKKRCPYTKHQTLELEKEFLYNMYLTRDRRLEVAGLLNLTERQVKIWFQNRRMKMKKLM HoxD9b (hoxD9b)
DH_2168  Haplochromis burtoni            TRKKRCPYTKHQTLELEKEFLYNMYLTRDRRLEVAGLLNLTERQVKIWFQNRRMKMKKLM Hoxd9b (hoxd9b)
DH_643   Fugu rubripes                   TRKKRCPYTKHQTLELEKEFLYNMYLTRDRRLEVAGLLNLTERQVKIWFQNRRMKMKKLM Homeobox protein Hox-D9b (hoxd9b)
DH_6668  Fugu rubripes                   TRKKRCPYTKHQTLELEKEFLYNMYLTRDRRLEVAGLLNLTERQVKIWFQNRRMKMKKLM HoxD9b (Fragment)
DH_7717  Sphoeroides nephelus            -ERRRCPYTKHQTLELEKEFLYNMYLTRDRRLEVAGLLNLTERQVKIWFQNRRMKMKKLM HoxD9b (Fragment)
DH_6827  Oreochromis niloticus           TRNKRCPYTKRQTLELEKEFLYNMYLTRDRRLEVAGLLNLTERQVKIWFQNRRMKMKKLM Hox protein (Fragment)
DH_9382  Oryzias latipes                 TRKKRCPYTKRQTLELEKEFLYNMYLTRDRRLEVAGLLNLTERQVKIWFQNRRMKMKKLM HOXD9B (Fragment) (hoxd9b)
DH_436   Heterodontus francisci          TRKKRCPYTKHQTLELEKEFLFNMYLTRDRRYEVARVLNLTERQVKIWFQNRRMKMKKIN Homeobox protein Hox-A9 (HOXA9)
DH_7519  Lampetra japonica               SRKKRCPYTKFQTLELEKEFLFNMYLTRDRRYEVARVLSLTERQVKIWFQNRRMKMKKMN LjHox9r Homeobox (Fragment) (LjHox9r)
DH_8500  Petromyzon marinus              SRKKRCPYTKFQTLELEKEFLFNMYLTRDRRYEVARVLSLTERQVKIWFQNRRMKMKKMN HoxT9 homeobox (Fragment)
DH_524   Homo sapiens                    SRKKRCPYTKYQTLELEKEFLFNMYLTRDRRHEVARLLNLSERQVKIWFQNRRMKMKKMN Homeobox protein Hox-B9 (HOXB9)
DH_2846  Homo sapiens                    SRKKRCPYTKYQTLELEKEFLFNMYLTRDRRHEVARLLNLSERQVKIWFQNRRMKMKKMN cDNA, FLJ96541, Homo sapiens homeo box B9 (HOXB9), mRNA (Homeobox B9) (HOXB9)
DH_525   Mus musculus                    SRKKRCPYTKYQTLELEKEFLFNMYLTRDRRHEVARLLNLSERQVKIWFQNRRMKMKKMN Homeobox protein Hox-B9 (Hoxb9)
DH_1417  Mus musculus                    SRKKRCPYTKYQTLELEKEFLFNMYLTRDRRHEVARLLNLSERQVKIWFQNRRMKMKKMN Homeo box B9 (Hoxb9)
DH_7680  Pleurodeles waltlii             SRKKRCPYTKYQTLELEKEFLFNMYLTRDRRHEVARLLNLSERQVKIWFQNRRMKMKKMN Transcription factor HoxB9 (Fragment)
DH_526   Xenopus laevis                  SRKKRCPYSKYQTLELEKEFKFNMYLTRDRRHEVARLLNLSERQVKIWFQNRRMKMKKLN Homeobox protein Hox-B9 (hoxb9)
DH_6056  Xenopus laevis                  SRKKRCPYSKYQTLELEKEFLFNMYLTRDRRHEVARLLNLSERQVKIWFQNRRMKMKKLN Putative uncharacterized protein
DH_7571  Xenopus tropicalis              SRKKRCPYTKYQTLELEKEFLFNMYLTRDRRHEVARLLNLSERQVKIWFQNRRMKMKKLN Homeobox B9 (hoxb9)
DH_527   Danio rerio                     SRKKRCPYTKYQTLELEKEFLFNMYLTRDRRHEVARLLNLTERQVKIWFQNRRMKMKKMN Homeobox protein Hox-B9a (hoxb9a)
DH_1544  Megalobrama amblycephala        SRKKRCPYTKYQTLELEKEFLFNMYLTRDRRHEVARLLNLTERQVKIWFQNRRMKMKKMN Homeodomain protein (Fragment) (HoxB9a)
DH_3316  Salmo salar                     SRKKRCPYTKYQTLELEKEFLFNMYLTRDRRHEVARSLNLTERQVKIWFQNRRMKMKKMN Homeobox protein HoxB9ab (HoxB9ab)
DH_3345  Salmo salar                     SRKKRCPYTKYQTLELEKEFLFNMYLTRDRRHEVARSLNLTERQVKIWFQNRRMKMKKMN Homeobox protein HoxB9aa (HoxB9aa)
DH_528   Fugu rubripes                   SRKKRCPYTKYQTLELEKEFLFNMYLTRDRRHEVARALNLTERQVKIWFQNRRMKMKKQS Homeobox protein Hox-B9a (hoxb9a)
DH_6168  Oryzias latipes                 SRKKRCPYTKYQTLELEKEFLFNMYLTRDRRHEVARALNLTERQVKIWFQNRRMKMKKQS HoxB9a (hoxB9a)
DH_9381  Oryzias latipes                 SRKKRCPYTKYQTLELEKEFLFNMYLTRDRRHEVARALNLTERQVKIWFQNRRMKMKKQS HOXB9A (Fragment) (hoxb9a)
DH_2127  Haplochromis burtoni            SRKKRCPYTKYQTLELEKEFLFNMYLTRDRRHEVARALNLTERQVKIWFQNRRMKMKKQN Hoxb9a (hoxb9a)
DH_8499  Petromyzon marinus              GRKKACPNSKQQTLELEKEFLFNMYLTRDRRYEVARGLNLTERQVKIWFQNRRMKLKKMK HoxV9 homeobox (Fragment)
DH_9242  Branchiostoma floridae          TRKKRCPYTKYQTLELEKEFLFNMFVTRERRQEIARQLNLTDRQVKIWFQNRRMKMKRMK Homeodomain-containing protein Hox11 (Fragment) (Hox11)
DH_11597 Branchiostoma floridae          TRKKRCPYTKYQTLELEKEFLFNMFVTRERRQEIARQLNLTDRQVKIWFQNRRMKMKRMK e_gw.260.82.1|Brafl1
DH_12687 Branchiostoma floridae          TRKKRCPYTKYQTLELEKEFLFNMFVTRERRQEIARQLNLTDRQVKIWFQNRRMKMKRMK AmphiHox11
DH_11551 Branchiostoma floridae          TRKKRCPYTKYQTLELEKEFLFNMFVTRERRQEIARQLNLTDRQVCNKIYIKI------- e_gw.260.85.1|Brafl1
DH_12626 Strongylocentrotus purpuratus   GRKKRCPYTKFQTLELEKEFLFNMYLTRDRRLEIARLLSLTERQVKIWFQNRRMKMKKQN GLEAN3_02633|Sp-Hox9/10
DH_9274  Tripneustes gratilla            GRKKRCPYTKFQTLELEKEFLFNMYLTRDRRLEIARLLSLTERQVKIWFQNRRMKMKKQN Homeodomain protein TgHBox4
DH_268   Tripneustes gratilla            GRKKRCPYTKFQTLELEKEFLFNMYLTRDRRLEIARLLSLTERQVKIWFQNRRMKMKKQN Homeobox protein HB4 (Fragment)
DH_1264  Saccoglossus kowalevskii        GRKKRCPYTKFQTLELEKEFLFNMYLTRERRVDIARLLNLTERQVKIWFQNRRMKLKKQN Hox 9/10
DH_7668  Ptychodera flava                GRKKRCPYTKFQTLELEKEFLFNMYLTRERRVEISRLLNLTERQVKIWFQNRRMKLKKQN Transcription factor Hox9/10
DH_1329  Metacrinus rotundus             GRKKRCPYTKFQTLELEKEFLFNMYLTRDRRVEIARLLNLTERQVKIWFQNRRMKMKKMN Transcription factor Hox9/10 (MrHox9/10)
DH_12652 Strongylocentrotus purpuratus   TRKKRKPYTKFQTFELEKEFLYNMYLTRDRRSHISRALSLTERQVKIWFQNRRMKLKKMR GLEAN3_02632|Sp-Hox11/13a
DH_8870  Heliocidaris erythrogramma      TRKKRKPYTKFQTFELEKEFLYNMYLTRDRRSHISRALSLTERQVKIWFQNRRMKLKKMR HEHBOX10 (Fragment) (HeHbox10)
DH_1265  Saccoglossus kowalevskii        NRKKRKPYTKYQTLELEKEFLYNMYLTRDRRTDIARALNLSERQIKIWFQNRRMKLKKMR Hox 11/13a
DH_7667  Ptychodera flava                NRKKRKPYTKYQTLELEKEFLYNMYLTRDRRTDISRALNLTERQVKIWFQNRRMKLKKMR Transcription factor Hox11/13a
DH_2430  Branchiostoma floridae          SRKKRCPYTRFQTLELEKEFLYNMYLTRERRYEISQHVNLTERQVKIWFQNRRMKMKKMS AmphiHox9 (AmphiHox9)
DH_12685 Branchiostoma floridae          SRKKRCPYTRFQTLELEKEFLYNMYLTRERRYEISQHVNLTERQVKIWFQNRRMKMKKMS AmphiHox9
DH_5295  Branchiostoma floridae          SRKKRCPYTRFQTLELEKEFLYNMYLTRERRYEISQHVNLTERQVKIWFQNRRMKMKKMS Hox-9 homeodomain protein (Fragment)
DH_11629 Branchiostoma floridae          SRKKRCPYTRFQTLELEKEFLYNMYLTRERRYEISQHVNLTERQVKIWFQNRRMKMKKMS e_gw.402.47.1|Brafl1
DH_11636 Branchiostoma floridae          SRKKRCPYTRFQTLELEKEFLYNMYLTRERRYEISQHVNLTERQVKIWFQNRRMKMKKMS e_gw.260.52.1|Brafl1
DH_9241  Branchiostoma floridae          SRKKRCPYSKVQLLELEKEFLYNMYITREQRGEIARKVNLTDRQVKIWFQNRRMKMKRMK Homeodomain-containing protein Hox12 (Fragment) (Hox12)
DH_12688 Branchiostoma floridae          SRKKRCPYSKVQLLELEKEFLYNMYITREQRGEIARKVNLTDRQVKIWFQNRRMKMKRMK AmphiHox12
DH_11593 Branchiostoma floridae          SRKKRCPYSKVQLLELEKEFLYNMYITREQRGEIARKVNLTDRQVKIWFQNRRMKMKRMK gw.260.71.1|Brafl1
DH_11641 Branchiostoma floridae          SRKKRCPYSKVQLLELEKEFLYNMYITREQRGEIARKVNLTDRQVKIWFQNRRMKMKRMK gw.260.58.1|Brafl1
DH_6675  Oikopleura dioica               QRKKRVPYSRTQLLELEKEFRFNQYLSRDRRLELASLVNLTDRQVKIWFQNRRMKWKRER Hox9 (Fragment) (Hox9)
DH_7338  Oikopleura dioica               QRKKRVPYSRTQLLELEKEFRFNQYLSRDRRLELASLVNLTDRQVKIWFQNRRMKWKRER HOX9 (005-03)
DH_9234  Branchiostoma floridae          VRPKRRPYSKYQLNELENEYVQNQYISRDKRLQLSQKLNLTERQVKIWFQNRRIKQKKLD Homeodomain-containing protein Hox14 (Fragment) (Hox14)
DH_12690 Branchiostoma floridae          VRPKRRPYSKYQLNELENEYVQNQYISRDKRL---------------------------- AmphiHox14
DH_8628  Euprymna scolopes               LRKRRRPYSKYQIAELEREYALSTYISKSRRWELSQLLNLSERQIKIWFQNRRIKAKKLQ Posterior-1 homeodomain protein (Fragment) (Post1)
```

Save alignment [fasta format] - Save complete sequences [fasta format]

PG10

ID	organism	10 20 30 40 50 60	description
DH_3343	Salmo salar	GRKKRCPYTKYQTLELEKEYLFNMYLTRDRRLEIARSVNLTDRQVKIWFQNRRMKMKKMT	Homeobox protein HoxB10aa (HoxB10aa)
DH_3350	Salmo salar	GRKKRCPYSKYQTLELEKEFLFNVYLTRDRRLEIARSVNLTDRQVKIWFQNRRMKMKKMT	Homeobox protein HoxB10ab (HoxB10ab)
DH_7919	Symsagittifera roscoffensis	SRKKRRPYPKNQTLELEKEFLFNTYITRERRLEIARSLNLTDRQVKIWFQNRRMKNKKQH	Posterior class Hox protein Srpost (Fragment) (Post)
DH_1806	Lampetra japonica	GRKKRCPYTKYQTLELEKEFLFNMYLTRERRLEISRGVNLTDRQVKIWFQNRRMKLKKLS	LjHoxW10a homeobox (Fragment) (LjHoxW10a)
DH_8498	Petromyzon marinus	GRKKRCPYTKYQTLELEKEFLFNMYLTRERRLEISRGVNLTDRQVKIWFQNRRMKLKKLS	HoxW10a homeobox (Fragment)
DH_1807	Lampetra japonica	GRKKRCPYTKYQTLELEKEFLFNMYLTRERRLEISRGVNLTDRQVKIWFQNRRMKLKKMN	LjHox10s homeobox (Fragment) (LjHox10s)
DH_8497	Petromyzon marinus	GRKKRCPYTKYQTLELEKEFLFNMYLTRERRLEISRGVNLTDRQVKIWFQNRRMKLKKMN	HoxW10b homeobox (Fragment)
DH_11713	Gasterosteus aculeatus	GRKKRCPYTKHQTLELEKEFLFNMYLTRERRLEISRGVNLTDRQVKIWFQNRRMKLKKMN	Homeobox protein Hox-D10 (Hox-4D) (Hox-4E) [Source:UniProtKB/Swiss-Prot;Acc:P28358]
DH_3365	Salmo salar	GRKKRCPYTKHQTLELEKEFLFNMYLTRERRLEISKGVNLTDRQVKIWFQNRRMKLKKMS	Homeobox protein HoxD10aa (HoxD10aa)
DH_6778	Oncorhynchus mykiss	GRKKRCPYTKHQTLELEKEFLFNMYLTRERRLEISKGVNLTDRQVKIWFQNRRMKLKKMS	HoxD10ai (Fragment) (Hox)
DH_3272	Salmo salar	GRKKRCPYTKHQTLELEKEFLFNMYLTRERRLEISKGVNLTDRQVKIWFQNRRMKLKKMS	Homeobox protein HoxD10aa (HoxD10aa)
DH_3279	Salmo salar	GRKKRCPYTKHQTLELEKEFLFNMYLTRERRLEISKGVNLTDRQVKIWFQNRRMKLKKMN	Homeobox protein HoxD10ab (HoxD10ab)
DH_4432	Danio aff. tweediei	GRKKRCPYPKHQTLELEKEFLFNMYLTRERRLEISKSVNLTDRQVKIWFQNRRMKLKKMS	Hoxd10 (Fragment)
DH_4433	Devario cf. aequipinnatus	GRKKRCPYSKHQTLELEEEFLFNMYLTRERRLEISKSVNLTDRQVKIWFQNRRMKLKKMS	Hoxd10 (Fragment)
DH_7177	Pongo abelii	GRKKRCPYTKHQTLELEKEFLFNMYLTRERRLEVSKSVNLTDRQVKIWFQNRRMKLKKMS	Putative uncharacterized protein DKFZp469H0921 (DKFZp469H0921)
DH_588	Lagothrix lagotricha	GRKKRCPYTKHQTLELEKEFLFNMYLTRERRLEISKSVNLTDRQVKIWFQNRRMKLKKMS	Homeobox protein Hox-D10 (HOXD10)
DH_4437	Devario pathirana	GRKKRCPYTKHQTLELEKEFLFNMYLTRERRLEITKSVNLTDRQVKIWFQNRRMKLKKMS	Hoxd10 (Fragment)
DH_4435	Devario devario	GRKKRCPYTKHQTLELEKEFLFNMYLTRERRLEITKSVNLTDRQVKIWFQNRRMKLKKMS	Hoxd10 (Fragment)
DH_4436	Devario malabaricus	GRKKRCPYTKHQTLELEKEFLFNMYLTRERRLEITKSVNLTDRQVKIWFQNRRMKLKKMS	Hoxd10 (Fragment)
DH_4434	Danio frankei	GRKKRCPYTKHQTLELEKEFLFNMYLTRERRLEITKSVNLTDRQVKIWFQNRRMKLKKMS	Hoxd10 (Fragment)
DH_7561	Xenopus laevis	GRKKRCPYTKHQTLELEKEFLFNMYLTRERRLEISKSVNLTDRQVKIWFQNRRMKLKKMS	Hoxd10 protein (hoxd10)
DH_4620	Ambystoma mexicanum	GRKKRSPYTKHQTLELEKEFLFNMYLTRERRLEISKSVNLTDRQVKIWFQNRRMKLKKMS	Homeobox transcription factor (Fragment) (Hoxd-10)
DH_9344	Ambystoma mexicanum	GRKKRCPYTKHQTLELEKEFLFNMYLTRERRLEISKSVNLTDRQVKIWFQNRRMKLKKMS	Homeobox AHOX-4.5 protein (Fragment)
DH_1441	Mus musculus	GRKKRCPYTKHQTLELEKEFLFNMYLTRERRLEISKSVNLTDRQVKIWFQNRRMKLKKMS	Homeo box D10 (Hoxd10)
DH_589	Mus musculus	GRKKRCPYTKHQTLELEKEFLFNMYLTRERRLEISKSVNLTDRQVKIWFQNRRMKLKKMS	Homeobox protein Hox-D10 (Hoxd10)
DH_1811	Bos taurus	GRKKRCPYTKHQTLELEKEFLFNMYLTRERRLEISKSVNLTDRQVKIWFQNRRMKLKKMS	HOXD10 protein (HOXD10)
DH_591	Saguinus labiatus	GRKKRCPYTKHQTLELEKEFLFNMYLTRERRLEISKSVNLTDRQVKIWFQNRRMKLKKMS	Homeobox protein Hox-D10 (HOXD10)
DH_590	Pan troglodytes	GRKKRCPYTKHQTLELEKEFLFNMYLTRERRLEISKSVNLTDRQVKIWFQNRRMKLKKMS	Homeobox protein Hox-D10 (HOXD10)
DH_587	Homo sapiens	GRKKRCPYTKHQTLELEKEFLFNMYLTRERRLEISKSVNLTDRQVKIWFQNRRMKLKKMS	Homeobox protein Hox-D10 (HOXD10)
DH_585	Gallus gallus	GRKKRCPYTKHQTLELEKEFLFNMYLTRERRLEISKSVNLTDRQVKIWFQNRRMKLKKMS	Homeobox protein Hox-D10 (Fragment) (HOXD10)
DH_1619	Pongo pygmaeus	GRKKRCPYTKHQTLELEKEFLFNMYLTRERRLEISKSVNLTDRQVKIWFQNRRMKLKKMS	HOXD10 (Fragment) (HOXD10)
DH_4708	Rasbora paviei	GRKKRCPYTKHQTLELEKEFLFNMYLTRERRLEISKSVNLTDRQVKIWFQNRRMKLKKMS	Hoxd10 (Fragment)
DH_4707	Trigonostigma heteromorpha	GRKKRCPYTKHQTLELEKEFLFNMYLTRERRLEISKSVNLTDRQVKIWFQNRRMKLKKMS	Hoxd10 (Fragment)
DH_4700	Danio kerri	GRKKRCPYTKHQTLELEKEFLFNMYLTRERRLEISKSVNLTDRQVKIWFQNRRMKLKKMS	Hoxd10 (Fragment)
DH_2179	Danio rerio	GRKKRCPYTKHQTLELEKEFLFNMYLTRERRLEISKSVNLTDRQVKIWFQNRRMKLKKMS	Hoxd10a protein (Homeo box D10a) (hoxd10a)
DH_644	Danio rerio	GRKKRCPYTKHQTLELEKEFLFNMYLTRERRLEISKSVNLTDRQVKIWFQNRRMKLKKMS	Homeobox protein Hox-D10a (hoxd10a)
DH_4701	Danio albolineatus pulcher	GRKKRCPYTKHQTLELEKEFLFNMYLTRERRLEISKSVNLTDRQVKIWFQNRRMKLKKMS	Hoxd10 (Fragment)
DH_4704	Pseudorasbora cf. parva	GRKKRCPYTKHQTLELEKEFLFNMYLTRERRLEISKSVNLTD------------------	Hoxd10 (Fragment)
DH_6389	Tetraodon nigroviridis	GRKKRCPYTKHQTLELEKEFLFNMYLTRERRLEISRSVHLTDRQGKSTLFAGSWNPITAH	Chromosome 8 SCAF15044, whole genome shotgun sequence (GSTENG00034117001)
DH_4709	Tanichthys albonubes	GRKKRCPYTKHQTLELEKEFSFNMYLTRERRLEISKSVNLTD------------------	Hoxd10 (Fragment)
DH_8746	Xenopus laevis	GRKKRCPYTKHHTLELEKEFLFNMYLTRERRLEISKSVNLTDRQVNIWL-----------	Homeobox protein, Hoxd10 (Fragment) (hoxd10)
DH_4431	Danio aff. albolineatus	GRKKRCPYTKHQTLELEKEFLFNMYLTRERRLEISKSVNLTDRQVKIWFPNRRMKLKKMS	Hoxd10 (Fragment)
DH_4706	Rasbora elegans	GRKKRCPYTKHQTLELEKEFLFNMYLTRERRLEISKSVNLTDRQVKIWFQNRRMELKKMS	Hoxd10 (Fragment)
DH_6417	Tetraodon nigroviridis	GRKKRCPYTKHQTLELEKEFLFNMYLSRERRLEISRSVNLTDRQVKIWFQNRRMKLKKMS	Chromosome 2 SCAF15010, whole genome shotgun sequence (GSTENG00031461001)
DH_645	Fugu rubripes	GRKKRCPYTKHQTLELEKEFLFNMYLTRERRLEISRSVNLTDRQVKIWFQNRRMKLKKMS	Homeobox protein Hox-D10a (hoxd10a)
DH_7999	Latimeria menadoensis	GRKKRCPYTKHQTLELEKEFLFNMYLTRERRLEISRSVNLTDRQVKIWFQNRRMKLKKMS	HoxD10 (Fragment)
DH_586	Heterodontus francisci	GRKKRCPYTKYQTLELEKEFLFNMYLTRERRLEISKSVNLTDRQVKIWFQNRRMKLKKMN	Homeobox protein Hox-D10 (HOXD10)
DH_4816	Scyliorhinus canicula	GRKKRCPYTKYQTLELEKEFLFNMYLTRERRLEISKSVNLTDRQVKIWFQ----------	Hoxd10 (Fragment)
DH_532	Homo sapiens	GRKKRCPYTKHQTLELEKEFLFNMYLTRERRLEISKTINLTDRQVKIWFQNRRMKLKKMN	Homeobox protein Hox-C10 (HOXC10)
DH_6738	Homo sapiens	GRKKRCPYTKHQTLELEKEFLFNMYLTRERRLEISKTINLTDRQVKIWFQNRRMKLKKMN	Homeo box C10 (Homeobox C10, isoform CRA_a) (cDNA, FLJ94360, Homo sapiens homeo box C10 (HOXC10), mRNA) (HOXC10)
DH_1387	Pan paniscus	GRKKRCPYTKHQTLELEKEFLFNMYLTRERRLEISKTINLTDRQVKIWFQNRRMKLKKMN	HOXC10 (Fragment) (HOXC10)
DH_533	Macaca mulatta	GRKKRCPYTKHQTLELEKEFLFNMYLTRERRLEISKTINLTDRQVKIWFQNRRMKLKKMN	Homeobox protein Hox-C10 (HOXC10)
DH_534	Macaca nemestrina	GRKKRCPYTKHQTLELEKEFLFNMYLTRERRLEISKTINLTDRQVKIWFQNRRMKLKKMN	Homeobox protein Hox-C10 (HOXC10)
DH_535	Mus musculus	GRKKRCPYTKHQTLELEKEFLFNMYLTRERRLEISKTINLTDRQVKIWFQNRRMKLKKMN	Homeobox protein Hox-C10 (Hoxc10)
DH_8322	Mus musculus	GRKKRCPYTKHQTLELEKEFLFNMYLTRERRLEISKTINLTDRQVKIWFQNRRMKLKKMN	Putative uncharacterized protein (Hoxc10)
DH_536	Pongo pygmaeus	GRKKRCPYTKHQTLELEKEFLFNMYLTRERRLEISKTINLTDRQVKIWFQNRRMKLKKMN	Homeobox protein Hox-C10 (HOXC10)
DH_1370	Saguinus labiatus	GRKKRCPYTKHQTLELEKEFLFNMYLTRERRLEISKTINLTDRQVKIWFQNRRMKLKKMN	HOXC10 (Fragment) (HOXC10)
DH_1592	Pan troglodytes	GRKKRCPYTKHQTLELEKEFLFNMYLTRERRLEISKTINLTDRQVKIWFQNRRMKLKKMN	HOXC10 (Fragment) (HOXC10)
DH_1477	Ateles geoffroyi	GRKKRCPYTKHQTLELEKEFLFNMYLTRERRLEISKTINLTDRQVKIWFQNRRMKLKKMN	HOXC10 (Fragment) (HOXC10)
DH_1492	Lagothrix lagotricha	GRKKRCPYTKHQTLELEKEFLFNMYLTRERRLEISKTINLTDRQVKIWFQNRRMKLKKMN	HOXC10 (Fragment) (HOXC10)
DH_9108	Ambystoma mexicanum	GRKKRCPYTKHQTLELEKEFLFNMYLTRERRLEISKSINLTDRQVKIWFQNRRMKLKKMN	Homeodomain protein Hoxc10 long form (Hoxc10)
DH_7396	Xenopus laevis	GRKKRCPYTKHQTLELEKEFLFNMYLTRERRLEISKSINLTDRQVKIWFQNRRMKLKKMN	Hoxc10 protein (hoxc10)
DH_8032	Xenopus laevis	GRKKRCPYTKHQTLELEKEFLFNMYLTRERRLEISKSINLTDRQVKIWFQNRRMKLKKMN	Hoxc10

DH_9107	Ambystoma mexicanum	GRKKRCPYTKHQTLELEKEFLFNMYLTRERRLEISKSINLTDRQVKIWFQNRRMKLKKMN	Homeodomain protein Hoxc10 short form (Hoxc10)
DH_8006	Latimeria menadoensis	GRKKRCPYTKHQTLELEKEFLFNMYLTRERRLEISKSINLTDRQVKIWFQNRRMKLKKMN	HoxC10 (Fragment)
DH_1551	Megalobrama amblycephala	GRKKRCPYTKHQTLELEKEFLFNMYLTRERRLEISKSINLTDRQVKIW------------	Homeodomain protein (Fragment) (HoxC10a)
DH_11780	Gasterosteus aculeatus	GRKKRCPYTKHQTLELEKEFLFNMYLSRERRLEISRSINLTDRQVKIWFQNRRMKLKKLN	groupXII
DH_2139	Haplochromis burtoni	GRKKRCPYTKHQTLELEKEFLFNMYLSRERRLEISRSINLTDRQVKIWFQNRRMKLKKLN	Hoxc10a (hoxc10a)
DH_6156	Oryzias latipes	GRKKRCPYTKHQTLELEKEFLFNMYLSRERRLEISRSINLTDRQVKIWFQNRRMKLKKLN	HoxC10a (hoxC10a)
DH_6833	Oreochromis niloticus	GRKKRCPYTKHQTLELEKEFLFNMYLSRERRLEISRSINLTDRQVKIWFQNRRMKLKKLN	Hox protein (Fragment)
DH_577	Fugu rubripes	GRKKRCPYTKHQTLELEKEFLFNMYLSRERRLEISRSINLTDRQVKIWFQNRRMKLKKLN	Homeobox protein Hox-C10a (hoxc10a)
DH_6406	Tetraodon nigroviridis	GRKKRCPYTKHQTLELEKEFLFNMYLSRERRLEISRSINLTDRQVKIWFQNRRMKLKKLN	Chromosome 3 SCAF15018, whole genome shotgun sequence. (Fragment) (GSTENG00031863001)
DH_6450	Tetraodon nigroviridis	GRKKRCPYTKHQTLELEKEFLFNMYLSRERRLEISRSINLTDRQVKIWFQNRRMKLKKLN	Chromosome 9 SCAF14991, whole genome shotgun sequence (GSTENG00028041001)
DH_3236	Salmo salar	GRKKRCPYTKHQTLELEKEFLYNMYLSRERRLEISKSIDLTDRQVKIWFQNRRMKLKKLN	Homeobox protein HoxC10aa (HoxC10aa)
DH_3245	Salmo salar	GRKKRCPYTKYQTLELEKEFLFNMYLSRERRLEISKSIDLTDRQVKIWFQNRRMKLKKLN	Homeobox protein HoxC10ab (HoxC10ab)
DH_1464	Danio rerio	GRKKRCPYTKHQTLELEKEFLFNMYLTRERRLEISKSINLTDRQVKIWFQNRRMKLKKLN	Homeo box C10a (hoxc10a)
DH_9651	Danio rerio	GRKKXCPNTKHQTLELEKEFLFNMYLTRERRLEISKSINLTDRQVKIWFQNRRMKLKKLN	Homeobox protein (Fragment) (hoxc10a)
DH_3255	Salmo salar	RRKKRCPYTKHQILELEKEFLFNMYLTRERRLEISKSIDLSDRQVKIWFQNRRMKLKKFN	Homeobox protein HoxC10ba (HoxC10ba)
DH_4469	Fugu rubripes	GRKKRCPYTKHQTLELEKEFLFNMYLTRERRLEISKVSTLTDRQVKIWFQNRRMKLKKMT	Homeobox protein HOXA-10 (FrHOXA-10)
DH_8496	Petromyzon marinus	TRKKRCPYTKHQTLELEKEFLFSMYLTRERRLEISHLLSLTDRQVKIWFQNRRMKLKKMN	HoxX10 homeobox (Fragment)
DH_358	Morone saxatilis	GRKKRCPYTKHQTLELEKEFLFNMYLTRERRLEISKSVHLTDRQVKIWFQNRRMKLKKMT	Homeobox protein Hox-A10 (hoxa10)
DH_2149	Haplochromis burtoni	GRKKRCPYTKHQTLELEKEFLFNMYLTRERRLEISKSVHLTDRQVKIWFQNRRMKLKKMT	Hoxa10a (hoxa10a)
DH_6183	Oryzias latipes	GRKKRCPYTKHQTLELEKEFLFNMYLTRERRLEISKSVHLTDRQVKIWFQNRRMKLKKMT	HoxA10a (hoxA10a)
DH_444	Fugu rubripes	GRKKRCPYTKHQTLELEKEFLFNMYLTRERRLEISKSVHLTDRQVKIWFQNRRMKLKKMT	Homeobox protein Hox-A10a (hoxa10a)
DH_11843	Gasterosteus aculeatus	GRKKRCPYSKHQTLELEKEFLFNMYLTRERRLEISKSVHLTDRQVKIWFQNRRMKLKKMT	groupX
DH_3332	Salmo salar	GRKKRCPYSKHQTLELEKEFLFNMYLTRERRLEISRSVHLTDRQVKIWFQNRRMKLKKMT	Homeobox protein HoxA10aa (HoxA10aa)
DH_2442	Danio rerio	GRKKRCPYSKHQILELEKEFLFNMYLTRERRLEISRSINLTDRQVKIWFQNRRMKLKKMT	Hoxb10a protein (hoxb10a)
DH_2932	Danio rerio	GRKKRCPYSKHQILELEKEFLFNMYLTRERRLEISRSINLTDRQVKIWFQNRRMKLKKMT	Homeo box B10a (hoxb10a)
DH_529	Danio rerio	GRKKRCPYSKHQILELEKEFLFNMYLTRERRLEISRSINLTDRQVKIWFQNRRMKLKKMT	Homeobox protein Hox-B10a (Fragment) (hoxb10a)
DH_3294	Salmo salar	GRKKRCPYTKHQTLELEKEFLYNMYLTRERRLEISRSVHLTDRQVKIWFQNRRMKLKKMT	Homeobox protein HoxA10ab (HoxA10ab)
DH_446	Fugu rubripes	GRKKRCPYTKHQTLELEKEFLFNMYLTRERRLEISRSVHLTDRQVKIWFQNRRMKLKKMT	Homeobox protein Hox-A10b (hoxa10b)
DH_1654	Carassius auratus	-RKKRVPYTKHQTLELEKEFLFNMYLTRERRLEISRSVHLTDRQVKIWSKTAG-------	HoxA10b protein (Fragment) (Hox)
DH_1655	Carassius auratus	-RKKRVPYTKHQTLELEKEFLFNMYLTRERRLEISRSVHLTDRQVKIWFQTAG-------	HoxA10b protein (Fragment) (Hox)
DH_1657	Carassius auratus	-RKKRVPYTKHQTLELEKEFLFNMYLTRERRLEISRSVHLTDRQVKIWFQNRRM------	HoxA10b protein (Fragment) (Hox)
DH_356	Heterodontus francisci	GRKKRCPYTKHQTLELEKEFLFNMYLTRERRLEISRSVHLTDRQVKIWFQNRRMKLKKMN	Homeobox protein Hox-A10 (HOXA10)
DH_8023	Latimeria menadoensis	GRKKGCPYTKHQTLELEKEFLFNMYLTRERRLEISRSVHLTDRQVKIWFQNRRMKLKKMN	HoxA10 (Fragment)
DH_2578	Callithrix jacchus	GRKKRCPYTKHQTLELEKEFLFNMYLTRERRLEISRSVHLTDRQVKIWFQNRRMKLKKMN	Homeobox A10 isoform a (Predicted) (HOXA10)
DH_2748	Callicebus moloch	GRKKRCPYTKHQTLELEKEFLFNMYLTRERRLEISRSVHLTDRQVKIWFQNRRMKLKKMN	Homeobox A10 isoform a (Predicted) (HOXA10)
DH_357	Homo sapiens	GRKKRCPYTKHQTLELEKEFLFNMYLTRERRLEISRSVHLTDRQVKIWFQNRRMKLKKMN	Homeobox protein Hox-A10 (HOXA10)
DH_2813	Rhinolophus ferrumequinum	GRKKRCPYTKHQTLELEKEFLFNMYLTRERRLEISRSVHLTDRQVKIWFQNRRMKLKKMN	Homeobox A10 isoform a (Predicted) (HOXA10)
DH_359	Mus musculus	GRKKRCPYTKHQTLELEKEFLFNMYLTRERRLEISRSVHLTDRQVKIWFQNRRMKLKKMN	Homeobox protein Hox-A10 (Hoxa10)
DH_9132	Sus scrofa	GRKKRCPYTKHQTLELEKEFLFNMYLTRERRLEISRSVHLTDRQVKIWFQNRRMKLKKMN	Homeobox protein A10 (Fragment)
DH_2449	Papio anubis	GRKKRCPYTKHQTLELEKEFLFNMYLTRERRLEISRSVHLTDRQVKIWFQNRRMKLKKMN	Homeobox A10, isoform 1 (Predicted) (HOXA10)
DH_1853	Bos taurus	GRKKRCPYTKHQTLELEKEFLFNMYLTRERRLEISRSVHLTDRQVKIWFQNRRMKLKKMN	HOXA10 protein (HOXA10)
DH_6689	Homo sapiens	GRKKRCPYTKHQTLELEKEFLFNMYLTRERRLEISRSVHLTDRQVKIWFQNRRMKLKKMN	HOXA10 protein (Homeobox A10, isoform CRA_c) (cDNA, FLJ92804, Homo sapiens homeo box A10 (HOXA10), transcript variant
DH_11774	Gasterosteus aculeatus	GRKKRCPYTKHQTLELEKEFLFNMYLTRERRLEISRSVHLTDRQVKIWFQNRRMKLKKMS	groupXX
DH_2133	Haplochromis burtoni	GRKKRCPYTKHQTLELEKEFLFNMYLTRERRLEISRSVHLTDRQVKIWFQNRRMKLKKMS	Hoxa10b (hoxa10b)
DH_3303	Salmo salar	GRKKRCPYTKHQTLELEKEFLFNMYLTRERRLEISRSVHLTDRQVKIWFQNRRMKLKKMS	Homeobox protein HoxA10b (HoxA10b)
DH_3342	Salmo salar	GRKKRCPYTKHQTLELEKEFLFNMYLTRERRLEISRSVHLTDRQVKIWFQNRRMKLKKMS	Homeobox protein HoxA10b (HoxA10b)
DH_6178	Oryzias latipes	GRKKRCPYTKHQTLELEKEFLFNMYLTRERRLEISRSVHLTDRQVKIWFQNRRMKLKKMS	HoxA10b (hoxA10b)
DH_6723	Oreochromis niloticus	GRKKRCPYTKHQTLELEKEFLFNMYLTRERRLEISRSVHLTDRQVKIWFQNRRMKLKKMS	Hox protein (Fragment)
DH_445	Danio rerio	GRKKRCPYTKHQTLELEKEFLFNMYLTRERRLEISRSVHLTDRQVKIWFQNRRMKLKKMS	Homeobox protein Hox-A10b (hoxa10b)
DH_2899	Danio rerio	GRKKRCPYTKHQTLELEKEFLFNMYLTRERRLEISRSVHLTDRQVKIWFQNRRMKLKKMS	Homeo box A10b (hoxa10b)
DH_1529	Megalobrama amblycephala	GRKKRCPYTKHQTLELEKEFLFNMYLTRERRLEISRSVHLTDRQVKIWFQNRRMKLKKMS	Homeodomain protein (Fragment) (HoxA10b)
DH_2164	Haplochromis burtoni	GRKKRCPYTKHQTLELEKEFLFNMYLTRERRLEISRSVNLTDRQVKIWFQNRRMKLKKMN	Hoxd10a (hoxd10a)
DH_6703	Oreochromis niloticus	GRKKRCPYTKHQTLELEKEFLFNMYLTRERRLEISRSVNLTDRQVKIWFQNRRMKLKKMN	Hox protein (Fragment)
DH_6149	Oryzias latipes	GRKKRCPYTKHQTLELEKEFLFNMYLTRERRLEISRSVNLTDRQVKIWFQNRRMKLKKMN	HoxD10a (hoxD10a)
DH_5287	Branchiostoma floridae	GRKKRCPYTKYQILELEKEFLFNMYVSRERRQEISRHVNLSDRQVKIWFQNRRMKMKRMN	Hox-10 homeodomain protein (Fragment)
DH_12686	Branchiostoma floridae	GRKKRCPYTKYQILELEKEFLFNMYVSRERRQEISRHVNLSDRQVKIWFQNRRMKMKRMN	AmphiHox10
DH_11573	Branchiostoma floridae	GRKKRCPYTKYQILELEKEFLFNMYVSRERRQEISRHVNLSDRQVKIWFQNRRMKMKRMN	e_gw.260.79.1|Brafl1
DH_9240	Branchiostoma floridae	GRKKRCPYTKYQLSVLEQEYIQNRYVSRETRLELSQRLNLTDRQVKIWFQNRRMKQKRLE	Homeodomain-containing protein Hox13 (Fragment) (Hox13)
DH_12689	Branchiostoma floridae	GRKKRCPYTKYQLSVLEQEYIQNRYVSRETRLELSQRLNLTDRQVKIWFQNRRMKQKRLE	AmphiHox13
DH_11608	Branchiostoma floridae	GRKKRCPYTKYQLSVLEQEYIQNRYVSRETRLELSQRLNLTDRQVKIWFQNRRMKQKRLE	e_gw.260.41.1|Brafl1
DH_11615	Branchiostoma floridae	GRKKRCPYTKYQLSVLEQEYIQNRYVSRETRLELSQRLNLTDRQVKIWFQNRRMKQKRLE	e_gw.260.40.1|Brafl1
DH_5674	Styela clava	GRKKRVPYTKYQLLELEKEFHYNQYLSRERRQEVAKAVSLSDRQVKIWFQNRRMKWKKEK	Homeobox protein (Fragment)
DH_5690	Styela plicata	GRKKRVPYTKYQLLELEKEFHYNQYLSRERRQEVAKAVSLSDRQVKIWFQNRRMKWKKEK	Homeobox protein (Fragment)
DH_5649	Styela clava	GRKKRVPYTKYQLLELEKEFHYNQYLSRERRQEVAKAVSLSDRQVKIWFQNRRMKWKKEK	DNA binding protein AHox2 (Fragment) (AHox2)
DH_6282	Ciona intestinalis	GRKKRVPYTKYQLLELEKEFHYNQYLSRERRLEVAKSVKLTDRQVKIWFQNRRMKWKKER	Transcription factor protein (Ci-Hox10)
DH_8357	Ciona intestinalis	GRKKRVPYTKYQLLELEKEFHYNQYLSRERRLEVAKSVKLTDRQVKIWFQNRRMKWKKER	Putative homeobox protein Hox10 (hox10)
DH_6674	Oikopleura dioica	GRKKRVPYTKFQLLELEKEFHFNQYLSRERRLEVAKNVGLTDRQVKIWFQNRRMKWKKER	Hox10 (Fragment) (Hox10)
DH_7333	Oikopleura dioica	GRKKRVPYTKFQLLELEKEFHFNQYLSRERRLEVAKNVGLTDRQVKIWFQNRRMKWKKER	Hox10 (008-19)

Save alignment [fasta format] - Save complete sequences [fasta format]

PG11

ID	organism	10 20 30 40 50 60	description
DH_3278	Salmo salar	SRKKRCPYSKFQIRELEREFFFNVYINKEKRLQMTRKLNLTDRQVKIWFQNRRMKEKKLN	Homeobox protein HoxD11ab (HoxD11ab)
DH_8494	Petromyzon marinus	SRKKRCPYTKFQIRELEREFFFNVYINKEKRLQLSRLLNLTDRQVKIWFQNRRMREKKLN	HoxZ11a homeobox (Fragment)
DH_7997	Latimeria menadoensis	SRKKRCPYTKFQIRELEREFFFNVYINKEKRLQLSRMLNLTDRQVKIWFQNRRMKEKKLN	HoxD11 (Fragment)
DH_1553	Megalobrama amblycephala	--KKRCPYSKFQIRELEREFFFNVYINKEKGLQLSRILNLTDRHVKIWFQKRGMNEKKLS	Homeodomain protein (Fragment) (HoxC11b)
DH_8493	Petromyzon marinus	VRKKRCPYTKFQIRELEREFFFNVYINKEKRLQLSRLLNLTDRQVKIWFQNRRMSEKKLS	HoxZ11b homeobox (Fragment)
DH_9650	Danio rerio	--KKRCPYTKFQIRELEREFFFNVYINKEKRLQLSRILNLTDRQVKIWFQNRRMKEKKLS	Homeobox protein (Fragment) (hoxc11b)
DH_3254	Salmo salar	SRKKRCPYTKFQIRELEREFFFNVYINKEKRLQLSRMLNLTDRQVKIWFQNRRMKEKKLS	Homeobox protein HoxC11ba (Homeobox protein HoxC11bb) (HoxC11ba)
DH_3363	Salmo salar	SRKKRCPYTKFQIRELEREFFFNVYINKEKRLQLSRMLNLTDRQVKIWFQNRRMKEKKLS	Homeobox protein HoxC11bb (HoxC11bb)
DH_1465	Danio rerio	SRKKRCPYSKFQIRELEREFFFNVYINKEKRLQLSRMLNLTDRQVKIWFQNRRMKEKKLS	Homeo box C11a (hoxc11a)
DH_3244	Salmo salar	SRKKRCPYSKFQIRELEREFFFNVYINKEKRLQLSRMLNLTDRQVKIWFQNRRMKEKKLS	Homeobox protein HoxC11ab (HoxC11ab)
DH_5897	Rana temporaria	---------KFQIRELEREFFFNVYINKEKRLQLSRMLNLTDRQVKIWFQNRRMKEKKLS	HOXC11 (Fragment) (Hoxc11)
DH_2138	Haplochromis burtoni	TRKKRCPYSKFQIRELEREFFFNVYINKEKRLQLSRMLNLTDRQVKIWFQNRRMKEKKLS	Hoxc11a (hoxc11a)
DH_6155	Oryzias latipes	TRKKRCPYSKFQIRELEREFFFNVYINKEKRLQLSRMLNLTDRQVKIWFQNRRMKEKKLS	HoxC11a (hoxC11a)
DH_11786	Gasterosteus aculeatus	TRKKRCPYSKFQIRELEREFFFNVYINKEKRLQLSRMLNLTDRQVKIWFQNRRMKEKKLS	Homeobox protein Hox-C11 [Source:UniProtKB/Swiss-Prot;Acc:O43248]
DH_579	Fugu rubripes	TRKKRCPYSKFQIRELEREFFFNVYINKEKRLQLSRMLNLTDRQVKIWFQNRRMKEKKLS	Homeobox protein Hox-C11a (hoxc11a)
DH_578	Danio rerio	TRKKRCPYSKFQIRELEREFFFNVYINKEKRLQLSRMLNLTDRQVKIWFQNRRMKEKKLS	Homeobox protein Hox-C11a (hoxc11a)
DH_537	Homo sapiens	TRKKRCPYSKFQIRELEREFFFNVYINKEKRLQLSRMLNLTDRQVKIWFQNRRMKEKKLS	Homeobox protein Hox-C11 (HOXC11)
DH_2210	Homo sapiens	TRKKRCPYSKFQIRELEREFFFNVYINKEKRLQLSRMLNLTDRQVKIWFQNRRMKEKKLS	cDNA FLJ77163, highly similar to Homo sapiens homeo box C11 (HOXC11), mRNA (Homeobox C11) (HOXC11)
DH_538	Mus musculus	TRKKRCPYSKFQIRELEREFFFNVYINKEKRLQLSRMLNLTDRQVKIWFQNRRMKEKKLS	Homeobox protein Hox-C11 (Hoxc11)
DH_6708	Oreochromis niloticus	TRKKRCPYSKFQIRELEREFFFNVYINKEKRLQLSRMLNLTDRQVKIWFQNRRMKEKKLS	Hox protein (Fragment)
DH_1552	Megalobrama amblycephala	--KKCCPYSNFQIRELERELFFNVYINKEKRLQLSRMLNLTDCQVKIWFHNGRMKEKNLS	Homeodomain protein (Fragment) (HoxC11a)
DH_5896	Leiopelma archeyi	---------KFQIRELEREFFFNVYINKEKRLQLSRMLNLTDRQVKIWFQNRRMKEKNLA	HOXC11 (Fragment) (Hoxc11)
DH_11726	Gasterosteus aculeatus	SRKKRCPYTKYQIRELEREFFFNVYINKEKRLQLSRMLNLSDRQVKIWFQNRRMKEKKLN	groupXVI
DH_4622	Ambystoma mexicanum	SRKKRCPYTKYQIRELEREFFFNVYINKEKRLQLSRMLNLSDRQVKIWFQNRRMREKKLN	Homeobox transcription factor (Hoxd-11)
DH_647	Fugu rubripes	SRKKRCPYSKYQIRELEREFFFNVYINKEKRLQLSRMLNLSDRQVKIWFQNRRMKEKKLN	Homeobox protein Hox-D11a (hoxd11a)
DH_6148	Oryzias latipes	SRKKRCPYSKYQIRELEREFFFNVYINREKRLQLSRMLNLTDRQVKIWFQNRRMKEKKLN	HoxD11a (hoxD11a)
DH_6702	Oreochromis niloticus	SRKKRCPYSKYQIRELEREFFFNVYINKEKRLQLSRMLNLTDRQVKIWFQNRRMKEKKLN	Hox protein (Fragment)
DH_3271	Salmo salar	SRKKRCPYSKYQIRELEREFFFNVYINKEKRLQLSRMLNLTDRQVKIWFQNRRMKEKKLN	Homeobox protein HoxD11aa (HoxD11aa)
DH_646	Danio rerio	SRKKRCPYSKYQIRELEREFFFNVYINKEKRLQLSRMLSLTDRQVKIWFQNRRMKEKKLN	Homeobox protein Hox-D11a (hoxd11a)
DH_2557	Danio rerio	SRKKRCPYSKYQIRELEREFFFNVYINKEKRLQLSRMLSLTDRQVKIWFQNRRMKEKKLN	Homeo box D11a (hoxd11a)
DH_5497	Xenopus laevis	LRKKRCPYSKYQIRELEREFFFNVYINKEKRLQLSRMLNLTDRQVKIWFQNRRMKEKKLN	MGC131014 protein (hoxd11)
DH_2163	Haplochromis burtoni	SRKKRCPYTKYQIRELEREFFFNVYINKEKRLQLSRMLNLTDRQVKIWFQNRRMKEKKLN	Hoxd11a (hoxd11a)
DH_594	Homo sapiens	SRKKRCPYTKYQIRELEREFFFNVYINKEKRLQLSRMLNLTDRQVKIWFQNRRMKEKKLN	Homeobox protein Hox-D11 (HOXD11)
DH_1844	Homo sapiens	SRKKRCPYTKYQIRELEREFFFNVYINKEKRLQLSRMLNLTDRQVKIWFQNRRMKEKKLN	Putative uncharacterized protein HOXD11 (HOXD11)
DH_595	Mus musculus	SRKKRCPYTKYQIRELEREFFFNVYINKEKRLQLSRMLNLTDRQVKIWFQNRRMKEKKLN	Homeobox protein Hox-D11 (Hoxd11)
DH_1440	Mus musculus	SRKKRCPYTKYQIRELEREFFFNVYINKEKRLQLSRMLNLTDRQVKIWFQNRRMKEKKLN	Homeo box D11 (Homeobox D11, isoform CRA_b) (Hoxd11)
DH_5200	Homo sapiens	SRKKRCPYTKYQIRELEREFFFNVYINKEKRLQLSRMLNLTDRQVKIWFQNRRMKEKKLN	HOXD11 protein (HOXD11)
DH_592	Gallus gallus	SRKKRCPYTKYQIRELEREFFFNVYINKEKRLQLSRMLNLTDRQVKIWFQNRRMKEKKLN	Homeobox protein Hox-D11 (HOXD11)
DH_596	Notophthalmus viridescens	SRKKRCPYTKYQIRELEREFFFNVYINKEKRLQLSRMLNLTDRQVKIWFQNRRMKEKKLN	Homeobox protein Hox-D11 (HOXD11)
DH_447	Danio rerio	FRKKRCPYTKFQIRELEREFFFSVYINKEKRLQLSRMLNLTDRQVKMWFQNRRMKEKKLN	Homeobox protein Hox-A11a (hoxa11a)
DH_2933	Danio rerio	FRKKRCPYTKFQIRELEREFFFSVYINKEKRLQLSRMLNLTDRQVKIWFQNRRMKEKKLN	Hoxa11a protein (hoxa11a)
DH_8495	Petromyzon marinus	PRKKRCPYTKYQIRELEREFFFSVYINKEKRLQLSRLLNLTDRQVKIWFQNRRMKEKKLN	HoxY11 homeobox (Fragment)
DH_1808	Lampetra japonica	PRKKRCPYTKYQIRELEREFFFSVYINKEKRLQLSRLLNLTDRQVKIWFQNRRMKEKKLN	LjHox11t homeobox (Fragment) (LjHox11t)
DH_11771	Gasterosteus aculeatus	TRKKRCPYSKYQIRELEREFFFSVYINKEKRMQLSRMLNLTDRQVKIWFQNRRMKEKKLN	Homeobox protein Hox-A11 (Hox-1I) [Source:UniProtKB/Swiss-Prot;Acc:P31270]
DH_6177	Oryzias latipes	TRKKRCPYSKYQIRELEREFFFSVYINKEKRMQLSRMLNLTDRQVKIWFQNRRMKEKKLN	HoxA11b (hoxA11b)
DH_448	Fugu rubripes	TRKKRCPYSKYQIRELEREFFFSVYINKEKRMQLSRMLNLTDRQVKIWFQNRRMKEKKLN	Homeobox protein Hox-A11a (hoxa11a)
DH_6543	Tetraodon nigroviridis	TRKKRCPYSKYQIRELEREFFFSVYINKEKRMQLSRMLNLTDRQVKIWFQNRRMKEKKLN	Chromosome 21 SCAF14577, whole genome shotgun sequence (GSTENG00017477001)
DH_2132	Haplochromis burtoni	TRKKRCPYSKYQIRELEREFFFSVYINKEKRLQLSRMLNLTDRQVKIWFQNRRMKEKKLN	Hoxa11b (hoxa11b)
DH_6388	Tetraodon nigroviridis	TRKKRCPYSKYQIRELEREFFFSVYINKEKRLQLSRMLNLTDRQVKIWFQNRRMKEKKLN	Chromosome 8 SCAF15044, whole genome shotgun sequence (GSTENG00034118001)
DH_2148	Haplochromis burtoni	TRKKRCPYSKYQIRELEREFFFSVYINKEKRLQLSRMLNLTDRQVKIWFQNRRMKEKKLN	Hoxa11a (hoxa11a)
DH_6182	Oryzias latipes	TRKKRCPYSKYQIRELEREFFFSVYINKEKRLQLSRMLNLTDRQVKIWFQNRRMKEKKLN	HoxA11a (hoxA11a)
DH_11801	Gasterosteus aculeatus	TRKKRCPYSKYQIRELEREFFFSVYINKEKRLQLSRMLNLTDRQVKIWFQNRRMKEKKLN	Homeobox protein Hox-A11 (Hox-1I) [Source:UniProtKB/Swiss-Prot;Acc:P31270]
DH_4568	Xenopus laevis	----------YQIRELEREFFFSVYINKEKRLQLSRMLNLTDRQVKIWFQNRRMKEKKVN	Homeobox containing transcription factor (Fragment) (Hoxa-11)
DH_8619	Sus scrofa	--KKRCPYTKYQIRELEREFFFSVYINKEKRLQLSRMLNLTDRQVKIWFQNRRMKEKKIN	Homeobox A11 (Fragment)
DH_8743	Xenopus laevis	TRKKRCPYTKYQIRELEREFFFSVYINKEKRLQLSRMLNLTDRQVKIWFQNRRMKEKKIN	Homeobox protein Hox A11 (Fragment) (hoxa11)
DH_2867	Rattus norvegicus	TRKKRCPYTKYQIRELEREFFFSVYINKEKRLQLSRMLNLTDRQVKIWFQNRRMKEKKIN	RGD1566402 protein (RGD1566402)

```
DH_362   Mus musculus                TRKKRCPYTKYQIRELEREFFFSVYINKEKRLQLSRMLNLTDRQVKIWFQNRRMKEKKIN Homeobox protein Hox-A11 (Hoxa11)
DH_2814  Rhinolophus ferrumequinum   TRKKRCPYTKYQIRELEREFFFSVYINKEKRLQLSRMLNLTDRQVKIWFQNRRMKEKKIN Homeobox A11 (Predicted) (HOXA11)
DH_2579  Callithrix jacchus          TRKKRCPYTKYQIRELEREFFFSVYINKEKRLQLSRMLNLTDRQVKIWFQNRRMKEKKIN Homeobox A11 (Predicted) (HOXA11)
DH_2450  Papio anubis                TRKKRCPYTKYQIRELEREFFFSVYINKEKRLQLSRMLNLTDRQVKIWFQNRRMKEKKIN Homeobox A11 (Predicted) (HOXA11)
DH_3194  Sorex araneus               TRKKRCPYTKYQIRELEREFFFSVYINKEKRLQLSRMLNLTDRQVKIWFQNRRMKEKKIN Homeobox protein Hox-A11 (Predicted) (HOXA11)
DH_2749  Callicebus moloch           TRKKRCPYTKYQIRELEREFFFSVYINKEKRLQLSRMLNLTDRQVKIWFQNRRMKEKKIN Homeobox A11 (Predicted) (HOXA11)
DH_2834  Homo sapiens                TRKKRCPYTKYQIRELEREFFFSVYINKEKRLQLSRMLNLTDRQVKIWFQNRRMKEKKIN cDNA, FLJ94560, highly similar to Homo sapiens homeo box A11 (HOXA11), mRNA
DH_1691  Homo sapiens                TRKKRCPYTKYQIRELEREFFFSVYINKEKRLQLSRMLNLTDRQVKIWFQNRRMKEKKIN Homeo box A11 (Homeobox A11, isoform CRA_b) (HOXA11)
DH_361   Homo sapiens                TRKKRCPYTKYQIRELEREFFFSVYINKEKRLQLSRMLNLTDRQVKIWFQNRRMKEKKIN Homeobox protein Hox-A11 (HOXA11)
DH_360   Gallus gallus               TRKKRCPYTKYQIRELEREFFFSVYINKEKRLQLSRMLNLTDRQVKIWFQNRRMKEKKIN Homeobox protein Hox-A11 (HOXA11)
DH_6795  Oncorhynchus mykiss         TRKKRCPYTKYQIRELEREFFFSVYINKE------------------------------- HoxA11b-2 (Fragment) (HoxA11b-2)
DH_6796  Oncorhynchus mykiss         TRKKRCPYTKYQIRELEREFFFSVYINKE------------------------------- HoxA11bi (Fragment) (HoxA11bi)
DH_3284  Salmo salar                 TRKKRCPYTKYQIRELEREFFFSVYINKEKRLQLSRMLNLTDRQVKIWFQNRRMKEKKLS Homeobox protein HoxA11aa (HoxA11aa)
DH_3331  Salmo salar                 TRKKRCPYTKYQIRELEREFFFSVYINKEKRLQLSRMLNLTDRQVKIWFQNRRMKEKKLS Homeobox protein HoxA11aa (HoxA11aa)
DH_450   Danio rerio                 TRKKRCPYTKYQIRELEREFFFSVYINKEKRLQLSRMLNLTDRQVKIWFQNRRMKEKKLN Homeobox protein Hox-A11b (hoxa11b)
DH_2916  Danio rerio                 TRKKRCPYTKYQIRELEREFFFSVYINKEKRLQLSRMLNLTDRQVKIWFQNRRMKEKKLN Homeo box A11b (hoxa11b)
DH_1530  Megalobrama amblycephala    TRKKRCPYTKYQIRELEREFFFSVYINKEKRLQLSRMLNLTDRQVKIWFQNRRMKEKKLN Homeodomain protein (Fragment) (HoxA11b)
DH_449   Danio aequipinnatus         TRKKRCPYTKYQIRELEREFFFSVYINKEKRLQLSRMLNLTDRQVKIWFQNRRMKEKKLN Homeobox protein Hox-A11b (hoxa11b)
DH_3302  Salmo salar                 TRKKRCPYTKYQIRELEREFFFSVYINKEKRLQLSRMLNLTDRQVKIWFQNRRMKEKKLN Homeobox protein HoxA11b (HoxA11b)
DH_3341  Salmo salar                 TRKKRCPYTKYQIRELEREFFFSVYINKEKRLQLSRMLNLTDRQVKIWFQNRRMKEKKLN Homeobox protein HoxA11b (HoxA11b)
DH_3293  Salmo salar                 TRKKRCPYTKYQIRELEREFFFSVYINKEKRLQLSRMLNLTDRQVKIWFQNRRMKEKKLN Homeobox protein HoxA11ab (HoxA11ab)
DH_3337  Salmo salar                 TRKKRCPYTKYQIRELEREFFFSVYINKEKRLQLSRMLNLTDRQVKIWFQNRRMKEKKLN Homeobox protein HoxA11ab (HoxA11ab)
DH_8022  Latimeria menadoensis       TRKKRCPYTKYQIRELEREFFFSVYINKEKRLQLSRMLNLTDRQVKIWFQNRRMKEKKLN HoxA11 (Fragment)
DH_8491  Heterodontus francisci      TRKKRCPYTKYQIRELEREFFFSVYINKEKRLQLSRMLNLTDRQVKIWFQNRRMKEKKLN Homeobox A11 (hoxa11)
DH_1782  Polyodon spathula           IRKKRCPYTKYQIRELEREFFFSVYINKEKRLQLSRMLNLTDR----------------- Transcription factor HOXA11 (Fragment) (HoxA11)
DH_7371  Polyodon spathula           IRKKRCPYTKYQIRELEREFFFSVYINKEKRLQLSRMLNLTDRQVKIWFQNRRMKEKK-- Hoxa-11 (Fragment) (Hoxa-11)
DH_451   Fugu rubripes               ARKKRCPYTKYQIRELEREFFFSVYINKEKRLQLSRMLNLTDRQVKIWFQNRRMKEKKLN Homeobox protein Hox-A11b (hoxa11b)
DH_593   Heterodontus francisci      CRKKRCPYTKYQIRELEREFFFNVYINKEKRLQLSRMLNLTDRQVKIWFQNRRMKEKKLS Homeobox protein Hox-D11 (HOXD11)
DH_5022  Bos taurus                  TRKKRCPYSKFQIRELEREFFFNVYINKEKRLQLSRMLNLTDRQVKIWFQNRRM------ Homeobox C11 (Fragment) (HOXC11)
DH_3235  Salmo salar                 TRKKRCPYSKFQIRELEREFFFNVYINKEKRMQLSRMLNLTDRQVKIWFQNRRMKEKKLS Homeobox protein HoxC11aa (HoxC11aa)
DH_6529  Tetraodon nigroviridis      RRKKRCPYSKQQIRELEREFLFNIYINKDRRMQLSHLLRLTDRQVKIWFQNRRMKEKKLK Chromosome 17 SCAF14597, whole genome shotgun sequence. (Fragment) (GSTENG00018725001)
DH_6608  Tetraodon nigroviridis      -RKKRCPYSKQQIRELEREFLFNIYINKDRRMQLSHLLRLTDRCV--------------- Chromosome undetermined SCAF9085, whole genome shotgun sequence. (Fragment) (GSTENG00006619001)
```

Save alignment [fasta format] - Save complete sequences [fasta format]

PG12

ID	organism	10 20 30 40 50 60	description
DH_6672	Oikopleura dioica	GRKKRRPYTKLQIQELETEFRRTEFVTREQRQEISRRLNLTDRQVKIWFQNRRMKEKRLR	Hox12 (Fragment) (Hox12)
DH_7341	Oikopleura dioica	GRKKRRPYTKLQIQELETEFRRTEFVTREQRQEISRRLNLTDRQVKIWFQNRRMKEKRLR	HOX12 (003-24)
DH_6281	Ciona intestinalis	QRRRRRPYTKYQLSELEREFGANEFISREMREQIAVRVGLNDRQVKIWFQNRRMKKKRMQ	Transcription factor protein (Ci-Hox12)
DH_3270	Salmo salar	PRKKRKPYTKPQLAELENEYMMNEFINRQKRKELSERLDLSDQQVKIWFQNRRMKKKRLM	Homeobox protein HoxD12aa (HoxD12aa)
DH_3364	Salmo salar	PRKKRKPYTKPQLAELENEYMMNEFINRQKRKELSERLDLSDQQVKIWFQNRRMKKKRLM	Homeobox protein HoxD12aa (HoxD12aa)
DH_11746	Gasterosteus aculeatus	TRKKRKPYTKLQLAELENEFMMNEFINRQKRKDLSERLDLSDQQVKIWFQNRRMKKKRMM	Homeobox protein Hox-D12 (Hox-4H) [Source:UniProtKB/Swiss-Prot;Acc:P35452]
DH_650	Fugu rubripes	TRKKRKPYSKPQLAELENEFLMNEFINRQKRKELSDRLDLSDQQVKIWFQNRRMKKKRLM	Homeobox protein Hox-D12a (hoxd12a)
DH_6418	Tetraodon nigroviridis	TRKKRKPYSKPQLAELENEFLMNEFINRQKRKELSDRLDLSDQQVKIWFQNRRMKKKRLM	Chromosome 2 SCAF15010, whole genome shotgun sequence. (Fragment) (GSTENG00031460001)
DH_6147	Oryzias latipes	SRKKRKPYSKPQLAELENEFLMNEFINRQKRKELSDKLDLSDQQVKIWFQNRRMKKKRLM	HoxD12a (hoxD12a)
DH_6701	Oreochromis niloticus	TRKKRKPYSKPQLAELENEFMMNEFINRQKRKELSDRLDLSDQQVKIWFQNRRMKKKRLM	Hox protein (Fragment)
DH_2162	Haplochromis burtoni	TRKKRKPYSKLQLAELENEFMMNEFINRQKRKELSDRLDLSDQQVKIWFQNRRMKKKRLM	HoxD12a (hoxd12a)
DH_649	Danio rerio	SRKKRKPYTKPQLTELENEFMMNEFINRQKRKELSDRLELSDQQVKIWFQNRRMKKKRLM	Homeobox protein Hox-D12a (hoxd12a)
DH_2558	Danio rerio	SRKKRKPYTKPQLTELENEFMMNEFINRQKRKELSDRLELSDQQVKIWFQNRRMKKKRLM	Homeo box D12a (hoxd12a)
DH_1559	Megalobrama amblycephala	SRKKRKPYTKPQLAELEHEFMINEFINRQKRKELSDRLELSDQQVKIWFQNRRMKKKRLM	Homeodomain protein (Fragment) (HoxD12a)
DH_3277	Salmo salar	LRKKRKPYTKPQLAELENEFMVNEFITRQKRKELSDRLELSDQQVKIWFQNRRMKKKRLV	Homeobox protein HoxD12ab (HoxD12ab)
DH_600	Homo sapiens	ARKKRKPYTKQQIAELENEFLVNEFINRQKRKELSNRLNLSDQQVKIWFQNRRMKKKRVV	Homeobox protein Hox-D12 (HOXD12)
DH_4393	Homo sapiens	ARKKRKPYTKQQIAELENEFLVNEFINRQKRKELSNRLNLSDQQVKIWFQNRRMKKKRVV	Putative uncharacterized protein HOXD12 (Homeobox D12, isoform CRA_b) (HOXD12)
DH_601	Mus musculus	ARKKRKPYTKQQIAELENEFLVNEFINRQKRKELSNRLNLSDQQVKIWFQNRRMKKKRVV	Homeobox protein Hox-D12 (Hoxd12)
DH_8316	Mus musculus	ARKKRKPYTKQQIAELENEFLVNEFINRQKRKELSNRLNLSDQQVKIWFQNRRMKKKRVV	Putative uncharacterized protein (Hoxd12)
DH_597	Carollia perspicillata	ARKKRKPYTKQQIAELENEFLLNEFINRQKRKELSNRLNLSDQQVKIWFQNRRMKKKRVV	Homeobox protein Hox-D12 (HOXD12)
DH_598	Gallus gallus	SRKKRKPYTKQQIAELENEFLLNEFINRQKRKELSNRLNLSDQQVKIWFQNRRMKKKRVV	Homeobox protein Hox-D12 (HOXD12)
DH_599	Heterodontus francisci	SRRKRKPYTKQQIAELENEFLANEFINRQKRKELSDRLNLSDQQVKIWFQNRRMKKKRLV	Homeobox protein Hox-D12 (HOXD12)
DH_4815	Scyliorhinus canicula	SRRKRKPYTKQQIAELENEFLANEFINRQKRKELSDRLNLSDQQVKIWFQ----------	Hoxd12 (Fragment)
DH_7998	Latimeria menadoensis	SRKKRKPYTKQQIAHLENEFLINEFINRQKRKELSDRLNLSDQQVKIWFQNRRMKKKRLI	HoxD12 (Fragment)
DH_3262	Salmo salar	TRKKRKPYSKLQLAELEGDFMLNEFITRQRRRELSDRLNLSDQQVKIWFQNRRMKKKRLM	Homeobox protein HoxC12bb (HoxC12bb)
DH_6707	Oreochromis niloticus	TRKKRKPYSKLQLAELEGEFMLNEFITRQRRRELSDRLNLSDQQVKIWFQNRRMKKKRLM	Hox protein (Fragment)
DH_3253	Salmo salar	TRKKRKPYSKLQLAELEGEFMLNEFITRQRRRELSDRLNLSDQQVKIWFQNRRMKKKRLM	Homeobox protein HoxC12ba (HoxC12ba)
DH_6154	Oryzias latipes	TRKKRKPYSKLQLAELEGEFMLNEFITRQRRRELSDRLNLSDQQVKIWFQNRRMKKKRLM	HoxC12a (hoxC12a)
DH_2137	Haplochromis burtoni	TRKKRKPYSKLQLAELEGEFMLNEFITRQRRRELSDRLNLSDQQVKIWFQNRRMKKKRLM	Hoxc12a (hoxc12a)
DH_3234	Salmo salar	TRKKRKPYSKLQLAELEGEFMLNEFITRQRRRELSDRLNLSDQQVKIWFQNRRMKKKRLM	Homeobox protein HoxC12aa (HoxC12aa)
DH_1466	Danio rerio	TRKKRKPYSKLQLAELEGEFMLNEFITRQRRRELSDRLNLSDQQVKIWFQNRRMKKKRLL	Novel protein similar to vertebrate homeo box C12 (HOXC12) (Homeo box C12a) (hoxc12a)
DH_1554	Megalobrama amblycephala	TRKKRKPYSKLQLAELEGEFMLNEFITRQRRRELSDRLNLSDQQVKIWFQNRRM------	Homeodomain protein (Fragment) (HoxC12a)
DH_580	Fugu rubripes	TRKKRKPYSKLQLAELEGEFMMNEFITRQRRRELSDRLNLSDQQVKIWFQNRRMKKKRLM	Homeobox protein Hox-C12a (hoxc12a)
DH_6449	Tetraodon nigroviridis	TRKKRKPYSKLQLAELEGEFIMNEFITRQRRRELSDRLNLSDQQVKIWFQNRRMKKKRLM	Chromosome 9 SCAF14991, whole genome shotgun sequence (GSTENG00028042001)
DH_3243	Salmo salar	TRKKRKPYSKLQLSDLEGEFMLNEFITRQRRRELSNRLNLSDQQVKIWFQNRRMKKKRLM	Homeobox protein HoxC12ab (HoxC12ab)
DH_3360	Salmo salar	TRKKRKPYSKLQLSDLEGEFMLNEFITRQRRRELSNRLNLSDQQVKIWFQNRRMKKKRLM	Homeobox protein HoxC12ab (HoxC12ab)
DH_581	Danio rerio	TRKKRKPYSKLQLNELEGEFILNEFITRQRRRELSDRLNLTDQQVKIWFQNRRMKKKRLL	Homeobox protein Hox-C12b (hoxc12b)
DH_1555	Megalobrama amblycephala	TRKKRKPYSKLQLNELEGEFILNEFITRQRRKELSNQLNLTDQQVKIGFQNRRM------	Homeodomain protein (Fragment) (HoxC12b)
DH_539	Homo sapiens	SRKKRKPYSKLQLAELEGEFLVNEFITRQRRRELSDRLNLSDQQVKIWFQNRRMKKKRLL	Homeobox protein Hox-C12 (HOXC12)
DH_540	Mus musculus	SRKKRKPYSKLQLAELEGEFLVNEFITRQRRRELSDRLNLSDQQVKIWFQNRRMKKKRLL	Homeobox protein Hox-C12 (Hoxc12)
DH_8295	Pleurodeles waltlii	SRKKRKPYSKLQLAELEGEFLVNEFITRQRRRELSDRLNLSDQQVKIWFQNRRMKKKRLL	Transcription factor HoxC12
DH_8030	Latimeria menadoensis	SRKKRKPYSKLQLAELEGEFMVNEFITRQRRRELSDRLNLSDQQVKIWFQNRRMKKKRLL	HoxC12 (Fragment)
DH_8744	Xenopus laevis	SRKKRKPYSKIQLAELEGEFMVNEIITRQRRRELSDRLSLSDQQVKIWFQNRRMLRKRLL	Homeobox protein Hox C12 (Fragment) (hoxc12)
DH_6673	Oikopleura dioica	ARKKRRPYTKQQIAELEKEYMSSTYIAREKRQELGDRLNLSDRQVKVWFQNRRMKEKKLQ	Hox11 (Fragment) (Hox11)
DH_7339	Oikopleura dioica	ARKKRRPYTKQQIAELEKEYMSSTYIAREKRQELGDRLNLSDRQVKVWFQNRRMKEKKLQ	HOX11 (004-18)
DH_9477	Nereis virens	MRKKRKPYSKYQIAELEKEYVNNTYITKPKRWELSQRLNLSERQVKIWFQNRRMKEKKVT	Post1 homeobox protein (Fragment) (Post1)
DH_9488	Lingula unguis	MRKKRKPYSKYQIAELEREYVSNTYISKPKRWELSQRLQLSERQVKIWFQNRRMKEKKVK	Post-1 homeodomain protein (Fragment)
DH_12383	Helobdella robusta	QRKKRKPYTRYQTMVLENEFVSNSYITRQKRWEISCKLHLSERQVKVWFQNRRMKRKKLN	estExt_Genewise1Plus.C_180102|Helro1
DH_9476	Nereis virens	QRKKRKPYTRYQTMVLENEFMGNSYITRQKRWEISCKLHLSERQVKVWFQNRRMKRKKLN	Post2 homeobox protein (Fragment) (Post2)
DH_12183	Lottia gigantea	GRKKRKPYTRYQTMVLENEFLSSSYITRQKRWEISCKLQLSERQVKVWFQNRRMKRKKLT	gw1.12.448.1|Lotgi1
DH_9056	Dugesia japonica	SRKKRKPYTRYQTMVLESEFTGNAYITRQKRWEISCKLHLSERQVKVWFQNRRMKKKKLQ	Hox class homeodomain protein DjAbd-Bb (DjAbd-Bb)

Save alignment [fasta format] - Save complete sequences [fasta format]

PG13

ID	organism	10 20 30 40 50 60	description
DH_11556	Branchiostoma floridae	TRKKRRPYSKPQLALLEDEYASQKFLTKEKRKEISESSSLSERQVMIWFQNRRMKEKKLA	gw.260.84.1|Brafl1
DH_11558	Branchiostoma floridae	TRKKRRPYSKPQLALLEDEYASQKFLTKEKRKEISESSSLSERQVMIWFQNRRMKEKKLA	gw.260.83.1|Brafl1
DH_11682	Branchiostoma floridae	TRKKRRPYSKPQLALLEDEYASQKFLTKEKRKEISESSSLSERQVMIWFQNRRMKEKKLA	Hox15
DH_8021	Latimeria menadoensis	GRKKRVPYTKVQLKELEREYATNKFITKDKRRRISATTNLSERQVTIWFQNRRVKEKKVI	HoxA13 (Fragment)
DH_8745	Xenopus laevis	GRKKRVPYTKVQLKELEREYGTNKFITKDKRRRISATTNLSERQVTIWFQNRRVKEKKVI	Homeobox protein, Hoxa13 (hoxa13)
DH_364	Gallus gallus	GRKKRVPYTKVQLKELEREYATNKFITKDKRRRISATTNLSERQVTIWFQNRRVKEKKVI	Homeobox protein Hox-A13 (HOXA13)
DH_363	Ambystoma mexicanum	GRKKRVPYTKVQLKELEREYATNKFITKDKRRRISATTNLSERQVTIWFQNRRVKEKKVI	Homeobox protein Hox-A13 (Fragment) (HOXA13)
DH_3195	Sorex araneus	GRKKRVPYTKVQLKELEREYATNKFITKDKRRRISATTNLSERQVTIWFQNRRVKEKKVI	Hoxa13 protein (Predicted) (HOXA13)
DH_6204	Mus musculus	GRKKRVPYTKVQLKELEREYATNKFITKDKRRRISATTNLSERQVTIWFQNRRVKEKKVI	Hoxa13 protein (Hoxa13)
DH_6203	Mus musculus	GRKKRVPYTKVQLKELEREYATNKFITKDKRRRISATTNLSERQVTIWFQNRRVKEKKVI	Hoxa13 protein (Hoxa13)
DH_6205	Mus musculus	GRKKRVPYTKVQLKELEREYATNKFITKDKRRRISATTNLSERQVTIWFQNRRVKEKKVI	Hoxa13 protein (Hoxa13)
DH_366	Mus musculus	GRKKRVPYTKVQLKELEREYATNKFITKDKRRRISATTNLSERQVTIWFQNRRVKEKKVI	Homeobox protein Hox-A13 (Hoxa13)
DH_2451	Papio anubis	GRKKRVPYTKVQLKELEREYATNKFITKDKRRRISATTNLSERQVTIWFQNRRVKEKKVI	Homeobox A13 (Predicted) (HOXA13)
DH_7423	Homo sapiens	GRKKRVPYTKVQLKELEREYATNKFITKDKRRRISATTNLSERQVTIWFQNRRVKEKKVI	Homeobox A13 (HOXA13)
DH_1690	Homo sapiens	GRKKRVPYTKVQLKELEREYATNKFITKDKRRRISATTNLSERQVTIWFQNRRVKEKKVI	Homeo box A13 (HOXA13)
DH_365	Homo sapiens	GRKKRVPYTKVQLKELEREYATNKFITKDKRRRISATTNLSERQVTIWFQNRRVKEKKVI	Homeobox protein Hox-A13 (HOXA13)
DH_4291	Tadorna tadorna	-RKKSVPYTKVQLKELEREYATNKFITKDKRRRISATTNLSERQVTIWFQNRSM------	HoxA13 (Fragment)
DH_4290	Trachemys scripta elegans	-RKKRVPYTKVQLKELEREYATNKFITKDKRRRISATTNLSERQVTIWFQIAA-------	HoxA13 (Fragment)
DH_452	Danio rerio	GRKKRVPYTKVQLKELEREYATNKFITKDKRRRISAQTNLSERQVTIWFQNRRVKEKKVV	Homeobox protein Hox-A13a (hoxa13a)
DH_1678	Danio rerio	GRKKRVPYTKVQLKELEREYATNKFITKDKRRRISAQTNLSERQVTIWFQNRRVKEKKVV	Hoxa13a protein (hoxa13a)
DH_7370	Polyodon spathula	GRKKRVPYTKVQLKELEREYATNKFITKDKRRRISAQTNLSERQVTI-------------	Hoxa-13 (Fragment) (Hoxa-13)
DH_1783	Polyodon spathula	GRKKRVPYTKVQLKELEREYATNKFITKDKRRRISAQTNLSERQVTIWFQNRRVKEKKV-	Transcription factor HOXA13 (Fragment) (HoxA13)
DH_8492	Heterodontus francisci	GRKKRVPYTKVQLKELEREYATNKFITKDKRRRISASTNLSERQVTIWFQNRRVKEKKVL	Homeobox A13 (hoxa13)
DH_4282	Carassius auratus	-RKKSVPYTKVQLKELEREYATNKFITKDKRRRISAHTNLTERQVTMWFQNRRM------	HoxA13b (Fragment)
DH_4289	Ctenopharyngodon idella	-RKKSVPYTKVQLKELEREYATNKFITKDKRRRISAHTNLTERQVTMWFQNRRM------	HoxA13b (Fragment)
DH_4283	triploid hybrids of tetraploid male x Carassius cuvieri	-RKKSVPYTKVQLKDLEREYATNKFITKDKRRRISAHTNLTERQVTIWFQNSSM------	HoxA13b (Fragment)
DH_4288	Cyprinus carpio	-RKKSVPYTKVQLKELERGYATNKFITKDKRRRISAHTNLTERQVTIWFQNSRM------	HoxA13b (Fragment)
DH_4287	triploid hybrids of tetraploid male x Carassius cuvieri	-RKKSVPYTKVQLKELEREYATNKFITKDKRRRISAHTNLTERQVTIWFQNRSM------	HoxA13b (Fragment)
DH_4286	triploid hybrids of tetraploid male x Carassius cuvieri	-RKKRVPYTKVQLKELEREYATNKFITKDKRRRISAHTNLTERQVTIWFQNRSM------	HoxA13b (Fragment)
DH_4285	Carassius auratus x Cyprinus carpio	-RKKRVPYTKVQLKELERECATNKFITKDKRRRISAHTNLTERQVTIWFQNSRM------	HoxA13b (Fragment)
DH_1662	Carassius auratus	-RKKRVPYTKVQLKELEREYATNKFITKDKRRRISAHTNLTERQVTIWFQNRRM------	HoxA13b protein (Fragment) (Hox)
DH_1661	Carassius auratus	-RKKRVPYTKVQLKELEREYATNKFITKDKRRRISAHTNLTERQVTIWFQNAG-------	HoxA13b protein (Fragment) (Hox)
DH_1531	Megalobrama amblycephala	GRKKRVPYTKVQLKELEREYATNKFITKDKRRRISAHTNLTERQVTIWF-----------	Homeodomain protein (Fragment) (HoxA13b)
DH_454	Danio rerio	GRKKRVPYTKVQLKELEREYATNKFITKDKRRRISAHTNLTERQVTIWFQNRRVKEKKVV	Homeobox protein Hox-A13b (hoxa13b)
DH_4305	Danio rerio	GRKKRVPYTKVQLKELEREYATNKFITKDKRRRISAHTNLTERQVTIWFQNRRVKEKKVV	Hoxa13b protein (hoxa13b)
DH_1631	Xenopus laevis	GRKKRVPYTKVQLKELEREYTTNKFITKDKRRRISANTNLSERQVTIWFQNRRVKEKKVV	Putative uncharacterized protein
DH_11776	Gasterosteus aculeatus	GRKKRVPYTKVQLKELEREYNANKFITKDKRRRISAQTNLSERQVTIWFQNRRVKEKKVI	Transcription factor HOXA13 (Fragment). [Source:UniProtKB/TrEMBL;Acc:Q9PWC4]
DH_1660	Carassius auratus	--KEAVPYTKVQLKELEREYNANKFITKDKRRRISAQTNLSERQVTIWFQNRRM------	HoxA13a protein (Fragment) (Hox)
DH_453	Fugu rubripes	GRKKRVPYTKVQLKELEREYAANKFITKDKRRRISAQTNLSERQVTIWFQNRRVKEKKVV	Homeobox protein Hox-A13a (hoxa13a)
DH_6544	Tetraodon nigroviridis	GRKKRVPYTKVQLKELEREYAANKFITKDKRRRISAQTNLSERQVTIWFQNRRVKEKKVV	Chromosome 21 SCAF14577, whole genome shotgun sequence (GSTENG00017476001)
DH_3283	Salmo salar	GRKKRVPYTKVQLKELEREYAANKFITKDKRRRISAQTNLSERQVTIWFQNRRVKEKKVV	Homeobox protein HoxA13aa (HoxA13aa)
DH_3292	Salmo salar	GRKKRVPYTKVQLKELEREYAANKFITKDKRRRISAQTNLSERQVTIWFQNRRVKEKKVV	Homeobox protein HoxA13ab (HoxA13ab)
DH_2147	Haplochromis burtoni	GRKKRVPYTKVQLKELEREYAANKFITKDKRRRISAQTNLSERQVTIWFQNRRVKEKKIV	Hoxa13a (hoxa13a)
DH_6181	Oryzias latipes	GRKKRVPYTKVQLKELEREYAANKFITKDKRRRISAQTNLSERQVTIWFQNRRVKEKKIV	HoxA13a (hoxA13a)
DH_2131	Haplochromis burtoni	GRKKRVPYTKVQLKELEREYATNKFITKDKRRRISAQTNLTERQVTIWFQNRRVKEKKIV	Hoxa13b (hoxa13b)
DH_6851	Oreochromis niloticus	-------------KELEREYATNKFITKDKRRRISAQTNLTERQVTIWFQNRRVKEKKIV	Hox protein (Fragment)
DH_6176	Oryzias latipes	GRKKRVPYTKVQLKELEREYASNKFITKDKRRRISAQTNLTERQVTIWFQNRRVKEKKIV	HoxA13b (hoxA13b)
DH_6387	Tetraodon nigroviridis	GRKKRVPYTKVQLKELEREYAANKFITKDKRRRISVQTNLTERQVTIWFQNRRVKEKKIV	Chromosome 8 SCAF15044, whole genome shotgun sequence. (Fragment) (GSTENG00034119001)
DH_3301	Salmo salar	GRKKRVPYTKVQLKELEREYAANKFITKDKRRRISDQSNLTERQVTIWFQNRRVKEKKVV	Homeobox protein HoxA13b (HoxA13b)
DH_3340	Salmo salar	GRKKRVPYTKVQLKELEREYAANKFITKDKRRRISDQSNLTERQVTIWFQNRRVKEKKVV	Homeobox protein HoxA13b (HoxA13b)
DH_455	Fugu rubripes	GRKKRVPYTKVQLKELEREYAANKFITKDKRRRISAQTNLTERQVTIWFQNRRVKEKKVV	Homeobox protein Hox-A13b (hoxa13b)
DH_11772	Gasterosteus aculeatus	GRKKRVPYTKVQLKELEREYSANKFITKDKRRRISAQTNLTERQVTIWFQNRRVKEKKVV	Homeobox protein Hox-A13 (Hox-1J) [Source:UniProtKB/Swiss-Prot;Acc:P31271]
DH_604	Heterodontus francisci	GRKKRVPYTKTQLKELEREYATNKFITKEKRRRISTATNLTERQVTIWFQNRRVKEKKVV	Homeobox protein Hox-D13 (HOXD13)
DH_1303	Raja erinacea	GRKKRVPYTKTQLKELEREYATNKFITKEKRRRISTATNLTERQVTIWFQNRRVKEKKVV	Hoxd13
DH_4814	Scyliorhinus canicula	GRKKRVPYTKTQLKELEREYATNKFITKEKRRRISTTTNLSERQVTIWFQ----------	Hoxd13 (Fragment)
DH_651	Danio rerio	GRKKRVPYTKFQLKELEREYNTTKFITKENRRRIASSTNLSERQVTIWFQNRRVKDKKRP	Homeobox protein Hox-D13a (hoxd13a)
DH_2559	Danio rerio	GRKKRVPYTKFQLKELEREYNTTKFITKENRRRIASSTNLSERQVTIWFQNRRVKDKKRP	Homeo box D13a (hoxd13a)
DH_2178	Danio rerio	GRKKRVPYTKFQLKELEREYNTTKFITKENRRRIASSTNLSERQVTIWFQNRRVKDKKRP	Hoxd13a protein (hoxd13a)
DH_1560	Megalobrama amblycephala	GRKKRVPYTYFQLKELEREYNTTKFITKEFRRRIASSTNLAERQVTIWFQCRPVK-----	Homeodomain protein (Fragment) (HoxD13a)
DH_3269	Salmo salar	GRKKRVPYTKLQLKELEREYTICKFITKEKRQRIASSTILSERQVTIWFQNRRVKNKKLI	Homeobox protein HoxD13aa (HoxD13aa)
DH_602	Carollia perspicillata	GRKKRVPYTKLQLKELENEYAINKFINKDKRRRISAATNLSERQVTIWFQNRRVKDKKIV	Homeobox protein Hox-D13 (HOXD13)
DH_605	Homo sapiens	GRKKRVPYTKLQLKELENEYAINKFINKDKRRRISAATNLSERQVTIWFQNRRVKDKKIV	Homeobox protein Hox-D13 (HOXD13)
DH_606	Mus musculus	GRKKRVPYTKLQLKELENEYAINKFINKDKRRRISAATNLSERQVTIWFQNRRVKDKKIV	Homeobox protein Hox-D13 (Hoxd13)

```
DH_603   Gallus gallus              GRKKRVPYTKLQLKELENEYAINKFINKDKRRRISAATNLSERQVTIWFQNRRVKDKKIV Homeobox protein Hox-D13 (HOXD13)
DH_5865  Trionyx sinensis           GRKKRVPYTKLQLKELENEYAINKFINKDKRRRISAATNLSERQVTIWFQNRRVKDKKIV Homeobox protein HoxD13 (Fragment) (PsHoxD13)
DH_7329  Xenopus laevis             GRKKRVPYTKLQLKELENEYAMNKFINKDKRRRISATTNLSERQVTIWFQNRRVKDKKIV Hoxd13 protein (Hoxd13)
DH_8031  Xenopus laevis             GRKKRVPYTKLQLKELENEYAMNKFINKDKRRRISATTNLSERQVTIWFQNRRVKDKKIV Hoxd13 (hoxd13)
DH_7681  Pleurodeles waltlii        GRKKRVPYTKVQLKELEGEYALSKFINKDKRRRISASTSLSERQVTIWFQNRRVKDKKII Transcription factor HoxD13
DH_2136  Haplochromis burtoni       GRKKRVPYTKIQLKELEKEYAASKFITKDKRRRISAATNLSERQVTIWFQNRRVKEKKFV Hoxc13a (hoxc13a)
DH_6153  Oryzias latipes            GRKKRVPYTKIQLKELEKEYAASKFITKDKRRRISAATNLSERQVTIWFQNRRVKEKKFV HoxC13a (hoxC13a)
DH_11820 Gasterosteus aculeatus     GRKKRVPYTKIQLKELEKEYAASKFITKDKRRRISAGTNLSERQVTIWFQNRRVKEKKFV Homeobox protein Hox-C13 (Hox-3G) [Source:UniProtKB/Swiss-Prot;Acc:P31276]
DH_1470  Danio rerio                GRKKRVPYTKIQLKELEKEYAASKFITKDKRRRISATTNLSERQVTIWFQNRRVKEKKFV Homeo box C13a (hoxc13a)
DH_1709  Danio rerio                GRKKRVPYTKIQLKELEKEYAASKFITKDKRRRISATTNLSERQVTIWFQNRRVKEKKFV Hoxc13a protein (hoxc13a)
DH_582   Danio rerio                GRKKRVPYTKIQLKELEKEYAASKFITKDKRRRISATTNLSERQVTIWFQNRRVKEKKFV Homeobox protein Hox-C13a (hoxc13a)
DH_3252  Salmo salar                GRKKRVPYTKIQLKELEKEYAASKFITKDKRRRISATTNLSERQVTIWFQNRRVKEKKFV Homeobox protein HoxC13ba (HoxC13ba)
DH_584   Danio rerio                GRKKRVPYTKIQLKELEKEYAASKFITKDRRRRISATTSLSERQVTIWFQNRRVKEKKFV Homeobox protein Hox-C13b (hoxc13b)
DH_1760  Danio rerio                GRKKRVPYTKIQLKELEKEYAASKFITKDRRRRISATTSLSERQVTIWFQNRRVKEKKFV Homeo box C13b (hoxc13b)
DH_3261  Salmo salar                GRKKRVPYTKIQLKELEKEYAASKFITKDKRRRISATSNLSERQVTIWFQNRRVKEKKCV Homeobox protein HoxC13bb (HoxC13bb)
DH_8005  Latimeria menadoensis      GRKKRVPYTKIQLKELEKEYAASKFITKEKRRRISATTNLSERQVTIWFQNRRVKEKKIL HoxC13 (Fragment)
DH_1668  Carassius auratus          -RKKRVPYTKIQLKELEKEYAASKFITKDKRRRISATTNLSERQVTIWFQNRRM------ HoxC13a protein (Fragment) (Hox)
DH_583   Fugu rubripes              GRKKRVPYTKLQLKELEKEYAASKFITKDKRRRISAATNLSERQVTIWFQNRRVKEKKFI Homeobox protein Hox-C13a (hoxc13a)
DH_6448  Tetraodon nigroviridis     GRKKRVPYTKIQLKELEKEYAASKFITKDKRRRISAATNLSERQVTIWFQNRRVKEKKFI Chromosome 9 SCAF14991, whole genome shotgun sequence. (Fragment) (GSTENG00028043001)
DH_3233  Salmo salar                GRKKRVPYTKLQLKELEKEYAASKFITKDKRRRISAGTNLSERQVTIWFQNRRVKEKKFV Homeobox protein HoxC13aa (HoxC13aa)
DH_3358  Salmo salar                GRKKRVPYTKLQLKELEKEYAASKFITKDKRRRISAGTNLSERQVTIWFQNRRVKEKKFV Homeobox protein HoxC13aa (HoxC13aa)
DH_3242  Salmo salar                GRKKRVPYTKLQLKELEKEYAGSKFITKDKRRRISAGTNLSERQVTIWFQNRRVKEKKFV Homeobox protein HoxC13ab (HoxC13ab)
DH_6832  Oreochromis niloticus      GRKKRVPYTKLQLKELEKEYAASKFITKDKRRRISAATNLSERQVTIWFQNRRVKEKKFV Hox protein (Fragment)
DH_541   Homo sapiens               GRKKRVPYTKVQLKELEKEYAASKFITKEKRRRISATTNLSERQVTIWFQNRRVKEKKVV Homeobox protein Hox-C13 (HOXC13)
DH_542   Mus musculus               GRKKRVPYTKVQLKELEKEYAASKFITKEKRRRISATTNLSERQVTIWFQNRRVKEKKVV Homeobox protein Hox-C13 (Hoxc13)
DH_1706  Bos taurus                 GRKKRVPYTKVQLKELEKEYAASKFITKEKRRRISATTNLSERQVTIWFQNRRVKEKKVV HOXC13 protein (HOXC13)
DH_4284  Capra hircus               GRKKRVPYTKVQLKELEKEYAASKFITKEKRRRISATTNLSERQVTIWFQNRRVKEKKVV Homeobox C13 (Fragment) (Hoxc13)
DH_8062  Ovis aries                 GRKKRVPYTKVQLKELEKEYAASKFITKEKRRRISATTNLSERQVTIWFQNRRVKEKKVV Hoxc13 (Fragment) (Hoxc13)
DH_8268  Homo sapiens               GRKKRVPYTKVQLKELEKEYAASKFITKEKRRRISATTNLSERQVTIWFQNRRVKEKKVV Putative uncharacterized protein NUP98/HOXC13 (Fragment) (NUP98/HOXC13)
DH_8293  Pleurodeles waltlii        GRKKRVPYTKAQLKELEKEYAASKFITKEKRRRISAATGLSERQVTIWFQNRRVKEKKVV Transcription factor HoxC13
DH_2824  Lampetra japonica          GRKKRVPYTKVQLKELEREYAANKFITKDKRRKISAAISLSERQVTIWFQNRRVKEKKVV Hox13 homolog (Fragment) (LjHox13-alpha)
DH_11754 Gasterosteus aculeatus     GRKKRIPYTKLQLKELEKEYAANKFITKEKRRKISAATNLSERQITIWFQNRRVKEKKFM Homeobox protein Hox-B13 [Source:UniProtKB/Swiss-Prot;Acc:Q92826]
DH_11833 Gasterosteus aculeatus     GRKKRIPYTKLQLKELEKEYAANKFITKEKRRKISAATNLSERQITIWFQNRRVKEKKFM Homeobox protein Hox-B13 [Source:UniProtKB/Swiss-Prot;Acc:Q92826]
DH_7715  Sphoeroides nephelus       GRKKRIPYTKIQLKELEKEYAANKFITKDKRRKISAATNLSERQITIWFQNRRVKEKKFG HoxB13a
DH_6841  Oreochromis niloticus      -------------KELEKEYAANKFITKDKRRKISADTNLSERHITIWLQKRRVKDKKFL Hox protein (Fragment)
DH_6509  Tetraodon nigroviridis     GRKKRIPYTKVQLKELEKEYAANKFITKDKRRKISAATNLSERQITIWFQNRRVKEKKFV Chromosome undetermined SCAF14653, whole genome shotgun sequence. (Fragment) (GSTENG000204
DH_531   Fugu rubripes              GRKKRIPYTKVQLKELEKEYAANKFITKDKRRKISAATNLSERQITIWFQNRRVKEKKFV Homeobox protein Hox-B13a (hoxb13a)
DH_6167  Oryzias latipes            GRKKRIPYTKIQLKELEKEYAANKFITKDKRRKISAVTNLSERQITIWFQNRRVKEKKFV HoxB13a (hoxB13a)
DH_2126  Haplochromis burtoni       GRKKRIPYTKVQLKELEKEYAANKFITKDKRRKISAVTNLSERQITIWFQNRRVKEKKFV Hoxb13a (hoxb13a)
DH_530   Danio rerio                GRKKRIPYTKVQLKELEKEYAANKFITKDKRRKISAVTNLSERQITIWFQNRRVKEKKFI Homeobox protein Hox-B13a (hoxb13a)
DH_3306  Salmo salar                GRKKRIPYTKVQLKELEKEYAANKFITKDKRRKISAGTNLSERQITIWFQNRRVKEKKFV Homeobox protein HoxB13aa (HoxB13aa)
DH_3344  Salmo salar                GRKKRIPYTKVQLKELEKEYAANKFITKDKRRKISAGTNLSERQITIWFQNRRVKEKKFV Homeobox protein HoxB13aa (HoxB13aa)
DH_3315  Salmo salar                GRKKRIPYTKVQLKELEKEYAANKFITKDKRRKISTGTNLSERQITIWFQNRRVKEKKFV Homeobox protein HoxB13ab (HoxB13ab)
DH_8012  Latimeria menadoensis      GRKKRIPYTKVQLKELEKEYATNKFITKDKRRKISAATNLSERQITIWFQNRRVKEEKVV HoxB13 (Fragment)
DH_1663  Carassius auratus          -RKKRVPYTKVQLKELEKEYAANKFITKDKRRKISAVTNLSERQITIWFQNRRM------ HoxB13b protein (Fragment) (Hox)
DH_1667  Carassius auratus          --KERVPYTKVQLKELEKEYAANKFITKDKRRKISAVTNLSERQITIWFQNRRM------ HoxB13b protein (Fragment) (Hox)
DH_1666  Carassius auratus          -RKKRVPYTKVQLKELEKEYAANKFITKDKRRKISAVTNLSERQITIWFQYRRM------ HoxB13b protein (Fragment) (Hox)
DH_1664  Carassius auratus          -RKKRVPYTKVQLKELEKEYAANKFITKDKRRKISAVTNLSERQITIWFQNRG------- HoxB13b protein (Fragment) (Hox)
DH_1665  Carassius auratus          -RKKRVPYTKVQLKELEKEYAANKFITKDKRRKISAVTNLSERQITIWSKTAG------- HoxB13b protein (Fragment) (Hox)
DH_456   Homo sapiens               GRKKRIPYSKGQLRELEREYAANKFITKDKRRKISAATSLSERQITIWFQNRRVKEKKVL Homeobox protein Hox-B13 (HOXB13)
DH_2828  Homo sapiens               GRKKRIPYSKGQLRELEREYAANKFITKDKRRKISAATSLSERQITIWFQNRRVKEKKVL cDNA, FLJ93776, highly similar to Homo sapiens homeo box B13 (HOXB13), mRNA (Homeobox B13)
DH_457   Mus musculus               GRKKRIPYSKGQLRELEREYAANKFITKDKRRKISAATSLSERQITIWFQNRRVKEKKVL Homeobox protein Hox-B13 (Hoxb13)
DH_1416  Mus musculus               GRKKRIPYSKGQLRELEREYAANKFITKDKRRKISAATSLSERQITIWFQNRRVKEKKVL Homeo box B13 (Hoxb13)
DH_6323  Homo sapiens               GRKKRTPYSKGQLRELEREYAANKFITKDKRRKISAATSLSERQITIWFQNRRVKEKKVL PSGD protein (HOXB13)
DH_9109  Ambystoma mexicanum        DRKKRVPYSKGQLRELEKEYASSKFITKDRRRQIATATNLSERQITIWFQNRRVKEKKVF Homeodomain protein Hoxb13 (Fragment) (Hoxb13)
DH_2825  Lampetra japonica          ARKRRVPYSKAQLRELEAEFGASRFVSRERRRGVAASTQLEERQVTIWFQNRRVKEKKVA Putative Hox13 homolog (Fragment) (LjHox13-beta)
DH_11607 Branchiostoma floridae     -RPKRRPYSKYQLNELENEYVQNQYISRDKRLQLSQKLNLTERQVKIWFQNRRIKQKKLD e_gw.260.37.1|Brafl1
DH_11643 Branchiostoma floridae     -RPKRRPYSKYQLNELENEYVQNQYISRDKRLQLSQKLNLTERQVKIWFQNRRIKQKKLD e_gw.260.36.1|Brafl1
DH_6280  Ciona intestinalis         SRKKRQPYSKTQISSLEREYKANNFITRQKRENIARDLKLSDRQVKIWFQNRRVKDKKIK Transcription factor protein (Ci-Hox13)
DH_7970  Diplosoma listerianum      SRKKRQPYSKTQINNLEQEYQNSNFITRQKRELIARDLKLTDRQVKIWFQNRRVKDKKIK Hox-POST1 (Fragment) (Hoxpost1)
DH_6671  Oikopleura dioica          IRKKRQPYSKQQTRQLEQEYAQNKFITRQKREQISRDLSLTDRQVKIWFQNRRVKEKKER Hox13 (Fragment) (Hox13)
```

Save alignment [fasta format] - Save complete sequences [fasta format]

PG14

ID	organism	sequence (positions 10, 20, 30, 40, 50, 60)	description
DH_11529	Latimeria menadoensis	QRKKRVPYSKHQITELERAFEENRFLTPEIRQNISVKLGLTERQVKIWFQNQRQKEKKLL	HoxA14
DH_2826	Lampetra japonica	PRKKRVPYSKQQISELERAFDENRFLTPELRLSISHRLSLTERQVKIWFQNQRQKEKKLM	Hox14 homolog (Fragment) (LjHox14-alpha)
DH_11530	Heterodontus francisci	QRKKRVPYSKQQIAELEMAYENNRFLTPEVRLNISFKLGLTERQVKIWFQNQRQKEKKLL	HoxD14

Save alignment [fasta format] - Save complete sequences [fasta format]

CDX

ID	organism	10 20 30 40 50 60	description
DH_12061	Capitella sp. I Grassle and Grassle, 1976	KDKYRIVYSEYQKVELEKEYLYS-KYITIQRKAELSRSIGLSERQVKIWFQNRRAKERKQK	e_gw1.444.23.1|Capca1
DH_5850	Capitella sp. I Grassle and Grassle, 1976	KDKYRIVYSEYQKVELEKEYLYS-KYITIQRKAELSRSIGLSERQVKIWFQNRRAKERKQK	Caudal protein (Cdx)
DH_12500	Helobdella robusta	KDKYRIVYSELQKVELEKEFLYN-QYVTIQRKSELAAFIGLSDRQVKIWFQNRR-------	gw2.39.154.1|Helro1
DH_7279	Tubifex tubifex	---YRVVYSELQKVELEKEFLYN-QYITIQRKAELATSIGLSDLQVKIWFQNRRAKERKTK	Caudal (Fragment) (cdx)
DH_6698	Perionyx excavatus	KDKYRVVYSEHQKVELEKEFLYN-PYITIQRKAELASIIGLSDRQVKIWFQNRR-------	Homeodomain transcription factor Cdx (Fragment)
DH_2756	Flaccisagitta enflata	KDKYRVVYSDHQRIELEKEYLQS-PYITIKRKAELAQTLDLSERQVKIWFQNRRAKERKQF	Caudal (Fragment)
DH_11623	Branchiostoma floridae	KDKYRVVYSDHQRLELEKEFYSN-KYITIKRKVQLANELGLSERQVKIWFQNRRAKQRKMA	e_gw.24.290.1|Brafl1
DH_11645	Branchiostoma floridae	KDKYRVVYSDHQRLELEKEFYSN-KYITIKRKVQLANELGLSERQVKIWFQNRRAKQRKMA	e_gw.24.291.1|Brafl1
DH_9439	Branchiostoma floridae	KDKYRVVYSDHQRLELEKEFYSN-KYITIKRKVQLANELGLSERQVKIWFQNRRAKQRKMA	Homeobox protein AmphiCdx (Fragment)
DH_7030	Oikopleura dioica	RDKYRVVYSNYQRVELEKEYNFN-KYITIRRKVELSKMLDLSERQVKIWFQNRRAKERKMV	Cdx1 (Cdx1)
DH_7029	Oikopleura dioica	REKYRVVYTNFQRLELEKEYRFN-RYITISKKAELAKQLQLSERQIKIWFQNR--------	Cdx2 (Fragment) (Cdx2)
DH_7028	Oikopleura dioica	KDKYRVVYSDTQRLELEKEFAFN-KYITIQRKGELSQQLNLTERQVKIWFQNRRAKERKIS	Cdx3 (Fragment) (Cdx3)
DH_4667	Tribolium castaneum	KDKYRVVYTDLQRIELEKEFTFVSKYITIKRKSELAENLGLSERQIKIWFQNRRAKERKQN	Caudal protein
DH_899	Caenorhabditis elegans	ADKYRMVYSDYQRLELEKEFHTS-PFITSDRKSQLSTMLSLTERQIKIWFQNRRAKDRRDK	Homeobox protein pal-1 (pal-1)
DH_7323	Caenorhabditis elegans	ADKYRMVYSDYQRLELEKEFHTS-PFITSDRKSQLSTMLSLTERQIKIWFQNRRAKDRRDK	Protein C38D4.6b, confirmed by transcript evidence (pal-1)
DH_2393	Caenorhabditis briggsae	ADKYRMVYSDYQRLELEKEFHTS-AFITSDRKSQLSTMLSLTERQIKIWFQNRRAKDRRDK	CBR-PAL-1 protein (Cbr-pal-1)
DH_8078	Pristionchus pacificus	QDKYRMVYSDYIRLELEKEFHMN-QFINADRKADLATKLNLTEAQIKIWFQNRRAKKRRDE	Posterior alae protein
DH_9137	Discocelis tigrina	CDKYRQVYTEQQKLELEKEFLTQ-KYVNARRKSEMARALQLTERQVKIWFQNRRAKERKLF	Caudal protein (Fragment) (Cdx)
DH_11968	Gasterosteus aculeatus	KDKYRVVYTDQQRLELEKEFQCN-QYITMRRKTELSMALSLSERQVKIWFQNRRAKERKIN	groupIV
DH_4380	Convolutriloba longifissura	KDKYRVVYTDRQRAELENEFRSA-QYITIRRKSELAMQVGLSERQVKIWFQNRRAKERKVS	Cdx
DH_7961	Symsagittifera roscoffensis	KDKYRVVYTDRQRAELENEFRSA-QYITIRRKSELAMQVGLSERQVKIWFQNRRAKERKVS	Parahox class caudal-like protein Srcdx (Fragment) (Cdx)
DH_2478	Endeis spinosa	REKYRVVYSDHQRLELEKEFHYN-RYITIRRKSEVASFLGLSERQIKIWFQNRRAKERKQL	Homeobox protein caudal (Fragment) (Cad)
DH_7363	Euscorpius flavicaudis	--------TDHQRLELEKEFHYS-RYITIRRKSELASSLGLSERQVKIWFQNRRAKERKQA	Caudal (Fragment)
DH_11423	Daphnia pulex	KDKYRVVYTDHQRLELEKEFHYS-RYITIRRKSELASVLGLSERQVKIWFQNRRAKERKQV	gw1.7.441.1|Dappu1
DH_11521	Daphnia pulex	KDKYRVVYTDHQRLELEKEFHYS-RYITIRRKSELASVLGLSERQVKIWFQNRRAKERKQV	YAS_pHox_Cad|Dappu1
DH_7759	Artemia sanfranciscana	KDKYRVVYTDHQRLELEKEFHYN-KYITIRRKSELATVLGLSERQVKIWFQNRRAKDRKQM	Homeodomain transcription factor (cad)
DH_2216	Danio rerio	KEKYRVVYTDHQRLELEKEFHFN-RYITIRRKSELAVNLGLSERQVKIWFQNRRAKERKLI	Cdx4 protein (Caudal type homeo box transcription factor 4) (cdx4)
DH_7414	Danio rerio	KEKYRVVYTDHQRLELEKEFHFN-RYITIRRKSELAVNLGLSERQVKIWFQNRRAKERKLI	Cdx4 protein (Putative uncharacterized protein) (cdx4)
DH_2739	Xenopus tropicalis	KEKYRVVYTDHQRLELEKEFHFN-RYITIRRKSELAVNLGLSERQVKIWFQNRRAKERKLI	LOC100145392 protein (LOC100145392)
DH_8689	Danio rerio	KEKYRVVYTDHQRLELEKEFHFN-RYITIRRKSELAVNLGLSERQVKIWFQNRRAKERKLI	Zf-cad1 protein (cdx4)
DH_8707	Cyprinus carpio	KEKYRVVYTDHQRLELEKEFHFN-RYITIRRKSELAVNLGLSERQVKIWFQNRRAKERKLI	Cdx1 protein (cdx1)
DH_7673	Oncorhynchus mykiss	KEKYRVVYTDHQRLELEKEFHFN-RYITIRRKSELAGNLGLSERQVKIWFQNRRAKERKLI	Caudal-type homeobox protein 1 (Cdx1)
DH_11889	Gasterosteus aculeatus	KEKYRVVYTDHQRLELEKEFHFN-RYITIRRKSELALNLGLSERQVKIWFQNRRAKERKLI	Homeobox protein CDX-4 (Caudal-type homeobox protein 4) [Source:UniProtKB/Swiss-Prot;Acc:O14627]
DH_78	Homo sapiens	KEKYRVVYTDHQRLELEKEFHCN-RYITIQRKSELAVNLGLSERQVKIWFQNRRAKERKMI	Homeobox protein CDX-4 (CDX4)
DH_1305	Homo sapiens	KEKYRVVYTDHQRLELEKEFHCN-RYITIQRKSELAVNLGLSERQVKIWFQNRRAKERKMI	Caudal type homeobox 4 (CDX4)
DH_79	Mus musculus	KEKYRVVYTDHQRLELEKEFHCN-RYITIRRKSELAVNLGLSERQVKIWFQNRRAKERKMI	Homeobox protein CDX-4 (Cdx4)
DH_7788	Mus musculus	KEKYRVVYTDHQRLELEKEFHCN-RYITIRRKSELAVNLGLSERQVKIWFQNRRAKERKMI	Caudal type homeo box 4 (Cdx4)
DH_6123	Mus musculus	KEKYRVVYTDHQRLELEKEFHCN-RYITIRRKSELAVNLGLSERQVKIWFQNRRAKERKMI	Putative uncharacterized protein (Cdx4)
DH_272	Gallus gallus	KEKYRVVYTDHQRLELEKEFHCN-RYITIRRKSELAVNLGLSERQVKSWFQNRRAKERKII	Homeobox protein CHOX-CAD2 (Fragment) (CHOX-CAD2)
DH_8755	Gallus gallus	KEKYRVVYTDHQRLELEKEFHCN-RYITIRRKSELAANLGLSERQVKIWFQNRRAKERKII	Caudal-type homeobox protein CDXB (CDXB)
DH_5240	Aedes aegypti	KDKYRVVYTDQQRLELEKEFHYT-RYITIRRKSELAQSLQLSERQVKIWFQNRRAKDRKQK	Homeobox protein cdx (AAEL014557)
DH_2629	Culex quinquefasciatus	KDKYRVVYTDQQRLELEKEFHYT-RYITIRRKAELAQSLQLSERQVKIWFQNRRAKDRKQK	Homeobox protein cdx (CpipJ_CPIJ004813)
DH_9600	Anopheles gambiae	KDKYRVVYTDQQRLELEKEFHYT-RYITIRRKAELAQNLQLSERQVKIWFQNRRAKDRKQK	Caudal protein homolog (AGAP009646-PA) (cad)
DH_66	Drosophila melanogaster	KDKYRVVYTDFQRLELEKEYCTS-RYITIRRKSELAQTLSLSERQVKIWFQNRRAKERKQN	Homeotic protein caudal (cad)
DH_1768	Drosophila melanogaster	KDKYRVVYTDFQRLELEKEYCTS-RYITIRRKSELAQTLSLSERQVKIWFQNRRAKERKQN	CG1759-PB, isoform B (cad)
DH_4088	Drosophila yakuba	KDKYRVVYTDFQRLELEKEYCTS-RYITIRRKSELAQTLSLSERQVKIWFQNRRAKERKQN	GE12346 (GE12346)
DH_3609	Drosophila sechellia	KDKYRVVYTDFQRLELEKEYCTS-RYITIRRKSELAQTLSLSERQVKIWFQNRRAKERKQN	GM23369 (GM23369)
DH_4171	Drosophila simulans	KDKYRVVYTDFQRLELEKEYCTS-RYITIRRKSELAQTLSLSERQVKIWFQNRRAKERKQN	GD24280 (GD24280)
DH_3124	Drosophila erecta	KDKYRVVYTDFQRLELEKEYCTS-RYITIRRKSELAQTLSLSERQVKIWFQNRRAKERKQN	GG21252 (GG21252)
DH_3046	Drosophila ananassae	KDKYRVVYTDFQRLELEKEYCTS-RYITIRRKSELAQTLSLSERQVKIWFQNRRAKERKQN	GF15355 (GF15355)
DH_3441	Drosophila persimilis	KDKYRVVYTDFQRLELEKEYCTS-RYITIRRKSELAQTLSLSERQVKIWFQNRRAKERKQN	GL18519 (GL18519)
DH_5792	Drosophila pseudoobscura pseudoobscura	KDKYRVVYTDFQRLELEKEYCTS-RYITIRRKSELAQTLSLSERQVKIWFQNRRAKERKQN	GA14567 (GA14567)
DH_3979	Drosophila willistoni	KDKYRVVYTDFQRLELEKEYCTS-RYITIRRKSELAQTLALSERQVKIWFQNRRAKERKQN	GK14914 (GK14914)
DH_3776	Drosophila mojavensis	KDKYRVVYTDFQRLELEKEYCTS-RYITIRRKSELAQSLSLSERQVKIWFQNRRAKERKQN	GI17922 (GI17922)
DH_3879	Drosophila virilis	KDKYRVVYTDFQRLELEKEYCTS-RYITIRRKSELAQSLSLSERQVKIWFQNRRAKERKQN	GJ17694 (GJ17694)
DH_3668	Drosophila grimshawi	KDKYRVVYTDFQRLELEKEYCTS-RYITIRRKSELAQSLSLSERQVKIWFQNRRAKERKQN	GH11619 (GH11619)
DH_2497	Lonchoptera lutea	KDKYRVVYTDYQRLELEKEYCTS-RYITIRRKTELAQSLQLSERQVKIWFQNRRAKERKQN	Caudal (cad)
DH_2500	Megaselia abdita	KDKYRVVYSDFQRLELEKEYCTA-RYITIRRKSELATSLQLSERQVKIWFQNRRAKERKQQ	Caudal' (cad)
DH_2502	Megaselia abdita	KDKYRVVYSDFQRLELEKEYCTA-RYITIRRKSELATSLQLSERQVKIWFQNRRAKERKQQ	Caudal' (cad)
DH_2501	Megaselia abdita	KDKYRVVYSDFQRLELEKEYCTA-RYITIRRKSELATSLQLSERQVKIWFQNRRAKERKQQ	Caudal (cad)
DH_2498	Empis livida	KDKYRVVYTDFQRLELEKEYHTS-RYITIRRKTELAQGLQLSERQVKIWFQNRRAKERKQT	Caudal (cad)
DH_2499	Haematopota pluvialis	KDKYRVVYTDFQRLELEKEYHTS-RYITIRRKTELAQSLQLSERQVKIWFQNRRAKERKQT	Caudal (Fragment) (cad)

DH_7280	Gryllus bimaculatus	KDKYRVVYSDHQRLELEKEFHYS-RYITIRRKAELAASLGLSERQVKIWFQNRRAKERKQV	Caudal (Fragment) (cad)
DH_5305	Bombyx mori	KDKYRVVYSDHQRLELEKEFHYS-RYITIRRKAELAVSLGLSERQVKIWFQNRRAKERKQV	Cad protein (cad homologue)
DH_7386	Strigamia maritima	KDKYRVVYSDIQRLELEKEFHYS-RYITIRRKAELAQLLGLSERQVKIWFQNRRAKERKQV	Caudal (Fragment)
DH_4666	Tribolium castaneum	KDKYRVVYTDHQRVELEKEFYYS-RYITIRRKAELANSLGLSERQVKIWFQNRRAKERKQV	Caudal protein
DH_7239	Diplosoma listerianum	KDKYRVVYSDHQRLELEKEFRFS-RYITIRRRSELAEQLCLSERQVKIWFQNRRAKERKAS	ParaHox CDX (Fragment)
DH_7972	Diplosoma listerianum	KDKYRVVYSDHQRLELEKEFRFS-RYITIRRRSELAEQLCLSERQVKIWFQNRRAKERKAS	Caudal ParaHox-CDX (cdx)
DH_6299	Ciona intestinalis	KDKYRVVYSDHQRLELEKEFRFS-RYITIRRKAELAGLLCLSERQIKIWFQNRRAKERKAT	Transcription factor protein (Ci-Cdx)
DH_9471	Halocynthia roretzi	KDKYRVVYSDHQRLELEKEFHFS-RYITIRRKSELAMQLSLSERQIKIWFQNRRAKERKIS	Caudal homeoprotein (Hrcad)
DH_9278	Herdmania curvata	KDKYRVVYSDHQRLELEKEFRYS-RYITIRRKSELASQLHLSERQVKIWFQNRRAKERKNN	Cdx
DH_8970	Procambarus clarkii	KDKYRVVYSDHQRLELEKEFHYS-RYITIRRKSELASMLGLSERQVKIW------------	Caudal protein (Fragment)
DH_12605	Strongylocentrotus purpuratus	KDKYRVVYTDHQRLELEKEFHYS-RYITIRRKSELALALGLSERQVKIWFQNRRAKERKMA	GLEAN3_24715|Sp-Cdx
DH_5048	Polypterus senegalus	KDKYRVVYTDHQRLELEKEFHYS-RYITIRRKSEIAMSLGLSERQVKIWFQNRRAKERKLA	Cdx2 (Cdx2)
DH_76	Mesocricetus auratus	KDKYRVVYTDHQRLELEKEFHYS-RYITIRRKAELAATLGLSERQVKIWFQNRRAKERKIN	Homeobox protein CDX-2 (CDX2)
DH_9128	Rattus norvegicus	KDKYRVVYTDHQRLELEKEFHYS-RYITIRRKAELAATLGLSERQVKIWFQNRRAKERKIN	CDX2 protein (Cdx2)
DH_75	Homo sapiens	KDKYRVVYTDHQRLELEKEFHYS-RYITIRRKAELAATLGLSERQVKIWFQNRRAKERKIN	Homeobox protein CDX-2 (CDX2)
DH_7173	Mustela vison	-DKYRVVYTDHQRLELEKEFHYS-RYITIRRKAELAATLGLSERQVKIWF-----------	Caudal-type homeobox transcription factor 2 (Fragment)
DH_1937	Sus scrofa	KDKYRVVYTDHQRLELEKEFHYS-RYITIRRKAELAATLGLSERQVKIWFQNRRA------	Caudal type homeobox transcription factor 2 (Fragment) (CDX-2)
DH_77	Mus musculus	KDKYRVVYTDHQRLELEKEFHFS-RYITIRRKSELAATLGLSERQVKIWFQNRRAKERKIK	Homeobox protein CDX-2 (Cdx2)
DH_6749	Mus musculus	KDKYRVVYTDHQRLELEKEFHFS-RYITIRRKSELAATLGLSERQVKIWFQNRRAKERKIK	Caudal type homeo box 2 (Cdx2)
DH_8311	Mus musculus	KDKYRVVYTDHQRLELEKEFHFS-RYITIRRKSELAATLGLSERQVKIWFQNRRAKERKIK	Putative uncharacterized protein (Cdx2)
DH_4749	Triops longicaudatus	-----VVYTDHQRLELEKEFHYS-RYITIRRKSELAAMLGLS-------------------	Caudal (Fragment) (cad)
DH_12663	Strongylocentrotus purpuratus	KDKYRVVYTDHQRLELEKEFHYS-RYITIRRKSELALALGLSERQGTLRRPNMDMSIKQEA	GLEAN3_19656|Sp-Cdx-like
DH_1354	Gorilla gorilla gorilla	KDKYRVVYTDHQRLELEKEFHYS-RYITIRRKSELAANLGLTERQ----------------	CDX1 (Fragment) (CDX1)
DH_8932	Schistocerca gregaria	KDKYRVVYSDHQRLELEKEFHYS-RYITIRRKAELAANLGLSERQV---------------	Caudal (Fragment) (cad)
DH_68	Gallus gallus	KDKYRVVYTDHQRLELEKEFHYS-RYITIRRKAELAAALGLTERQVKIWFQNRRAKERKVN	Homeobox protein CDX-1 (CDX1)
DH_271	Gallus gallus	KDKYRVVYTDHQRLELEKEFHYS-RYITIRRKAELAAALGLTERQVKIWFQNRRAKERKVN	Homeobox protein CHOX-CAD (CHOX-CAD1)
DH_7525	Gallus gallus	KDKYRVVYTDHQRLELEKEFHYS-RYITIRRKAELAAALGLTERQVKIWFQNRRAKERKVN	CHox-cad protein (Fragment) (CHox-cad)
DH_73	Xenopus laevis	KDKYRVVYTDHQRLELEKEFHYS-RYITIRRKAELAAALGLTERQVKIWFQNRRAKERKVN	Homeobox protein CDX-1 (cdx1)
DH_1833	Xenopus laevis	KDKYRVVYTDHQRLELEKEFHYS-RYITIRRKAELAAALGLTERQVKIWFQNRRAKERKVN	XCAD2 protein (xCAD2)
DH_4756	Xenopus laevis	KDKYRVVYTDHQRLELEKEFHYS-RYITIRRKAELAAALGLTERQVKIWFQNRRAKERKVN	Xcad 2 protein (Fragment) (Xcad 2)
DH_69	Homo sapiens	KDKYRVVYTDHQRLELEKEFHYS-RYITIRRKSELAANLGLTERQVKIWFQNRRAKERKVN	Homeobox protein CDX-1 (CDX1)
DH_71	Pongo pygmaeus	KDKYRVVYTDHQRLELEKEFHYS-RYITIRRKSELAANLGLTERQVKIWFQNRRAKERKVN	Homeobox protein CDX-1 (CDX1)
DH_1386	Pan paniscus	KDKYRVVYTDHQRLELEKEFHYS-RYITIRRKSELAANLGLTERQVKIWFQNRRAKERKVN	CDX1 (Fragment) (CDX1)
DH_6650	Homo sapiens	KDKYRVVYTDHQRLELEKEFHYS-RYITIRRKSELAANLGLTERQVKIWFQNRRAKERKVN	CDX1 protein (CDX1)
DH_1851	Bos taurus	KDKYRVVYTDHQRLELEKEFHYS-RYITIRRKSELAANLGLTERQVKIWFQNRRAKERKVN	CDX1 protein (CDX1)
DH_70	Mus musculus	KDKYRVVYTDHQRLELEKEFHYS-RYITIRRKSELAANLGLTERQVKIWFQNRRAKERKVN	Homeobox protein CDX-1 (Cdx1)
DH_72	Rattus norvegicus	KDKYRVVYTDHQRLELEKEFHYS-RYITIRRKSELAANLGLTERQVKIWFQNRRAKERKVN	Homeobox protein CDX-1 (Fragment) (Cdx1)
DH_4300	Saccoglossus kowalevskii	KDKYRVVYTDHQRLELEKEFHYS-RYITIRRKAELAHALGLSERQVKIWFQNRRAKERKQN	Caudal homeobox protein
DH_7661	Ptychodera flava	KDKYRVVYTDHQRLELEKEFHYS-RYITIRRKAELALALGLSERQVKIWFQNRRAKERKQN	Transcription factor Cad (Fragment)
DH_1813	Danio rerio	KDKYRVVYTDHQRLELEKEFHYS-RYITIRRKAELATALSLSERQVKIWFQNRRAKERKIN	Cdx1b protein (cdx1b)
DH_74	Xenopus tropicalis	KDKYRVVYTDHQRLELEKEFHYS-RYITIRRKAELAASLALTERQVKIWFQNRRAKERKVN	Homeobox protein CDX-1 (cdx1)
DH_4797	Xenopus tropicalis	KDKYRVVYTDHQRLELEKEFHYS-RYITIRRKAELAASLALTERQVKIWFQNRRA------	Caudal type homeo box transcription factor 1 (Fragment) (cdx1)
DH_80	Lineus sanguineus	PDKYRVVYTDYQRLELEKEFHYS-RYITMNRKAELAKSLDLTERQIKIWFQNRRAKERKIN	Homeobox protein CDX (Fragment) (CDX)
DH_4703	Gallus gallus	KDKYRVVYTDHQRLELEKEFHYS-RYITIRRKAELASSLGLSERQVKIWFQNRRAKERKIN	Homeobox protein (Cdx-C)
DH_6034	Xenopus laevis	KDKYRVVYTDQQRLELEKEFHYS-RYITIRRKAELAVNLGLSERQVKIWFQNRRAKERKIN	Putative uncharacterized protein
DH_8799	Xenopus laevis	KDKYRVVYTDQQRLELEKEFHYS-RYITIRRKAELAVNLGLSETQVKIWFQNRRAKERKIN	Xcad 1 protein (Fragment) (Xcad 1)
DH_5968	Xenopus laevis	KDKYRVVYTDQQRLELEKEFHYS-RYITIRRKAELAVGLGLSERQVKIWFQNRRAKERKIN	MGC130987 protein (cdx2)
DH_8727	Xenopus tropicalis	KDKYRVVYTDQQRLELEKEFHYS-RYITIRRKAELAANLGLSERQVKIWFQNRRAKERKIT	Cad1 (Caudal type homeo box transcription factor 2) (Caudal type homeobox 2) (cdx2)
DH_6040	Xenopus laevis	KEKYRVVYTDHQRLELEKEFHYS-RYITIRRKTELAANLRLSERQVKIWFQNRRAKERKLF	Cdx4 protein (cdx4)
DH_7900	Xenopus laevis	KEKYRVVYTDHQRLELEKEFHYS-RYITIRRKTELAANLRLSERQVKIWFQNRRAKERKLF	Cdx4 protein (cdx4)
DH_8726	Xenopus tropicalis	KEKYRVVYTDHQRLELEKEFHYS-RYITIRRKTELAASLRLSERQVKIWFQNRRAKERKLF	Cad3 (Caudal type homeobox 4) (cdx4)
DH_8791	Xenopus laevis	KEKYTVVYTDHQRLELEKEFHYS-RYITIRRKTELAANLRLSERQVKIWFQNRRAKEGAIQ	Xcad-3 protein (cdx4)
DH_8953	Phascolion strombus	--KYRVVYTDHQRLELEKEFHYS-RYITIRRKAELAQNLSLSERQVKI-------------	Homeodomain transcription factor Cdx (Fragment) (Cdx)
DH_8074	Achaearanea tepidariorum	KDKYRIVYTDHQRLELEKEFHYS-RYITIRRKVELAASLNLSERQIKIWFQNRRAKERRQA	Caudal (At.cad)
DH_12219	Lottia gigantea	KDKYRNVYTDHQRLELEKEFHYS-KFITIRRKTELSAKLNLSQRQVKIWFQNRRAKERKQN	gw1.85.148.1|Lotgi1
DH_6063	Platynereis dumerilii	KDKYRVVYTDHQRLELEKEFHYS-RYTTIRRKAELAQTLNLSERQVKIWFQNRRAKERKQN	Caudal (cad)
DH_7964	Nereis virens	KDKYRVVYTDHQRLELEKEFHYS-RYITIRRKAELAQNLNLSERQVKIWFQNRRAKERKQN	Caudal parahox protein Nvcdx (Fragment) (Cdx)
DH_8283	Danio rerio	KDKYRVVYSDVQRLELEKEFHFS-RYITIRRKAELAGTLNLSERQVKIWFQNRRAKERKMN	Homeodomain protein Cdx1a (Cdx1a protein) (cdx1a)
DH_12251	Lottia gigantea	KDKYRVVYSDHQRLELEKEFHYS-RYITIRRKAEIAAQLQLSERQVKIWFQNRRAKERKQN	estExt_Genewise1.C_sca_850005|Lotgi1
DH_8353	Patella vulgata	KDKYRVAYTDHQRLELEKEFHYS-RYITIRRKAEIAAQLALSERQVKIWFQNRRAKERKQN	Caudal protein (cdx)
DH_5051	Amia calva	KDKYRVVYSDHQRLELEKEFHSN-RYITIRRKADIAATLGLSERQVKIWFQNRRAKERKLD	Caudal homeobox gene 2 (Cdx2)
DH_1276	Nasonia vitripennis	RDKYRVVYTEHQRLELEKEFYSS-RYITIRRKAELASSLALSERQVKIWFQNRRAKDRKQS	Caudal
DH_4803	Nasonia vitripennis	RDKYRVVYTEHQRLELEKEFYSS-RYITIRRKAELASSLALSERQVKIWFQNRRAKDRKQS	Caudal (Fragment)
DH_1274	Nasonia vitripennis	RDKYRVVYTEHQRLELEKEFYSS-RYITIR-------------------------------	Caudal (Fragment)
DH_6463	Tetraodon nigroviridis	KDKYRVVYTDRQRLELEKEFQSN-RYITMRRKADCRWNWASLRDR----------------	Chromosome 1 SCAF14944, whole genome shotgun sequence (GSTENG00026576001)

Save alignment [fasta format] - Save complete sequences [fasta format]

GSX

ID	organism	alignment (10 · 20 · 30 · 40 · 50 · 60)	description
DH_12335	Helobdella robusta	IKRLRTAFSSKQLLGLEKEFLNGGMYLSRLRRIEIAENLKLSEKQ-----VKIWFQNRRVKHKKGS	estExt_Genewise1.C_180241|Helro1
DH_12344	Helobdella robusta	-----TAFSSVQLLQLERCFNDN-MYLSRIRRIEIAASLHLTEKQASNAFVKIWFQNRRVKYKK--	gw2.53.143.1|Helro1
DH_6700	Perionyx excavatus	SKRIRTAFDSRQLLDLEREFGAN-MYLSRLRRIEIANSLSLTEKQ-----VKIWFQNSR-------	Homeodomain transcription factor Gsx (Fragment)
DH_2651	Culex quinquefasciatus	SKRIRTAFTSTQLLELEREFASN-MYLTRLRRIEIATRLRLSEKQ-----VKIWFQNRRVKRKKGD	Intermediate neuroblasts defective protein (CpipJ_CPIJ009458)
DH_8539	Anopheles gambiae	SKRIRTAFTSTQLLELEREFAGN-MYLTRLRRIEIATRLRLSEKQ-----VKIWFQNRRVKRKKGD	Putative hox protein (AGAP001560-PA) (30E5.4)
DH_5330	Aedes aegypti	SKRIRTAFTSTQLLELEREFSSN-MYLTRLRRIEIATRLRLSEKQ-----VKIWFQNRRVKRKKGD	Putative uncharacterized protein (AAEL004130)
DH_7815	Drosophila melanogaster	SKRIRTAFTSTQLLELEREFSHN-AYLSRLRRIEIANRLRLSEKQ-----VKIWFQNRRVKQKKGG	CG11551-PA (RT01026p) (HDC10207) (ind)
DH_8956	Drosophila melanogaster	SKRIRTAFTSTQLLELEREFSHN-AYLSRLRRIEIANRLRLSEKQ-----VKI-------------	Intermediate neuroblasts defective (Fragment) (ind)
DH_4660	Drosophila melanogaster	SKRIRTAFTSTQLLELEREFSHN-AYLSRLRRIEIANRLRLSEKQ-----VKIWFQNRRVKQKKGG	Intermediate neuroblasts defective protein (ind)
DH_4207	Drosophila simulans	SKRIRTAFTSTQLLELEREFSHN-AYLSRLRRIEIANRLRLSEKQ-----VKIWFQNRRVKQKKGG	GD12586 (GD12586)
DH_3541	Drosophila sechellia	SKRIRTAFTSTQLLELEREFSHN-AYLSRLRRIEIANRLRLSEKQ-----VKIWFQNRRVKQKKGG	GM24515 (GM24515)
DH_3117	Drosophila erecta	SKRIRTAFTSTQLLELEREFSHN-AYLSRLRRIEIANRLRLSEKQ-----VKIWFQNRRVKQKKGG	GG13695 (GG13695)
DH_4114	Drosophila yakuba	SKRIRTAFTSTQLLELEREFSHN-AYLSRLRRIEIANRLRLSEKQ-----VKIWFQNRRVKQKKGG	GE19990 (GE19990)
DH_3958	Drosophila willistoni	SKRIRTAFTSTQLLELEREFSHN-AYLSRLRRIEIANRLRLSEKQ-----VKIWFQNRRVKQKKGG	GK17410 (GK17410)
DH_3498	Drosophila persimilis	SKRIRTAFTSTQLLELEREFSHN-AYLSRLRRIEIANRLRLSEKQ-----VKIWFQNRRVKQKKGG	GL25910 (GL25910)
DH_4325	Drosophila pseudoobscura pseudoobscura	SKRIRTAFTSTQLLELEREFSHN-AYLSRLRRIEIANRLRLSEKQ-----VKIWFQNRRVKQKKGG	GA23649 (Dpse\GA23649)
DH_3640	Drosophila grimshawi	SKRIRTAFTSTQLLELEREFSHN-AYLSRLRRIEIANRLRLSEKQ-----VKIWFQNRRVKQKKGG	GH16676 (GH16676)
DH_3854	Drosophila virilis	SKRIRTAFTSTQLLELEREFSHN-AYLSRLRRIEIANRLRLSEKQ-----VKIWFQNRRVKQKKGG	GJ11617 (GJ11617)
DH_3816	Drosophila mojavensis	SKRIRTAFTSTQLLELEREFSHN-AYLSRLRRIEIANRLRLSEKQ-----VKIWFQNRRVKQKKGG	GI11362 (GI11362)
DH_3016	Drosophila ananassae	SKRIRTAFTSTQLLELEREFSQN-AYLSRLRRIEIANRLRLSEKQ-----VKIWFQNRRVKQKKGG	GF10750 (GF10750)
DH_6930	Liolophura japonica	--------------------AAN-MYLSRLRRIEIATYLNLSEKQ-----V---------------	Gsx-like homeodomain protein (Fragment) (Gsx)
DH_7664	Ptychodera flava	SKRIRTAFTSTQLLELEREFAAN-MYLSRLRRIEIATYLNLSEKQ-----VKIWFQNRRVQYKKRR	Transcription factor Gsx (Fragment)
DH_8119	Kelletia kelletii	-----------------REFAAN-MYLSRLRRIEIATLLNLSEKQ-----VKIWF-----------	Ind-like homeodomain-containing protein (Fragment)
DH_12631	Strongylocentrotus purpuratus	SKRIRTAFTSTQLLELEREFAAN-MYLSRLRRIEIATYLNLSEKQ-----VKIWFQNRRVKYKKEG	GLEAN3_13436|Sp-Gsx
DH_6213	Euprymna scolopes	SKRIRTAFTSTQLLELEREFASN-MYLSRLRRIEIATYLNLSEKQ-----VKIWFQNRRVKYKKEG	Gsx homeodomain protein
DH_11548	Branchiostoma floridae	SRRMRTAFSSTQLLELEREFASN-MYLSRLRRIEIATFLNLSEKQ-----VKIWFQNRRVKHKKEA	fgenesh2_pg.scaffold_116000088|Brafl1
DH_4532	Branchiostoma floridae	SRRMRTAFSSTQLLELEREFASN-MYLSRLRRIEIATFLNLSEKQ-----VKIWFQNRRVKHKKEA	Homeobox protein AmphiGsx (Fragment)
DH_11627	Branchiostoma floridae	---MRTAFSSTQLLELEREFASN-MYLSRLRRIEIATFLNLSEKQ-----VKIWFQNRRVKHKKEA	gw.24.256.1|Brafl1
DH_11647	Branchiostoma floridae	---MRTAFSSTQLLELEREFASN-MYLSRLRRIEIATFLNLSEKQ-----VKIWFQNRRVKHKKEA	e_gw.24.254.1|Brafl1
DH_12012	Capitella sp. I Grassle and Grassle, 1976	VKRMRTAFSSTQLLELEREFASN-MYLSRLRRIEIATYLSLSEKQ-----VKIWFQNRRVKFKKEG	estExt_Genewise1.C_7600007|Capca1
DH_5848	Capitella sp. I Grassle and Grassle, 1976	VKRMRTAFSSTQLLELEREFASN-MYLSRLRRIEIATYLSLSEKQ-----VKIWFQNRRVKFKKEG	Gsx (Fragment)
DH_5721	Xenopus tropicalis	GKRMRTAFSSTQLLELEREFSSN-MYLSRLRRIEIATYLSLSEKQ-----VKIWFQNRRVKHKKEN	GS homeobox 2 (Genomic-screened homeobox 2) (gsx2)
DH_199	Homo sapiens	SKRMRTAFTSTQLLELEREFASN-MYLSRLRRIEIATYLNLSEKQ-----VKIWFQNRRVKHKKEG	GS homeobox 1 (GSX1)
DH_5504	Xenopus tropicalis	SKRMRTAFTSTQLLELEREFASN-MYLSRLRRIEIATYLNLSEKQ-----VKIWFQNRRVKHKKEG	Genomic-screened homeobox 1 (gsx1)
DH_2855	Mus musculus	SKRMRTAFTSTQLLELEREFASN-MYLSRLRRIEIATYLNLSEKQ-----VKIWFQNRRVKHKKEG	Gsx1 protein (Genomic screened homeobox 1) (Gsx1)
DH_200	Mus musculus	SKRMRTAFTSTQLLELEREFASN-MYLSRLRRIEIATYLNLSEKQ-----VKIWFQNRRVKHKKEG	GS homeobox 1 (Gsx1)
DH_9521	Homo sapiens	--------TSTQLLELEREFASN-MYLSRLRRIEIATYLNLSEKQ-----VKI-------------	HPX homeobox protein (Fragment)
DH_2125	Haplochromis burtoni	GKRMRTAFTSTQLLELEREFSTN-MYLSRLRRIEIATYLNLSEKQ-----VKIWFQNRRVKHKKEG	Gsh2 (gsh2)
DH_2110	Oryzias latipes	GKRMRTAFTSTQLLELEREFSTN-MYLSRLRRIEIATYLNLSEKQ-----VKIWFQNRRVKHKKEG	GS homeobox 2
DH_11853	Gasterosteus aculeatus	GKRMRTAFTSTQLLELEREFSTN-MYLSRLRRIEIATYLSLSEKQ-----VKIWFQNRRVKHKKEG	GS homeobox 2 (Homeobox protein GSH-2) [Source:UniProtKB/Swiss-Prot;Acc:Q9BZM3]
DH_1818	Danio rerio	GKRMRTAFTSTQLLELEREFSSN-MYLSRLRRIEIATYLNLSEKQ-----VKIWFQNRRVKHKKEG	GS homeobox 2 (gsh2)
DH_7222	Danio rerio	GKRMRTAFTSTQLLELEREFSSN-MYLSRLRRIEIATYLNLSEKQ-----VKIWFQNRRVKHKKEG	Novel homeobox protein (GS homeobox 2) (gsx2)
DH_201	Homo sapiens	GKRMRTAFTSTQLLELEREFSSN-MYLSRLRRIEIATYLNLSEKQ-----VKIWFQNRRVKHKKEG	GS homeobox 2 (GSX2)
DH_202	Mus musculus	GKRMRTAFTSTQLLELEREFSSN-MYLSRLRRIEIATYLNLSEKQ-----VKIWFQNRRVKHKKEG	GS homeobox 2 (Gsx2)
DH_6091	Mus musculus	-----TAFTSTQLLELEREFSSN-MYLSRLRRIEIATYLNLSEKQ-----VKIWFQNRRVKHKKEG	Putative uncharacterized protein (Fragment) (Gsx2)
DH_5053	Amia calva	SKRMRTAFTSTQLLELEREFSSN-MYLSRLRRIEIATYLNLSEKQ-----VKIWFQNRRVKHKKEG	Genomic screen homeobox 1 (Gsh1)
DH_11971	Gasterosteus aculeatus	SKRMRTAFTSTQLLELEREFTSN-MYLSRLRRIEIATYLNLSEKQ-----VKIWFQNRRVKHKKEG	GS homeobox 1 (Homeobox protein GSH-1) [Source:UniProtKB/Swiss-Prot;Acc:Q9H4S2]
DH_4623	Oryzias latipes	SKRMRTAFTSTQLLELEREFTSN-MYLSRLRRIEIATYLNLSEKQ-----VKIWFQNRRVKHKKEG	Developmental transcription factor (Gsh-1)
DH_7172	Danio rerio	SKRMRTAFTSTQLLELEREFTSN-MYLSRLRRIEIATYLNLSEKQ-----VKIWFQNRRVKHKKEG	Genetic screen homeobox 1 protein (GS homeobox 1) (gsx1)
DH_6821	Danio rerio	SKRMRTAFTSTQLLELEREFTSN-MYLSRLRRIEIATYLNLSEKQ-----VKIWFQNRRVKHKKRR	Gsh1 protein (Putative uncharacterized protein) (gsx1)
DH_3376	Salmo salar	----------TQLLELEREFTSN-MYLSRLRRIEIATYLNLSEK----------------------	Genetic screen homeobox 1 protein (Fragment)
DH_6528	Tetraodon nigroviridis	SKRMRTAFTSTQLLELEREFTSN-MYLSRLRRIEIATYLNLSEKQ-----VKIWFQNRRVKTKRRA	Chromosome 7 SCAF14601, whole genome shotgun sequence (GSTENG00019041001)
DH_8958	Phascolion strombus	SKRIRTAFTSTQLLELEREFTSN-MYLSRLRRIEIATYLNL-------------------------	Homeodomain transcription factor Gsx (Fragment) (Gsx)
DH_8957	Nephasoma minuta	SKRIRTAFTSTQLLELEREFNAN-MYLSRLRRIEIATYLNL-------------------------	Homeodomain transcription factor Gsx (Fragment) (Gsx)
DH_5506	Nuttallochiton mirandus	SKRIRTAFTSTQLLELEREFAVN-MYLSRLRRIEIATYLNL-------------------------	Homeobox protein (Fragment) (gsx)
DH_5186	Nereis virens	GKRIRTAFTSTQLLELEREFSSN-MYLSRLRRIEIATYLNLSEKQ-----VKIWFQNRRVKYKKEG	Gsx parahox protein (Fragment) (Gsx)
DH_1864	Platynereis dumerilii	GKRIRTAFTSTQLLELEREFSSN-MYLSRLRRIEIATYLNLSE-----------------------	Gsh (Fragment)
DH_12286	Lottia gigantea	-KRIRTAFTSTQLLELEREFNSS-MYLSRLRRIEIATYLNLSEKQ-----VKIWFQNRRVKYKKD-	gw1.80.263.1|Lotgi1

DH_6854	Tribolium castaneum	SKRIXTAFTSTQLLELEREFASN-MYLSRLRRIEIATCLRLSEKQ-----VKIWFQNRRVKYKKED	Intermediate neuroblasts defective protein (ind)
DH_7032	Oikopleura dioica	RKRSRTAFSGRQLLELENEFLTD-SYLTRLRRVRIAQSLGLSEKQ-----VKIWFQNRRVKQKKEE	Gsx (Gsx)
DH_7971	Diplosoma listerianum	RKRTRTAFTGHQLMELEKEFRAD-MYLTRLRRIQIAQDLLLSEKQ-----VKIWFQNRRVKEKKGN	ParaHox-GSX (Fragment) (gsx)
DH_6289	Ciona intestinalis	RKRMRTAFTGSQLLELEREFHSD-MYLTRLRRIRIAQMLNLSEKQ-----IKIWFQNRRVKKKKGE	Transcription factor protein (Fragment) (Ci-gsx)
DH_9062	Ciona intestinalis	RKRMRTAFTGSQLLELEREFHSD-MYLTRLRRIRIAQMLNLSEKQ-----IKIWFQNRRVKKKKGE	Gsx
DH_1977	Nematostella vectensis	SKRIRTAYTSMQLLELEKEFSQN-RYLSRLRRIQIAALLDLSEKQ-----VKIWFQNRRVKWKKDK	Predicted protein (v1g91593)
DH_9645	Nematostella vectensis	SKRIRTAYTSMQLLELEKEFSQN-RYLSRLRRIQIAALLDLSEKQ-----VKIWFQNRRVKWKKDK	Hox2 protein (Fragment) (hox2)
DH_5112	Nematostella vectensis	SKRIRTAYTSMQLLELEKEFSQN-RYLSRLRRIQIAALLDLSEKQ-----VKIWFQNRRVKWKKDK	GSX-ANTP class homeobox protein (Fragment)
DH_4978	Nematostella vectensis	SKRIRTAYTSMQLLELEKEFSQN-RYLSRLRRIQIAALLDLSEKQ-----VKIWFQNRRVKWKKDK	GSX (Fragment)
DH_9053	Acropora millepora	SKRIRTAYTSMQLLELEKEFSQN-RYLSRLRRIQIAALLDLSEKQ-----VKIWFQNRRVKWKKDK	Homeodomain protein cnox-2 (cnox-2)
DH_4449	Hydractinia symbiolongicarpus	VKRIRTAYTSIQLLELEKEFQNN-RYLSRLRRIQIAAMLDLTEKQ-----VKIWFQNRRVKWKKDK	Hox homeobox protein (Cnox-2)
DH_5962	Hydractinia symbiolongicarpus	VKRIRTAYTSIQLLELEKEFQNN-RYLSRLRRIQIAAMLDLTEKQ-----VKIWFQNRRVKWKKDK	Cnox-2
DH_9183	Podocoryne carnea	AKRIRTAYTSIQLLELEKEFQNN-RYLSRLRRIQIAAMLDLTEKQ-----VKIWFQNRRVKWKKDK	GSX transcription factor (Gsx)
DH_9602	Cassiopea xamachana	SKRIRTAYTSIQLLELEKEFQNN-RYLSRLRRIQIAAMLDLTEKQ-----VKIWFQNRRVKWKKDK	Scox-2 homeodomain protein (Fragment) (Scox-2)
DH_9181	Sarsia sp. 'Long Island Sound'	SKRIRTAYTSIQLLELEKGFQNN-RYLSRLRRIQIAAMLHLTEKQ-----VKIWFQNRRVKWKKDK	Homeodomain protein Cnox-2 (Fragment) (Cnox-2)
DH_5537	Chlorohydra viridissima	SKRIRTAYTSIQLLELEKEFQNN-RYLSRLRRIQIAAILDLTEKQ-----VKIWFQNRRVKWKKDK	Cnox2 protein (Fragment) (cnox2)
DH_5984	Chlorohydra viridissima	SKRIRTAYTSIQLLELEKEFQNN-RYLSRLRRIQIAAILDLTEKQ-----VKIWFQNRRVKWKKDK	Cnox-2 homeoprotein (cnox-2)
DH_5589	Hydra attenuata	SKRIRTAYTSIQLLELEKEFQNN-RYLSRLRRIQIAAILDLTEKQ-----VKIWFQNRRVKWKKDK	Cnox-2 protein (Cnox-2)
DH_9256	Hydra attenuata	SKRIRTAYTSIQLLELEKEFQNN-RYLSRLRRIQIAAILDLTEKQ-----VKIWFQNRRVKWKKDK	Cnox-2 homeoprotein (cnox-2)
DH_3232	Trichoplax adhaerens	TKRIRTAYTSMQLLELEKEFNSS-RYLSRLRRIEIANMLNLSEKQ-----VKIWFQNRRVKWKKDN	Trox-2 homeobox transcription factor (TRIADDRAFT_33728)
DH_7698	Trichoplax adhaerens	TKRIRTAYTSMQLLELEKEFNSS-RYLSRLRRIEIANMLNLSEKQ-----VKIWFQNRRVKWKKDN	Trox-2 homeobox transcription factor (Hox/ParaHox protein) (Trox-2)
DH_4530	Trichoplax adhaerens	TKRIRTAYTSMQLLELEKEFNSS-RYLSRLRRIEIANMLNLSEKQ-----VKIWFQNRRVKWKKDN	Hox protein (Fragment) (Trox-2)
DH_4349	Trichoplax adhaerens	TKRIRTAYTSMQLLELEKEFNSS-RYLSRLRRIEIANMLNLSEKQ-----VKIWFQNRRVKWKKDN	Trox-2/Gsx (Fragment)

Save alignment [fasta format] - Save complete sequences [fasta format]

XLOX

ID	organism	10 20 30 40 50 60	descrip
DH_1256	Nemertoderma westbladi	--------------------HFNKYISRPRRIELAAMLNLTERHIKIWFQNRRMKWKKDE	Xlox (Fragment)
DH_12265	Lottia gigantea	NKRTRTAYTRGQLLELEKEFHFNKYISRPRRIELAAMLNLTERHIKIWFQNRRMKWKKDE	gw1.80.268.1|Lotgi1
DH_1573	Euprymna scolopes	NKRTRTAYTRGQLLELEKEFHFNKYISRPRRIELAAMLNLTERHIKIWFQNRRMKWKKDE	Xlox (Fragment)
DH_8146	Archaster typicus	NKRTRTAYTRGQLLELEKEFHFNKYISRPRRIELAAMLNLTERHIKIWFQNRRMKWKKEE	Homeodomain transcription factor Xlox (Xlox)
DH_12650	Strongylocentrotus purpuratus	NKRTRTAYTRGQLLELEKEFHFNKYISRPRRIELAAMLNLTERHIKIWFQNRRMKWKKEE	GLEAN3_26099|Sp-lox
DH_8382	Strongylocentrotus purpuratus	NKRTRTAYTRGQLLELEKEFHFNKYISRPRRIELAAMLNLTERHIKIWFQNRRMKWKKEE	Homeobox protein Splox
DH_9440	Branchiostoma floridae	NKRTRTAYTRGQLLELEKEFHFNKYISRPRRIELAAMLNLTERHIKIWFQNRRMKWKKEQ	Homeobox protein AmphiXlox (Fragment)
DH_11584	Branchiostoma floridae	NKRTRTAYTRGQLLELEKEFHFNKYISRPRRIELAAMLNLTERHIKIWFQNRRMKWKKEQ	gw.24.415.1|Brafl1
DH_7973	Diplosoma listerianum	NKRTRTAYTRKQLLELEKEFHFNKYISRPRRIELAAGLNLTERHIKIWFQNR--------	ParaHox-XLOX (Fragment) (xlox)
DH_7662	Ptychodera flava	NKRTRTAYTRSQLLELEKEFHFNKYISRPRRIELAAMLNLTERHIKIWFQNRRMKFKKEE	Transcription factor Lox2
DH_7663	Ptychodera flava	NKRTRTAYTRSQLLELEKEFHFNKYISRPRRIELAAMLNLTERHIKIWFQNRRMKFKKEE	Transcription factor Lox1
DH_5010	Nereis virens	NKRTRTAYTRSQLLELEKEFHFNKYISRPRRIELASMLSLTERHI---------------	Xlox parahox protein (Fragment) (Xlox)
DH_8954	Nephasoma minuta	------AYTRAQLLELEKEFHFNKYISRPRRIELAAMLNLTERHIKI-------------	Homeodomain transcription factor Xlox (Fragment) (Xlox)
DH_8955	Phascolion strombus	NKRTRTAYTRAQLLELEKEFHFNKYISRPRRIELAAMLNLTERHIKIWFQNRRMKWKKDE	Homeodomain transcription factor Xlox (Fragment) (Xlox)
DH_1481	Ateles geoffroyi	NKRTRTAYTRAQLLELEKEFLFNKYISRPRRVELAVMLNLTERHIKIWFQNRRMKWKKEE	IPF1 (Fragment) (IPF1)
DH_7526	Rattus sp	------------------EFLFNKYISRPRRVELAVMLNLTERHIKIWFQNRRMKWKKEE	Homeoprotein 13A (Fragment) (RHbox-13A)
DH_1496	Lagothrix lagotricha	NKRTRTAYTRAQLLELEKEFLFNKYISRPRRVELAVMLNLTERHIKIWFQNRRMKWKKEE	IPF1 (Fragment) (IPF1)
DH_934	Pan troglodytes	NKRTRTAYTRAQLLELEKEFLFNKYISRPRRVELAVMLNLTERHIKIWFQNRRMKWKKEE	Pancreas/duodenum homeobox protein 1 (PDX1)
DH_7244	Homo sapiens	NKRTRTAYTRAQLLELEKEFLFNKYISRPRRVELAVMLNLTERHIKIWFQNRRMKWKKEE	Pancreatic and duodenal homeobox 1 (Insulin promoter factor 1, homeodomain transcription factor) (PDX1)
DH_932	Mus musculus	NKRTRTAYTRAQLLELEKEFLFNKYISRPRRVELAVMLNLTERHIKIWFQNRRMKWKKEE	Pancreas/duodenum homeobox protein 1 (Pdx1)
DH_931	Mesocricetus auratus	NKRTRTAYTRAQLLELEKEFLFNKYISRPRRVELAVMLNLTERHIKIWFQNRRMKWKKEE	Pancreas/duodenum homeobox protein 1 (PDX1)
DH_262	Xenopus laevis	NKRTRTAYTRAQLLELEKEFLFNKYISRPRRVELAVMLNLTERHIKIWFQNRRMKWKKEE	Homeobox protein 8 (xlhbox8)
DH_1513	Macaca mulatta	NKRTRTAYTRAQLLELEKEFLFNKYISRPRRVELAVMLNLTXRHIKIWFQNRRMKWKKEE	IPF1 (Fragment) (IPF1)
DH_9131	Sus scrofa	NKRTRTAYTRAQLLELEKEFLFNKYISRPRRVELAVML----------------------	Homeodomain protein PDX-1 (Fragment)
DH_933	Pan paniscus	NKRTRTAYTRAQLLELEKEFLFNKYISRPRRVELAVMLNLTERHIKIWFQNRRMKWKKEE	Pancreas/duodenum homeobox protein 1 (PDX1)
DH_1584	Macaca nemestrina	NKRTRTAYTRAQLLELEKEFLFNKYISRPRRVELAVMLNLTERHIKIWFQNRRMKWKKEE	IPF1 (Fragment) (IPF1)
DH_930	Homo sapiens	NKRTRTAYTRAQLLELEKEFLFNKYISRPRRVELAVMLNLTERHIKIWFQNRRMKWKKEE	Pancreas/duodenum homeobox protein 1 (PDX1)
DH_6192	Mus musculus	NKRTRTAYTRAQLLELEKEFLFNKYISRPRRVELAVMLNLTERHIKIWFQNRRMKWKKEE	Pancreatic and duodenal homeobox 1 (Pdx1)
DH_5052	Amia calva	NKRTRTAYTRAQLLELEKEFLFNKYISRPRRVELAVMLNLTERHIKIWFQNRRMKWKKEE	Pancreatic and duodenal homeobox (Pdx1)
DH_5050	Polypterus senegalus	NKRTRTAYTRAQLLELEKEFLFNKYISRPRRVELAVMLNLTERHIKIWFQNRRMKWKKEE	Pancreatic and duodenal homeobox (Fragment) (Pdx1)
DH_935	Rattus norvegicus	NKRTRTAYTRAQLLELEKEFLFNKYISRPRRVELAVMLNLTERHIKIWFQNRRMKWKKEE	Pancreas/duodenum homeobox protein 1 (Pdx1)
DH_929	Gorilla gorilla gorilla	NKRTRTAYTRAQLLELEKEFLFNKYISRPRRVELAVMLNLTERHIKIWFQNRRMKWKKEE	Pancreas/duodenum homeobox protein 1 (PDX1)
DH_11709	Gasterosteus aculeatus	NKRTRTAYTRAQLLELEKEFLFNRYISRPRRVELALTLSLTERHIKIWFQNRRMKWKKEE	Pancreas/duodenum homeobox protein 1 (PDX-1) (Insulin promoter factor 1) (IPF-1) (Islet/duodenum homeobc
DH_9375	Oryzias latipes	--------------------LFNRYISRPRRVELALTLSLTERHIKIWFQNRRMKWKKEE	PDX (Fragment) (pdx)
DH_6490	Tetraodon nigroviridis	NKRTRTAYTRAQLLELEKEFLFNRYISRPRRVELALTLNLTERHIKIWFQNRRMKWKKEE	Chromosome 6 SCAF14737, whole genome shotgun sequence. (Fragment) (GSTENG00023870001)
DH_4567	Danio rerio	NKRTRTAYTRAQLLELEKEFLFNKYISRPRRVELALTLSLTERHIKIWFQNRRMKWKKEE	Homeobox protein PDX-1 (pdx1)
DH_7404	Danio rerio	NKRTRTAYTRAQLLELEKEFLFNKYISRPRRVELALTLSLTERHIKIWFQNRRMKWKKEE	Pancreatic and duodenal homeobox 1 (pdx1)
DH_4497	Helobdella triserialis	NKRTRTAYSRSQLLELEKEFHFDKYISRPRRVELASSLNLTERHIKIWFQNRRMKWKKME	Lox3 (Fragment) (A2)
DH_12316	Helobdella robusta	NKRTRTAYSRSQLLELEKEFHFDKYISRPRRVELASSLNLTERHIKIWFQNRRMKWKKME	estExt_Genewise1.C_300434|Helro1
DH_264	Helobdella triserialis	NKRTRTAYSRSQLFELEKEFHFDKYISRPRRVELASSLNLTERHIKIWFQNRRMKWKKME	Homeobox protein HTR-A2 (Fragment)
DH_5670	Hirudo medicinalis	NKRTRTAYSRAQLLELEKEFHYDKYISRPRRLELAASLNLTECHIKIWFQNRRMKWEKFE	Homeobox protein (Fragment) (Lox3C)
DH_5671	Hirudo medicinalis	NKRTRTAYSRAQLLELEKEFHYDKYISRPRRLELAASLNLTECHIKIWFQNRRMKWEKFE	Homeobox protein (Fragment) (Lox3)
DH_5669	Hirudo medicinalis	NKRTRTAYSRAQLLELEKEFHYDKYISRPRRVELAASLNLTECHIKIWFQNRRMKWEKFE	Lox3B protein (Fragment) (Lox3B)
DH_5668	Hirudo medicinalis	NKRTRTAYSRAQLLELEKEFHYDKYISRPRRLELAASLNLTERHIKIWFQNRRMKWKKLE	Lox3A protein (Fragment) (Lox3A)
DH_6699	Perionyx excavatus	NKRTRTAYTRAQLLELEKEFHFDKYISRPRRVELAGLLNLTERHIKIWFQNRR-------	Homeodomain transcription factor Xlox (Fragment)
DH_11994	Capitella sp. I Grassle and Grassle, 1976	NKRTRTAYTRAQLLELEKEFHFNRYITRPRRVELAAHLNLTEQHIKIWFQNRRMKWKKDV	fgenesh1_pg.C_scaffold_444000011|Capca1
DH_5849	Capitella sp. I Grassle and Grassle, 1976	NKRTRTAYTRAQLLELEKEFHFNRYITRPRRVELAAHLNLTEQHIKIWFQNRRMKWKKDV	Xlox protein (Xlox)
DH_9141	Ciona intestinalis	NKRTRTAYTRWQLLELEKEFHFSRYISRPRRIELAAMLNLTERHIKIWFQNRRMKWKKDQ	Transcription factor (IPF1)
