# Supplementary material for: A non-tree-based comprehensive study of metazoan Hox and ParaHox genes prompts new insights into their origin and evolution
Source: BMC Evol Biol. 2010 Mar 11;10:73. doi: 10.1186/1471-2148-10-73 (PMC2842273; doi:10.1186/1471-2148-10-73)
Supplement: Additional file 4 — Accession numbers of training sequences. List of accession number and name of sequences used in the training dataset. Corresponding multiple alignments are available upon request. [file 1471-2148-10-73-S4.PDF]

Accession number and names of the sequences used in the training dataset

UniProtKB	
A0FDP3	HOX2.SACKO
A0FDP5	Hox6.SACKO
A0FDP6	Hox9_10.SACKO
A0FDP7	Hox11_13a.SACKO
A0FDP8	Hox11_13b.SACKO
A1IGZ1	HOX1.Mro
A1IGZ2	HOX2.Mro
A1IGZ3	Hox4.Mro
A1IGZ4	Hox5.Mro
A1IGZ5	Hox7.Mro
A1IGZ6	Hox8.Mro
A1IGZ7	Hox9_10.Mro
A1IGZ8	Hox11_13c.Mro
A4F5H2	Scr1.CUPSA
A4F5H4	Dfd2.CUPSA
A8P9J6	ABDB.BRUMA
A9X3X0	ABDB.Esp
B0W1V6	ABDB.CULQU
B0W3N4	PG1.CULQU
HM13_CAEEL	HM13.CAEEL
HOX3_BRAFL	HOX3.BRAFL
LIN39_CAEEL	LIN39.CAEEL
MAB5_CAEEL	MAB5.CAEEL
O18313	Hox5.CIOIN
O44262	ABDA.Aka
O44268	Scr.Aka
O44269	UBX.Aka
O62523	Hox3.CUPSA
O62548	Hox1.LINSA
O62549	Hox3.LINSA
O62550	Hox6.LINSA
O62551	Hox7.LINSA
O62552	Hox9.LINSA
O76842	LAB.CUPSA
O76843	Dfd.CUPSA
O76846	UBX2.CUPSA
O76937	HoxC.DUGTI
O76938	HoxD.DUGTI
O96896	Scr.Sca
P02833	ANTP.DROME
P07548	DFD.DROME
P09077	SCR.DROME
P09087	ABDB.DROME
P10105	LAB.DROME
P15856	ABDA.APIME
P29555	ABDA.DROME
P31264	PB.DROME
P81192	Hox4.LINSA
P83949	UBX.DROME
P91712	HoxA.DUGTI
P91714	HoxF.DUGTI
Q0QII7	Hox7..NERVI
Q17136	Hox10.BRAFL
Q17140	Hox5.BRAFL
Q17141	Hox6.BRAFL
Q17142	Hox7.BRAFL
Q17143	Hox8.BRAFL
Q17144	Hox9.BRAFL
Q17188	ANTP.BOMMO
Q17AS0	PG1.AEDAE
Q1A1Y5	ABDB.ANOGA
Q1G689	Scr.Esp
Q1G690	Dfd.Esp
Q1G691	PB.Esp
Q1KY83	ABDA_like.Sma
Q309F5	LAB.Sca
Q4H3C6	Hox4.CIOIN
Q4H3C7	Hox3.CIOIN
Q4H3C9	Hox13.CIOIN

Species abbreviations

Acanthokara kaputensis	Aka
Capitella	Capca
Endeis spinosa	Esp
Euprymna scolopes	Esc
Glomeris marginata	Gma
Haliotis asinina	Has
Lottia gigantea	Lotgi
Metacrinus rotundus	Mro
Priapulus caudatus	Pca
Ptychodera flava	Pfl
Sacculina carcini	Sca
Strigamia maritima	Sma
Symsagittifera roscoffensis	Sro
Thermobia domestica	Tdo
Xenoturbella bocki	Xbo

Also see the Uniprot species list
http://www.uniprot.org/docs/speclist

Q4H3D0	Hox12.CIOIN
Q4H3D1	Hox10.CIOIN
Q4H3D2	HOX1.CIOIN
Q4VWT5	Hox13.OIKDI
Q4VWU0	Hox4.OIKDI
Q4VWU1	HOX2.OIKDI
Q5D1N8	Smox1.SCHMA
Q5U8M1	ABDB..PORSC
Q66S38	Hox10.OIKDI
Q66S86	Hox4.1.OIKDI
Q675P0	HOX1.OIKDI
Q675T8	Hox9.OIKDI
Q675V3	Hox11.OIKDI
Q675Y2	Hox12.OIKDI
Q6T4Q7	Hox11_13c.Pfl
Q6T4Q8	Hox11_13b.Pfl
Q6T4Q9	Hox11_13a.Pfl
Q6T4R0	Hox9_10.Pfl
Q6T4R1	Hox6.Pfl
Q6T4R2	Hox5.Pfl
Q6T4R3	Hox4.Pfl
Q6T4R4	HOX1.Pfl
Q6V4Z7	LAB.SCHMA
Q6V4Z8	ABDA.SCHMA
Q6V7W5	Dfd.SCHMA
Q75WS3	LAB.BOMMO
Q7Q4Z9	PG1.ANOGA
Q7YSX9	Post.Sro
Q7YTB7	Hox4.SACKO
Q7YTB8	HOX1.SACKO
Q7YTC5	Hox11_13c.SACKO
Q7YTC6	Hox7.SACKO
Q7Z0F3	SrHox4_5.Sro
Q7Z0F4	Hox1.Sro
Q86LC3	ABDB.Sca
Q8I7A4	HOX1.PLADU
Q8I7C9	Hox6_7.CIOIN
Q8I7D1	HOX2.CIOIN
Q8WQR6	Post2.Esc
Q8WQR7	Post1.Esc
Q8WQR9	ANTP.Esc
Q8WQS0	Lox5.Esc
Q8WQS1	Scr.Esc
Q8WQS2	Hox3.Esc
Q95UQ9	LAB.TRICA
Q9BLF8	ABDBb.DUGJA
Q9BLF9	ABDBa.DUGJA
Q9BN32	ABDA.PORSC
Q9GYV0	ANTP.APIME
Q9N2K2	Hox14.BRAFL
Q9NAZ0	Hox13.BRAFL
Q9NAZ1	Hox12.BRAFL
Q9NAZ2	Hox11.BRAFL
Q9NHB7	Scr.TRICA
Q9U9T2	Post2.NERVI
Q9U9T3	Post1.NERVI
Q9U9T4	Lox5..NERVI
Q9U9T8	Scr.NERVI
Q9U9T9	Dfd.NERVI
Q9U9U0	Hox3.NERVI
Q9U9U1	PB.NERVI
Q9U9Z0	Post1.LINUN
Q9U9Z1	Lox4.LINUN
Q9U9Z3	Lox5.LINUN
Q9U9Z4	ANTP.LINUN
Q9U9Z5	Scr.LINUN
Q9U9Z6	Hox3.LINUN
Q9U9Z7	LAB.LINUN
Q9UAL6	HOX1.BRAFL
Q9UAL7	HOX2.BRAFL
Q9UAL8	Hox4.BRAFL
Q9XW88_CAEEL	PHP3.CAEEL
Q9XY00	Plox3.DUGJA
Q9XY01	Plox4.DUGJA

Q9XY02	Plox5.DUGJA
Q9XY03	Plox6.DUGJA
Q9XZR3	ABDB.CUPSA
Q9XZR4	Scr.BOMMO
Q9Y183	ABDB.Pca
Q9Y184	HB4.Pca
Q9Y185	UBX.Pca
Q9Y186	HB3.Pca
Q9Y187	HB2.Pca
UCSC genome browser A. mellifera Jan. 2005 Assembly	
AmeLG16_WGA452_2.502725.502725.m_prot	LAB.APIME
HomeoDB: http://homeodb.cbi.pku.edu.cn/	
Bfl_Hox15	Hox15.BRAFL
Refseq	
XP_623903.1	Scr.APIME
XP_623989.2	UBX.APIME
XP_781650	Hox11_13c.STPUR
XP_781966	HOX1.STRPU
Annotation of AC165428 (Genbank)	
AC165428_11a	Hox11_13a.STPUR
AC165428_11b	Hox11_13b.STPUR
AC165428_2	HOX2.STRPU
AC165428_3	Hox3.STRPU
AC165428_5	Hox5.STPUR
AC165428_6	Hox6.STPUR
AC165428_7	Hox7.STPUR
AC165428_8	Hox8.STPUR
AC165428_9	Hox9_10.STPUR
JGI	
e_gw1.12.439.1	Hox4.Logti
e_gw1.12.69.1	PB.Lotgi
e_gw1.292.19.1	Post2.Capca
e_gw1.70.60.1	Hox8.Capca
estExt_fgenesh1_pg.C_2920001	Lox2.Capca
estExt_fgenesh1_pg.C_700021	Hox1.Capca
estExt_fgenesh1_pg.C_700024	Hox3.Capca
estExt_Genewise1.C_700045	Hox4.Capca
estExt_Genewise1.C_sca_120441	Lox4.Logti
estExt_Genewise1Plus.C_700047	Hox5.Capca
estExt_Genewise1Plus.C_700050	Hox6.Capca
fgenesh1_pg.C_scaffold_33000038	Post1.Capca
fgenesh2_pg.C_sca_12000235	Hox3.Lotgi
fgenesh2_pg.C_sca_12000242	Lox2.Logti
fgenesh2_pg.C_sca_12000246	Post1.Lofgi
fgenesh2_pm.C_sca_12000048	Hox7.Logti
gw1.12.429.1	Hox5.Logti
gw1.12.445.1	Hox6.Logti
gw1.12.448.1	Post2.Lotgi
gw1.12.670.1	LAB.Lotgi
vertebrate sequences manually curated in [1]	
A1_CHICK	A1.CHICK
A1_HETFR	A1.HETFR
A1_MONDO	A1.MONDO
A1_MOUSE	A1.MOUSE
A1_XENLA	A1.XENLA
A10_LATME	A10.LATME
A10_MOUSE	A10.MOUSE
A10_XENTR	A10.XENTR
A10a_FUGRU	A10a.FUGRU
A10b_BRARE	A10b.BRARE
A10b_FUGRU	A10b.FUGRU
A11_HETFR	A11.HETFR
A11_MOUSE	A11.MOUSE
A11_POLSP	A11.POLSP
A11a_BRARE	A11a.BRARE
A11a_FUGRU	A11a.FUGRU
A11a_ORYLA	A11a.ORYLA
A11b_BRARE	A11b.BRARE
A11b_FUGRU	A11b.FUGRU
A13_HETFR	A13.HETFR
A13_MOUSE	A13.MOUSE
A13a_BRARE	A13a.BRARE
A13a_FUGRU	A13a.FUGRU
A13a_ORYLA	A13a.ORYLA

A13b_BRARE	A13b.BRARE
A13b_FUGRU	A13b.FUGRU
A13b_ORYLA	A13b.ORYLA
A13b_TETNG	A13b.TETNG
A14_LATME	A14.LATME
A1a_BRARE	A1a.BRARE
A2_LATME	A2.LATME
A2_MOUSE	A2.MOUSE
A2a_FUNHE	A2a.FUNHE
A2b_BRARE	A2b.BRARE
A2b_FUGRU	A2b.FUGRU
A2b_ORENI	A2b.ORENI
A2b_ORYLA	A2b.ORYLA
A2bi_ONCMY	A2bi.ONCMY
A3_HETFR	A3.HETFR
A3_MOUSE	A3.MOUSE
A3a_BRARE	A3a.BRARE
A3EYA2	Hox3a.Sma
A3EYA3	Hox3b.Sma
A4_MOUSE	A4.MOUSE
A4a_BRARE	A4a.BRARE
A4a_FUGRU	A4a.FUGRU
A4aii_ONCMY	A4aii.ONCMY
A5_AMBME	A5.AMBME
A5_HETFR	A5.HETFR
A5_MOUSE	A5.MOUSE
A5a_BRARE	A5a.BRARE
A6_HETFR	A6.HETFR
A6_LATME	A6.LATME
A6_MOUSE	A6.MOUSE
A7_COTJA	A7.COTJA
A7_HETFR	A7.HETFR
A7_LATME	A7.LATME
A7_MOUSE	A7.MOUSE
A7a_MORSA	A7a.MORSA
A7a_ORYLA	A7a.ORYLA
A9_HETFR	A9.HETFR
A9_MOUSE	A9.MOUSE
A9a_BRARE	A9a.BRARE
A9a_FUGRU	A9a.FUGRU
A9b_BRARE	A9b.BRARE
A9b_FUGRU	A9b.FUGRU
A9b_ORENI	A9b.ORENI
A9b_ORYLA	A9b.ORYLA
B1_AMBME	B1.AMBME
B1_CHICK	B1.CHICK
B1_HUMAN	B1.HUMAN
B1_LATME	B1.LATME
B1_MONDO	B1.MONDO
B1_MOUSE	B1.MOUSE
B1_RAT	B1.RAT
B10a_BRARE	B10a.BRARE
B13_AMBME	B13.AMBME
B13_CANFA	B13.CANFA
B13_CHICK	B13.CHICK
B13_LATME	B13.LATME
B13_MOUSE	B13.MOUSE
B13a_FUGRU	B13a.FUGRU
B13a_ORYLA	B13a.ORYLA
B13a_SPHNE	B13a.SPHNE
B1a_BRARE	B1a.BRARE
B1a_ORYLA	B1a.ORYLA
B1a_TETNG	B1a.TETNG
B1b_BRARE	B1b.BRARE
B1b_FUGRU	B1b.FUGRU
B1b_ORENI	B1b.ORENI
B1b_ORYLA	B1b.ORYLA
B1b_TETNG	B1b.TETNG
B2_HUMAN	B2.HUMAN
B2_MONDO	B2.MONDO
B2_MOUSE	B2.MOUSE
B2a_MORSA	B2a.MORSA
B2a_ORENI	B2a.ORENI
B2a_SALSA	B2a.SALSA

B3_MOUSE	B3.MOUSE
B3_PLEWA	B3.PLEWA
B3a_FUGRU	B3a.FUGRU
B3b_FUGRU	B3b.FUGRU
B3b_ORENI	B3b.ORENI
B3b_ORYLA	B3b.ORYLA
B4_LATME	B4.LATME
B4_MOUSE	B4.MOUSE
B4_XENTR	B4.XENTR
B4a_BRARE	B4a.BRARE
B5_MOUSE	B5.MOUSE
B5_PANTR	B5.PANTR
B5_TRISI	B5.TRISI
B5_XENLA	B5.XENLA
B5a_FUGRU	B5a.FUGRU
B5bi_ONCMY	B5bi.ONCMY
B6_CHICK	B6.CHICK
B6_LATME	B6.LATME
B6_MONDO	B6.MONDO
B6_MOUSE	B6.MOUSE
B6a_BRARE	B6a.BRARE
B6a_FUGRU	B6a.FUGRU
B6b_BRARE	B6b.BRARE
B6b_FUGRU	B6b.FUGRU
B6b_ORYLA	B6b.ORYLA
B7_MOUSE	B7.MOUSE
B7_RAT	B7.RAT
B7_XENLA_a	B7.XENLA.a
B7_XENLA_b	B7.XENLA.b
B7a_BRARE	B7a.BRARE
B7a_ORENI	B7a.ORENI
B8_MOUSE	B8.MOUSE
B8a_FUGRU	B8a.FUGRU
B8b_BRARE	B8b.BRARE
B9_MOUSE	B9.MOUSE
B9_XENLA_1	B9.XENLA.1
B9_XENLA_SP	B9.XENLA.SP
B9a_BRARE	B9a.BRARE
B9a_FUGRU	B9a.FUGRU
C1_LATME	C1.LATME
C10_LATME	C10.LATME
C10_MOUSE	C10.MOUSE
C10a_FUGRU	C10a.FUGRU
C11_HUMAN	C11.HUMAN
C12_LATME	C12.LATME
C12_MOUSE	C12.MOUSE
C12_XENTR	C12.XENTR
C12a_FUGRU	C12a.FUGRU
C12a_ORYLA	C12a.ORYLA
C12a_TETNG	C12a.TETNG
C13_CHICK	C13.CHICK
C13_LATME	C13.LATME
C13_MOUSE	C13.MOUSE
C13_PLEWA	C13.PLEWA
C13a_BRARE	C13a.BRARE
C13a_FUGRU	C13a.FUGRU
C13a_ORENI	C13a.ORENI
C13a_ORYLA	C13a.ORYLA
C13a_TETNG	C13a.TETNG
C13b_BRARE	C13b.BRARE
C1a_BRARE	C1a.BRARE
C3a_BRARE	C3a.BRARE
C3a_ORENI	C3a.ORENI
C3a_ORYLA	C3a.ORYLA
C4_MOUSE	C4.MOUSE
C4a_BRARE	C4a.BRARE
C4a_FUGRU	C4a.FUGRU
C4a_ORYLA	C4a.ORYLA
C5_MOUSE	C5.MOUSE
C5_XENLA	C5.XENLA
C5a_FUGRU	C5a.FUGRU
C5a_ORYLA	C5a.ORYLA
C5a_TETNG	C5a.TETNG
C6_MOUSE	C6.MOUSE

C6_NOTVI	C6.NOTVI
C6a_BRARE	C6a.BRARE
C6b_BRARE	C6b.BRARE
C8_MOUSE	C8.MOUSE
C8_XENLA	C8.XENLA
C8a_BRARE	C8a.BRARE
C9_CHICK	C9.CHICK
C9_MOUSE	C9.MOUSE
C9a_FUGRU	C9a.FUGRU
C9a-1-R_ONCMY	C9a_1_R.ONCMY
D1_BOVIN	D1.BOVIN
D1_HUMAN	D1.HUMAN
D1_LATME	D1.LATME
D1_MACMU	D1.MACMU
D1_MONDO	D1.MONDO
D1_MOUSE	D1.MOUSE
D1_RAT	D1.RAT
D1_XENLA	D1.XENLA
D1_XENTR	D1.XENTR
D10_AMBME	D10.AMBME
D10_HETFR	D10.HETFR
D10_LATME	D10.LATME
D10_MOUSE	D10.MOUSE
D10a_BRARE	D10a.BRARE
D10a_DANAA	D10a.DANAA
D10a_DANAE	D10a.DANAE
D10a_DANAT	D10a.DANAT
D10a_DANFR	D10a.DANFR
D10a_ORYLA	D10a.ORYLA
D10a_RASEL	D10a.RASEL
D10a_TETNG	D10a.TETNG
D11_HETFR	D11.HETFR
D11_MOUSE	D11.MOUSE
D11a_BRARE	D11a.BRARE
D11a_FUGRU	D11a.FUGRU
D11a_ORENI	D11a.ORENI
D11a_ORYLA	D11a.ORYLA
D12_CARPS	D12.CARPS
D12_CHICK	D12.CHICK
D12_HETFR	D12.HETFR
D12_HUMAN	D12.HUMAN
D12_LATME	D12.LATME
D12_MOUSE	D12.MOUSE
D12a_BRARE	D12a.BRARE
D12a_FUGRU	D12a.FUGRU
D12a_ORENI	D12a.ORENI
D12a_ORYLA	D12a.ORYLA
D13_HETFR	D13.HETFR
D13_MOUSE	D13.MOUSE
D13_PLEWA	D13.PLEWA
D13_XENTR	D13.XENTR
D13a_BRARE	D13a.BRARE
D14_HETFR	D14.HETFR
D3_LATME	D3.LATME
D3_MOUSE	D3.MOUSE
D3a_FUGRU	D3a.FUGRU
D4_LATME	D4.LATME
D4_MOUSE	D4.MOUSE
D4_XENLA	D4.XENLA
D4_XENTR	D4.XENTR
D4a_BRARE	D4a.BRARE
D4a_FUGRU	D4a.FUGRU
D4b_FUGRU	D4b.FUGRU
D4b_ORYLA	D4b.ORYLA
D4b_SPHNE	D4b.SPHNE
D5_HETFR	D5.HETFR
D8_AMBME	D8.AMBME
D8_HUMAN	D8.HUMAN
D8_LATME	D8.LATME
D8_MOUSE	D8.MOUSE
D9_HETFR	D9.HETFR
D9_LATME	D9.LATME
D9_MOUSE	D9.MOUSE
D9a_BRARE	D9a.BRARE

D9a_FUGRU	D9a.FUGRU
D9a_ORENI	D9a.ORENI
D9a_ORYLA	D9a.ORYLA
D9a_TETNG	D9a.TETNG
D9b_FUGRU	D9b.FUGRU
D9b_ORENI	D9b.ORENI
D9b_ORYLA	D9b.ORYLA

[1]: Thomas-Chollier et al. **HoxPred: automated classification of Hox proteins using combinations of generalised profiles**. BMC Bioinformatics (2007) vol. 8 pp. 247
see additionnal file 1 for corresponding accession numbers

Accession number and names of the sequences used in the training dataset: ParaHox

**UniprotKB**	
Q5EVF1	Cdx1.OIKDI
Q5EVE9	Cdx3.OIKDI
CDX2_HUMAN	CDX2.HUMAN
CDX2_MOUSE	CDX2.MOUSE
CDX4_HUMAN	CDX4.HUMAN
CDX4_MOUSE	CDX4.MOUSE
HMD2_CHICK	HMD2.CHICK
CDX1_XENLA	CDX1.XENLA
CDX1_XENTR	CDX1.XENTR
CDX1_HUMAN	CDX1.HUMAN
O96714	Cdx.TRICA
P09085	Cad.DROME
Q4H3T1	Cdx.CIOIN
O96715	Cdx.TRICA
P34766	Pal1.CAEEL
Q7KUL4	Ind.DROME
Q0N4M5	Gsx.NEMVE
GSH2_HUMAN	GSH2.HUMAN
GSHI_HUMAN	GSHI.HUMAN
Q58Y77	Ind.TRICA
Q4H3E7	GSX.CIOIN
Q5EVF3	GSX.OIKDI
Q9GP86	IPF1.CIOIN
IPF1_HUMAN	IPF1.HUMAN
**HomeoDB: http://homeodb.cbi.pku.edu.cn/**	
Cdx|Cdx|Amphioxus|HD1	Cdx.BRAFLE
Gsx|Gsx|Amphioxus|HD1	Gsx.BRAFLE
Pdx|Xlox|Amphioxus|HD1	Xlox.BRAFLE
**JGI**	
e_gw1.444.23.1|Capca1	Cdx.Capca
estExt_Genewise1.C_7600007|Capca1	Gsx.Capca
fgenesh1_pg.C_scaffold_444000011|Capca1	Xlox.Capca
estExt_Genewise1.C_sca_850005|Lotgi1	Cdx.Lotgi
gw1.85.148.1|Lotgi1	Cdx.Lotgi
gw1.80.268.1|Lotgi1	Xlox.Lotgi
gw1.80.263.1|Lotgi1	Gsx.Lotgi
**SpBase:http://www.spbase.org/SpBase/**	
GLEAN3_26099	Splox.STRPU
GLEAN3_13436	Gsx.STRPU
GLEAN3_24715	Cdx.STRPU
